# Rational design of 2*H*-chromene-based antiphytovirals that inhibit virion assembly by outcompeting virus capsid-RNA interactions

**DOI:** 10.1016/j.isci.2024.111210

**Published:** 2024-10-18

**Authors:** Xiong Yang, Deguo Liu, Chunle Wei, Jianzhuan Li, Chunni Zhao, Yanping Tian, Xiangdong Li, Baoan Song, Runjiang Song

**Affiliations:** 1 National Key Laboratory of Green Pesticide, Key Laboratory of Green Pesticide and Agricultural Bioengineering, Ministry of Education, Center for R & D of Fine Chemicals of Guizhou University, Guiyang City, Guizhou Province 550025, P.R. China; 2 College of Plant Protection, Shandong Agricultural University, Tai’an City, Shandong Province 271018, P.R. China

**Keywords:** Chemistry, Biochemistry, Virology

## Abstract

Although the determination of the structural basis of potato virus Y (PVY) coat protein (CP) provides the possibility for CP-based antiviral drug design, the role of many specific residues on CP in regulating virion pathogenicity is largely unknown, and fewer small-molecular drugs have been discovered to act on these potential sites. In this study, a series of derivatives of 2,2-dimethyl-2*H*-chromene are rationally designed by employing a molecular hybridization strategy. We screen a case of phytovirucide **C50** that could form a stable H-bond with Ser^125^ of PVY CP to exert antiviral properties. Ser^125^ is further identified to be crucial for CP-viral RNA (vRNA) interaction, enabling PVY virion assembly. This interaction can be significantly inhibited through competitive binding with compound **C50**. The study enhances our understanding of anti-PVY drug mechanisms and provides a basis for developing new CP-targeting virus particle assembly inhibitors.

## Highlights

• Discovery of 2*H*-chromene derivatives with potent inhibitory activity against PVY
• Constructed 3D-QASR models of 2*H*-chromene derivatives to guide compound optimization
• Discovery of inhibitors for PVY particle assembly based on the PVY CP conserved region
• Point mutation strategy to validate the effect of potential sites on PVY function

## Graphical abstract

**Figure:**
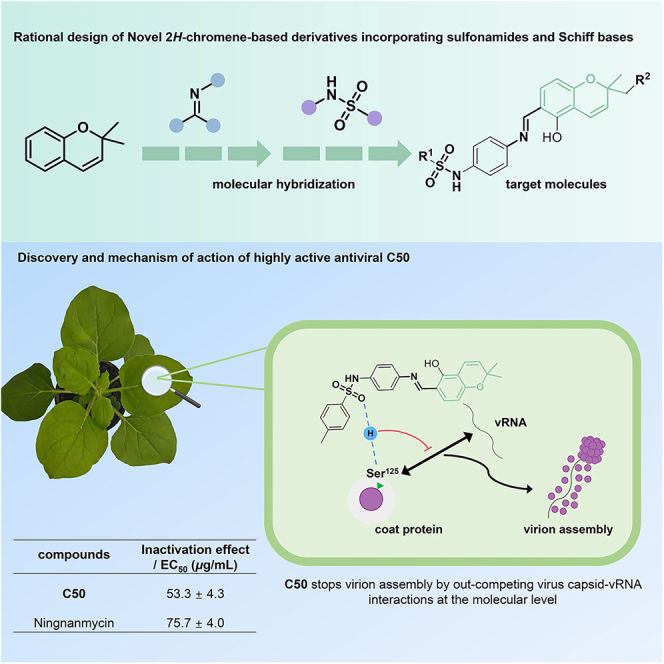

## Introduction

**Figure 1:**
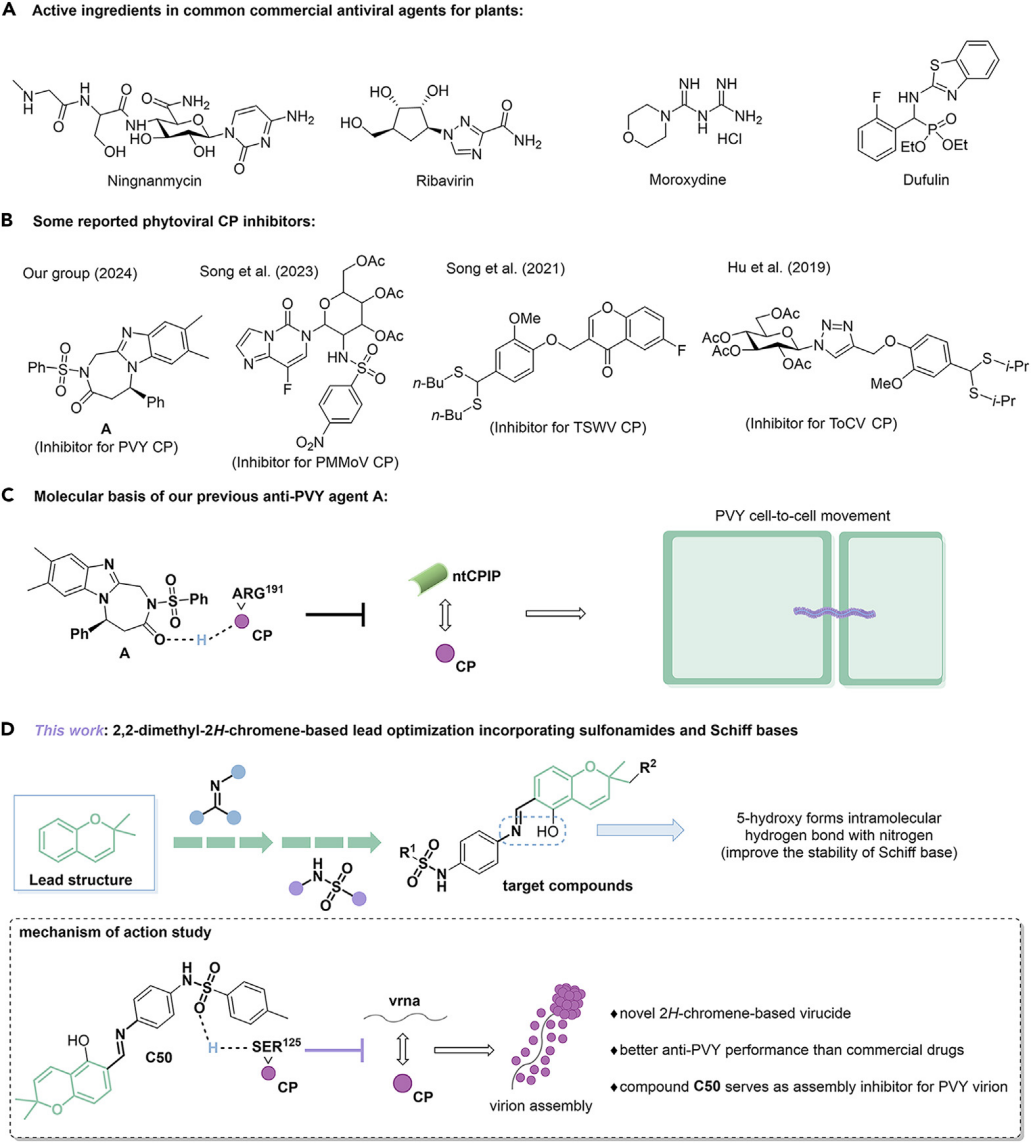
Rational design of 2*H*-chromene-based antiphytovirals (A) Active ingredients in common commercial antiviral agents for plants. (B) Some reported phytoviral CP inhibitors. (D) 2.2-dimethyl-2*H*-chromene-based lead optimization incorporating sulfonamides and Schif bases. (C) Molecular basis of our previous anti-PVY agent A.

Potato (*Solanum tuberosum* L.) is one of the crucial essential staple foods on which humanity relies for survival. More than 150 countries and regions worldwide cultivate potato, which serves food, vegetable, and feed functions and has excellent potential for further processing, playing an essential role in ensuring food security.^1^^,^^2^^,^^3^ However, plant pests and diseases can lead to crop failure, seriously restricting agricultural production.^4^^,^^5^^,^^6^ A notable instance of damaging pests encompasses potato virus Y (PVY), a linear RNA-based pathogen categorized within the *Potyviridae* family. Since it was initially reported in 1931, PVY had caused significant damage to crop yields by infecting various cash crops from the *Solanaceae* family, tobacco (*Nicotiana tabacum* L.) and potato being a prominent example.^7^^,^^8^^,^^9^ Currently, managing such viral pests by agrochemicals serves as the most direct route, but there were yet satisfactory results from the exploration of commercial drugs like ningnanmycin (NNM), ribavirin, and moroxydine for the control of PVY-induced disease (Figure 1A), which in turn sparked considerable interest in the development of new molecular entities for PVY management.^10^^,^^11^^,^^12^

The coat protein (CP) plays multiple roles in the PVY life cycle, including virus particle assembly, virus movement, and aphid transmission, etc.^13^ Consequently, CP is considered a promising candidate for biochemical targeting in developing new pesticides with antiviral properties that bind to CP, thereby suppressing virus infection.^14^ Indeed, CP has emerged as an ideal drug target and has been widely utilized in developing antiviral agents against plant viruses. Many CP inhibitors with diverse chemical structures and activities against various plant viruses have been reported (Figure 1B).^15^^,^^16^^,^^17^^,^^18^ Despite identifying critical sites on the CP that significantly mediate viral pathogenicity, a deeper understanding of molecular interactions remains lacking. Recently, we confirmed that chiral arylimidazole-fused compound **A** could competitively bind to the R^191^ site of PVY CP, interfering with the mutual effect between CP and the host factor ntCPIP at the molecular level, thereby preventing virions from moving across plant cells (Figure 1C).^15^ Therefore, it is essential to identify additional key sites that significantly influence viral behavior and elucidate the molecular mechanisms of drugs, providing a basis for developing effective and sustainable pest management strategies.

In 2019, Podobnik and co-workers reported the structural basis of PVY CP, which demonstrated that the interaction between viral RNA (vRNA) and CP is important for virion to maintain a stable helical configuration.^13^ This pioneering work provides opportunities for the design of antiviral agents based on PVY CP. It has been suggested that the conserved residues Ser^125^, Arg^157^, and Asp^201^ of PVY CP are related to the binding of vRNA. Hence, using drugs to interfere with these sites may hinder the interaction between CP and vRNA, thereby affecting downstream pathogenic behaviors. Moreover, because these sites belong to conserved regions, this enables the broad spectrum of the drug and makes it less likely to develop drug resistance. Unfortunately, no molecules have yet been discovered to enter this cavity and bind to its sites.

Here, we disclose a case of phytovirucide that could form a stable H-bond with Ser^125^ of PVY CP to exert antiviral properties (Figure 1D). The present investigation explores a range of innovative compound derivatives of 2,2-dimethyl-2*H*-chromene that were designed by using a molecular hybridization strategy. Notably, these target compounds bear a unique Schiff base unit and a phenolic hydroxyl group, which introduces additional hydrogen bond donors. This inclusion facilitates the formation of intramolecular hydrogen bonds with nitrogen atoms in neighboring Schiff base units, creating a six-membered ring and enhancing structural stability, as confirmed by X-ray diffraction results (CCDC: 2297246). Many of our synthesized derivatives displayed potent curative, protective, and inactivating effects against PVY during bioactivity assays. Among them, compound **C50**, identified through constructing a three-dimensional quantitative structure-activity relationship (3D-QSAR) model, demonstrated significantly better inactivation effects (EC_50_ = 53.3 μg/mL) compared to the commercial drug NNM (EC_50_ = 73.7 μg/mL). Combining molecular docking, molecular dynamics simulation, and a range of biological experiments allowed us to discover that compound **C50** can specifically bind to the conserved residue Ser^125^ on CP. We confirmed that this site is crucial for the interaction between vRNA and CP, which can be competitively inhibited by our drug, leading to dysfunction in the assembly of virus particles. Our research enhances knowledge of the mode of action of PVY-targeting drugs and lays the groundwork for designing innovative inhibitors of virus CP assembly at the molecular level.

## Results

### Chemistry

**Figure 2:**
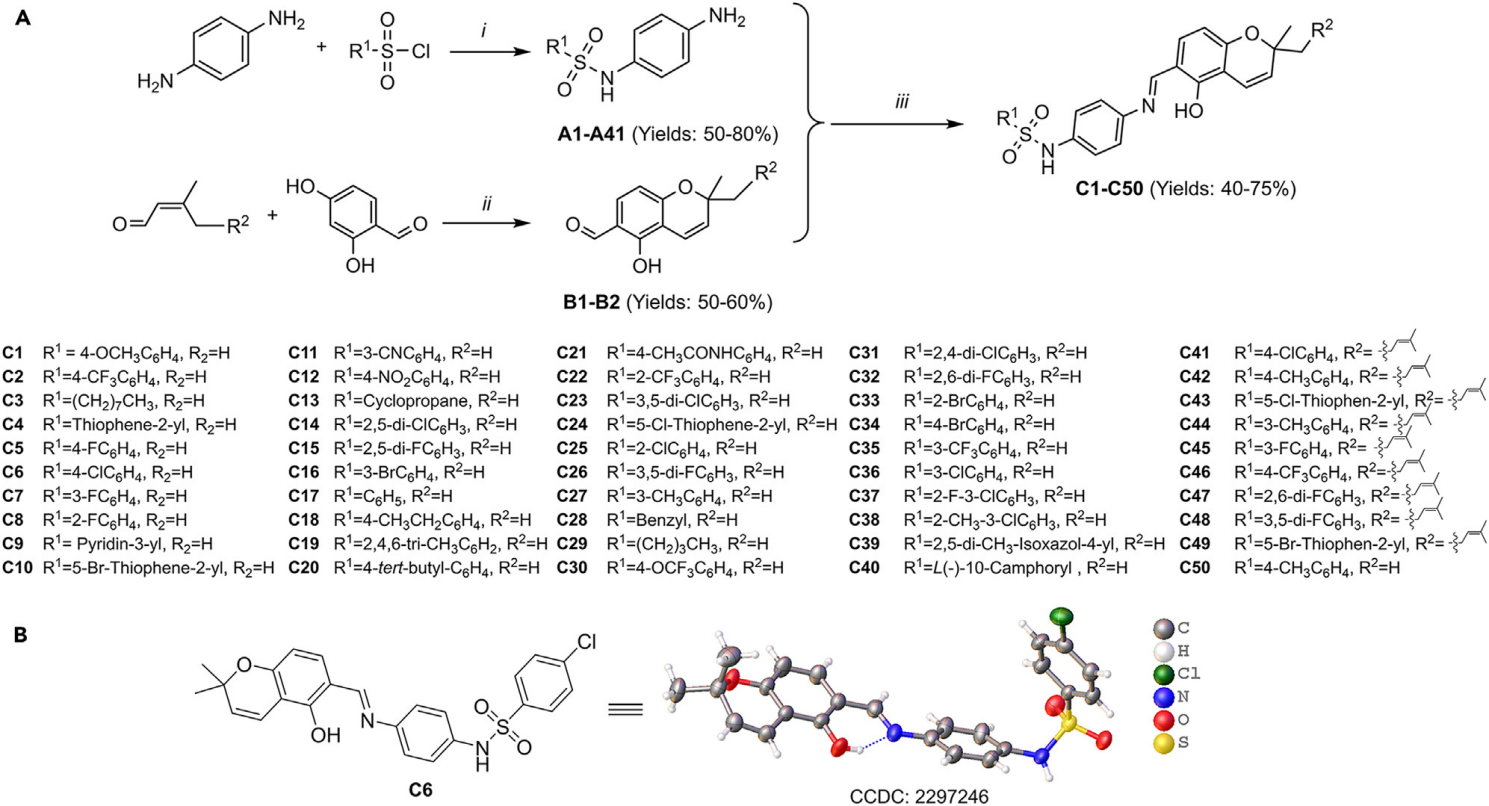
Production of the intended compounds (A) Manufacturing pathways for the target molecules. Reagent and conditions: (*i*) Et_3_N, DCM, 0°C 30 min, then at rt 3 h, 50%–80% yield; (*ii*) Et_3_N, CaCl_2_, EtOH, reflux, 3 h, 50%–60% yield; (*iii*) cat. AcOH, EtOH, reflux, 5 h, 40%–75% yield. (B) X-ray crystal structure of compound **C6** (CCDC: 2297246).

Firstly, *p*-phenylenediamine and substituted sulfonyl chloride were reacted in dichloromethane solvent, utilizing triethylamine as an acid scavenger, to synthesize intermediates designated as **A1**–**A41**; secondly, 2,4-dihydroxybenzaldehyde was used as raw materials with 3-methyl-2-butene or citral, and refluxed in an ethanol solution of triethylamine and anhydrous calcium chloride, and carried out the ring-combining reaction to acquire intermediates **B1** and **B2**. Finally, **A1**–**A41** and **B1** and **B2** were used as raw materials and heated to reflux with ethanol as a solvent to obtain 2,2-dimethyl-2*H*-chromene derivatives **C1**–**C50** with sulfonamide units (Figure 2A). As X-ray diffraction results showed, the hydroxyl group at the 5-position on the chromene ring of compound **C6** formed an intramolecular hydrogen bonding interaction with the nitrogen atom on the imine unit at the 6-position. This force formed a six-membered ring structure, which led to a more stable structure of the synthesized title compounds of the salicylaldehyde Schiff base class (Figure 2B). It is clear that the chemical properties of this class of compounds fully exhibit the *E* configuration.

### Assay anti-PVY activity

**Figure 3:**
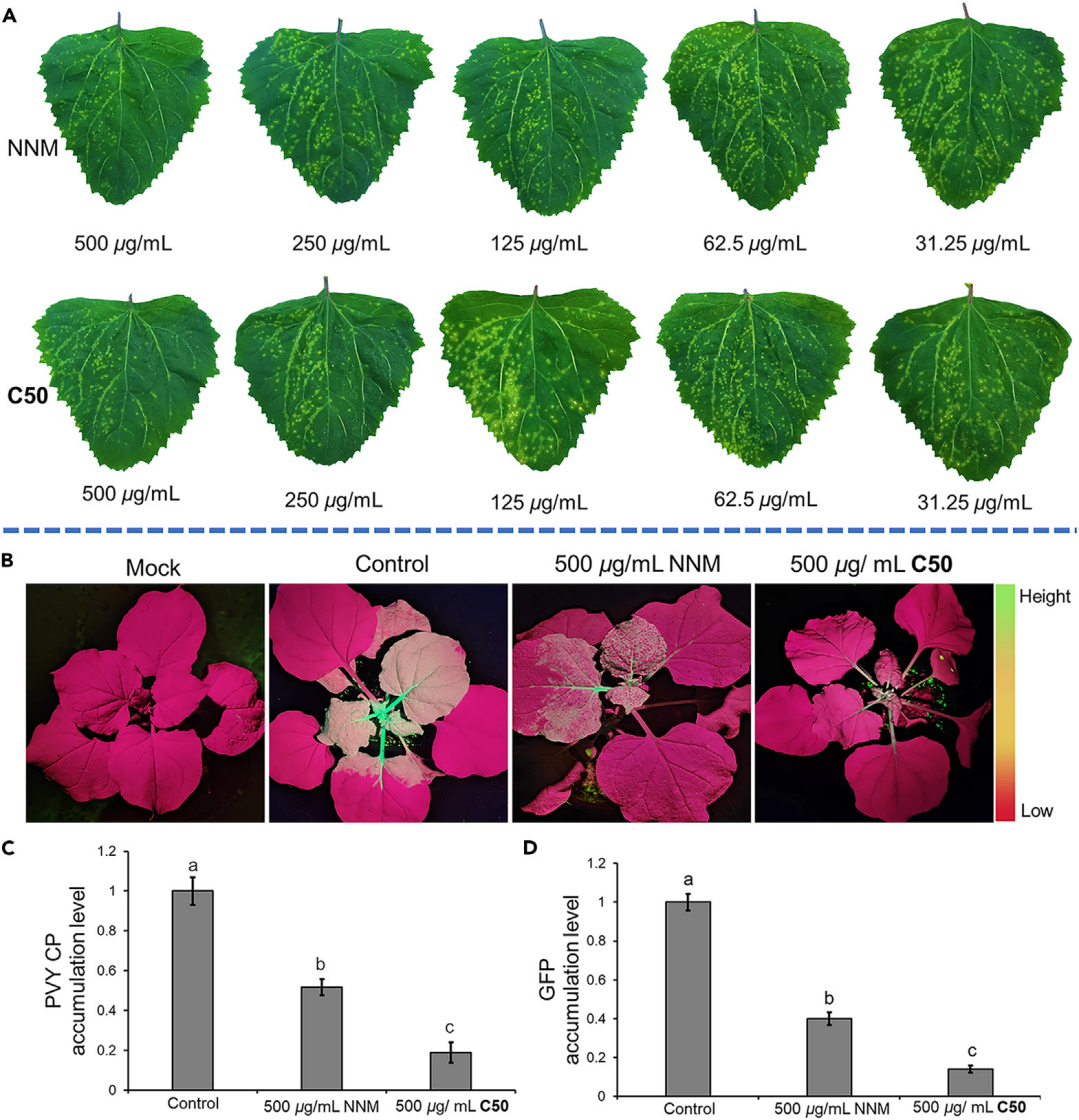
Effect of C50 against PVY infection (A) Effect of **C50** against PVY infection in *Chenopodium amaranticolor*. Ningnanmycin (NNM) was used as a control. The concentration was marked below the inoculated leaves. (B) Green fluorescence expression map of *Nicotiana benthamiana* leaf blades treated with PVY-GFP infectious **C50** and NNM under UV illumination. (C and D) Quantitative assessment of PVY CPand GFP accumulation in systemically infected leaves of *N. benthamiana* plants at 7 days post-agroinfiltration (dpai), utilizing RT-qPCR method. After injection of Agrobacterium with pCamPVY-GFP, a solution of 1% Tween 80 containing **C50** (500 μg/mL), NNM (500 μg/mL), and DMSO (as a control) was sprayed on the leaves of *N. benthamiana*. RT-qPCR normalization was achieved using EF1*α* as an internal control. Data are presented as mean ± SD from three biological replicates per treatment, with statistical significance indicated by different letters (*p* < 0.05, one-way ANOVA).

Utilizing the half-leaf blight method with *Chenopodium amaranticolor* as the indicator host and NNM as the positive control, the anti-PVY efficacy of all target compounds at a concentration of 500 μg/mL was assessed and tabulated in Table 1. The target compounds exhibited notable to superior antiviral potency toward PVY, with 13 of the target compounds (**C7**, **C10**, **C16**, **C24**, **C26**, **C27**, **C28**, **C29**, **C32**, **C34**, **C36**, **C37**, **C38**, and **C50**) having curative activities and protective activities in the range of 60%–70%, significantly higher than those of the commercial agent NNM (50.1%, 50.3%). Compounds **C6**, **C26**, **C27**, **C32**, **C40**, and **C50** showed comparable or better activity than NNM for inactivating effect. Based on the excellent inactivating performance of this group of compounds (inhibition rate around 80%), we determined the EC_50_ values of inactivating activity for all target compounds. We found that target compounds **C6** and **C50** showed better inactivating EC_50_ values of 56.7 and 53.3 μg/mL compared to the control agent NNM (73.7 μg/mL). The inactivating property of compound **C50** at different concentrations is shown in Figure 3A. NNM serves as the positive control, whereas the negative control is positioned on the left flank of the leaf. Given the excellent inactivating activity of **C6** and **C50**, we next studied the preliminary mechanism behind this.

**Table 1:** Antiviral efficacies of target compounds directed against PVY

Compd.		Curative activity (%)^a^	Protective activity (%)^a^	Inactivating activity (%)^a^	EC_50_ for inactivating activity (μg/mL)
**C1**	R^1^ = 4-OCH_3_C_6_H_4_, R^2^ = H	50.7 ± 5.5	62.2 ± 6.1	55.1 ± 6.0	441.2 ± 5.2
**C2**	R^1^ = 4-CF_3_C_6_H_4_, R^2^ = H	63.1 ± 5.8	58.9 ± 3.5	71.5 ± 3.5	255.7 ± 0.72
**C3**	R^1^ = (CH_2_)_7_CH_3_, R^2^ = H	12.3 ± 2.1	39.4 ± 3.7	40.1 ± 3.0	605.1 ± 12.2
**C4**	R^1^ = Thiophene-2-yl, R^2^ = H	28.1 ± 1.7	29.3 ± 2.9	38.1 ± 3.1	667.3 ± 20.1
**C5**	R^1^ = 4-FC_6_H_4_, R^2^ = H	70.3 ± 5.8	63.0 ± 1.6	66.7 ± 6.4	165.3 ± 8.7
**C6**	R^1^ = 4-ClC_6_H_4_, R^2^ = H	63.1 ± 2.1	57.3 ± 3.5	80.0 ± 1.9	56.7 ± 4.2
**C7**	R^1^ = 3-FC_6_H_4_, R^2^ = H	73.7 ± 2.6	70.2 ± 2.6	74.9 ± 2.4	100.3 ± 5.7
**C8**	R^1^ = 2-FC_6_H_4_, R^2^ = H	67.7 ± 2.0	58.4 ± 3.7	58.9 ± 2.6	369.2 ± 9.9
**C9**	R_1_ = Pyridin-3-yl, R^2^ = H	30.4 ± 2.9	32.7 ± 3.5	60.1 ± 2.7	310.2 ± 8.6
**C10**	R^1^ = 5-Br-Thiophene-2-yl, R^2^ = H	68.1 ± 3.0	63.3 ± 4.4	50.2 ± 2.4	492.3 ± 13.3
**C11**	R^1^ = 3-CNC_6_H_4_, R^2^ = H	29.1 ± 6.1	26.7 ± 3.7	44.2 ± 4.6	703.3 ± 25.3
**C12**	R^1^ = 4-NO_2_C_6_H_4_, R^2^ = H	31.8 ± 3.9	31.9 ± 3.9	58.9 ± 4.9	400.3 ± 7.5
**C13**	R^1^ = Cyclopropane, R^2^ = H	33.4 ± 3.5	27.8 ± 3.5	49.1 ± 3.1	531.2 ± 20.7
**C14**	R^1^ = 2,5-di-ClC_6_H_3_, R^2^ = H	31.2 ± 4.1	28.6 ± 2.4	35.2 ± 2.8	751.1 ± 19.4
**C15**	R^1^ = 2,5-di-FC_6_H_3_, R^2^ = H	54.1 ± 2.8	54.6 ± 3.1	30.7 ± 2.7	903.2 ± 14.3
**C16**	R^1^ = 3-BrC_6_H_4_, R^2^ = H	61.2 ± 2.8	63.4 ± 3.1	41.1 ± 1.5	688.4 ± 17.2
**C17**	R^1^ = C_6_H_5_, R^2^ = H	56.8 ± 5.6	61.2 ± 2.9	74.3 ± 1.8	168.4 ± 4.8
**C18**	R^1^ = 4-CH_3_CH_2_C_6_H_4_, R^2^ = H	57.4 ± 4.3	59.0 ± 2.9	79.1 ± 1.2	202.1 ± 3.8
**C19**	R^1^ = 2,4,6-tri-CH_3_-C_6_H_2_, R^2^ = H	25.8 ± 2.6	58.7 ± 3.5	56.7 ± 1.9	492.3 ± 11.2
**C20**	R^1^ = 4-*tert*-butylC_6_H_4_, R^2^ = H	32.4 ± 5.8	24.5 ± 2.2	20.1 ± 2.5	1,003.4 ± 18.2
**C21**	R^1^ = 4-CH_3_CONHC_6_H_4_, R^2^ = H	25.7 ± 2.6	22.7 ± 2.9	33.5 ± 2.1	1,021.3 ± 20.7
**C22**	R^1^ = 2-CF_3_C_6_H_4_, R^2^ = H	50.2 ± 2.9	53.7 ± 2.0	45.7 ± 2.0	587.0 ± 9.9
**C23**	R^1^ = 3,5-di-ClC_6_H_3_, R^2^ = H	19.1 ± 3.2	28.0 ± 2.7	23.4 ± 1.4	870.9 ± 17.3
**C24**	R^1^ = 5-Cl-Thiophene-2-yl, R^2^ = H	67.6 ± 3.1	65.8 ± 2.9	34.5 ± 2.3	753.6 ± 10.3
**C25**	R^1^ = 2-ClC_6_H_4_, R^2^ = H	56.8 ± 5.0	70.9 ± 4.0	67.3 ± 2.7	243.9 ± 10.2
**C26**	R^1^ = 3,5-di-FC_6_H_3_, R^2^ = H	65.2 ± 2.0	63.7 ± 2.9	81.3 ± 3.8	100.3 ± 4.5
**C27**	R^1^ = 3-CH_3_C_6_H_4_, R^2^ = H	63.5 ± 3.0	68.7 ± 3.5	81.8 ± 3.3	90.3 ± 7.1
**C28**	R^1^ = Benzyl, R^2^ = H	64.4 ± 3.3	68.9 ± 2.3	47.5 ± 1.2	503.1 ± 12.8
**C29**	R^1^ = (CH_2_)_3_CH_3_, R^2^ = H	64.2 ± 2.3	66.6 ± 4.5	35.9 ± 5.0	682.1 ± 10.0
**C30**	R^1^ = 4-OCF_3_C_6_H_4_, R^2^ = H	31.0 ± 2.6	39.6 ± 6.2	32.2 ± 4.7	834.1 ± 18.3
**C31**	R^1^ = 2,4-di-ClC_6_H_3_, R^2^ = H	29.1 ± 2.4	39.7 ± 6.2	35.6 ± 4.7	975.7 ± 20.0
**C32**	R^1^ = 2,6-di-FC_6_H_3_, R^2^ = H	66.8 ± 2.2	63.4 ± 6.0	80.9 ± 3.6	88.3 ± 4.0
**C33**	R^1^ = 2-BrC_6_H_4_, R^2^ = H	52.1 ± 0.6	34.6 ± 3.6	78.1 ± 5.1	139.2 ± 3.2
**C34**	R^1^ = 4-BrC_6_H_4_, R^2^ = H	66.4 ± 2.9	65.2 ± 5.3	79.5 ± 3.9	147.3 ± 8.3
**C35**	R^1^ = 3-CF_3_C_6_H_4_, R^2^ = H	48.6 ± 2.6	64.3 ± 4.2	29.2 ± 3.3	803.0 ± 12.3
**C36**	R^1^ = 3-ClC_6_H_4_, R^2^ = H	68.5 ± 4.9	61.0 ± 2.3	44.4 ± 2.0	557.2 ± 14.3
**C37**	R^1^ = 2-F-3-ClC_6_H_3_, R^2^ = H	63.1 ± 3.5	60.2 ± 2.3	59.5 ± 4.2	306.3 ± 11.0
**C38**	R^1^ = 2-CH_3_-3-ClC_6_H_3_, R^2^ = H	65.5 ± 3.0	61.1 ± 3.1	64.0 ± 3.3	410.3 ± 9.7
**C39**	R^1^ = 2,5-di-CH_3_-Isoxazol-4-yl, R^2^ = H	41.0 ± 2.7	47.8 ± 4.4	63.1 ± 3.1	397.1 ± 2.3
**C40**	R^1^ = *L*(−)-10-Camphoryl, R^2^ = H	60.2 ± 3.8	53.2 ± 1.8	81.2 ± 1.8	204.3 ± 7.1
**C41**	R^1^ = 4-ClC_6_H_4_, R^2^ =	34.2 ± 4.7	36.9 ± 2.2	36.5 ± 1.5	780.8 ± 15.3
**C42**	R^1^ = 4-CH_3_C_6_H_4_, R^2^ =	38.8 ± 3.3	44.5 ± 3.4	52.2 ± 3.5	503.2 ± 17.0
**C43**	R^1^ = 5-Cl-Thiophene-2-yl, R^2^ =	33.1 ± 3.7	40.1 ± 2.8	39.7 ± 3.8	512.3 ± 11.0
**C44**	R^1^ = 3-CH_3_C_6_H_4_, R^2^ =	29.7 ± 6.3	40.0 ± 1.7	34.0 ± 2.8	881.7 ± 17.3
**C45**	R^1^ = 3-F C_6_H_4_, R^2^ =	32.1 ± 1.4	42.4 ± 2.4	32.7 ± 3.3	515.9 ± 9.0
**C46**	R^1^ = 4-CF_3_C_6_H_4_, R^2^ =	33.5 ± 2.1	41.1 ± 3.8	49.4 ± 3.1	522.4 ± 8.1
**C47**	R^1^ = 2,6-di-FC_6_H_3_，R^2^ =	30.7 ± 4.9	37.1 ± 2.7	44.7 ± 1.2	629.5 ± 11.2
**C48**	R^1^ = 3,5-di-FC_6_H_3_, R^2^ =	34.7 ± 1.1	31.9 ± 2.7	23.3 ± 2.2	716.6 ± 17.8
**C49**	R^1^ = 5-Br-Thiophene-2-yl, R^2^ =	36.2 ± 1.1	39.4 ± 2.6	40.4 ± 3.3	552.7 ± 9.3
**C50**	R^1^ = 4-CH_3_C_6_H_4_, R^2^ = H	68.7 ± 3.8	70.1 ± 1.7	84.1 ± 1.0	53.3 ± 4.3
NNM^b^	/	50.1 ± 2.9	50.7 ± 1.8	82.5 ± 1.3	75.7 ± 4.0

a Average of three replicates.

b Ningnanmycin (NNM) used as positive control.

### Compound C50 inhibits the systemic infection of PVY

To evaluate the effect of **C50** on PVY infection, we infiltrated the *Nicotiana benthamiana* plants with Agrobacterium carrying the PVY infectious clone pCamPVY-GFP. As depicted in Figure 3B, a notable presence of strong green fluorescence was observed in the stems and apical shoot leaves of the control group (DMSO, 500 μg/mL). On the other hand, there was a noteworthy decrease in green fluorescence observed in both the **C50** (500 μg/mL) and NNM (500 μg/mL) treatment cohorts, with a more substantial reduction observed in the **C50** treatment cohort. Reverse-transcription quantitative real-time PCR (RT-qPCR) analysis revealed that, as opposed to the control group (in which PVY RNA accumulation was normalized as 100%), the PVY RNA accumulation level in **C50** and NNM-treated *N. benthamiana* plant was significantly reduced to 18.9% and 51.8%, respectively, in the systemic leaves of the infected *N. benthamiana* leaves (Figure 3C). The RT-qPCR results for GFP were consistent with that of PVY RNA (Figure 3D). These results showed that **C50** is a potential antiviral agent against PVY.

### 3D-QSAR studies

**Figure 4:**
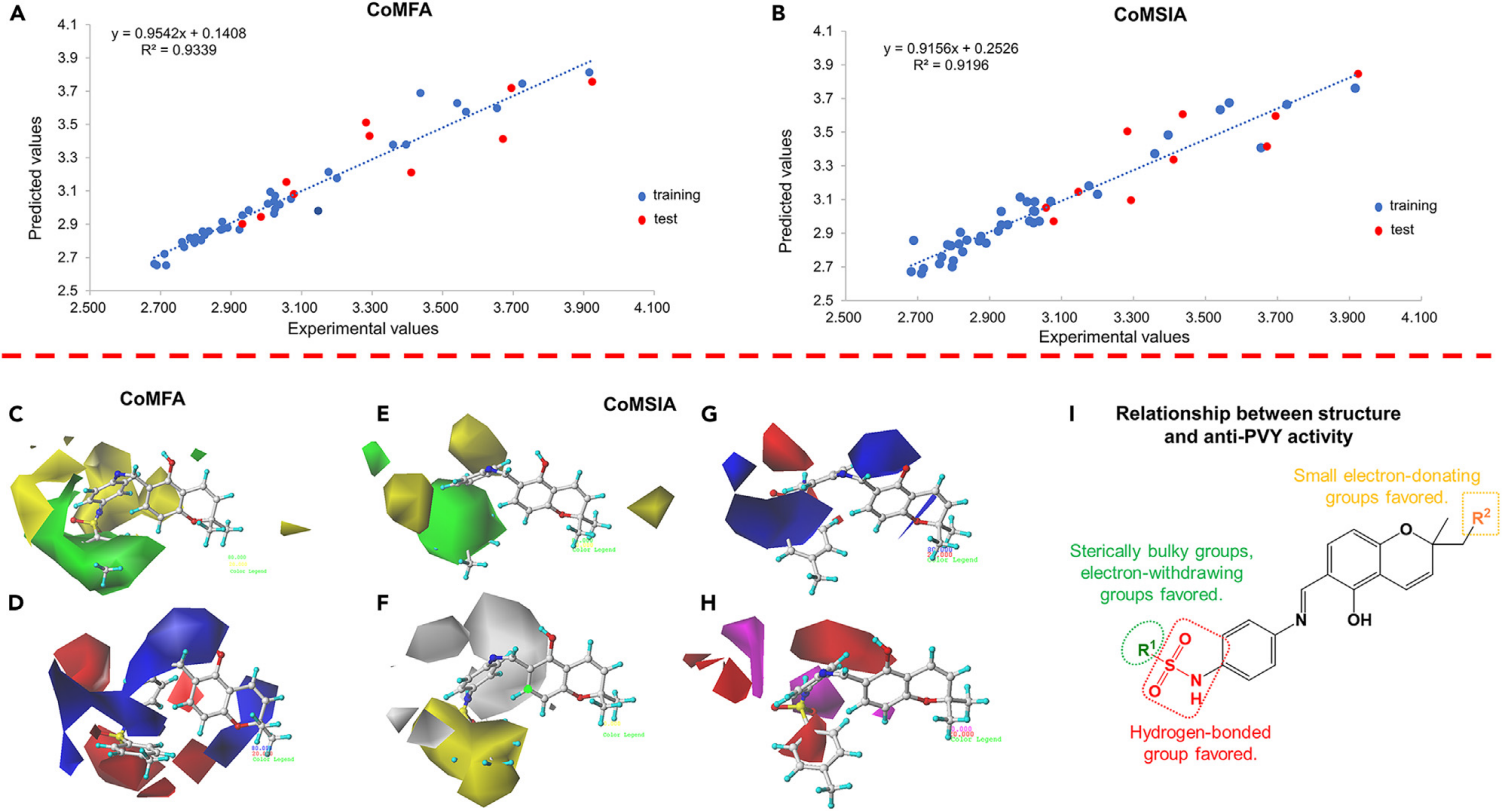
3D-QSAR analysis (A) CoMFA and (B) CoMSIA models for comparing experimental versus predicted pEC_50_. (C and D) CoMFA 3D isopotential maps illustrating (C) steric and (D) electrostatic contributions. (E–H) CoMSIA 3D isopotential maps depicting (E) steric, (F) electrostatic, (G) hydrophobic, and (H) hydrogen bond acceptor fields. (I) Relationship between structure and anti-PVY activity.

Using compound **C6** as a template, we aligned compounds in the training set and developed comparative molecular field analysis (CoMFA) and comparative molecular similarity index analysis (CoMSIA) models incorporating the anti-PVY inactivity of the targets. The parameters of these models were then scrutinized to assess their reliability. Table 2 displays the q^2^, *r*^2^, standard error of estimate (SEE), and F values of the built CoMFA and CoMSIA models. Both models’ q^2^, *r*^2^, and F values exhibited more significance than 0.5, 0.8, and 100, respectively. Additionally, the SEE values were comparatively less, suggesting that the built models possess predictive capabilities. According to the data in Figures 4A and 4B and Table S1, the experimental and theoretical pEC_50_ values closely matched. The residuals in the analysis had absolute values below 0.3. The linear correlation coefficients for experimental vs. predicted pEC_50_ were high, at 0.9339 for the CoMFA and 0.9196 for the CoMSIA. These findings provide further evidence that both CoMFA and CoMSIA models effectively forecast the anti-PVY activity of the compounds.

**Table 2:** Statistical outcomes belong to the CoMFA and CoMSIA models

Statistical parameter	CoMFA	CoMSIA	Validation criterion
q^2^	0.602	0.619	>0.5
ONC	5	4	–
*r*^2^	0.984	0.936	>0.8
SEE	0.042	0.081	–
F	408.575	127.810	–

**Fraction of field contributions**			

Steric	0.505	0.106	–
Electrostatic	0.495	0.164	–
Hydrophobic	–	0.221	–
Hydrogen-bond donor	–	0.349	–
Hydrogen-bond acceptor	–	0.160	–

The CoMFA model showed nearly equal contributions from steric (50.5%) and electrostatic (49.5%) fields, emphasizing their joint importance for anti-PVY activity. Both factors should be addressed when optimizing compounds. Based on the CoMFA model, it can be seen that the position of R^1^ is mainly a green module (Figure 4C), indicating that increasing the spatial site resistance of R^1^ is advantageous to the anti-PVY inactivating activity, in which substitution of the aromatic ring with a significant site resistance is better than that of the aliphatic or cycloalkanes with a small site resistance, such as **C7** (R^1^ = 3-FC_6_H_4_, R^2^ = H, EC_50_ = 100.3 μg/mL) < **C29** (R^1^ = (CH_2_)_3_CH_3_, R^2^ = H, EC_50_ = 682.1 μg/mL). Positions are mainly yellow modules, indicating that increasing the spatial site resistance of R^2^ is detrimental to anti-PVY inactivating activity, such as **C41** (R^1^ = 4-ClC_6_H_4_, R^2^ = CH_2_CHC(CH_3_)_2_, EC_50_ = 780.8 μg/mL) > **C6** (R^1^ = 4-ClC_6_H_4_, R^2^ = H, EC_50_ = 56.7 μg/mL), **C42** (R^1^ = 4-CH_3_C_6_H_4_, R^2^ = CH_2_CHC(CH_3_)_2_, EC_50_ = 503.2 μg/mL) > **C50** (R^1^ = 4-CH_3_C_6_H_4_, R^2^ = H, EC_50_ = 53.3 μg/mL), and **C48** (R^1^ = 3,5-di-FC_6_H_3_, R^2^ = CH_2_CHC(CH_3_)_2_, EC_50_ = 716.6 μg/mL) > **C26** (R^1^ = 3,5-di-FC_6_H_3_, R^2^ = H, EC_50_ = 100.3 μg/mL). In CoMFA’s electrostatic field (Figure 4D), R^1^’s dominant red module hints at enhanced anti-PVY activity with electron-withdrawing R^1^ groups. The position of R^2^ is mainly a blue module, indicating that introducing electron-donating groups in R^2^ can be conducive to raising the anti-PVY inactivating activity. In summary, the R^1^ substituent is an electron-withdrawing group, and the introduction of a large-site-resistance substituent is beneficial to increase the activity; the reduction of the spatial site resistance of the 2-position methyl group (R^2^) of the pyran ring and incorporating an electron-donating group boosts anti-PVY activity. Figures 4E and 4F depict the three-dimensional and electrostatic fields belonging to the CoMSIA model. Assessment of color block significance and roles yields conclusions mirroring those of the CoMFA model. The hydrophobic field of the CoMSIA model is displayed in Figure 4G. A yellow region surrounds R^1^, indicating that introducing hydrophobic groups promotes the anti-PVY activity of target compounds. In contrast, the white color does the opposite. Figure 4H shows the hydrogen bonding receptor field of the CoMSIA model, with a larger red module at the position of the sulfonamide group oxygen atom, suggesting that the hydrogen bonding receptor provided by the sulfonamide double-bonded oxygen favors the anti-PVY activity of the compounds.

The analysis conducted using the model aforementioned demonstrated that incorporating a sizable electron-withdrawing group at R^1^ and a small electron-donating group at R^2^ resulted in a favorable enhancement in the anti-PVY activity belonging to compounds under investigation. Furthermore, it was observed that the presence of a sulfonamide group played a crucial role as a hydrogen-bonding acceptor group (Figure 4I).

### Molecular docking and molecular dynamics simulation

**Figure 5:**
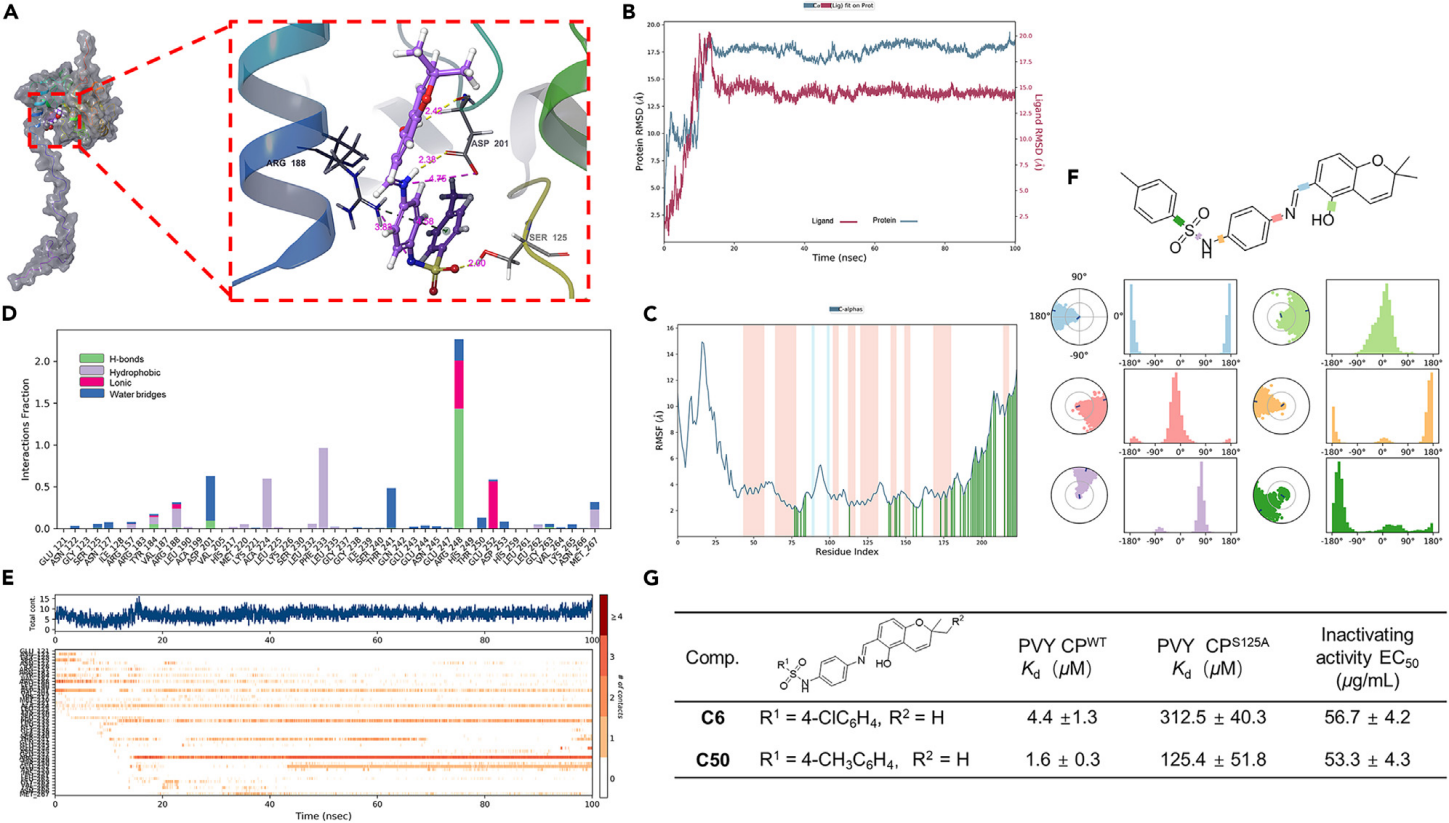
Ser^125^ is a key target for binding compound C50 to PVY CP (A) Computational binding analysis of **C50** to PVY CP using molecular docking techniques. (B–F) Comprehensive results of molecular dynamics simulations of **C50** and PVY CP. (B) RMSD analysis, (C) RMSF analysis, (D and E) interaction analysis, (F) ligand torsion diagram. (G) Analysis comparing the binding of PVY CP^wt^ and PVY CP^S125A^ mutant proteins to compounds.

To verify whether the compounds have interaction forces with PVY CP and to find their binding sites, compounds **C6** and **C50** were molecularly docked to PVY CP, respectively, employing ultra-precise XP docking under the Schrödinger software module. Detailed examination of XP docking and MM-GBSA outcomes revealed that compounds **C6** and **C50** had docking scores of −6.045 and −5.999 with PVY CP, and MM-GBSA results were −34.15 kcal/mol and −34.44 kcal/mol, with low docking scores and free energies of binding, which indicated that **C6** and **C50** were stable in binding to PVY CP. The molecular docking results showed that **C6** penetrated deep into the interior of the active pocket of PVY CP (Figure S1), developing one hydrogen bond with residue Ser^125^ of PVY CP with a bond length of 1.91 Å, two hydrogen bonds, and one salt bridge with residue Asp^201^ with bond lengths of 2.43, 2.34, and 4.70 Å, and one salt bridge and two π-cation bonds with residue Arg^188^ with bond lengths of 4.02, 3.70, and 4.63 Å. Compounds **C50** and **C6** are structurally distinguished only by methyl and chlorine atoms. The docking results are similar to the **C6** results, showcasing **C50**’s deep penetration into PVY CP’s active site. It forms a hydrogen bond with Ser^125^ (2.00 Å), two with Asp^201^, and a salt bridge (4.75, 2.42, and 2.38 Å). Additionally, it establishes a salt bridge and π-cation bond with Arg^188^, measuring 3.38 and 4.58 Å, respectively (Figure 5A). In summary, it can be seen that the compounds **C6** and **C50**, which are highly inactivating activity compounds for PVY, both form strong hydrogen bonding interactions forming hydrogen bonds with Ser^125^ of PVY CP at lengths of 1.91 and 2.00 Å.

Molecular dynamics trajectory simulations of **C50** with PVY CP were carried out for 100 ns, and then molecular dynamics trajectories were subsequently scrutinized. The conformational stability of root-mean-square deviation (RMSD) against simulation time is shown in the figure, where smaller fluctuations indicate that stable conformations were obtained for all the complexes. The analysis reveals a stable **C50**-PVY CP complex post-15 ns, indicating system equilibrium (Figure 5B). Root-mean-square fluctuation (RMSF) assesses local protein chain variations, highlighting peaks as areas experiencing the most significant fluctuations in the simulation. As shown in Figure 5C, the results showed that the protein displayed significant structural pliability within the residue region 12–35AA and 190–225AA after the binding of **C50** to PVY CP.

Throughout the simulation, protein-ligand engagements are tracked and classified into four primary types: H-bond, hydrophobic forces, ionic linkages, and aqueous bridges. Figure 5D illustrates that the pivotal amino acids crucial for **C50** binding to PVY CP protein are Ser^125^, Asn^127^, Asp^201^, Ala^224^, Phe^233^, and Arg^248^. Figure 5E demonstrates the interactions between the specific amino acids of **C50** and PVY CP in terms of time during the entire trajectory. The outcomes indicate that amino acid residues Ser^125^, Asp^201^, Ala^224^, Phe^233^, Thr^241^, Arg^248^, and Glu^252^ have multiple contacts with the ligand.

The ligand torsion diagram outlines the transformation of ligand conformations across rotatable bonds during the simulation path. The upper portion of Figure 5F depicts a 2D illustration of a ligand, featuring color-coded rotatable bonds. Each bond’s torsion is visually represented by a matching colored dial plot and bar graph. The dial plot in the bottom half of Figure 5F illustrates the torsion’s conformational changes during the simulation. The bar graph condenses the dial plot data, displaying the probability density distribution of the torsion.

The aforementioned results show that compound **C50** penetrated deep into the conserved folding region of PVY CP and interacted with amino acid residues such as Ser^125^ and Asp^201^. A strong hydrogen bonding interaction was formed with Ser^125^ suggesting that Ser^125^ may be the key site for binding 2*H*-chromene derivatives containing sulfonamide structure to PVY CP.

### Purification of the wild-type protein PVY CP^wt^ and the mutant protein PVY CP^S125A^ and microscale thermophoresis assay

PVY CP is involved in various biological functions and is pivotal in the life cycle of PVY. Hence, it is regarded as a promising candidate protein for antiphytoviral agent screening. The PVY CP^wt^ encoding sequence was constructed into the expression vector pET-32a (+) using whole-gene synthesis methodology. The resultant plasmid was named pET-PVY CP^wt^. The codon at position 125 was mutated to alanine based on the pET-PVY CP^wt^. The resultant plasmid was named pET-PVY CP^S125A^. The PVY CP^wt^ and its mutant CP^S125A^ were expressed and purified according to the previous protocol. SDS-PAGE results showed that an expected band of about 50 kDa was observed. After enterokinase digestion and His-tag purification resin, a specific band of about 33 kDa was observed (Figure S2). This result is consistent with the theoretical value.^13^

Microscale thermophoresis (MST) serves as a potent technique for quantifying the strength of interactions occurring between small molecules and their respective ligands.^19^^,^^20^ The binding strength between specific target drugs and PVY CP^wt^ was investigated *in vitro* using MST, as depicted in Figures 5G and S3. This experimental approach aimed to acquire insights into how target compounds exert their influences against PVY. The findings indicated that the binding strength of target compounds to PVY CP^wt^ was in line with their effectiveness in anti-PVY. The *K*_D_ values for binding of compounds **C6** and **C50** to PVY CP^wt^ were 4.4 and 1.6 μM, respectively, indicating that compounds **C6** and **C50** have a strong affinity for PVY CP, which is superior to NNM (*K*_D_ = 8.6 μM). Compound **C42** had low inactivating activity against PVY (EC_50_ = 503.2 μg/mL). Its binding to PVY CP^wt^ was also relatively weak (*K*_D_ = 497.1 μM). To further confirm that the serine at position 125 of the PVY CP protein is a key site of action for the binding of PVY by 2,2-dimethyl-2*H*-chromene derivatives containing a sulfonamide unit, the serine at position 125 of the PVY CP protein was fixed point mutated to an alanine called PVY CP^S125A^. Similarly, the affinity of target compounds for interacting with PVY CP^S125A^ was tested. The findings indicated a markedly diminished binding affinity between the target compounds and PVY CP^S125A^, such as the *K*_D_ value of compound **C50** was decreased from 1.6 to 125.4 μM. Among them, the weakening of binding between NNM and PVY CP^S125A^ was not very obvious, indicating that the serine at position 125 of PVY CP protein is not a key site for binding of NNM to PVY CP, which further indicates that this site is the critical site for anti-PVY of 2,2-dimethyl-2*H*-chromene derivatives containing sulfonamide unit.

### Effects of mutation at position Ser^125^ of CP on PVY accumulation levels

**Figure 6:**
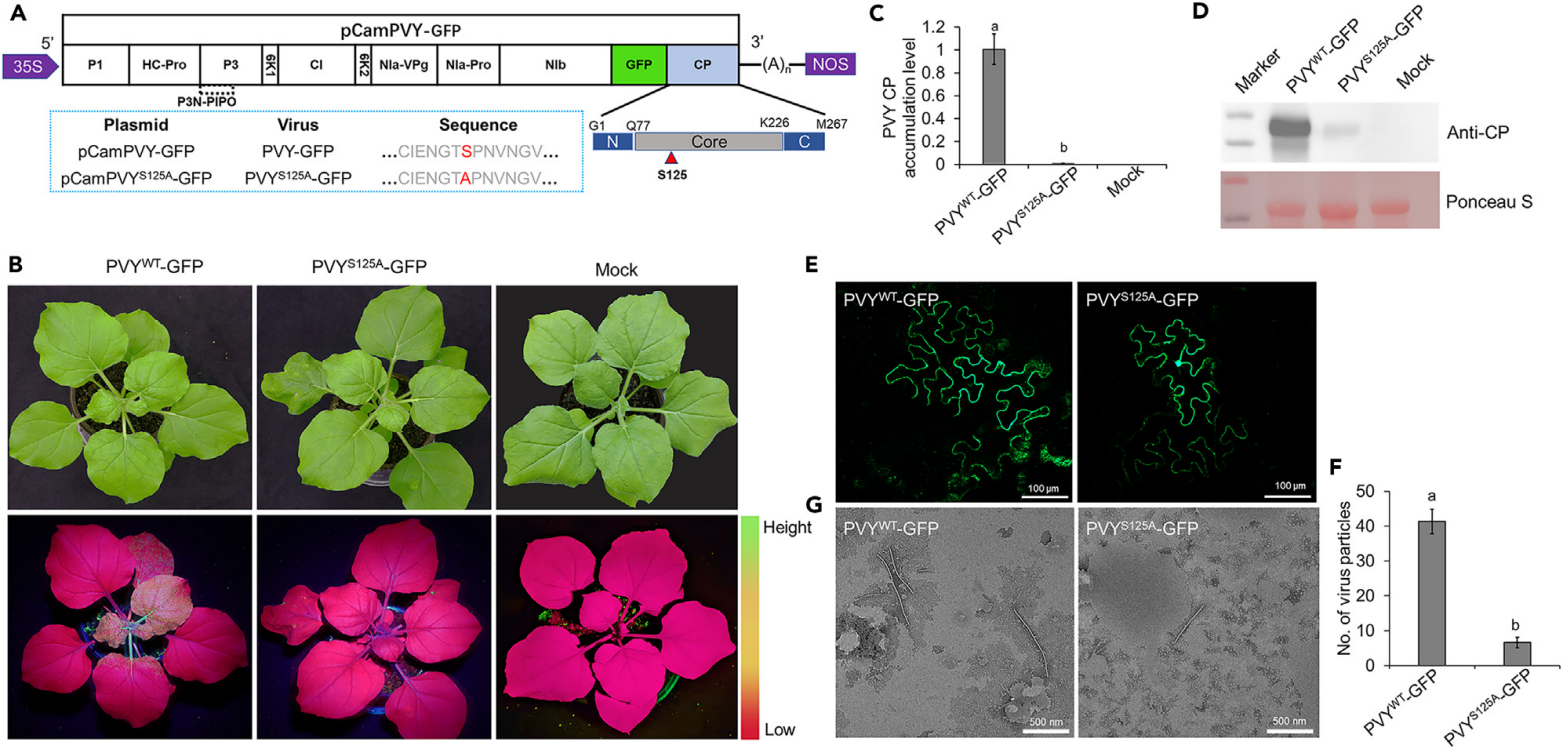
Effect of mutation on Ser^125^ in CP on PVY infection (A) Illustrative representation of the pCamPVY-GFP genome structure, highlighting the Ser^125^ residue (marked by red arrows) within the core domain of PVY CP. The blue-bordered box encapsulates the site-specific mutants, wild-type plasmids, viruses, and their respective sequences. (B) Comparative visualization of disease symptoms (top) and green fluorescent expression (bottom) under UV illumination in *N. benthamiana* leaves inoculated with wild-type and mutated PVY strains. (C and D) Quantitative assessment of PVY CP accumulation in systemically infected leaves of *N. benthamiana* plants at 7 dpai, utilizing RT-qPCR (C) and western blot (D) methods. RT-qPCR normalization was achieved using EF1*α* as an internal control, while Ponceau S-stained RuBisCO served as a loading control. Data are presented as mean ± SD from three biological replicates per treatment, with statistical significance indicated by different letters (*p* < 0.05, one-way ANOVA). (E) Examination of cell-to-cell movement dynamics in *N. benthamiana* plants infected with wild-type and mutated PVY strains at 3 dpai. (F) Enumeration of PVY particles within 70 μm^2^ microscopic fields, presented as mean ± SD derived from five fields per treatment. Statistical significance is denoted by different letters (*p* < 0.05, one-way ANOVA). (G) Particles of PVY-GFP and PVY^S125A^-GFP under transmission electron microscope.

To further validate the possibility of the residue Ser^125^ of CP function as a potential target site for the small molecule **C50** *in vivo*, we mutated the codon at position 125 in CP based on the infectious clone pCamPVY-GFP using site-directed mutagenesis; the resultant plasmid was named pCamPVY^S125A^-GFP (the produced virus named as PVY^S125A^-GFP) (Figure 6A). The plasmids pCamPVY-GFP and pCamPVY^S125A^-GFP were transformed into Agrobacterium GV3101 by freeze-thawing, respectively. Agrobacterium carrying those plasmids were infiltrated on the fully expanded leaves of 4- to 6-week-old *N. benthamiana* plants. At 7 days post-agroinfiltration (dpai), a strong green fluorescence was observed on the systemic leaves of *N. benthamiana* plants infiltrated with Agrobacterium carrying pCamPVY-GFP under UV light. However, no green fluorescence was observed on the *N. benthamiana* plants infiltrated with Agrobacterium cells carrying pCamPVY^S125A^-GFP (Figure 6B). Western blot and RT-qPCR analyses revealed a substantial accumulation of PVY CP in the systemic leaves of wild-type PVY GFP-infected *N. benthamiana* plants. In contrast, only minimal traces were detected in the corresponding leaves of PVY^S125A^-GFP-infected *N. benthamiana* (Figures 6C and 6D). In summary, PVY CP^S125^ significantly affected virus accumulation in *N. benthamiana*.

Virus particle assembly and movement are two critical processes for potyviruses infection. To test the effect of mutation on the Ser^125^ in CP on PVY cell-to-cell movement, we adjusted the OD_600_ value of Agrobacterium cells carrying pCamPVY-GFP and pCamPVY^S125A^-GFP to 0.5, and then further diluted 1,000 times. The diluted Agrobacterium cells were infiltrated on 4- to 6-week-old *N.benthamiana* plants. The cell-to-cell movement was observed using a laser confocal microscope at 3 dpai. Confocal microscopy analysis showed that PVY could move to multiple cells in the *N. benthamiana* plants inoculated with PVY-GFP. However, the number of cells displaying green fluorescence was much less in the *N. benthamiana* plants inoculated with PVY^S125A^-GFP (Figure 6E). The findings suggested that the PVY CP^S125^ mutation did not impair the cell-to-cell movement of PVY-GFP.

Based on the aforementioned experimental results, we found that the substitution of Ser^125^ with alanine in CP significantly affected the PVY accumulation in *N. benthamiana* plants but did not abolish its cell-to-cell movement. Disturbing the interaction between CP and vRNA reduces the stability of the virus particles. Previous studies have shown that Ser^125^ of CP binds vRNA.^13^ It is interesting to know whether the mutation on Ser^125^ affects the virus particle assembly. We purified the virus particles of PVY-GFP and PVY^S125A^-GFP, respectively, followed by the previously reported protocol.^15^ The stability of PVY virus particles is derived from many CP-vRNA interactions, and Ser^125^ serves as one of the sites where the PVY CP binds to the vRNA. Mutation of this polar amino acid residue (Ser) to the non-polar amino acid Ala may impact virus particle assembly. Therefore, we further observed the effect of PVY CP^S125^ on virus particle assembly by transmission electron microscopy. Wild-type PVY-GFP and mutant PVY CP^S125A^-GFP were infiltrated into leaves of age-appropriate *N. benthamiana*, and virus particles were collected for virus particle extraction at 5 dpai. The purified specimens were treated with 1% phosphotungstic acid staining, followed by morphological observation of the refined PVY virus particles via transmission electron microscopy. Intact PVY virus particles, curved linear particles of approximately 11 nm × 680–900 nm in size (Figure 6G), were observed in PVY-GFP and PVY-GFP^S125A^-treated leaves, which is in accordance with literature reports.^15^ Notably, the viral particle count in PVY^S125A^-GFP samples was markedly lower than that in PVY-GFP-infected counterparts (Figure 6F). Thus, PVY CP^S125^ is essential for forming PVY virus particles in plants.

## Discussion

Natural compounds serve as a vital resource in the pursuit of innovative antiviral medications. Among them, 2,2-dimethyl-2*H*-chromene is present in large quantities in nature and possesses a vast range of antiviral activities. Calanolide A, an extract from the tropical rainforest plant *Calophyllum lanigerum*, was one of the first natural products to be discovered with anti-HIV-1 activity.^21^ Recently, a new indole alkaloid, isoaspergilline A, has been isolated from the fermentation product of the tobacco endophyte fungus *Aspergillus versicolor* and has been shown to have excellent anti-TMV activity.^22^ Sulfonamide derivatives have always been the star structures in drug discovery and development, which increase the water solubility of drugs and provide additional hydrogen-bonded receptors. Previous work by our group has shown that sulfonamides have excellent antiphytoviral activity, and some of the compounds can bind specifically to CP.^15^^,^^16^^,^^23^^,^^24^^,^^25^^,^^26^ Salicylaldehyde Schiff base derivatives (*E*) -2,4-dichloro-6- (((3-methoxyphenyl) imino) methyl) phenol functions as synthetic elicitors to enhance the immune response of plants. To resist persecution by harmful organisms, salicylaldehyde Schiff base derivatives also have excellent antiviral activity.^27^^,^^28^

In drug design, a molecular hybridization strategy is an important approach based on combining two or more molecular fragments with different biological activities into a new molecular entity by chemical or biological means. This strategy aims to combine the individual fragments’ strengths to improve the biological activity, pharmacokinetic properties, and selectivity of the new molecule against a specific target.^29^^,^^30^ Here, we have developed sulfonamide-containing 2*H*-chromene derivatives with anti-PVY activity by a clever combination of a sulfonamide structure that increases the water solubility of the drug and provides an additional hydrogen-bonded receptor and a natural product structure 2,2-dimethyl-2H-chromene, which has antiviral activity. Notably, these target compounds carry a unique salicylaldehyde Schiff base unit, as confirmed by X-ray diffraction results, where the phenolic hydroxyl group forms intramolecular hydrogen bonds with nitrogen atoms in the neighboring Schiff base unit to constitute a six-membered ring, which enhances the structural stability of the target compounds. 3D-QSAR plays a crucial role in drug design.^31^^,^^32^^,^^33^ This approach employs theoretical modeling and statistical methodologies to investigate the quantitative correlation between the 3D molecular configurations of a compound series and their respective biological outcomes, and it can reveal conformational relationships and guide structural modifications. Once a reliable 3D-QSAR model has been established, it facilitates the prediction of the biological potency of compounds. A 3D-QSAR model was constructed based on the EC_50_ value of the anti-PVY inactivating activity of this series of compounds, which further guided the synthesis of **C50**. The bioactivity assessment revealed **C50** to exhibit potent anti-PVY efficacy, with an EC_50_ of 53.3 μg/mL for its inactivating activity, outperforming the standard agent NNM, which had an EC_50_ of 73.7 μg/mL. This led to our interest in the mechanism of antiviral action of **C50**.

Currently, there are two primary approaches for tackling plant viral infections. One prominent method is stimulating the host immune system against viral invasion through plant immune inducers.^24^^,^^25^^,^^26^^,^^32^^,^^34^ The other is the use of antiviral drugs, which target the virus’s genetic material or essential proteins to stop its pathogenic behavior, a simple mode of action, and a much faster onset of action.^14^^,^^15^^,^^16^^,^^17^^,^^35^ The CP of plant viruses holds a pivotal function throughout the viral life cycle, including assembly and movement of virus particles, transmission by vectors, and RNA translation and replication, and is a key biochemical target for developing new pesticides.^14^^,^^15^^,^^16^^,^^35^^,^^36^

Moreover, based on the excellent inactivating activity of **C50** against PVY, molecular docking and molecular dynamics simulations revealed that there is a strong interaction between **C50** and the active pocket in the conserved folding region of the PVY CP, where a segment of the amino acid sequences within the specified area are among all the flexible filamentous viruses highly conserved.^13^^,^^36^^,^^37^ Thus, scientists consider this a key target for developing antiviral agent drugs.^36^ Therefore, interfering with these sites using drugs may hinder the interaction between CPs and vRNA, thereby affecting downstream pathogenic behavior. In addition, since these sites are conserved regions, this allows for a broad range of drug action and less likely resistance. What is exciting is that **C50** forms a strong hydrogen bonding interaction with amino acid residue Ser^125^ in the conserved folding region of CP, which serves as one of the key sites for CP binding to vRNA.^13^ It is reasonable to believe that our drug can compete with viral single-stranded RNAs to bind to this site, which leads to dysfunction of viral particle assembly and thus inhibits viral infestation in plants.

In the early stages of drug discovery, targeted mutagenesis strategies and MST techniques can be used to validate the effectiveness of potential drug targets.^15^^,^^20^ The mechanism of action and target selectivity of drug molecules can be initially assessed by altering critical residues of the target protein and observing the attachment of the drug molecule to the mutated target protein. The targeted mutation of Ser^125^ on PVY CP to Ala^125^ combined with MST assay revealed that the attachment of **C50** to PVY CP was noticeably diminished, which further indicates that this site is the critical site for anti-PVY of **C50**.

Previous research has demonstrated that the CP of PVY engages with vRNA twice: firstly, at three critical conserved vRNA-binding amino acids, Ser^125^, Arg^157^, and Asp^201^, within a structured region; secondly, at a non-conserved site, Ser^240^, positioned in the C-terminal segment of CPn.^13^ From a functional standpoint, mutations in the conserved Arg and Asp residues of CPs disrupt the *in vitro* assembly of potyvirus Johnsongrass mosaic virus and hinder the assembly and intercellular trafficking of potexvirus Pepino mosaic virus within plants.^37^^,^^38^ For PVY, however, there is no information on whether mutations in conserved residues of the CP affect virus function. We successfully expressed and purified a mutant CP (PVY CP^S125A^) in *E. coli* that mutated Ser at position 125 in the conserved region of the PVY CP to Ala, and this result preliminarily demonstrated that the Ser^125^ mutation did not affect the *in vitro* assembly of the CP. We constructed the mutant PVY^S125A^-GFP using a targeted mutagenesis strategy to verify this target’s validity and other functions *in vivo*. Infestation of *N. benthamiana* with *Agrobacterium tumefaciens* containing the PVY^S125A^-GFP plasmid revealed a considerable decrease in the accumulation of PVY in the plant, the same as that found after drug treatment. The accumulation of plant viruses in the host is mainly influenced by the replicative assembly and movement of virus particles.^48^,^39^^,^^40^^,^^41^^,^^42^^,^^43^ Our experimental results by confocal microscopy and analysis indicated that the mutation in the PVY CP^S125^ locus would not impede the intercellular dissemination of PVY but would result in fewer virus particles. The likely reason for this is that the Ser^125^ mutation affects viral assembly, resulting in fewer viral particles being formed.

### Conclusion

**Figure 7:**
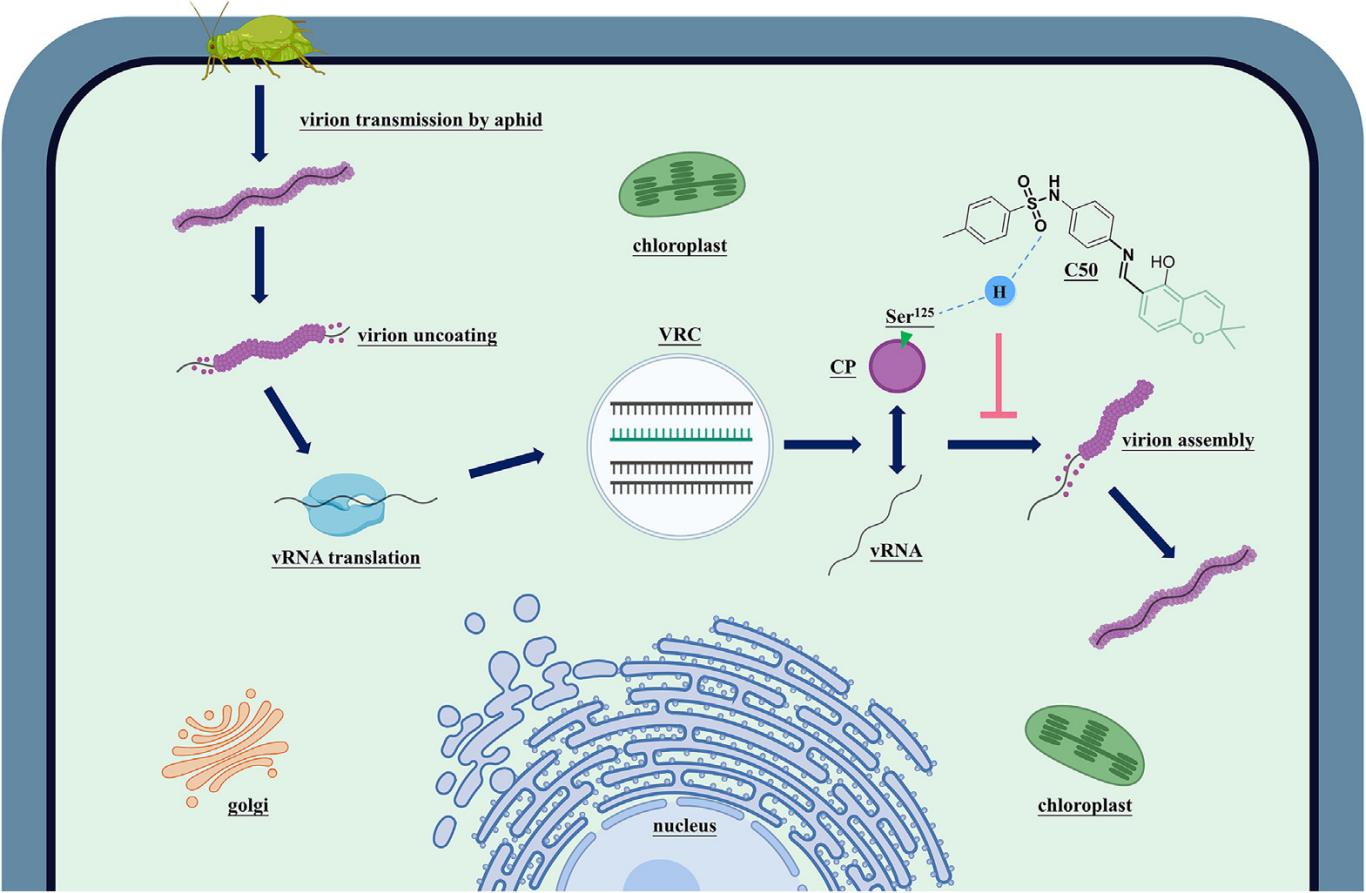
Mechanism of anti-PVY action of compound C50

CP is an essential component of utmost utility in viral life cycle, as it unlocks a variety of important downstream pathogenic processes. Efforts in developing effective CP inhibitors with unique action mechanisms continue to the present day; the contribution reported here provides a myriad of 2*H*-chromene-based antiphytovirals that have shown promising abilities against PVY infection. Compound **C50** stood out among the 50 products and control commercial drug. *In vitro*, the binding site on CP was discovered and verified as Ser^125^ using molecular docking paired with MST. Several tests conducted *in vivo* indicated that Ser^125^ is important during the mutual effect between PVY CP and vRNA, which is necessary for the assembly of PVY virions. Compound **C50** can dramatically decrease the viral pathogenicity through competitively inhibiting position Ser^125^ (Figure 7). In conclusion, this study provides a theoretical basis and potential action sites for the development of CP-based virus assembly inhibitory pesticides, offering new avenues for combating plant viral infections.

### Limitations of the study

Although compounds with good inhibitory activity against PVY were identified, subsequent structural optimization of this class of compounds is required to discover more active and less costly lead small molecules against plant viruses. The antiviral efficacy of **C50** was initially substantiated in this study solely through molecular docking and dynamic simulations; MST and preliminary investigations on the effect of mutated amino acid sites on the function of PVY virus infestation, movement, and viral particle formation were carried out. However, whether the drug can make host plants resistant to the disease is still unknown. In addition, changes in the binding affinity of CP to vRNA after Ser^125^ mutation or drug treatment have not been verified by bio-layer interferometry technology.

## Resource availability

### Lead contact

Further information and requests for resources and reagents should be directed to and will be fulfilled by the lead contact, Prof. Runjiang Song (songrj@gzu.edu.cn).

### Materials availability

Plasmids generated in this study are available from the lead contact, Prof. Runjiang Song (songrj@gzu.edu.cn).

### Data and code availability

• All requested data are shared promptly upon receipt of a request.
• The current study does not encompass the development or reporting of novel coding methodologies.
• Additional details necessary for re-examination of the data presented in this article can be sourced from the lead contact upon submission of a request.

## Acknowledgments

Thanks to the State Key Program of National Natural Science of China (no. 32330087), the General Program of the 10.13039/501100001809National Natural Science Foundation of China (no. 31871933), the National Science Foundation for Young Scientists of China (no. 32302388), and the Central Government Guides Local Science and Technology Development Fund Projects (Qiankehezhongyindi) (no. 2024007) for supporting the project.

Thanks to the Center of Pharmaceutical Technology of Tsinghua University for supporting Schrödinger software, molecular dynamics simulation, and molecular docking.

## Author contributions

Conceptualization, R.S. and X.Y.; methodology, D.L., C.W., and Y.T.; investigation, X.Y., D.L., J.L., and C.Z.; writing – original draft, X.Y.; writing – review and editing, R.S. and Y.T.; funding acquisition, B.S. and R.S.; resources, B.S., X.L., and Y.T.; supervision, R.S. and B.S.

## Declaration of interests

The authors declare no competing interests.

## STAR★Methods

### Key resources table

**Table:** 

REAGENT or RESOURCE	SOURCE	IDENTIFIER
**Antibodies**		

anti-GFP	Proteintech	Cat#66002-1-Ig; RRID: AB_11182611
anti- PVY CP	Youke	Custom synthesis, http://www.youke-ab.cn/
Horseradish peroxidase-conjugated goat anti-rabbit IgG	Proteintech	Cat#SA00001-2; RRID: AB_2722564

**Bacterial and virus strains**		

*Escherichia coli* DH5*α* Competent Cells	Sangon Biotech	Cat#B528413-0010
*Escherichia coli* BL21 (DE3) Competent Cells	Sangon Biotech	Cat#B528414-0010
Agrobacterium GV3101 Competent Cells	Sangon Biotech	Cat#B528430-0010
Potato virus Y	Our laboratory collection	N/A

**Biological samples**		

*Nicotiana benthamiana*	Our laboratory collection	N/A
*Nicotiana tabacum* K326	Our laboratory collection	N/A
*Chenopodium amaranticolor*	Our laboratory collection	N/A

**Chemicals, peptides, and recombinant proteins**		

Isopropyl *β*-D-1-thiogalactopyranoside	Sangon Biotech	Cat#A600168-0005
2,4-dihydroxybenzaldehyde	Energy Chemical	Cas: 95-01-2
3-methylbut-2-enal	Energy Chemical	Cas: 107-86-8
citral	Energy Chemical	Cas: 5392-40-5
*p*-phenylenediamine	Energy Chemical	Cas: 106-50-3
Substituted sulfonyl chloride	Energy Chemical	N/A
DMSO-*d*_6_	Energy Chemical	Cas: 2206-27-1
Ningnanmycin	Bide Pharmatech	Cas: 156410-09-2
Ponceau S	Sangon Biotech	Cat#A610437-0025
PVY CP	Wei et al.^15^	https://doi.org/10.1002/advs.202309343
PVY CP^S125A^	This paper	N/A

**Critical commercial assays**		

Trizol reagent	Invitrogen	Cat#15596026
HiScript III 1st Strand cDNA Synthesis Kit (+gDNA wiper)	Vazyme	Cat#R312-01
TB Green	TaKaRa	Cat#R820A
Monolith RED-NHS second generation protein labeling kit	NanoTemper	Cat#MO-L011

**Deposited data**		

Potato Virus Y coat protein structure	Kežar et al. (2019)^13^	PDB: 6HXX
(*E*)-4-chloro-*N*-(4-(((5-hydroxy-2,2-dimethyl-2*H*-chromen-6-yl)methylene)amino)phenyl) benzenesulfonamide crystallographic data	This paper	CCDC: 2297246

**Oligonucleotides**		

Primers used for DNA amplification, RT-qPCR and mutagenesis	This paper	See supplemental information (Table S2)

**Recombinant DNA**		

pCAMBIA0390 expression vector for PVY-GFP	Cheng et al.^44^	https://doi.org/10.1016/j.virusres.2019.197827
pCAMBIA0390 expression vector for PVY^S125A^-GFP	This paper	N/A
pET32a (+) expression vector for PVY CP	Sangon Biotech	Custom synthesis, https://www.sangon.com
pET32a (+) expression vector for PVY CP^S125A^	Sangon Biotech	Custom synthesis, https://www.sangon.com

**Software and algorithms**		

NanoTemper Monolith Instrument (NT.115) Control and Analysis Software Package	NanoTemper	https://nanotempertech.com/microscale-thermophoresis
SYBYL-2.1	Tripos	https://www.tripos.com
Schrödinger Maestro 13.5	Schrödinger	https://www.schrodinger.com
MestReNova	Mestrelab Research	https://www.mestrelabcn.com
SPSS 26.0	IBM	https://www.ibm.com
Microsoft Excel 2021	Microsoft	https://www.microsoft.com
ZEN 2.1	ZEISS	https://www.zeiss.com
ChemDraw 23	CambridgeSoft	https://www.chemdraw.com.cn
Chemi Doc MP Imaging System	Bio-Rad	https://www.bio-rad.com
Desmond	Schrödinger	https://www.schrodinger.com

**Other**		

Copper mesh carbon support film	Zhongjingkeyi	Cat#BZ11022a

### Method details

#### Materials and instruments

2,4-dihydroxybenzaldehyde, 3-methylbut-2-enal, citral, *p*-phenylenediamine, and substituted sulfonyl chloride were purchased from Energy Chemical (Anqing, Anhui, China). The solvents employed as reagents were of a grade suitable for direct use, without the necessity for any additional purification steps. A greenhouse was used to cultivate *Nicotiana tabacum* cv. K326, *Nicotiana benthamiana*, and *Chenopodium amaranticolor* plants maintained at a 6/18-h (dark/light) photoperiod and 25°C. PVY were propagated in *N. tabacum* cv. K326.

A micro melting point apparatus (XGE X4B) was utilized to ascertain the melting point of the compounds. The ^1^H, ^13^C, and ^19^F spectra were acquired on Bruker AG 400 and a 500 MHz NMR apparatus, respectively, employing TMS as the internal reference and DMSO-*d*_6_ as the solvent medium. High-resolution mass spectrometry (HRMS), facilitated by the Thermo Scientific Q Exactive instrument (Waltham, MA, U.S.A.), was employed to determine the molecular weights of the compounds. Furthermore, X-ray crystallographic data were collected using a Bruker Smart Apex CCD area detector diffractometer (Bruker, Germany), which utilized Mo *Kα* radiation for precise measurements.

#### Preparation of compounds C1−C50

The synthesis of intermediate compounds **A1**−**A41** was achieved by adapting the methodology documented in literature source.^42^ In a 100 mL round-bottom flask, *p*-phenylenediamine (3 mmol) was dissolved in 20 mL of dichloromethane while being maintained under an ice-bath condition. Triethylamine (4.5 mmol), serving as an acid scavenger, was subsequently added. A solution of substituted sulfonyl chloride (3.00 mmol) in 5 mL of dichloromethane was then slowly dripped into the mixture. Following the completion of the addition, the ice bath was removed, and the reaction was allowed to proceed with stirring for 3 h. The resulting mixture was concentrated and subsequently purified via silica gel column chromatography, employing a mixture of petroleum ether and ethyl acetate (3:1 ratio) as the eluent. This purification process yielded the intermediate compounds **A1**−**A41.**

Intermediate compounds **B1** and **B2** were prepared by adapting and partially refining a literature-based procedure.^45^ In this synthesis, 3-methyl-2-butenal or citral (103.6 mmol), CaCl_2_ (43.0 mmol), and triethylamine (172.1 mmol) were dissolved in 200 mL of absolute ethanol. Subsequently, 2,4-dihydroxybenzaldehyde (51.8 mmol) was introduced into the solution. Formaldehyde (51.8 mmol) was then added, and the reaction mixture was subjected to reflux for 3 h. Following this, the ethanol was removed via vacuum distillation. To the residue, 100 mL of distilled water was added, and the pH was adjusted to 5 using dilute HCl. The aqueous mixture was then extracted with ethyl acetate (three times with 100 mL each). The combined organic extracts were washed with saturated brine, dried over anhydrous MgSO_4_, and concentrated. Finally, the crude product was purified by silica gel column chromatography, employing a mixture of petroleum ether and ethyl acetate (30:1) as the eluent, yielding the desired intermediates **B1** and **B2.**

Target compounds **C1**−**C50** were synthesized through the same method and procedure, with **C1** as a representative example, a solution of *N*-(4-aminophenyl)-4-methoxybenzenesulfonamide (1 mmol) and intermediate **B1** (1.2 mmol) was prepared by dissolving them in 10 mL of ethanol. A catalytic quantity of glacial acetic acid was then added and then heated. The reaction progress was monitored during the experiment using thin-layer chromatography. Once a significant portion of the starting materials had undergone a reaction, the solvent in the mixture was concentrated. After the evaporation of ethanol and subsequent aqueous workup, the obtained residue underwent purification through column chromatography. This process utilized a solvent system consisting of ethyl acetate and petroleum ether in a ratio of 5:1 as the eluent. This purification process ultimately yielded the target compound **C1**. (*E*) -*N*- (4-(((5-hydroxy-2,2-dimethyl-2*H*-chromen-6-yl) methylene) amino) phenyl) -4-methoxybenzenesulfonamide **(C1):** Yield: 60.13%; yellow solid; m. p. 139−141°C; ^1^H NMR (500 MHz, DMSO-*d*_6_) *δ* 14.25 (s, 1H, -OH), 10.25 (s, 1H, -SO_2_NH-), 8.76 (d, *J* = 4.1 Hz, 1H, -N=CH-), 7.73–7.67 (m, 2H, Ar-H), 7.34–7.26 (m, 3H, Ar-H), 7.14 (dd, *J* = 9.2, 3.6 Hz, 2H, Ar-H), 7.09–7.03 (m, 2H, Ar-H), 6.63 (d, *J* = 10.5 Hz, 1H, Ar-H), 6.38 (d, *J* = 7.9 Hz, 1H, Ar-H), 5.72 (d, *J* = 10.8 Hz, 1H, Ar-H), 3.79 (d, *J* = 4.2 Hz, 3H, -OCH_3_), 1.39 (s, 6H, -CH_3_). ^13^C NMR (126 MHz, DMSO-*d*_6_) *δ* 163.0, 162.4, 158.6, 157.3, 143.5, 136.9, 134.1, 131.5, 129.4, 129.2, 122.3, 121.4, 116.0, 114.9, 113.3, 109.05, 108.4, 77.6, 56.1, 28.3. HRMS (ESI) m/z for C_25_H_24_N_2_O_5_S [M + H]^+^ calcd 465.14787, found 465.14771.

The spectral profiles of all target compounds are provided in the accompanying Supporting information (see Data S2).

#### Antiviral bioassay

According to previous reports,^46^ PVY was infested in *Nicotiana tabacum* K326, and PVY was harvested and purified after showing severe infection symptoms on the leaves. The literature-guided protocol was followed to assess the curative, prophylactic, and non-reactive potential of the target compounds under investigation.^15^

##### Curative activity of compounds against PVY

To assess the curative effectiveness against PVY, a uniform layer of 200–300 mesh emery was applied to the leaves of *C. amaranticolor*, followed by rubbing with a virus solution. For 1.5 h, then the emery was washed off with water. After the leaves naturally dried, a 500 *μ*g/mL solution of the test compound was carefully applied to one side of the leaf, while the opposite side received a 1% Tween 80 solution as a control. The plants were subsequently incubated in a greenhouse maintained at 25°C. After 5−7 days, the presence of necrotic spots on the leaves was recorded, with each compound tested on three replicate plants. The average inhibition rate was determined by analyzing the data from these replications.

##### Protective activity of compounds against PVY

A 500 *μ*g/mL solution of the targeted compound was delicately applied to one side of *C. amaranticolor* leaves, while the other side was treated with a 1% Tween 80 aqueous solution. The plants were positioned in a greenhouse set at 25°C. Following a 24 h period, the entire leaf surface was inoculated with PVY by uniformly distributing 200–300 mesh emery. After 1.5 h, the emery was washed off with water, and the plants were returned to the greenhouse. After 5–7 days, the number of necrotic spots was tallied, with the assessment based on the greenhouse population. Each compound was tested in triplicate, and the average inhibition rate was subsequently calculated.

##### Inactivating activity of compounds against PVY

Target compound solution (1,000 *μ*g/mL) and 2 × virus solution were combined in equal quantities and incubated for 30 min. Evenly distribute emery onto the leaf blades, and apply the mixture of virus and compound solution onto the right side of *C. amaranticolor* leaf blades. Following this, an identical quantity of a 1× virus solution was applied to the untreated side of the leaf. Following a duration of 1.5 h, the emery residue present on the leaf surface was removed using a rinsing process utilizing water. The plants were incubated within a controlled greenhouse environment, maintaining a consistent temperature of 25°C. After 5−7 days, they proceeded to statistics the number of necrotic spots on the leaves. For each chemical, triplicate experiments were undertaken, and the average percentage of inhibition was subsequently determined.

#### 3D-QSAR studies

The software SYBYL-2.1 was used to produce comparative molecular field analysis (CoMFA) and comparative molecular similarity index analysis (CoMSIA) models. A total of 39 compounds were randomly chosen for training purposes, with the remaining 10 exclusively assigned for testing. 3D-QSAR models for the molecular structures of the target compounds and their effects on inactivating PVY were developed using partial least squares (PLS) regression. Initially, the structures of the target compounds were subjected to energy minimization within the software. Subsequently, compound **C6** served as the reference molecule, and the 3D conformations of all compounds in the training set were aligned by selecting the common scaffold. Cross-validation of the training set compounds was performed using the leave-one-out strategy (LOO) diligently. This process involved computing the cross-validation coefficient (q^2^) and determining the optimal number of principal components (ONC). The model’s performance was assessed in terms of its goodness of fit, non-cross-validation coefficient (*r*^2^), Fisher statistic (F), standard error of estimate (SEE), and relative force field contribution. Subsequently, the model was further refined through the conformational search technique. Finally, the optimized model was employed to predict the biological activities of the target compounds in the test set.

#### Molecular docking and molecular dynamics simulation

The molecular docking was performed using the Schrödinger Maestro 13.5 program (March 2023 edition). The crystallographic structure corresponding to PVY CP (PDB: 6HXX) was initially obtained from the RCSB PDB database. The protein crystals that were obtained underwent a series of protein preprocessing procedures, which included restoring natural ligand states, refining hydrogen bond assignments, minimizing the energy of protein, and eliminating water molecules. The LigPrep module was utilized to process the two-dimensional structure files of compounds **C6** and **C50**, generating all conceivable three-dimensional chiral conformations. The ligand molecules **C6** and **C50** were submitted to molecular docking with the active site of PVY CP utilizing the maximum precision XP docking method. The lower score signifies a diminished binding free energy between the chemical compound and the protein, implying a heightened binding stability.

To enhance the binding efficacy of compound-protein complexes, we employed conventional molecular dynamics simulations through the Desmond software. The OPLS4 force field was utilized to accurately model the interactions between the protein and small molecules, while the SPCE model was adopted for the water solvent. The complex was immersed in a cubic water box and fully solvated. To ensure system neutrality, 0.150 M chloride and sodium ions were added. An initial energy minimization was conducted using the steepest descent method, involving 50,000 steps. Following this, the heavy atoms were restrained during NVT and NPT equilibration phases, spanning another 50,000 steps. Throughout, the system temperature was held at 300 K and the pressure at 1 bar. Upon completion of these equilibration stages, an unrestrained simulation was executed for a duration of 100 ns. The interactions were scrutinized, and dynamic trajectory data was compiled using Maestro 13.5, facilitating further analysis and optimization of the binding mode.

#### Plasmid construction and site-directed mutagenesis

The pCamPVY-GFP plasmid (GenBank: MN381731) was constructed in the previous study.^44^ Subsequently, the coding region of PVY CP was amplified via PCR from the pCamPVY-GFP and fused into a modified pCambia0390 vector, which harbors a 35S promoter and the GFP gene, yielding the pCamGFP-PVY CP construct. For expression in *Escherichia coli*, the PVY CP sequence was transferred into the pET-32a (+) vector, creating the pET-PVY CP plasmid. Furthermore, to introduce a Ser^125^ substitution in both pCamPVY-GFP and pET-PVY CP, site-directed mutagenesis was employed, adhering to a previously established protocol.^47^ This led to the generation of pCamPVY^S125A^-GFP and pET-PVY CP^S125A^ constructs, respectively. All the constructed plasmids were sequenced. Table S2 lists the primers used in this study.

#### Agrobacterium infection of PVY-GFP, agent treatment, and photographing

The *Nicotiana benthamiana* plants were subjected to infestation by Agrobacterium tumefaciens, which carried either pCamPVY-GFP or an empty vector (mock). Following this a solution with target compound **C50** (500 *μ*g/mL), NNM (500 *μ*g/mL), and a volume equivalent to DMSO (serving as the control) was applied via spraying onto all of the leaves. At 7 dpai, the leaves of *N. benthamiana* were looked at for the GFP using a LUYOR UV light (Shanghai, China), and captured in photographs. The foliage was gathered, rapidly frozen with liquid nitrogen, and subsequently preserved in a refrigerator set at a temperature of −80°C.

#### RNA extraction, cDNA synthesis, and RT-qPCR

Leaf blades of *Nicotiana benthamiana* were pulverized into fine powder with the aid of liquid nitrogen, followed by the extraction of total RNA from the leaf tissue utilizing Trizol reagent (Invitrogen, California, USA). Subsequently, cDNA synthesis was conducted through reverse transcription with primers and a reverse transcription kit (Vazyme, Nanjing, China). Any potential DNA contamination was effectively removed using a gDNA wipe enzyme (Vazyme, Nanjing, China) as per the manufacturer’s guidelines. RT-qPCR analysis was carried out employing TB Green (TaKaRa, Beijing, China). Detailed information regarding the primers utilized in this investigation can be found in Table S2.

#### Expression and purification of PVY CP and PVY CP mutants

The PVY CP^wt^ gene, the wild-type variant of the Potato Virus Y coat protein, was cloned into the pET-32a (+) prokaryotic expression vector. This recombinant vector was then thermally transformed into BL21 (DE3) bacterial host cells for subsequent protein expression. The strains above were cultivated on a liquid Luria-Bertani medium (180 rpm, 37°C). To initiate the expression of PVY CP, isopropyl β-D-1-thiogalactopyranoside (IPTG) was added to the culture medium at a concentration of 1.0 mmol when the optical density at 600 nm (OD_600_) reached a range between 0.6 and 0.8. This step triggered the production of the PVY CP protein. The induction process was maintained for 10 h (180 rpm, 16°C). The 6 × -His-S-tagged PVY CP^wt^ were purified using nickel-nitriloacetic acid by high-performance column affinity chromatography. Subsequently, enterokinase was employed to digest the proteins, and the desired target proteins were isolated using His-tag purification resin. Ultimately, the proteins were identified through the employment of a 12% sodium dodecyl sulfate-polyacrylamide gel electrophoresis method. The pET-32a (+) plasmid containing the PVY CP^wt^ gene was used as a template. The gene encoding serine at position 125 on PVY CP^wt^ was a point mutation to the alanine gene. The mutant protein was purified using the method for the purification of PVY CP^wt^, which was PVY CP^S125A^.

#### MST assay for the affinity of the compounds with PVY CP

The proteins' binding affinity with target chemicals was evaluated using a Monolith NT.115 instrument (Nano Temper, Munich, Germany), utilizing established methods described in literature.^35^^,^^48^ A concentration of 2.0 mM was selected for the chemicals, and the proteins were tagged with the Monolith TM RED-NHS second-generation labeling kit. The MST assay analyzed the fluorescence intensity of the labeled proteins, focusing on a range from 400 to 1200 to investigate their interactions. The scanning method was conducted using an “LED power” setting of 40% and a temperature that was maintained at 25.0°C.

#### Western blot assay

The leaves of the assayed *N. benthamiana* were used to extract total protein according to the previously described.^15^ An anti-GFP (Proteintech, Wuhan, China) or anti-PVY CP (Youke, Shanghai, China) antibody and horseradish peroxidase-conjugated goat anti-rabbit IgG (Proteintech, Wuhan, China) were individually used as the primary and secondary antibody. The visualization of the target protein’s signal was facilitated through the use of the Chemi Doc MP Imaging System manufactured by Bio-Rad.

#### Virus particle purification

Leaves of *N. benthamiana* that had been infected with wild-type or mutated PVY for 5 days were harvested for the extraction of viral particles. The methodology for purifying virion particles was performed according to our established protocol. The supernatant containing PVY was absorbed onto a copper grid coated with a carbon support film (Zhongjingkeyi, Beijing, China) and subsequently stained with a 1% phosphotungstic acid buffer. Subsequently, the structure of PVY particles was visualized using transmission electron microscopy (TEM, Talos F200C, FEI, USA).

#### Confocal microscopy

To analyze cell-to-cell movement of PVY-GFP and its mutant in *N. benthamiana* plants, the leaf patches infiltrated with Agrobacterium carrying pCamPVY-GFP and pCamPVY^S125A^-GFP were observed using a confocal microscope (Carl Zeiss, Germany). The excitation wavelength for observing GFP fluorescence was adjusted to 488 nm, while the emission wavelengths were specified to fall within the 520 to 540 nm range. The captured images were subsequently analyzed and processed with ZEN 2.1 software.

### Quantification and statistical analysis

All experiments were conducted with three independent biological replicates per treatment. The sample size (n) for each measurement was determined in triplicate. Statistical analysis was performed using Microsoft Excel 2021. In Table 1 all data are presented as means ± Standard deviation (SD). SPSS software (version 26.0) was used for one-way ANOVA. Error bars on Figures 3C, 3D, and 6C–6F indicate SD. Different letters indicate statistically significant differences (*p* < 0.05).
