# Supplementary material for: Rational design of 2H-chromene-based antiphytovirals that inhibit virion assembly by outcompeting virus capsid-RNA interactions
Source: iScience. 2024 Oct 18;27(11):111210. doi: 10.1016/j.isci.2024.111210 (PMC11565046; doi:10.1016/j.isci.2024.111210)
Supplement: Document S1. Figures S1–S3, Tables S1, S2, Data S1 and S2 [file mmc1.pdf]

iScience, Volume *27*

Supplemental information

Rational design of 2*H*-chromene-based antiphytovirals that inhibit virion assembly by outcompeting virus capsid-RNA interactions

Xiong Yang, Deguo Liu, Chunle Wei, Jianzhuan Li, Chunni Zhao, Yanping Tian, Xiangdong Li, Baoan Song, and Runjiang Song

Supplemental figures

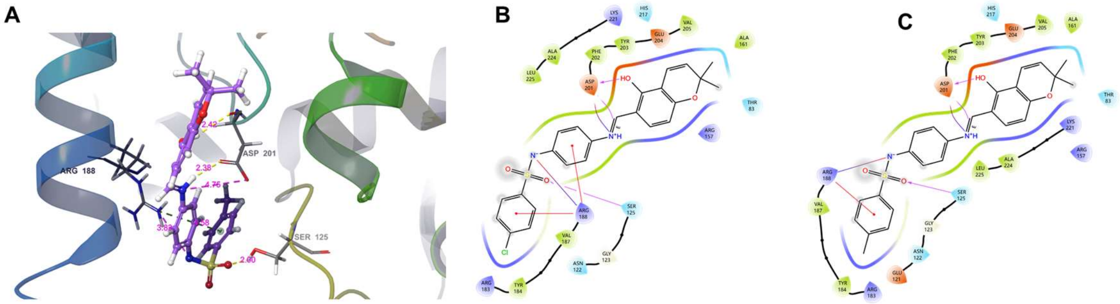

Figure S1. Docking results of C6 and C50 molecules with PVY CP, related to Figure 5A. (A) 3D map of **C6** molecule docking. **(B) C6** molecule docking plan view. **(D) C50** molecule docking plan view

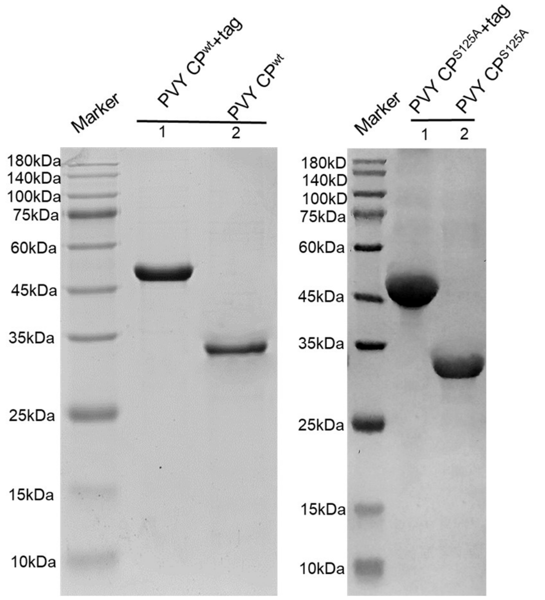

Figure S2. Identification of target proteins by 12% SDS-PAGE, related to Figure 5G. Lane 1 shows PVY CP^{wt} with His-S-tag and PVY CP^{S125A}, and lane 2 shows PVY CP^{wt} and PVY CP^{S125A} after digestion and desalting.

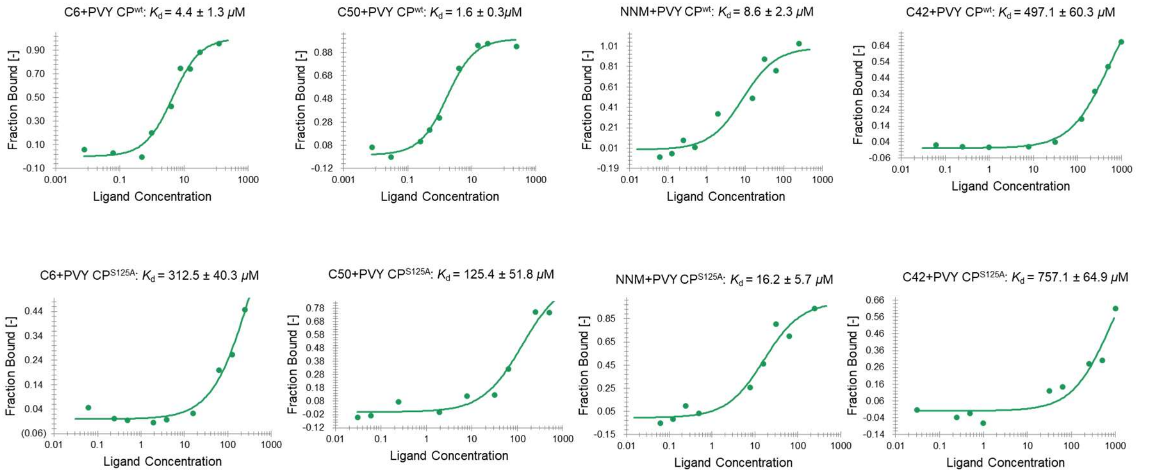

Figure S3. MST results of PVY CP^{wt} and mutant protein PVY CP^{S125A} with compounds C6, C42, C50, and Ningnanmycin (NNM), related to Figure 5G.

Supplemental tables

Table S1. Experimental and predicted results of pEC_{50} for the CoMFA and CoMSIA models, related to Figure 4A and 4B.

Compd.	Exp[a]	CoMFA		CoMSIA	
		pred[b]	res[c]	pred[b]	res[c]
**C1**	3.022	3.037	-0.015	3.033	-0.011
**C2**[d]	3.293	3.431	-0.138	3.095	0.198
**C3**	2.890	2.879	0.011	2.842	0.048
**C4**	2.819	2.857	-0.038	2.906	-0.087
**C5**[d]	3.437	3.689	-0.252	3.606	-0.169
**C6**	3.916	3.814	0.102	3.761	0.155
**C7**	3.654	3.598	0.056	3.407	0.247
**C8**	3.176	3.214	-0.038	3.181	-0.005
**C9**[d]	3.147	2.98	0.167	3.145	0.002
**C10**	3.022	2.965	0.057	2.962	0.060
**C11**	2.815	2.803	0.012	2.836	-0.021
**C12**[d]	3.078	3.08	-0.002	2.971	0.107
**C13**	2.875	2.916	-0.041	2.859	0.016
**C14**	2.825	2.836	-0.011	2.791	0.034
**C15**	2.716	2.653	0.063	2.69	0.026
**C16**	2.871	2.868	0.003	2.854	0.017
**C17**[d]	3.411	3.210	0.201	3.336	0.075
**C18**	3.359	3.378	-0.019	3.372	-0.013
**C19**	2.985	2.944	0.041	3.114	-0.129
**C20**	2.689	2.654	0.035	2.858	-0.169
**C21**	2.682	2.663	0.019	2.672	0.010
**C22**	2.932	2.954	-0.022	2.95	-0.018
**C23**	2.761	2.794	-0.033	2.719	0.042
**C24**	2.799	2.822	-0.023	2.737	0.062
**C25**[d]	3.283	3.511	-0.228	3.504	-0.221
**C26**[d]	3.671	3.412	0.259	3.415	0.256
**C27**[d]	3.695	3.719	-0.024	3.595	0.100
**C28**	2.950	2.984	-0.034	2.951	-0.001
**C29**	2.783	2.818	-0.035	2.833	-0.050
**C30**	2.793	2.803	-0.010	2.826	-0.033
**C31**	2.711	2.721	-0.010	2.661	0.050
**C32**	3.726	3.746	-0.020	3.664	0.062
**C33**	3.566	3.576	-0.010	3.674	-0.108
**C34**	3.541	3.628	-0.087	3.634	-0.093
**C35**	2.796	2.788	0.008	2.701	0.095

Compd.	Exp[a]	CoMFA		CoMSIA	
		pred[b]	res[c]	pred[b]	res[c]
**C36**	2.924	2.869	0.055	2.913	0.011
**C37**	3.200	3.177	0.023	3.131	0.069
**C38**	3.070	3.052	0.018	3.089	-0.019
**C39**[d]	3.057	3.153	-0.096	3.05	0.007
**C40**	3.396	3.379	0.017	3.483	-0.087
**C41**	2.837	2.859	-0.022	2.86	-0.023
**C42**	3.011	3.095	-0.084	2.973	0.038
**C43**	3.025	3.070	-0.045	3.029	-0.004
**C44**	2.767	2.763	0.004	2.76	0.007
**C45**	3.004	3.023	-0.019	3.086	-0.082
**C46**	3.038	3.017	0.021	2.972	0.066
**C47**	2.932	2.902	0.03	3.029	-0.097
**C48**	2.876	2.871	0.005	2.882	-0.006
**C49**	3.025	2.991	0.034	3.086	-0.061
**C50**[e]	3.924	3.757	0.167	3.846	0.078

[a] Experimental pEC_{50}. [b] Predicted pEC_{50}. [c] Residual error (experimental prediction). [d] Testing samples. [e] Compound synthesized basis on the CoMFA and CoMSIA mode.

Table S2. Primers used in this study, related to Figure 3C, 3D, 6A and 6C.

primer	Sequences 5'→3'
PVY CP^{S125A}-GFP-F	GAACCGCGCCAAATGTCAACGGAGTCTGGGTT
PVY CP^{S125A}-GFP-R	TTGGCGCGGTTCCATTTTCAATGCACCAAACC
qPCR-PVY CP-F	TGGCGAGGTTCCATTTTCA
qPCR-PVY CP-R	CATAGGAGAAACTGAGATGCCAACT
qPCR-EF1α-F	GACAAGCGTGTTATTGAGAGG
qPCR-EF1α-R	CACAGTGCAGTAGTACTTAGTG
qPCR-Actin-F	CACACTGGAGTGATGGTTGG
qPCR-Actin-R	GGTGTGGTGCCAAATCTTCT

Data S1. Full western blot images, related to Figure 6D.

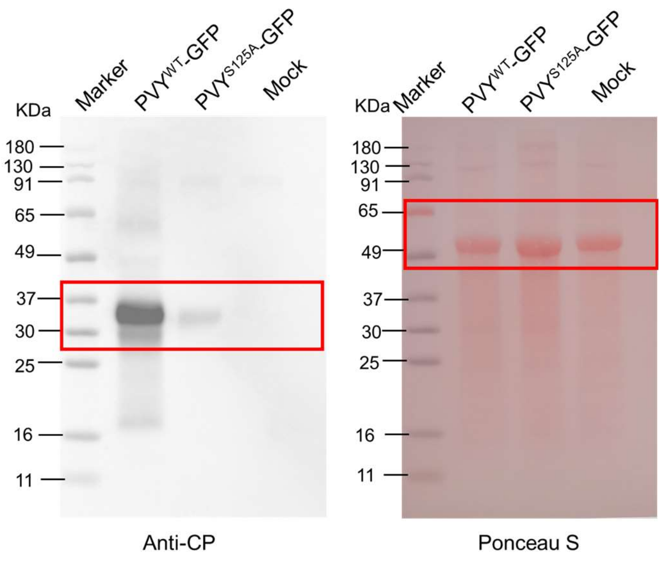

Data S2. Characterization of C1–C50, related to Figure 2A and STAR Methods.

A) Spectral and physicochemical data of the product.

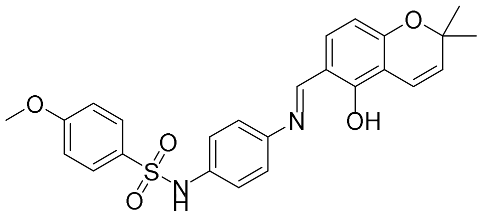

(*E*)-*N*-(4-(((5-hydroxy-2,2-dimethyl-2*H*-chromen-6-yl)methylene)amino)phenyl)-4-methoxybenzenesulfonamide (C1): Yield: 60.13%; yellow solid; m. p. 139−141 °C; ^{1}H NMR (500 MHz, DMSO-d_6) δ 14.25 (s, 1H, -OH), 10.25 (s, 1H, -SO_2NH-), 8.76 (d, J = 4.1 Hz, 1H, -N=CH-), 7.73 – 7.67 (m, 2H, Ar-H), 7.34 – 7.26 (m, 3H, Ar-H), 7.14 (dd, J = 9.2, 3.6 Hz, 2H, Ar-H), 7.09 – 7.03 (m, 2H, Ar-H), 6.63 (d, J = 10.5 Hz, 1H, Ar-H), 6.38 (d, J = 7.9 Hz, 1H, Ar-H), 5.72 (d, J = 10.8 Hz, 1H, Ar-H), 3.79 (d, J = 4.2 Hz, 3H, -OCH_3), 1.39 (s, 6H, -CH_3). ^{13}C NMR NMR (126 MHz, DMSO-d_6) δ 163.0, 162.4, 158.6, 157.3, 143.5, 136.9, 134.1, 131.5, 129.4, 129.2, 122.3, 121.4, 116.0, 114.9, 113.35, 109.0, 108.4, 77.6, 56.1, 28.3. HRMS (ESI) m/z for $C_{25}H_{24}N_2O_5S$ $[M+H]^+$ calcd 465.14787, found 465.14771.

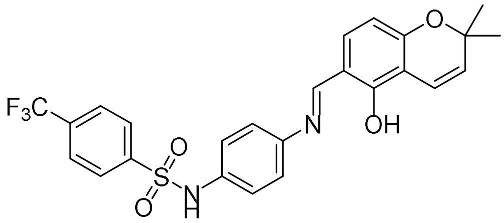

(*E*)-*N*-(4-(((5-hydroxy-2,2-dimethyl-2*H*-chromen-6-yl)methylene)amino)phenyl)-4-(trifluoromethyl)benzenesulfonamide (C2): Yield: 50.13%; yellow solid; m. p. 132−134 °C; ^{1}H NMR (500 MHz, DMSO-d_6) δ 14.21 (s, 1H, -OH), 8.76 (s, 1H, -N=CH-), 7.96 (s, 4H, Ar-H), 7.31 (t, J = 8.6 Hz, 3H, Ar-H), 7.15 (d, J = 8.9 Hz, 2H, Ar-H), 6.62 (d, J = 10.0 Hz, 1H, Ar-H), 6.38 (d, J = 8.5 Hz, 1H, Ar-H), 5.71 (d, J = 10.0 Hz, 1H, Ar-H), 1.39 (s, 6H, -CH_3). ^{13}C NMR (126 MHz, DMSO-d_6) δ 162.6, 158.6, 157.3, 144.1, 143.9, 134.1, 129.2, 128.1, 127.1 (d, J = 3.8 Hz), 122.5, 122.1, 115.9, 113.3, 109.0, 108.4, 77.6, 28.2. ^{19}F NMR (471 MHz, DMSO-d_6) δ -61.5. HRMS (ESI) m/z for $C_{25}H_{21}F_3N_2O_4S$ $[M+H]^+$ calcd 503.12469, found 503.12433.

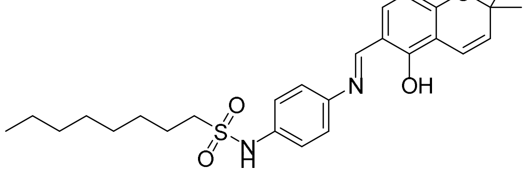

(*E*)-*N*-(4-(((5-hydroxy-2,2-dimethyl-2*H*-chromen-6-yl)methylene)amino)phenyl)octane-1-sulfonamide (C3): Yield: 43.34%; yellow solid; m. p. 94−96 °C; ^{1}H NMR (500 MHz, DMSO-d_6) δ 14.34

(s, 1H, -OH), 9.88 (s, 1H, -SO_2NH-), 8.82 (d, *J* = 4.0 Hz, 1H, -N=CH-), 7.42 – 7.33 (m, 3H, Ar-H), 7.26 (dd, *J* = 8.6, 3.6 Hz, 2H, Ar-H), 6.65 (d, *J* = 10.3 Hz, 1H, Ar-H), 6.39 (d, *J* = 8.9 Hz, 1H, Ar-H), 5.72 (d, *J* = 10.0 Hz, 1H, Ar-H), 3.08 (dd, *J* = 9.9, 5.5 Hz, 2H, -CH_2-), 1.70 – 1.62 (m, 2H, -CH_2-), 1.40 (s, 6H -CH_3), 1.31 (t, *J* = 7.5 Hz, 2H, -CH_2-), 1.19 (d, *J* = 12.4 Hz, 8H, -CH_2-), 0.82 (t, *J* = 7.2 Hz, 3H, -CH_3). ^{13}C NMR (101 MHz, DMSO-*d*$_6$) δ 162.2, 158.6, 157.2, 143.2, 137.4, 134.0, 129.1, 122.4, 120.7, 115.9, 113.3, 109.0, 108.3, 77.5, 50.9, 31.6, 28.8, 28.7, 28.2, 27.7, 23.4, 22.5, 14.3. HRMS (ESI) m/z for $C_{26}H_{34}N_2O_4S$ $[M+H]^+$ calcd 471.23120, found 471.23096.

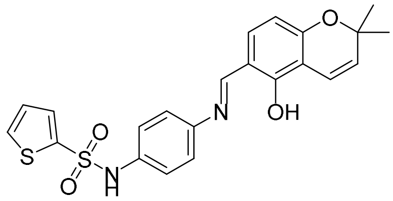

(*E*)-*N*-(4-(((5-hydroxy-2,2-dimethyl-2*H*-chromen-6-yl)methylene)amino)phenyl)thiophene-2-sulfonamide (C4): Yield: 59.31%; yellow solid; m. p. 148−150 °C; ^{1}H NMR (500 MHz, DMSO-*d*$_6$) δ 14.20 (s, 1H, -OH), 10.47 (s, 1H, -SO_2NH-), 8.74 (d, *J* = 3.0 Hz, 1H, -N=CH-), 7.86 (s, 1H, Ar-H), 7.51 (d, *J* = 4.2 Hz, 1H, Ar-H), 7.29 (dd, *J* = 8.4, 4.1 Hz, 3H, Ar-H), 7.15 (dd, *J* = 8.8, 3.4 Hz, 2H, Ar-H), 7.08 (d, *J* = 4.7 Hz, 1H, Ar-H), 6.59 (d, *J* = 11.3 Hz, 1H, Ar-H), 6.34 (d, *J* = 9.8 Hz, 1H, Ar-H), 5.68 (d, *J* = 9.9 Hz, 1H, Ar-H), 1.35 (s, 6H, -CH_3). ^{13}C NMR (126 MHz, DMSO-*d*$_6$) δ 162.7, 158.6, 157.4, 144.0, 140.3, 136.4, 134.2, 133.9, 133.0, 129.2, 128.2, 122.4, 121.9, 115.9, 113.3, 109.0, 108.5, 77.6, 28.3. HRMS (ESI): m/z for $C_{22}H_{20}N_2O_4S_2$ $[M+H]^+$ calcd 441.09373, found, 441.109320.

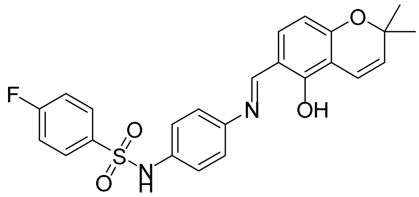

(*E*)-4-fluoro-*N*-(4-(((5-hydroxy-2,2-dimethyl-2*H*-chromen-6-yl)methylene)amino)phenyl) benzenesulfonamide (C5): Yield: 52.89%; yellow solid; m. p. 144−146 °C; ^{1}H NMR (500 MHz, DMSO-*d*$_6$) δ 14.22 (s, 1H, -OH), 10.41 (s, 1H, -SO_2NH-), 8.75 (s, 1H, -N=CH-), 7.86 – 7.79 (m, 2H, Ar-H), 7.41 (d, *J* = 7.7 Hz, 2H, Ar-H), 7.35 – 7.27 (m, 3H, Ar-H), 7.14 (t, *J* = 6.1 Hz, 2H, Ar-H), 6.62 (d, *J* = 10.6 Hz, 1H, Ar-H), 6.38 (d, *J* = 9.4 Hz, 1H, Ar-H), 5.72 (d, *J* = 10.7 Hz, 1H, Ar-H), 1.39 (s, 6H, -CH_3). ^{13}C NMR (126 MHz, DMSO-*d*$_6$) δ 162.6, 158.6, 157.3, 143.9, 136.4, 134.1, 130.2 (d, *J* = 9.6 Hz), 129.2, 122.4, 121.9, 117.1, 116.9, 115.9, 113.3, 109.0, 108.4, 77.6, 28.2. ^{19}F NMR (471 MHz, DMSO-*d*$_6$) δ -106.6. HRMS (ESI) m/z for $C_{24}H_{21}FN_2O_4S$ $[M+H]^+$ calcd 453.12788, found 453.12753.

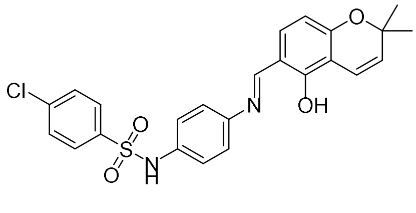

(*E*)-4-chloro-*N*-(4-(((5-hydroxy-2,2-dimethyl-2*H*-chromen-6-yl)methylene)amino)phenyl) benzenesulfonamide (C6): Yield: 69.32%; yellow solid; m. p. 139−141 °C; ^{1}H NMR (400 MHz, DMSO-*d*$_6$) δ 14.20 (s, 1H, -OH), 10.45 (s, 1H, -SO_2NH-), 8.76 (s, 1H, -N=CH-), 7.75 (d, *J* = 8.7 Hz, 2H, Ar-H),

7.64 (d, J = 8.6 Hz, 2H, Ar-H), 7.31 (t, J = 8.5 Hz, 3H, Ar-H), 7.14 (d, J = 8.8 Hz, 2H, Ar-H), 6.62 (d, J = 9.9 Hz, 1H, Ar-H), 6.38 (d, J = 8.4 Hz, 1H, Ar-H), 5.71 (d, J = 10.0 Hz, 1H, Ar-H), 1.39 (s, 6H, -CH_3). ^{13}C NMR (126 MHz, DMSO-d_6) δ 162.7, 158.6, 157.4, 144.0, 138.7, 138.4, 136.2, 134.1, 130.0, 129.2 (d, J = 12.0 Hz), 122.5, 122.0, 115.9, 113.3, 109.0, 108.5, 77.6, 28.3. HRMS (ESI) m/z for $C_{24}H_{21}ClN_2O_4S$ $[M+H]^+$ calcd 469.09833, found 469.09799.

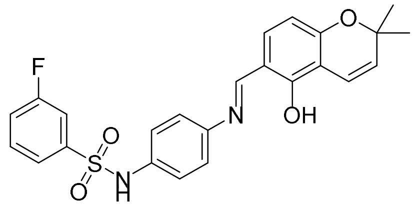

(*E*)-3-fluoro-*N*-(4-(((5-hydroxy-2,2-dimethyl-2*H*-chromen-6-yl)methylene)amino)phenyl) benzenesulfonamide (C7): Yield: 65.32%; yellow solid; m. p. 164−166 °C; ^{1}H NMR (500 MHz, DMSO-d_6) δ 14.20 (s, 1H, -OH), 10.49 (s, 1H, -SO_2NH-), 8.76 (s, 1H, -N=CH-), 7.64 – 7.59 (m, 2H, Ar-H), 7.56 (dd, J = 8.7, 2.6 Hz, 1H, Ar-H), 7.51 (dt, J = 9.2, 1.9 Hz, 1H, Ar-H), 7.34 – 7.28 (m, 3H, Ar-H), 7.16 (d, J = 8.9 Hz, 2H, Ar-H), 6.63 (d, J = 10.0 Hz, 1H, Ar-H), 6.38 (d, J = 8.5 Hz, 1H, Ar-H), 5.72 (d, J = 10.0 Hz, 1H, Ar-H), 1.39 (s, 6H, -CH_3). ^{13}C NMR (126 MHz, DMSO-d_6) δ 162.8, 158.6, 157.4, 144.1, 141.9 (d, J = 6.6 Hz), 136.1, 134.2, 132.3 (d, J = 8.2 Hz), 129.2, 123.5 (d, J = 3.2 Hz), 122.5, 122.0, 120.8, 120.7, 115.9, 114.3, 114.1, 113.3, 109.0, 108.5, 77.6, 28.3. ^{19}F NMR (471 MHz, DMSO-d_6) δ -110.1 (d, J = 5.9 Hz). HRMS (ESI) m/z for $C_{24}H_{21}FN_2O_4S$ $[M+H]^+$ calcd 453.12788, found 453.12747.

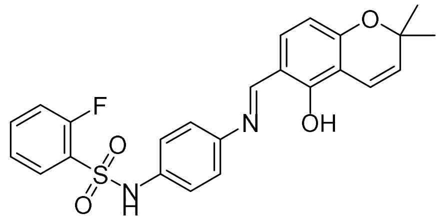

(*E*)-2-fluoro-*N*-(4-(((5-hydroxy-2,2-dimethyl-2*H*-chromen-6-yl)methylene)amino)phenyl) benzenesulfonamide (C8): Yield:59.29%; yellow solid; m. p. 175−177 °C; ^{1}H NMR (400 MHz, DMSO-d_6) δ 14.20 (s, 1H, -OH), 10.72 (s, 1H, -SO_2NH-), 8.74 (s, 1H, -N=CH-), 7.85 (d, J = 5.8 Hz, 1H, Ar-H), 7.69 (d, J = 8.0 Hz, 1H, Ar-H), 7.45 – 7.35 (m, 2H, Ar-H), 7.32 – 7.24 (m, 3H, Ar-H), 7.20 – 7.14 (m, 2H, Ar-H), 6.62 (d, J = 10.7 Hz, 1H, Ar-H), 6.37 (d, J = 7.7 Hz, 1H, Ar-H), 5.71 (d, J = 9.2 Hz, 1H, Ar-H), 1.38 (s, 6H, -CH_3). ^{13}C NMR (126 MHz, DMSO-d_6) δ 162.6, 158.6, 157.3, 143.8, 136.5 (d, J = 8.4 Hz), 136.1, 134.1, 131.0, 129.2, 127.4 (d, J = 13.5 Hz), 125.5 (d, J = 3.7 Hz), 122.4, 121.2, 117.9, 117.7, 115.9, 113.3, 109.0, 108.4, 77.6, 28.2. ^{19}F NMR (471 MHz, DMSO-d_6) δ -109.9. HRMS (ESI) m/z for $C_{24}H_{21}FN_2O_4S$ $[M+H]^+$ calcd 453.12788, found 453.12772.

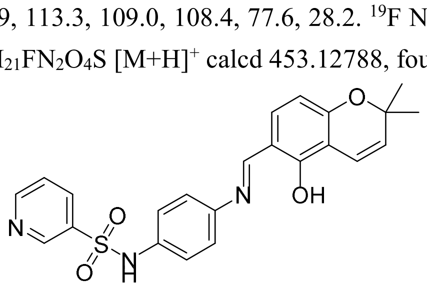

(*E*)-*N*-(4-(((5-hydroxy-2,2-dimethyl-2*H*-chromen-6-yl) methylene) amino) phenyl) pyridine-3-sulfonamide (C9): Yield: 40.91%; yellow solid; m. p. 150−152 °C; ^{1}H NMR (500 MHz, DMSO-d_6) δ 14.21 (s, 1H, -OH), 8.89 (d, J = 2.4 Hz, 1H, -N=CH-), 8.80 – 8.74 (m, 2H, Ar-H), 8.12 (dt, J = 8.1, 2.1 Hz, 1H, Ar-H), 7.60 (dd, J = 8.0, 4.8 Hz, 1H, Ar-H), 7.34 – 7.29 (m, 3H, Ar-H), 7.15 (d, J = 8.7 Hz, 2H,

Ar-H), 6.62 (d, J = 10.0 Hz, 1H, Ar-H), 6.38 (d, J = 8.5 Hz, 1H, Ar-H), 5.71 (d, J = 10.0 Hz, 1H, Ar-H), 1.39 (s, 6H, $-CH_3$). ^{13}C NMR (126 MHz, DMSO-d_6) δ 162.9, 158.6, 157.4, 154.1, 147.6, 144.3, 136.2, 135.9, 135.2, 134.2, 129.2, 124.9, 122.6, 122.2, 115.9, 113.3, 109.0, 108.5, 77.6, 28.3. HRMS (ESI) m/z for $C_{23}H_{21}N_3O_4S$ $[M+H]^+$ calcd 436.13255, found 436.13202.

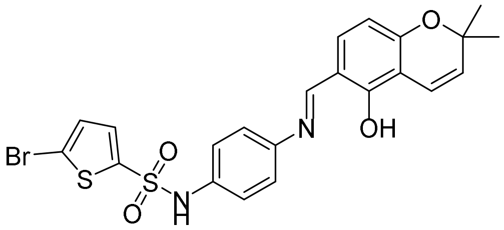

(*E*)-5-bromo-*N*-(4-(((5-hydroxy-2,2-dimethyl-2*H*-chromen-6-yl)methylene)amino)phenyl) thiophene-2-sulfonamide (C10): Yield: 45.67%; yellow solid; m. p. 140−142 °C; ^1H NMR (500 MHz, DMSO-d_6) δ 14.24 (s, 1H, -OH), 10.68 (s, 1H, $-SO_2NH-$), 8.80 (s, 1H, -N=CH-), 7.40 (d, J = 4.0 Hz, 1H, Ar-H), 7.35 (t, J = 8.3 Hz, 3H, Ar-H), 7.30 (d, J = 3.9 Hz, 1H, Ar-H), 7.20 (d, J = 8.8 Hz, 2H, Ar-H), 6.63 (d, J = 10.0 Hz, 1H, Ar-H), 6.39 (d, J = 8.5 Hz, 1H, Ar-H), 5.73 (d, J = 10.0 Hz, 1H, Ar-H), 1.39 (s, 6H, $-CH_3$). ^{13}C NMR (126 MHz, DMSO-d_6) δ 162.9, 158.6, 157.4, 144.4, 141.1, 135.9, 134.2, 133.7, 131.9, 129.2, 122.6, 122.2, 119.7, 115.9, 113.3, 109.0, 108.5, 77.6, 28.3. HRMS (ESI) m/z for $C_{22}H_{19}BrN_2O_4S_2$ $[M+H]^+$ calcd 519.00424, found 519.00458.

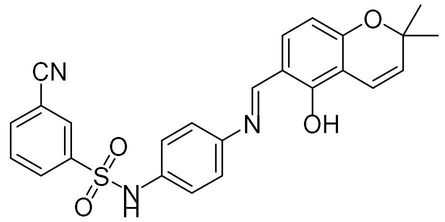

(*E*)-3-cyano-*N*-(4-(((5-hydroxy-2,2-dimethyl-2*H*-chromen-6-yl)methylene)amino)phenyl) benzenesulfonamide (C11): Yield: 69.74%; yellow solid; m. p. 152−154 °C; ^1H NMR (500 MHz, DMSO-d_6) δ 14.21 (s, 1H, -OH), 8.76 (s, 1H, -N=CH-), 8.19 (s, 1H, Ar-H), 8.11 (d, J = 7.8 Hz, 1H, Ar-H), 8.05 – 8.02 (m, 1H, Ar-H), 7.78 (t, J = 7.9 Hz, 1H, Ar-H), 7.31 (d, J = 9.2 Hz, 3H, Ar-H), 7.15 (d, J = 8.9 Hz, 2H, Ar-H), 6.62 (d, J = 10.0 Hz, 1H, Ar-H), 6.38 (d, J = 8.6 Hz, 1H, Ar-H), 5.71 (d, J = 10.0 Hz, 1H, Ar-H), 1.39 (s, 6H, $-CH_3$). ^{13}C NMR (126 MHz, DMSO-d_6) δ 162.8, 158.6, 157.4, 144.3, 141.1, 137.1, 135.8, 134.2, 131.6, 131.4, 130.7, 129.2, 122.5, 122.2, 117.9, 115.9, 113.3, 113.0, 109.0, 108.5, 77.6, 28.3. HRMS (ESI) m/z for $C_{25}H_{21}N_3O_4S$ $[M+H]^+$ calcd 460.13255, found 460.13220.

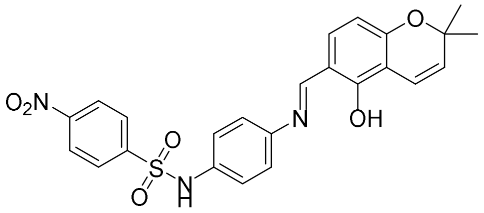

(*E*)-*N*-(4-(((5-hydroxy-2,2-dimethyl-2*H*-chromen-6-yl)methylene)amino)phenyl)-4-nitrobenzenesulfonamide (C12): Yield: 74.44%; yellow solid; m. p. 164−166 °C; ^1H NMR (500 MHz, DMSO-d_6) δ 14.19 (s, 1H, -OH), 10.69 (s, 1H, $-SO_2NH-$), 8.75 (s, 1H, -N=CH-), 8.38 (d, J = 8.8 Hz, 2H, Ar-H), 8.00 (d, J = 8.9 Hz, 2H, Ar-H), 7.32 (dd, J = 8.8, 7.2 Hz, 3H, Ar-H), 7.15 (d, J = 8.9 Hz, 2H, Ar-H), 6.62 (d, J = 10.0 Hz, 1H, Ar-H), 6.38 (d, J = 8.6 Hz, 1H, Ar-H), 5.71 (d, J = 10.0 Hz, 1H, Ar-H), 1.39 (s, 6H, $-CH_3$). ^{13}C NMR (126 MHz, DMSO-d_6) δ 162.9, 158.6, 157.4, 150.4, 145.3, 144.4, 135.7, 134.2,

129.2, 128.8, 125.2, 122.6, 122.3, 115.9, 113.3, 109.0, 108.5, 77.6, 28.3. HRMS (ESI) m/z for $C_{24}H_{21}N_3O_6S$ $[M+H]^+$ calcd 480.122238, found 480.12222.

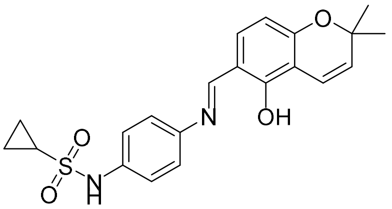

(*E*)-*N*-(4-(((5-hydroxy-2,2-dimethyl-2*H*-chromen-6-yl)methylene)amino)phenyl) cyclopropanesulfonamide (C13): Yield:57.99%; yellow solid; m. p. 147−149 °C; ^{1}H NMR (500 MHz, DMSO-*d*$_6$) δ 14.35 (s, 1H, -OH), 9.77 (s, 1H, -SO_2NH-), 8.83 (s, 1H, -N=CH-), 7.39 (d, *J* = 8.8 Hz, 2H, Ar-H), 7.36 (d, *J* = 8.5 Hz, 1H, Ar-H), 7.30 (d, *J* = 8.2 Hz, 2H, Ar-H), 6.65 (d, *J* = 10.0 Hz, 1H, Ar-H), 6.39 (d, *J* = 8.5 Hz, 1H, Ar-H), 5.73 (d, *J* = 10.0 Hz, 1H, Ar-H), 2.63 (t, *J* = 6.3 Hz, 1H, -CH-), 1.40 (s, 6H, -CH_3), 0.94 (d, *J* = 6.4 Hz, 4H, -CH_2-). ^{13}C NMR (126 MHz, DMSO-d$_6$) δ 162.3, 158.7, 157.3, 143.5, 137.4, 134.1, 129.2, 122.4, 121.8, 116.0, 113.3, 109.0, 108.4, 77.6, 30.0, 28.3, 5.5. HRMS (ESI) m/z for $C_{21}H_{22}N_2O_4S$ $[M+H]^+$ calcd 399.13730, found 399.13657.

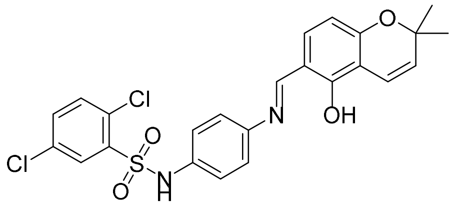

(*E*)-2,5-dichloro-*N*-(4-(((5-hydroxy-2,2-dimethyl-2*H*-chromen-6-yl)methylene)amino)phenyl) benzenesulfonamide (C14): Yield: 62.06%; yellow solid; m. p. 148−150 °C; ^{1}H NMR (500 MHz, DMSO-*d*$_6$) δ 14.19 (s, 1H, -OH), 8.75 (s, 1H, -N=CH-), 8.00 (d, *J* = 2.5 Hz, 1H, Ar-H), 7.72 (d, *J* = 2.6 Hz, 1H, Ar-H), 7.69 (d, *J* = 8.6 Hz, 1H, Ar-H), 7.31 (dd, *J* = 8.6, 2.3 Hz, 3H, Ar-H), 7.17 (d, *J* = 8.7 Hz, 2H, Ar-H), 6.62 (d, *J* = 10.1 Hz, 1H, Ar-H), 6.37 (d, *J* = 8.6 Hz, 1H, Ar-H), 5.71 (d, *J* = 10.0 Hz, 1H, Ar-H), 1.39 (s, 6H, -CH_3). ^{13}C NMR (126 MHz, DMSO-*d*$_6$) δ 162.8, 158.6, 157.4, 144.0, 138.5, 135.5, 135.0, 134.2 (d, *J* = 7.9 Hz), 132.7, 131.2, 130.1, 129.2, 122.6, 121.2, 115.9, 113.3, 109.0, 108.5, 77.6, 28.3. HRMS (ESI) m/z for $C_{24}H_{20}Cl_2N_2O_4S$ $[M+H]^+$ calcd 503.05936, found 503.05936.

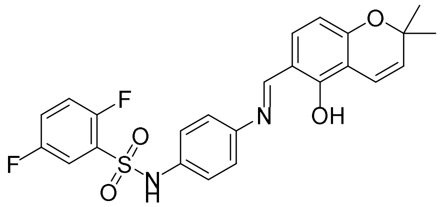

(*E*)-2,5-difluoro-*N*-(4-(((5-hydroxy-2,2-dimethyl-2*H*-chromen-6-yl)methylene)amino)phenyl) benzenesulfonamide (C15): Yield: 67.98%; yellow solid; m. p. 180−182 °C; ^{1}H NMR (500 MHz, DMSO-*d*$_6$) δ 14.21 (s, 1H, -OH), 10.89 (s, 1H, -SO_2NH-), 8.77 (s, 1H, -N=CH-), 7.66 (ddd, *J* = 7.9, 5.4, 3.2 Hz, 1H, Ar-H), 7.63 – 7.57 (m, 1H, Ar-H), 7.53 (td, *J* = 9.3, 4.1 Hz, 1H, Ar-H), 7.32 (dd, *J* = 8.7, 3.3 Hz, 3H, Ar-H), 7.18 (d, *J* = 8.9 Hz, 2H, Ar-H), 6.62 (d, *J* = 9.9 Hz, 1H, Ar-H), 6.38 (d, *J* = 8.5 Hz, 1H, Ar-H), 5.72 (d, *J* = 10.0 Hz, 1H, Ar-H), 1.39 (s, 6H, -CH_3). ^{13}C NMR (126 MHz, DMSO-*d*$_6$) δ 162.8, 158.6, 157.4, 144.1, 135.6, 134.2, 129.2, 123.3, 122.5, 121.5, 120.0, 117.4 (d, *J* = 28.4 Hz), 115.9, 113.3, 109.0, 108.5, 77.6, 28.3. ^{19}F NMR (471 MHz, DMSO-*d*$_6$) δ -115.2 (t, *J* = 9.6 Hz), -115.6 – -115.8 (m). HRMS (ESI) m/z for $C_{24}H_{20}F_2N_2O_4S$ $[M+H]^+$ calcd 471.11846, found 471.11819.

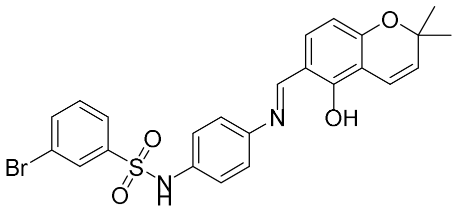

(E)-3-bromo-N-(4-(((5-hydroxy-2,2-dimethyl-2H-chromen-6-yl)methylene)amino)phenyl) benzenesulfonamide (C16): Yield: 68.38%; yellow solid; m. p. 161−163 ℃; ^{1}H NMR (500 MHz, DMSO-d_6) δ 14.20 (s, 1H, -OH), 10.48 (s, 1H, -SO_2NH-), 8.77 (d, J = 4.0 Hz, 1H, -N=CH-), 7.90 (s, 1H, Ar-H), 7.84 (d, J = 7.8 Hz, 1H, Ar-H), 7.77 – 7.72 (m, 1H, Ar-H), 7.52 (t, J = 7.8 Hz, 1H, Ar-H), 7.32 (d, J = 7.7 Hz, 3H, Ar-H), 7.18 – 7.13 (m, 2H, Ar-H), 6.62 (d, J = 10.0 Hz, 1H, Ar-H), 6.38 (d, J = 9.9 Hz, 1H, Ar-H), 5.72 (d, J = 10.4 Hz, 1H, Ar-H), 1.39 (s, 6H, -CH_3). ^{13}C NMR (126 MHz, DMSO-d_6) δ 162.8, 158.6, 157.4, 144.2, 141.8, 136.4, 136.1, 134.2, 132.1, 129.5, 129.2, 126.2, 122.6 (d, J = 16.3 Hz), 122.1, 115.9, 113.3, 109.0, 108.5, 77.6, 28.3. HRMS (ESI) m/z for $C_{24}H_{21}BrN_2O_4S$ $[M+H]^+$ calcd 513.04782, found 513.04767.

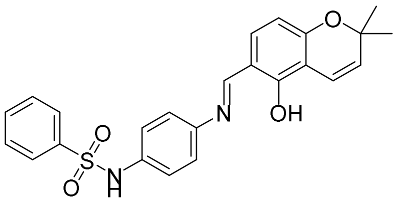

(E)-N-(4-(((5-hydroxy-2,2-dimethyl-2H-chromen-6-yl)methylene)amino)phenyl) benzenesulfonamide (C17): Yield:67.52%; yellow solid; m. p. 134−136℃; ^{1}H NMR (500 MHz, DMSO-d_6) δ 14.23 (s, 1H, -OH), 10.39 (s, 1H, -SO_2NH-), 8.75 (s, 1H, -N=CH-), 7.77 (d, J = 6.5 Hz, 2H, Ar-H), 7.58 (dd, J = 24.1, 9.0 Hz, 3H, Ar-H), 7.34 – 7.26 (m, 3H, Ar-H), 7.15 (t, J = 6.0 Hz, 2H, Ar-H), 6.62 (d, J = 10.1 Hz, 1H, Ar-H), 6.37 (d, J = 7.8 Hz, 1H, Ar-H), 5.71 (d, J = 10.1 Hz, 1H, Ar-H), 1.39 (d, J = 4.1 Hz, 6H, -CH_3). ^{13}C NMR (126 MHz, DMSO-d_6) δ 162.6, 158.6, 157.3, 143.7, 139.9, 136.6, 134.1, 133.5, 129.8, 129.2, 127.2, 122.4, 121.6, 116.0, 113.3, 109.0, 108.4, 77.6, 28.3. HRMS (ESI) m/z for $C_{24}H_{22}N_2O_4S$ $[M+H]^+$ calcd 435.13730, found 435.13712.

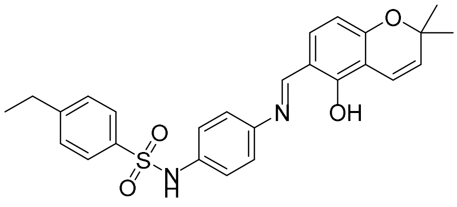

(E)-4-ethyl-N-(4-(((5-hydroxy-2,2-dimethyl-2H-chromen-6-yl)methylene)amino)phenyl) benzenesulfonamide (C18) : Yield: 59.37%; yellow solid; m. p. 121−123 ℃; ^{1}H NMR (500 MHz, DMSO-d_6) δ 14.26 (s, 1H, -OH), 10.35 (s, 1H, -SO_2NH-), 8.75 (s, 1H, -N=CH-), 7.69 (d, J = 8.3 Hz, 2H, Ar-H), 7.38 (d, J = 8.3 Hz, 2H, Ar-H), 7.31 (d, J = 8.6 Hz, 1H, Ar-H), 7.28 (d, J = 8.7 Hz, 2H, Ar-H), 7.15 (d, J = 8.9 Hz, 2H, Ar-H), 6.62 (d, J = 10.0 Hz, 1H, Ar-H), 6.37 (d, J = 8.3 Hz, 1H, Ar-H), 5.71 (d, J = 10.0 Hz, 1H, Ar-H), 2.63 (q, J = 7.6 Hz, 2H, -CH_2-), 1.39 (s, 6H, -CH_3), 1.14 (t, J = 7.6 Hz, 3H, -CH_3). ^{13}C NMR (126 MHz, DMSO-d_6) δ 162.4, 158.6, 157.3, 149.7, 143.5, 137.4, 136.8, 134.1, 129.1 (d, J = 11.9 Hz), 127.3, 122.4, 121.3, 116.0, 113.3, 109.0, 108.4, 77.6, 28.4, 28.3, 15.4. HRMS (ESI) m/z for $C_{26}H_{26}N_2O_4S$ $[M+H]^+$ calcd 463.16860, found 463.16830.

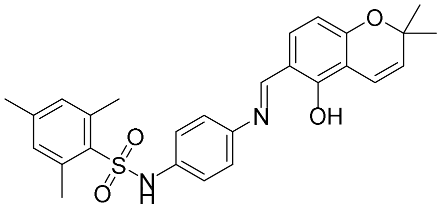

(*E*)-*N*-(4-(((5-hydroxy-2,2-dimethyl-2*H*-chromen-6-yl)methylene)amino)phenyl)-2,4,6-trimethylbenzenesulfonamide (C19) : Yield:58.74%; yellow solid; m. p. 137−139 °C; ^{1}H NMR (500 MHz, DMSO-d_6) δ 14.25 (s, 1H, -OH), 10.29 (s, 1H, -SO_2NH-), 8.74 (s, 1H, -N=CH-), 7.30 (d, J = 8.5 Hz, 1H, Ar-H), 7.27 (d, J = 8.6 Hz, 2H, Ar-H), 7.03 (d, J = 8.4 Hz, 2H, Ar-H), 7.00 (s, 2H, Ar-H), 6.62 (d, J = 10.1 Hz, 1H, Ar-H), 6.37 (d, J = 8.5 Hz, 1H, Ar-H), 5.71 (d, J = 10.0 Hz, 1H, Ar-H), 2.57 (s, 6H, 2-CH_3), 2.21 (s, 3H, -CH_3), 1.39 (s, 6H, -CH_3). ^{13}C NMR NMR (126 MHz, DMSO-d_6) δ 162.3, 158.6, 157.3, 143.2, 142.6, 139.2, 136.7, 134.3, 134.0, 132.3, 129.2, 122.4, 120.5, 116.0, 113.3, 109.0, 108.4, 77.5, 28.2, 23.0, 20.9. HRMS (ESI) m/z for $C_{27}H_{28}N_2O_4S$ $[M+H]^+$ calcd 477.18425, found 477.18396.

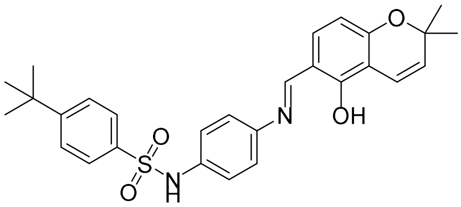

(*E*)-4-(tert-butyl)-*N*-(4-(((5-hydroxy-2,2-dimethyl-2*H*-chromen-6-yl)methylene)amino)phenyl) benzenesulfonamide (C20): Yield: 70.16%; yellow solid; m. p. 142−144 °C; ^{1}H NMR (500 MHz, DMSO-d_6) δ 14.26 (s, 1H, -OH), 10.42 (s, 1H, -SO_2NH-), 8.75 (s, 1H, -N=CH-), 7.72 (d, J = 8.6 Hz, 2H, Ar-H), 7.57 (d, J = 8.6 Hz, 2H, Ar-H), 7.30 (t, J = 8.5 Hz, 3H, Ar-H), 7.17 (d, J = 8.9 Hz, 2H, Ar-H), 6.62 (d, J = 10.0 Hz, 1H, Ar-H), 6.37 (d, J = 8.5 Hz, 1H, Ar-H), 5.71 (d, J = 10.0 Hz, 1H, Ar-H), 1.39 (s, 6H, -CH_3), 1.25 (s, 9H, -$C(CH_3)_3$. ^{13}C NMR (126 MHz, DMSO-d_6) δ 162.4, 158.6, 157.3, 156.4, 143.4, 137.3, 136.8, 134.1, 129.2, 127.0, 126.7, 122.4, 120.9, 116.0, 113.3, 108.4, 77.6, 35.4, 31.2, 28.2. HRMS (ESI) m/z for $C_{28}H_{30}N_2O_4S$ $[M+H]^+$ calcd 491.19990, found 491.19930.

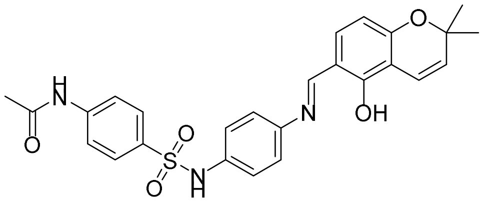

(*E*)-*N*-(4-(*N*-(4-(((5-hydroxy-2,2-dimethyl-2*H*-chromen-6-yl)methylene)amino)phenyl)sulfamoyl) phenyl)acetamide (C21): Yield:52.47%; yellow solid; m. p. 161−163 °C; ^{1}H NMR (500 MHz, DMSO-d_6) δ 14.24 (s, 1H, -OH), 10.32 (s, 1H, -SO_2NH-), 8.75 (s, 1H, -N=CH-), 7.65 (d, J = 8.3 Hz, 2H, Ar-H), 7.33 (dd, J = 13.8, 8.4 Hz, 3H, Ar-H), 7.28 (d, J = 8.9 Hz, 2H, Ar-H), 7.14 (d, J = 8.9 Hz, 2H, Ar-H), 6.62 (d, J = 10.0 Hz, 1H, Ar-H), 6.37 (d, J = 8.3 Hz, 1H, Ar-H), 5.71 (d, J = 10.0 Hz, 1H, Ar-H), 2.32 (s, 3H, -CH_3), 1.39 (s, 6H, -CH_3). ^{13}C NMR (126 MHz, DMSO-d_6) δ 169.5, 162.5, 158.6, 157.3, 143.6 (d, J = 13.4 Hz), 136.8, 134.1, 133.4, 129.2, 128.5, 122.3, 121.5, 119.1, 116.0, 113.3, 109.0, 108.4, 77.6, 28.3, 24.6. HRMS (ESI) m/z for $C_{26}H_{25}N_3O_5S$ $[M+H]^+$ calcd 492.15877, found 492.15845.

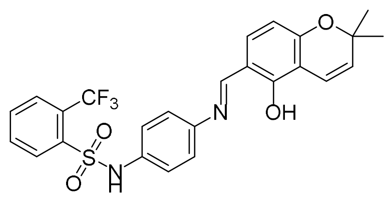

(*E*)-*N*-(4-(((5-hydroxy-2,2-dimethyl-2*H*-chromen-6-yl)methylene)amino)phenyl)-2-(trifluoromethyl)benzenesulfonamide (C22): Yield: 60.39%; yellow solid; m. p. 130−132 °C; ^{1}H NMR (500 MHz, DMSO-d_6) δ 14.22 (s, 1H, -OH), 10.80 (s, 1H, -SO$_2$NH-), 8.76 (s, 1H, -N=CH-), 8.10 (d, J = 7.8 Hz, 1H, Ar-H), 8.00 (d, J = 7.6 Hz, 1H, Ar-H), 7.89 – 7.81 (m, 2H, Ar-H), 7.32 (dd, J = 8.7, 0.8 Hz, 3H, Ar-H), 7.17 (d, J = 8.8 Hz, 2H, Ar-H), 6.62 (d, J = 10.0 Hz, 1H, Ar-H), 6.38 (d, J = 8.5 Hz, 1H, Ar-H), 5.72 (d, J = 10.0 Hz, 1H, Ar-H), 1.39 (s, 6H, -CH$_3$). ^{13}CNMR (126 MHz, DMSO-*d6*) δ 162.7, 158.6, 157.3, 143.7, 138.8, 136.3, 134.2 – 133.9, 131.1, 129.2, 122.5, 121.1, 115.9, 113.3, 109.0, 108.4, 77.6, 28.2. ^{19}F NMR (471 MHz, DMSO-d_6) δ -55.8 – -56.4 (m). HRMS (ESI) m/z for $C_{25}H_{21}F_3N_2O_4S$ [M+H]$^+$ calcd 503.12469, found 503.12451.

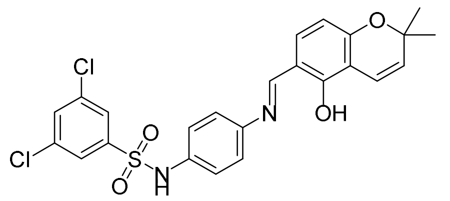

(*E*)-3,5-dichloro-*N*-(4-(((5-hydroxy-2,2-dimethyl-2*H*-chromen-6-yl)methylene)amino)phenyl) benzenesulfonamide (C23): Yield: 58.69%; yellow solid; m. p. 211−213 °C; ^{1}H NMR (500 MHz, DMSO-d_6) δ 14.19 (s, 1H, -OH), 10.61 (s, 1H, -SO$_2$NH-), 8.78 (s, 1H, -N=CH-), 7.96 (d, J = 2.1 Hz, 1H, Ar-H), 7.73 (d, J = 1.9 Hz, 2H, Ar-H), 7.33 (dd, J = 8.5, 3.9 Hz, 3H, Ar-H), 7.19 – 7.15 (m, 2H, Ar-H), 6.62 (d, J = 10.0 Hz, 1H, Ar-H), 6.38 (d, J = 8.5 Hz, 1H, Ar-H), 5.71 (d, J = 10.0 Hz, 1H, Ar-H), 1.39 (s, 6H, -CH$_3$). ^{13}C NMR (126 MHz, DMSO-d_6) δ 163.0, 158.6, 157.4, 144.5, 142.8, 135.6 (d, J = 2.6 Hz), 134.2, 133.3, 129.2, 125.7, 122.6, 122.4, 115.9, 113.3, 109.0, 108.5, 77.6, 28.2. HRMS (ESI) m/z for $C_{24}H_{20}C_{l2}N_2O_4S$ [M+H]$^+$ calcd 503.05936, found 503.05914.

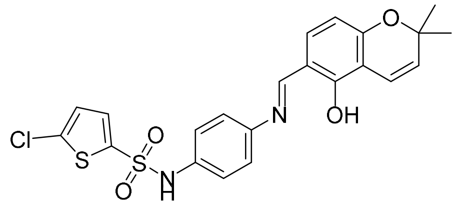

(*E*)-5-chloro-*N*-(4-(((5-hydroxy-2,2-dimethyl-2*H*-chromen-6-yl)methylene)amino)phenyl) thiophene-2-sulfonamide (C24): Yield: 72.36%; yellow solid; m. p. 141−143 °C; ^{1}H NMR (500 MHz, DMSO-d_6) δ 14.23 (s, 1H, -OH), 10.68 (s, 1H, -SO$_2$NH-), 8.80 (s, 1H, -N=CH-), 7.45 (d, J = 4.1 Hz, 1H, Ar-H), 7.35 (t, J = 8.5 Hz, 3H, Ar-H), 7.23 – 7.19 (m, 3H, Ar-H), 6.63 (d, J = 10.1 Hz, 1H, Ar-H), 6.39 (d, J = 8.5 Hz, 1H, Ar-H), 5.72 (d, J = 10.0 Hz, 1H, Ar-H), 1.39 (s, 6H, -CH$_3$). ^{13}C NMR (126 MHz, DMSO-d_6) δ 162.9, 158.6, 157.4, 144.5, 138.6, 135.8 (d, J = 14.1 Hz), 134.2, 133.0, 129.2, 128.5, 122.4 (d, J = 28.1 Hz), 116.0, 108.5, 77.6, 28.3 (d, J = 9.1 Hz). HRMS (ESI) m/z for $C_{22}H_{19}ClN_2O_4S_2$ [M+H]$^+$ calcd 475.05475, found 475.05475.

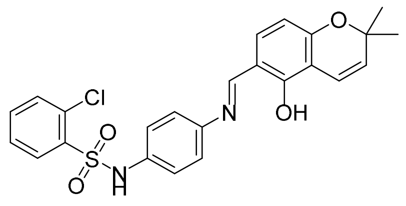

(*E*)-2-chloro-*N*-(4-(((5-hydroxy-2,2-dimethyl-2*H*-chromen-6-yl)methylene)amino)phenyl) benzenesulfonamide (C25): Yield: 59.74%; yellow solid; m. p. 166−168 °C; ^{1}H NMR (500 MHz, DMSO-d_6) δ 14.21 (s, 1H, -OH), 10.75 (s, 1H, -SO_2NH-), 8.74 (s, 1H, -N=CH-), 8.06 (d, J = 8.1 Hz, 1H, Ar-H), 7.63 (dd, J = 6.4, 1.7 Hz, 2H, Ar-H), 7.53 (ddd, J = 8.3, 6.5, 2.2 Hz, 1H, Ar-H), 7.33 – 7.26 (m, 3H, Ar-H), 7.16 (d, J = 8.8 Hz, 2H, Ar-H), 6.62 (dd, J = 9.9, 0.7 Hz, 1H, Ar-H), 6.38 (dd, J = 8.4, 0.7 Hz, 1H, Ar-H), 5.71 (d, J = 10.0 Hz, 1H, Ar-H), 1.39 (s, 6H, -CH_3). ^{13}C NMR (126 MHz, DMSO-d_6) δ 162.6, 158.6, 157.3, 143.6, 136.9, 135.9, 135.2, 134.1, 132.4, 132.1, 131.2, 129.2, 128.3, 122.4, 120.7, 115.9, 113.3, 109.0, 108.4, 77.6, 28.3. HRMS (ESI) m/z for $C_{24}H_{21}ClN_2O_4S$ $[M+H]^+$ calcd 469.09833, found 469.09818.

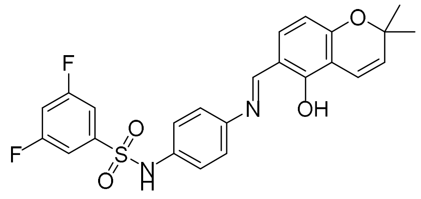

(*E*)-3,5-difluoro-*N*-(4-(((5-hydroxy-2,2-dimethyl-2*H*-chromen-6-yl)methylene)amino)phenyl) benzenesulfonamide (C26): Yield: 70.15%; yellow solid; m. p. 207−209 °C; ^{1}H NMR (500 MHz, DMSO-d_6) δ 14.20 (s, 1H, -OH), 10.61 (s, 1H, -SO_2NH-), 8.78 (s, 1H, -N=CH-), 7.64 (ddd, J = 9.2, 6.8, 2.4 Hz, 1H, Ar-H), 7.46 (d, J = 6.7 Hz, 2H, Ar-H), 7.33 (d, J = 8.3 Hz, 3H, Ar-H), 7.17 (d, J = 7.7 Hz, 2H, Ar-H), 6.63 (d, J = 10.0 Hz, 1H, Ar-H), 6.38 (d, J = 8.4 Hz, 1H, Ar-H), 5.72 (d, J = 9.9 Hz, 1H, Ar-H), 1.39 (s, 6H, -CH_3). ^{13}C NMR (126 MHz, DMSO-d_6) δ 163.6 (d, J = 12.3 Hz), 163.0, 161.6 (d, J = 12.3 Hz), 158.6, 157.4, 144.4, 143.1 (t, J = 8.4 Hz), 135.7, 134.2, 129.2, 122.6, 122.3, 115.9, 113.3, 111.1, 110.9, 109.5, 109.0, 108.5, 77.6, 28.2.

HRMS (ESI) m/z for $C_{24}H_{20}F_2N_2O_4S$ $[M+H]^+$ calcd 471.11846, found 471.11884.

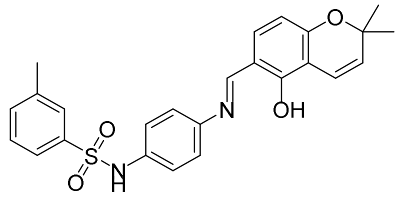

(*E*)-*N*-(4-(((5-hydroxy-2,2-dimethyl-2*H*-chromen-6-yl)methylene)amino)phenyl)-3-methylbenzenesulfonamide (C27): Yield: 65.13%; yellow solid; m. p. 152−154 °C; ^{1}H NMR (500 MHz, DMSO-d_6) δ 14.26 (s, 1H, -OH), 10.38 (s, 1H, -SO_2NH-), 8.76 (s, 1H, -N=CH-), 7.61 (d, J = 2.1 Hz, 1H, Ar-H), 7.56 (dt, J = 6.1, 2.6 Hz, 1H, Ar-H), 7.45 – 7.40 (m, 2H, Ar-H), 7.30 (dd, J = 13.4, 8.7 Hz, 3H, Ar-H), 7.15 (d, J = 8.7 Hz, 2H, Ar-H), 6.62 (d, J = 10.0 Hz, 1H, Ar-H), 6.38 (d, J = 8.6 Hz, 1H, Ar-H), 5.71 (d, J = 10.0 Hz, 1H, Ar-H), 2.34 (s, 3H, -CH_3), 1.39 (s, 6H, -CH_3). ^{13}C NMR (126 MHz, DMSO-d_6) δ 162.5, 158.6, 157.3, 143.6, 139.9, 139.5, 136.7, 134.1, 129.7, 129.2, 127.3, 124.3, 122.4, 121.4, 116.0, 113.3, 109.0, 108.4, 77.6, 28.2, 21.3. HRMS (ESI) m/z for $C_{25}H_{24}N_2O_4S$ $[M+H]^+$ calcd 449.15295, found 449.15326.

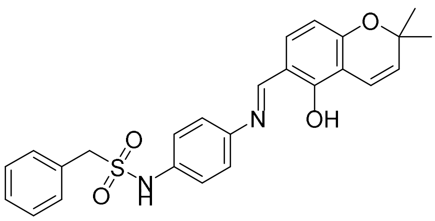

(*E*)-*N*-(4-(((5-hydroxy-2,2-dimethyl-2*H*-chromen-6-yl)methylene)amino)phenyl)-1-phenylmethanesulfonamide (C28): Yield: 49.88%; yellow solid; m. p. 143−145 °C; ^{1}H NMR (500 MHz, DMSO-*d*$_6$) δ 14.40 (s, 1H, -OH), 9.98 (s, 1H, -SO$_2$NH-), 8.85 (s, 1H, -N=CH-), 7.42 – 7.35 (m, 6H, Ar-H), 7.29 – 7.24 (m, 4H, Ar-H), 6.66 (d, *J* = 9.9 Hz, 1H, Ar-H), 6.40 (d, *J* = 8.5 Hz, 1H, Ar-H), 5.73 (d, *J* = 10.0 Hz, 1H, Ar-H), 4.49 (s, 2H, -CH$_2$-), 1.41 (s, 6H, -CH$_3$). ^{13}C NMR (126 MHz, DMSO-*d*$_6$) δ 162.2, 158.7, 157.32, 142.9, 137.5, 134.1, 131.5, 130.0, 129.2, 128.9, 128.8, 122.5, 120.3, 116.0, 113.4, 109.0, 108.4, 77.6, 57.3, 28.3. HRMS (ESI) m/z for $C_{25}H_{24}N_2O_4S$ [M+H]$^+$ calcd 449.15295, found 449.15308.

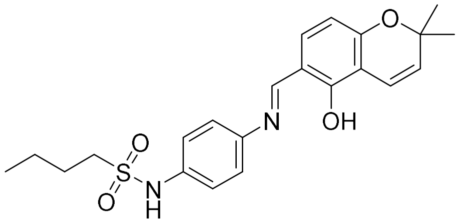

(*E*)-*N*-(4-(((5-hydroxy-2,2-dimethyl-2*H*-chromen-6-yl)methylene)amino)phenyl)butane-1-sulfonamide (C29): Yield: 39.58%; yellow solid; m. p. 132−134 °C; ^{1}H NMR (500 MHz, DMSO-*d*$_6$) δ 14.36 (s, 1H -OH), 9.91 (s, 1H, -SO$_2$NH-), 8.83 (s, 1H, -N=CH-), 7.39 (d, *J* = 8.7 Hz, 2H, Ar-H), 7.36 (d, *J* = 8.5 Hz, 1H, Ar-H), 7.26 (d, *J* = 8.7 Hz, 2H, Ar-H), 6.64 (d, *J* = 9.8 Hz, 1H, Ar-H), 6.40 (d, *J* = 8.5 Hz, 1H, Ar-H), 5.73 (d, *J* = 9.9 Hz, 1H, Ar-H), 3.13 – 3.06 (m, 2H, -CH$_2$-), 1.64 (tt, *J* = 7.8, 6.7 Hz, 2H, -CH$_2$-), 1.40 (s, 6H, -CH$_3$), 1.37 – 1.32 (m, 2H -CH$_2$-), 0.83 (t, *J* = 7.4 Hz, 3H, -CH$_3$). ^{13}C NMR (126 MHz, DMSO-*d*$_6$) δ 162.3, 158.7, 157.3, 143.2, 137.4, 134.1, 129.2, 122.6, 120.8, 116.0, 113.3, 109.0, 108.4, 77.6, 50.7, 28.2, 25.6, 21.2, 14.0. HRMS (ESI) m/z for $C_{22}H_{26}N_2O_4S$ [M+H]$^+$ calcd 415.16860, found 415.16876.

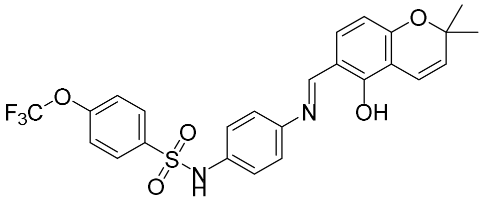

(*E*)-*N*-(4-(((5-hydroxy-2,2-dimethyl-2*H*-chromen-6-yl)methylene)amino)phenyl)-4-(trifluoromethoxy)benzenesulfonamide (C30): Yield: 48.39%; yellow solid; m. p. 128−130 °C; ^{1}H NMR (500 MHz, DMSO-*d*$_6$) δ 14.22 (s, 1H, -OH), 10.54 (s, 1H, -SO$_2$NH-), 8.77 (s, 1H, -N=CH-), 7.94 – 7.84 (m, 2H, Ar-H), 7.57 (d, *J* = 8.5 Hz, 2H, Ar-H), 7.32 (dd, *J* = 8.5, 5.8 Hz, 3H, Ar-H), 7.15 (d, *J* = 8.9 Hz, 2H, Ar-H), 6.62 (d, *J* = 10.0 Hz, 1H, Ar-H), 6.38 (d, *J* = 8.5 Hz, 1H, Ar-H), 5.72 (d, *J* = 10.0 Hz, 1H, Ar-H), 1.39 (s, 6H, -CH$_3$). ^{13}C NMR (126 MHz, DMSO-*d*$_6$) δ 162.8, 158.6, 157.3, 151.6, 144.0, 138.7, 136.2, 134.2, 129.8, 129.2, 122.5, 121.9 (d, *J* = 16.3 Hz), 115.9, 113.3, 109.0, 108.5, 77.6, 28.2.^{19}F NMR (471 MHz, DMSO-*d*$_6$) δ -56.6. HRMS (ESI) m/z for $C_{25}H_{21}F_3N_2O_5S$ [M+H]+ calcd 519.11960, found 519.11987.

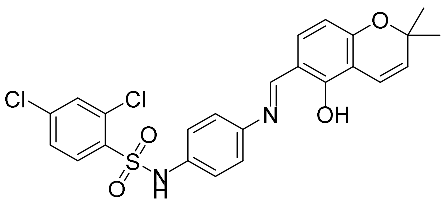

(*E*)-2,4-dichloro-*N*-(4-(((5-hydroxy-2,2-dimethyl-2*H*-chromen-6-yl)methylene)amino)phenyl) benzenesulfonamide (C31): Yield: 68.96%; yellow solid; m. p. 154−156 °C; ^{1}H NMR (500 MHz, DMSO-d_6) δ 14.20 (s, 1H, -OH), 10.84 (s, 1H, -SO_2NH-), 8.74 (s, 1H, -N=CH-), 8.03 (d, J = 8.6 Hz, 1H, Ar-H), 7.87 (d, J = 2.1 Hz, 1H, Ar-H), 7.62 (dd, J = 8.6, 2.1 Hz, 1H, Ar-H), 7.30 (t, J = 8.9 Hz, 3H, Ar-H), 7.15 (d, J = 8.8 Hz, 2H, Ar-H), 6.62 (d, J = 10.0 Hz, 1H, Ar-H), 6.38 (d, J = 8.5 Hz, 1H, Ar-H), 5.71 (d, J = 10.0 Hz, 1H, Ar-H), 1.39 (s, 6H, -CH_3). ^{13}C NMR (126 MHz, DMSO-d_6) δ 162.7, 158.5, 157.3, 143.8, 139.3, 135.8, 135.6, 134.1, 133.5, 132.5, 131.9, 129.2, 128.5, 122.5, 120.9, 115.9, 113.3, 109.0, 108.5, 77.6, 28.2. HRMS (ESI) m/z for $C_{24}H_{20}Cl_2N_2O_4S$ $[M+H]^+$ calcd 503.05936, found 503.05969.

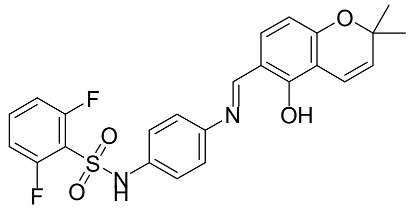

(*E*)-2,6-difluoro-*N*-(4-(((5-hydroxy-2,2-dimethyl-2*H*-chromen-6-yl)methylene)amino)phenyl) benzenesulfonamide (C32): Yield: 63.55%; yellow solid; m. p. 191−193 °C; ^{1}H NMR (500 MHz, DMSO-d_6) δ 14.20 (s, 1H, -OH), 11.01 (s, 1H, -SO_2NH-), 8.77 (s, 1H, -N=CH-), 7.70 (tt, J = 8.4, 6.0 Hz, 1H, Ar-H), 7.35 – 7.26 (m, 5H, Ar-H), 7.20 (d, J = 8.8 Hz, 2H, Ar-H), 6.62 (d, J = 10.0 Hz, 1H, Ar-H), 6.38 (d, J = 8.5 Hz, 1H, Ar-H), 5.71 (d, J = 10.0 Hz, 1H, Ar-H), 1.39 (s, 6H, -CH_3). ^{13}C NMR (126 MHz, DMSO-*d6*) δ 162.8, 158.6, 157.3, 144.0, 136.7, 135.8, 134.2, 129.2, 122.6, 120.8, 116.9, 115.9, 114.1 (d, J = 3.0 Hz), 114.0 (d, J = 3.4 Hz), 113.3, 109.0, 108.5, 77.6, 28.2. ^{19}F NMR (471 MHz, DMSO-d_6) δ -107.6. HRMS (ESI) m/z for $C_{24}H_{20}F_2N_2O4_S$ $[M+H]^+$ calcd 471.11846, found 471.11874.

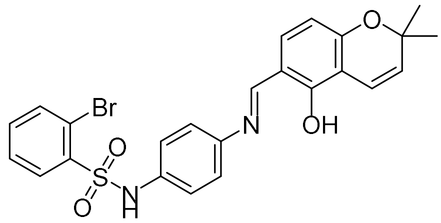

(*E*)-2-bromo-*N*-(4-(((5-hydroxy-2,2-dimethyl-2*H*-chromen-6-yl)methylene)amino)phenyl) benzenesulfonamide (C33): Yield: 60.42%; yellow solid; m. p. 170−172 °C; ^{1}H NMR (500 MHz, DMSO-d_6) δ 14.22 (s, 1H, -OH), 10.76 (s, 1H, -SO_2NH-), 8.74 (s, 1H, -N=CH-), 8.09 (dd, J = 7.8, 1.8 Hz, 1H, Ar-H), 7.82 (dd, J = 7.7, 1.5 Hz, 1H, Ar-H), 7.55 (dtd, J = 25.1, 7.5, 1.5 Hz, 2H, Ar-H), 7.29 (dd, J = 11.9, 8.6 Hz, 3H, Ar-H), 7.15 (d, J = 8.8 Hz, 2H, Ar-H), 6.62 (d, J = 9.9 Hz, 1H, Ar-H), 6.37 (d, J = 8.5 Hz, 1H, Ar-H), 5.71 (d, J = 10.0 Hz, 1H, Ar-H), 1.38 (s, 6H, -CH_3). ^{13}C NMR (126 MHz, DMSO-d_6) δ 162.6, 158.6, 157.3, 143.4, 138.5, 136.0, 135.2, 134.1, 132.4, 129.2, 128.8, 122.4, 120.5, 119.7, 115.9, 113.3, 109.0, 108.4, 77.6, 28.2. HRMS (ESI) m/z for $C_{24}H_{21}BrN_2O_4S$ $[M+H]^+$ calcd 513.04782, found 513.04834.

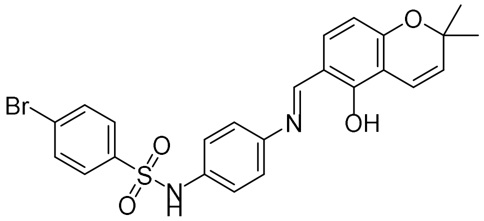

(*E*)-4-bromo-*N*-(4-(((5-hydroxy-2,2-dimethyl-2*H*-chromen-6-yl)methylene)amino)phenyl) benzenesulfonamide (C34): Yield: 53.64%; yellow solid; m. p. 158−160 °C; ^{1}H NMR (500 MHz, DMSO-d_6) δ 14.17 (s, 1H), 10.42 (s, 1H, -SO$_2$NH-), 8.72 (s, 1H, -N=CH-), 7.74 (d, J = 8.6 Hz, 2H, Ar-H), 7.64 (d, J = 8.6 Hz, 2H, Ar-H), 7.27 (dd, J = 10.9, 8.7 Hz, 4H, Ar-H), 7.10 (d, J = 8.7 Hz, 2H, Ar-H), 6.59 (d, J = 10.1 Hz, 1H, Ar-H), 6.34 (d, J = 8.4 Hz, 1H, Ar-H), 5.68 (d, J = 10.0 Hz, 1H, Ar-H), 1.35 (s, 6H, -CH$_3$). ^{13}C NMR (126 MHz, DMSO-d_6) δ 162.7, 158.6, 157.4, 144.0, 139.1, 136.2, 134.1, 132.9, 129.2 (d, J = 4.3 Hz), 127.4, 122.5, 122.0, 115.9, 113.3, 109.0, 108.5, 77.6, 28.3. HRMS (ESI) m/z for $C_{24}H_{21}BrN_2O_4S$ [M+H]$^+$ calcd 513.04782, found 513.04834.

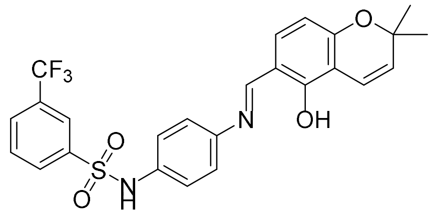

(*E*)-*N*-(4-(((5-hydroxy-2,2-dimethyl-2*H*-chromen-6-yl)methylene)amino)phenyl)-3-(trifluoromethyl)benzenesulfonamide (C35): Yield: 47.36%; yellow solid; m. p. 123−125 °C; ^{1}H NMR (500 MHz, DMSO-d_6) δ 14.19 (s, 1H, -OH), 10.55 (s, 1H, -SO$_2$NH-), 8.76 (s, 1H, -N=CH-), 8.04 (d, J = 2.3 Hz, 2H, Ar-H), 8.03 – 8.02 (m, 1H, Ar-H), 7.82 (t, J = 7.9 Hz, 1H, Ar-H), 7.32 (dd, J = 8.7, 5.4 Hz, 3H, Ar-H), 7.18 – 7.12 (m, 2H, Ar-H), 6.62 (d, J = 10.1 Hz, 1H, Ar-H), 6.38 (d, J = 8.6 Hz, 1H, Ar-H), 5.72 (d, J = 10.0 Hz, 1H, Ar-H), 1.39 (s, 6H, -CH$_3$). ^{13}C NMR (126 MHz, DMSO-d_6) δ 162.8, 158.6, 157.4, 144.4, 141.0, 135.9, 134.2, 131.5, 131.2, 130.3, 129.2, 123.7, 122.5 (d, J = 17.2 Hz), 115.9, 113.3, 109.0, 108.5, 77.6, 28.2. HRMS (ESI) m/z for $C_{25}H_{21}F_3N_2O_4S$ [M+H]$^+$ calcd 503.12469, found 503.12473.

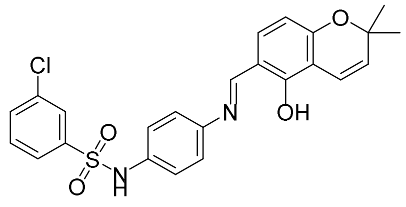

(*E*)-3-chloro-*N*-(4-(((5-hydroxy-2,2-dimethyl-2*H*-chromen-6-yl)methylene)amino)phenyl) benzenesulfonamide (C36): Yield: 71.36%; yellow solid; m. p. 189−191 °C; ^{1}H NMR (500 MHz, DMSO-d_6) δ 14.21 (s, 1H, -OH), 10.51 (s, 1H, -SO$_2$NH-), 8.77 (s, 1H, -N=CH-), 7.78 (t, J = 2.0 Hz, 1H, Ar-H), 7.73 – 7.69 (m, 2H, Ar-H), 7.60 (t, J = 8.0 Hz, 1H, Ar-H), 7.32 (dd, J = 8.7, 4.8 Hz, 3H, Ar-H), 7.15 (d, J = 8.7 Hz, 2H, Ar-H), 6.62 (d, J = 10.0 Hz, 1H, Ar-H), 6.38 (d, J = 8.6 Hz, 1H, Ar-H), 5.72 (d, J = 10.0 Hz, 1H, Ar-H), 1.39 (s, 6H, -CH$_3$). ^{13}C NMR (126 MHz, DMSO-d_6) δ 162.8, 158.6, 157.3, 144.1, 141.6, 136.1, 134.4, 134.2, 133.5, 132.0, 129.2, 126.7, 125.9, 122.5, 122.0, 115.9, 113.3, 109.0, 108.5, 77.6, 28.2. HRMS (ESI) m/z for $C_{24}H_{21}ClN_2O_4S$ [M+H]$^+$ calcd 469.09833, found 469.09866.

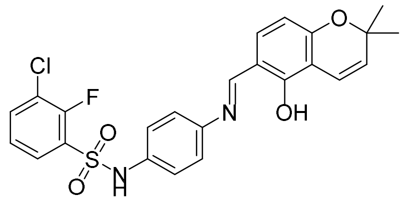

(*E*)-3-chloro-2-fluoro-*N*-(4-(((5-hydroxy-2,2-dimethyl-2*H*-chromen-6-yl)methylene)amino)phenyl) benzenesulfonamide (C37): Yield: 65.11%; yellow solid; m. p. 193−195 °C; ^{1}H NMR (500 MHz, DMSO-d_6) δ 14.20 (s, 1H, -OH), 10.94 (s, 1H, -SO_2NH-), 8.76 (s, 1H, -N=CH-), 7.93 – 7.87 (m, 1H, Ar-H), 7.81 (ddd, J = 7.9, 6.4, 1.7 Hz, 1H, Ar-H), 7.40 (t, J = 8.1 Hz, 1H, Ar-H), 7.32 (dd, J = 8.7, 2.5 Hz, 3H, Ar-H), 7.17 (d, J = 8.7 Hz, 2H, Ar-H), 6.62 (d, J = 10.0 Hz, 1H, Ar-H), 6.38 (d, J = 8.5 Hz, 1H, Ar-H), 5.71 (d, J = 10.0 Hz, 1H, Ar-H), 1.39 (s, 6H, -CH_3). ^{13}C NMR (126 MHz, DMSO-*d6*) δ 162.8, 158.6, 157.3, 144.1, 136.5, 135.6, 134.2, 129.8, 129.3 – 128.9 (m), 126.4 (d, J = 4.8 Hz), 122.6, 122.0 (d, J = 17.4 Hz), 121.5, 115.9, 113.3, 109.0, 108.5, 77.6, 28.2. ^{19}F NMR (471 MHz, DMSO-d_6) δ -112.7. HRMS (ESI) m/z for $C_{24}H_{20}ClFN_2O_4S$ $[M+H]^+$ calcd 487.08891, found 48.08920.

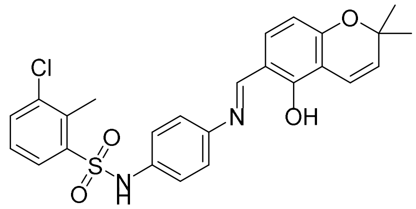

(*E*)-3-chloro-*N*-(4-(((5-hydroxy-2,2-dimethyl-2*H*-chromen-6-yl)methylene)amino)phenyl)-2-methylbenzenesulfonamide (C38): Yield: 75.61; yellow solid; m. p. 193−195 °C; ^{1}H NMR (500 MHz, DMSO-d_6) δ 14.22 (s, 1H, -OH), 10.74 (s, 1H, -SO_2NH-), 8.75 (s, 1H, -N=CH-), 7.90 (dd, J = 8.0, 1.3 Hz, 1H, Ar-H), 7.72 (dd, J = 7.9, 1.3 Hz, 1H, Ar-H), 7.40 (t, J = 8.0 Hz, 1H, Ar-H), 7.30 (dd, J = 10.8, 8.7 Hz, 3H, Ar-H), 7.13 (d, J = 8.7 Hz, 2H, Ar-H), 6.62 (d, J = 10.0 Hz, 1H, Ar-H), 6.38 (d, J = 8.5 Hz, 1H, Ar-H), 5.71 (d, J = 10.0 Hz, 1H, Ar-H), 2.65 (s, 3H, -CH_3), 1.39 (s, 6H, -CH_3). ^{13}C NMR (126 MHz, DMSO-d_6) δ 162.6, 158.5, 157.3, 143.5, 140.2, 136.4, 136.1, 134.8, 134.4, 134.1, 129.2 (d, J = 17.0 Hz), 128.0, 122.5, 120.6, 115.9, 113.3, 109.0, 108.4, 77.6, 28.2, 17.0. HRMS (ESI) m/z for $C_{25}H_{23}ClN_2O_4S$ $[M+H]^+$ calcd 483.11398, found 483.11441.

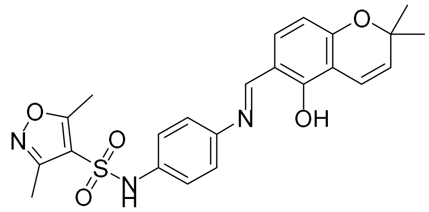

(*E*)-*N*-(4-(((5-hydroxy-2,2-dimethyl-2*H*-chromen-6-yl)methylene)amino)phenyl)-3,5-dimethylisoxazole-4-sulfonamide (C39): Yield: 58.90%; yellow solid; m. p. 195−197 °C; ^{1}H NMR (500 MHz, DMSO-d_6) δ 14.22 (s, 1H, -OH), 10.56 (s, 1H, -SO_2NH-), 8.81 (s, 1H, -N=CH-), 7.36 (dd, J = 10.8, 8.7 Hz, 3H, Ar-H), 7.15 (d, J = 8.6 Hz, 2H, Ar-H), 6.63 (d, J = 10.0 Hz, 1H, Ar-H), 6.40 (d, J = 8.6 Hz, 1H, Ar-H), 5.73 (d, J = 10.0 Hz, 1H), 2.47 (s, 3H, -CH_3), 2.24 (s, 3H, -CH_3), 1.40 (s, 6H, -CH_3). ^{13}C NMR (126 MHz, DMSO-d_6) δ 174.1, 163.1, 158.6, 157.8, 157.4, 144.6, 135.5, 134.2, 129.3, 122.7 (d, J = 13.8 Hz), 115.9, 115.6, 113.3, 109.0, 108.5, 77.6, 28.2, 12.7, 10.8. HRMS (ESI) m/z for $C_{23}H_{23}N_3O_5S$ $[M+H]^+$ calcd 454.14312, found 454.14328.

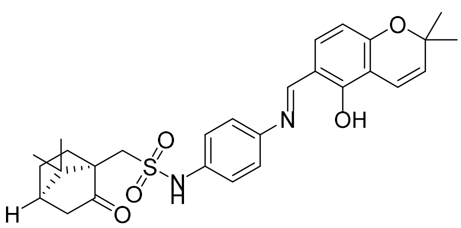

1-((1*S*,4*R*)-7,7-dimethyl-2-oxobicyclo[2.2.1]heptan-1-yl)-*N*-(4-(((*E*)-(5-hydroxy-2,2-dimethyl-2*H*-chromen-6-yl)methylene)amino)phenyl)methanesulfonamide (C40): Yield: 51.23%; yellow solid; m. p. 186−188 °C; ^{1}H NMR (500 MHz, DMSO-d_6) δ 14.36 (s, 1H, -OH), 9.97 (s, 1H, -SO$_2$NH-), 8.85 (s, 1H -N=CH-), 7.42 (d, J = 8.8 Hz, 2H, Ar-H), 7.37 (s, 1H, Ar-H), 7.31 (d, J = 8.9 Hz, 2H, Ar-H), 6.65 (d, J = 10.0 Hz, 1H, Ar-H), 6.40 (d, J = 8.5 Hz, 1H, Ar-H), 5.73 (d, J = 10.0 Hz, 1H, Ar-H), 3.41 (d, J = 15.1 Hz, 1H, -CH$_2$-), 3.00 (d, J = 15.0 Hz, 1H -CH$_2$-), 2.42 – 2.29 (m, 2H, -CH$_2$-), 2.05 (t, J = 4.5 Hz, 1H,-CH(CH$_2$)CH), 1.92 (d, J = 18.5 Hz, 2H, -CH$_2$-), 1.54 (ddd, J = 13.6, 9.3, 4.5 Hz, 1H, -CH$_2$-), 1.45 – 1.41 (m, 1H, -CH$_2$-), 1.40 (s, 6H, -CH$_3$), 1.00 (s, 3H, -CH$_3$), 0.76 (s, 3H, -CH$_3$). ^{13}C NMR (126 MHz, DMSO-d_6) δ 214.8, 162.4, 158.7, 157.3, 137.5, 134.1, 129.2, 120.6, 116.0, 113.3, 109.0, 108.4, 77.6, 48.1 (d, J = 9.7 Hz), 42.5 (d, J = 7.9 Hz), 28.2, 26.7, 25.1, 19.9, 19.8. HRMS (ESI) m/z for $C_{28}H_{32}N_2O_5S$ [M+H]$^+$ calcd 509.21047, found 509.21069.

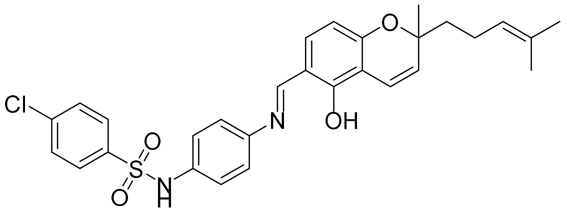

(*E*)-4-chloro-*N*-(4-(((5-hydroxy-2-methyl-2-(4-methylpent-3-en-1-yl)-2*H*-chromen-6-yl)methylene) amino)phenyl)benzenesulfinamide (C41): Yield: 68.36%; yellow solid; m. p. 115−117 °C; ^{1}H NMR (500 MHz, DMSO-d_6) δ 14.20 (s, 1H, -OH), 10.44 (s, 1H, -SO$_2$NH-), 8.75 (s, 1H, -N=CH-), 7.76 (d, J = 8.6 Hz, 2H, Ar-H), 7.64 (d, J = 8.6 Hz, 2H, Ar-H), 7.34 – 7.28 (m, 3H, Ar-H), 7.14 (d, J = 8.7 Hz, 2H, Ar-H), 6.67 (d, J = 10.2 Hz, 1H, Ar-H), 6.38 (d, J = 8.6 Hz, 1H, Ar-H), 5.68 (d, J = 10.2 Hz, 1H, Ar-H), 5.07 (tt, J = 7.2, 1.5 Hz, 1H, -CH=C(CH$_3$)$_2$), 2.03 (q, J = 8.0 Hz, 2H, -CH$_2$-), 1.73 – 1.63 (m, 2H, -CH$_2$-), 1.60 (d, J = 1.5 Hz, 3H, -CH$_3$), 1.51 (d, J = 1.3 Hz, 3H, -CH$_3$), 1.36 (s, 3H, -CH$_3$). ^{13}C NMR (126 MHz, DMSO-*d6*) δ 162.7, 158.6, 144.0, 138.6, 138.4, 136.2, 134.2, 131.5, 130.0, 129.1, 128.0, 124.3, 122.5, 121.9, 116.5, 113.2, 108.8, 108.3, 79.9, 41.3, 27.0, 25.9, 22.7, 17.9. HRMS (ESI) m/z for $C_{29}H_{29}ClN_2O_4S$ [M+H]$^+$ calcd 537.16093, found 537.16113.

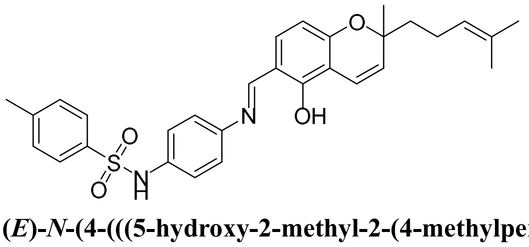

(*E*)-*N*-(4-(((5-hydroxy-2-methyl-2-(4-methylpent-3-en-1-yl)-2*H*-chromen-6-yl)methylene) amino)phenyl)-4-methylbenzenesulfonamide (C42): Yield: 55.90%; yellow solid; m. p. 95−97 °C; ^{1}H NMR (500 MHz, DMSO-d_6) δ 14.26 (s, 1H, -OH), 10.35 (s, 1H, -SO$_2$NH-), 8.75 (s, 1H, -N=CH-), 7.65 (d, J = 8.3 Hz, 2H, Ar-H), 7.35 (d, J = 8.1 Hz, 2H, Ar-H), 7.30 (dd, J = 13.5, 8.6 Hz, 3H, Ar-H), 7.14 (d, J = 8.9 Hz, 2H, Ar-H), 6.66 (d, J = 10.1 Hz, 1H, Ar-H), 6.38 (d, J = 8.5 Hz, 1H, Ar-H), 5.68 (d, J = 10.2 Hz, 1H, Ar-H), 5.07 (t, J = 7.2 Hz, 1H, -CH=C(CH$_3$)$_2$), 2.32 (s, 3H, -CH$_3$), 2.02 (q, J = 8.0 Hz, 2H, -

CH_2-), 1.70 – 1.63 (m, 2H, -CH_2-), 1.60 (s, 3H, -CH_3), 1.50 (s, 3H, -CH_3), 1.35 (s, 3H, -CH_3). ^{13}C NMR (126 MHz, DMSO-d_6) δ 162.4, 158.6, 157.7, 143.8, 143.5, 137.0, 136.7, 134.1, 131.5, 130.2, 128.0, 127.2, 124.3, 122.4, 121.4, 116.5, 113.2, 108.8, 108.2, 79.9, 41.3, 27.0, 25.9, 22.7, 21.4, 17.9. HRMS (ESI) m/z for $C_{30}H_{32}N_2O_4S$ $[M+H]^+$ calcd 517.21555, found 517.21582.

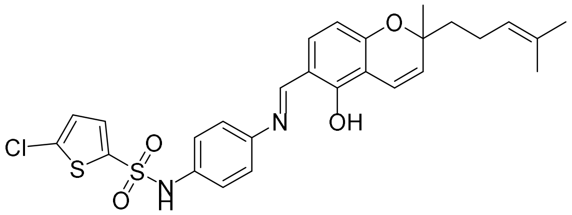

(*E*)-5-chloro-*N*-(4-(((5-hydroxy-2-methyl-2-(4-methylpent-3-en-1-yl)-2*H*-chromen-6-yl)methylene) amino)phenyl)thiophene-2-sulfonamide (C43): Yield: 65.36%; yellow solid; m. p. 97−99 °C; ^{1}H NMR (500 MHz, DMSO-d_6) δ 14.23 (s, 1H, -OH), 10.67 (s, 1H, -SO_2NH-), 8.80 (s, 1H, -N=CH-), 7.44 (d, J = 4.1 Hz, 1H, Ar-H), 7.35 (dd, J = 11.6, 8.6 Hz, 3H, Ar-H), 7.23 – 7.18 (m, 3H, Ar-H), 6.68 (d, J = 10.2 Hz, 1H, Ar-H), 6.39 (d, J = 8.4 Hz, 1H, Ar-H), 5.69 (d, J = 10.0 Hz, 1H, Ar-H), 5.08 (t, J = 7.3 Hz, 1H, -CH=C$(CH_3)_2$), 2.03 (d, J = 8.0 Hz, 2H, -CH_2-), 1.73 – 1.62 (m, 2H, -CH_2-), 1.60 (s, 3H, -CH_3), 1.51 (s, 3H, -CH_3), 1.36 (s, 3H, -CH_3). ^{13}C NMR (126 MHz, DMSO-d_6) δ 162.9, 158.6, 157.7, 144.4, 138.5, 135.8 (d, J = 8.8 Hz), 134.2, 133.0, 131.5, 128.5, 128.1, 124.3, 122.5, 122.3, 116.5, 113.2, 108.8, 108.3, 80.0, 41.3, 27.0, 25.9, 22.7, 17.9. HRMS (ESI) m/z for $C_{27}H_{27}ClN_2O_4S_2$ $[M+H]^+$ calcd 543.11735, found 543.11755.

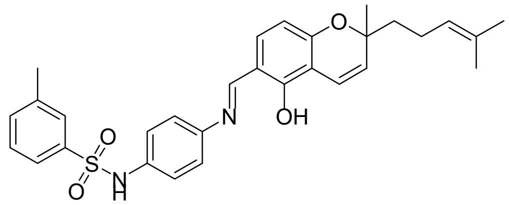

(*E*)-*N*-(4-(((5-hydroxy-2-methyl-2-(4-methylpent-3-en-1-yl)-2*H*-chromen-6-yl)methylene)amino) phenyl)-3-methylbenzenesulfonamide (C44): Yield: 57.20%; yellow solid; m. p. 84-86 °C; ^{1}H NMR (500 MHz, DMSO-d_6) δ 14.25 (s, 1H, -OH), 10.38 (s, 1H, -SO_2NH-), 8.75 (s, 1H, -N=CH-), 7.61 (s, 1H, Ar-H), 7.58 – 7.54 (m, 1H, Ar-H), 7.46 – 7.41 (m, 2H, Ar-H), 7.32 – 7.27 (m, 3H, Ar-H), 7.15 (d, J = 8.7 Hz, 2H, Ar-H), 6.66 (d, J = 10.2 Hz, 1H, Ar-H), 6.37 (d, J = 8.5 Hz, 1H, Ar-H), 5.67 (d, J = 10.1 Hz, 1H, Ar-H), 5.07 (t, J = 7.2 Hz, 1H, -CH=C$(CH_3)_2$), 2.34 (s, 3H, -CH_3), 2.02 (q, J = 7.9 Hz, 2H, -CH_2-), 1.69 – 1.62 (m, 2H, -CH_2-), 1.60 (s, 3H, -CH_3), 1.50 (s, 3H, -CH_3), 1.35 (s, 3H, -CH_3). ^{13}C NMR (126 MHz, DMSO-d_6) δ 162.5, 158.6, 157.7, 143.6, 139.5, 136.7, 134.1, 131.5, 129.6, 128.0, 127.3, 124.3, 122.4, 121.4, 116.5, 113.2, 108.8, 108.2, 79.9, 41.3, 27.0, 25.9, 22.7, 21.3, 17.9. HRMS (ESI) m/z for $C_{30}H_{32}N_2O_4S$ $[M+H]^+$ calcd 517.21600, found 517.21555.

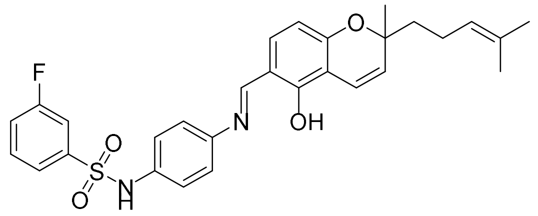

(*E*)-3-fluoro-*N*-(4-(((5-hydroxy-2-methyl-2-(4-methylpent-3-en-1-yl)-2*H*-chromen-6-yl)methylene) amino)phenyl)benzenesulfonamide (C45): Yield: 54.12%; yellow solid; m. p. 113−115 °C; ^{1}H NMR (500 MHz, DMSO-d_6) δ 14.22 (s, 1H, -OH), 10.52 (s, 1H, -SO_2NH-), 8.76 (s, 1H, -N=CH-), 7.66 – 7.59 (m, 2H, Ar-H), 7.56 (dt, J = 8.2, 2.1 Hz, 1H, Ar-H), 7.53 – 7.48 (m, 1H, Ar-H), 7.31 (dd, J = 8.7, 4.3 Hz, 3H, Ar-H), 7.16 (d, J = 8.8 Hz, 2H, Ar-H), 6.66 (d, J = 10.1 Hz, 1H, Ar-H), 6.38 (d, J = 8.5 Hz, 1H, Ar-H), 5.68 (d, J = 10.0 Hz, 1H, Ar-H), 5.07 (t, J = 7.2 Hz, 1H, -CH=C(CH_3)$_2$), 2.02 (q, J = 7.9 Hz, 2H, -CH_2-), 1.70 – 1.62 (m, 2H, -CH_2-), 1.60 (s, 3H, -CH_3), 1.50 (s, 3H, -CH_3), 1.35 (s, 3H, -CH_3). ^{13}C NMR (126 MHz, DMSO-d_6) δ 162.7, 158.5, 157.7, 144.1, 141.8 (d, J = 6.6 Hz), 136.1, 134.2, 132.3 (d, J = 7.7 Hz), 131.5, 128.0, 124.3, 123.5 (d, J = 3.0 Hz), 122.5, 121.9, 120.8, 116.5, 114.3, 114.1, 113.2, 108.3, 79.9, 41.3, 27.0, 25.9, 22.7, 17.9. ^{19}F NMR (471 MHz, DMSO-d_6) δ -110.1. HRMS (ESI) m/z for $C_{29}H_{29}FN_2O_4S$ [M+H]$^+$ calcd 521.19048, found 521.19080.

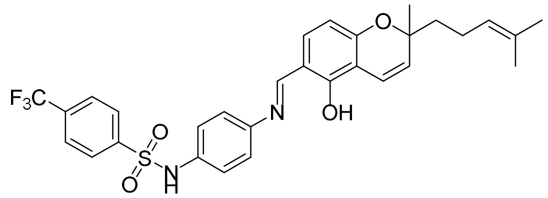

(*E*)-*N*-(4-(((5-hydroxy-2-methyl-2-(4-methylpent-3-en-1-yl)-2*H*-chromen-6-yl)methylene)amino) phenyl)-4-(trifluoromethyl)benzenesulfonamide (C46): Yield: 50.36%; yellow solid; m. p. 130−132 °C; ^{1}H NMR (500 MHz, DMSO-d_6) δ 14.16 (s, 1H, -OH), 10.60 (s, 1H, -SO_2NH-), 8.72 (s, 1H, -N=CH-), 7.93 (s, 4H, Ar-H), 7.27 (dd, J = 8.6, 1.7 Hz, 3H, Ar-H), 7.11 (d, J = 8.9 Hz, 2H, Ar-H), 6.62 (d, J = 10.2 Hz, 1H, Ar-H), 6.34 (d, J = 8.5 Hz, 1H, Ar-H), 5.64 (d, J = 10.1 Hz, 1H, Ar-H), 5.03 (d, J = 1.6 Hz, 1H, -CH=C(CH_3)$_2$)), 1.98 (q, J = 7.9 Hz, 2H, -CH_2-), 1.62 (t, J = 8.8 Hz, 2H, -CH_2-), 1.57 – 1.54 (m, 3H, -CH_3), 1.46 (d, J = 1.3 Hz, 3H, -CH_3), 1.31 (s, 3H, -CH_3). ^{13}C NMR (126 MHz, DMSO-d_6) δ 162.8, 158.5, 144.2, 143.7, 135.9, 134.2, 131.5, 128.1 (d, J = 14.7 Hz), 127.1 (d, J = 3.9 Hz), 124.3, 122.6, 122.1, 116.4, 113.2, 108.8, 108.3, 79.9, 41.3, 27.0, 25.9, 22.7, 17.9. ^{19}F NMR (471 MHz, DMSO-d_6) δ -61.53. HRMS (ESI) m/z for $C_{30}H_{29}F_3N_2O_4S$ [M+H]$^+$ calcd 571.18729, found 571.18762.

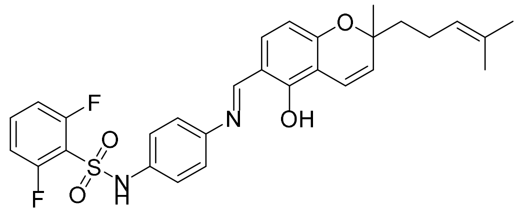

(*E*)-2,6-difluoro-*N*-(4-(((5-hydroxy-2-methyl-2-(4-methylpent-3-en-1-yl)-2*H*-chromen-6-yl) methylene)amino)phenyl)benzenesulfonamide (C47): Yield: 47.98%; yellow solid; m. p. 80−82 °C; ^{1}H NMR (500 MHz, DMSO-d_6) δ 14.21 (s, 1H, -OH), 11.02 (s, 1H, -SO_2NH-), 8.76 (s, 1H,-N=CH-), 7.75 – 7.67 (m, 1H, Ar-H), 7.35 – 7.26 (m, 5H, Ar-H), 7.20 (d, J = 8.7 Hz, 2H, Ar-H), 6.66 (d, J = 10.2 Hz, 1H, Ar-H), 6.38 (d, J = 8.5 Hz, 1H, Ar-H), 5.68 (d, J = 10.1 Hz, 1H, Ar-H), 5.12 – 5.01 (m, 1H, -CH=C(CH_3)$_2$), 2.02 (d, J = 8.0 Hz, 2H, -CH_2-), 1.72 – 1.62 (m, 2H, -CH_2-), 1.60 (s, 3H, -CH_3), 1.50 (s,

3H, -CH_3), 1.35 (s, 3H, -CH_3). ^{13}C NMR (126 MHz, DMSO-d_6) δ 162.8, 160.4 (d, J = 4.0 Hz), 158.6 – 158.3 (m), 157.7, 144.0, 136.6 (d, J = 11.0 Hz), 135.8, 134.2, 131.5, 128.1, 124.3, 122.6, 120.8, 116.4, 114.1 (d, J = 3.4 Hz), 114.0 (d, J = 3.4 Hz), 113.2, 108.8, 108.3, 79.9, 41.3, 27.0, 25.9, 22.7, 17.9. ^{19}F NMR (471 MHz, DMSO-d_6) δ -107.6 (d, J = 10.3 Hz). HRMS (ESI) m/z for $C_{29}H_{28}F_2N_2O_4S$ $[M+H]^+$ calcd 539.18121, found 539.18106.

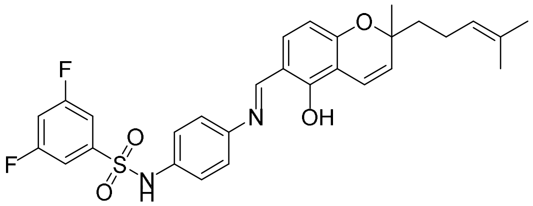

(*E*)-3,5-difluoro-*N*-(4-(((5-hydroxy-2-methyl-2-(4-methylpent-3-en-1-yl)-2*H*-chromen-6-yl) methylene)amino)phenyl)benzenesulfonamide (C48): Yield: 58.69%; yellow solid; m. p. 138−140 °C; ^{1}H NMR (500 MHz, DMSO-$d6$) δ 14.20 (s, 1H, -OH), 10.61 (s, 1H, -SO_2NH-), 8.77 (s, 1H,-N=CH-), 7.64 (m, J = 9.1, 2.4 Hz, 1H Ar-H), 7.51 – 7.44 (m, 2H Ar-H), 7.32 (dd, J = 8.7, 2.4 Hz, 3H Ar-H), 7.17 (d, J = 8.9 Hz, 2H Ar-H), 6.66 (d, J = 10.1 Hz, 1H Ar-H), 6.38 (d, J = 8.5 Hz, 1H Ar-H), 5.67 (d, J = 10.1 Hz, 1H Ar-H), 5.09 – 5.03 (m, 1H, -CH=C(CH_3)$_2$), 2.02 (q, J = 7.9 Hz, 2H, -CH_2-), 1.65 (m, J = 10.4, 8.2 Hz, 2H, -CH_2-), 1.59 (s, 3H, -CH_3), 1.50 (s, 3H, -CH_3), 1.35 (s, 3H). ^{13}C NMR (126 MHz, DMSO-$d6$) δ 163.6 (d, J = 12.5 Hz), 162.9, 161.6 (d, J = 12.3 Hz), 158.5, 157.7, 144.4, 143.1 (t, J = 8.3 Hz), 135.7, 134.2, 131.5, 128.0, 124.3, 122.6, 122.3, 116.4, 113.2, 111.2 – 110.8 (m), 108.8, 108.3, 79.9, 41.3, 27.0, 25.9, 22.7, 17.9. ^{19}F NMR (471 MHz, DMSO-$d6$) δ -105.8 – -105.9 (m). HRMS (ESI) m/z for $C_{29}H_{28}F_2N_2O_4S$ $[M+H]^+$ calcd 539.18146, found 539.18106.

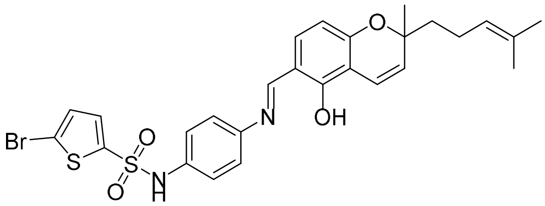

(*E*)-5-bromo-*N*-(4-(((5-hydroxy-2-methyl-2-(4-methylpent-3-en-1-yl)-2*H*-chromen-6-yl)methylene) amino)phenyl)thiophene-2-sulfonamide (C49): Yield: 60.74%; yellow solid; m. p. 75−77 °C; ^{1}H NMR (500 MHz, DMSO-d_6) δ 14.20 (s, 1H, -OH), 10.61 (s, 1H, -SO_2NH-), 8.77 (s, 1H, -N=CH-), 7.64 (m, J = 9.1, 2.4 Hz, 1H, Ar-H), 7.51 – 7.44 (m, 2H, Ar-H), 7.32 (dd, J = 8.7, 2.4 Hz, 3H, Ar-H), 7.17 (d, J = 8.9 Hz, 2H, Ar-H), 6.66 (d, J = 10.1 Hz, 1H, Ar-H), 6.38 (d, J = 8.5 Hz, 1H, Ar-H), 5.67 (d, J = 10.1 Hz, 1H, Ar-H), 5.09 – 5.03 (m, 1H, -CH=C(CH_3)$_2$), 2.02 (m, J = 7.9 Hz, 2H, -CH_2-), 1.65 (m, J = 10.4, 8.2 Hz, 2H, -CH_2-), 1.59 (s, 3H, -CH_3), 1.50 (s, 3H, -CH_3), 1.35 (s, 3H, -CH_3). ^{13}C NMR (126 MHz, DMSO-d_6) δ 163.6 (d, J = 12.5 Hz), 162.94, 161.6 (d, J = 12.3 Hz), 158.5, 157.7, 144.4, 143.1 (t, J = 8.3 Hz), 135.7, 134.2, 131.5, 128.0, 124.3, 122.6, 122.3, 116.4, 113.2, 111.2 – 110.8 (m), 108.8, 108.3, 79.9, 41.3, 27.0, 25.9, 22.7, 17.9. ^{19}F NMR (471 MHz, DMSO-d_6) δ -105.8 – -105.9 (m). HRMS (ESI) m/z for $C_{27}H_{27}BrN_2O_4S_2$ $[M+H]^+$ calcd 587.06684, found 587.06714.

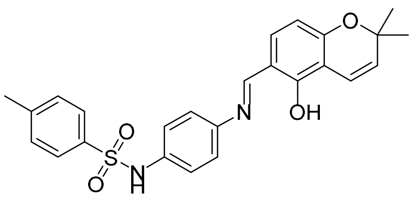

(*E*)-*N*-(4-(((5-hydroxy-2,2-dimethyl-2*H*-chromen-6-yl)methylene)amino)phenyl)-4-methylbenzenesulfonamide (C50): Yield: 70.13%; yellow solid; m. p. 127−129 °C; ^{1}H NMR (500 MHz, DMSO-*d6*) δ 14.24 (s, 1H, -OH), 10.32 (s, 1H, -SO_2NH-), 8.75 (s, 1H, -N=CH-), 7.65 (d, J = 8.3 Hz, 2H, Ar-H), 7.33 (dd, J = 13.8, 8.4 Hz, 3H, Ar-H), 7.28 (d, J = 8.9 Hz, 2H, Ar-H), 7.14 (d, J = 8.9 Hz, 2H, Ar-H), 6.62 (d, J = 10.0 Hz, 1H, Ar-H), 6.37 (d, J = 8.5 Hz, 1H, Ar-H), 5.71 (d, J = 10.0 Hz, 1H, Ar-H), 2.32 (s, 3H, -CH_3), 1.39 (s, 6H, -CH_3). ^{13}C NMR (126 MHz, DMSO-*d6*) δ 162.5, 158.6, 157.3, 143.8, 143.6, 137.1, 136.8, 134.1, 130.2, 129.2, 127.2, 122.4, 121.4, 116.0, 113.3, 109.0, 108.4, 77.6, 28.3, 21.4. HRMS (ESI) m/z for $C_{25}H_{24}N_2O_4S$ $[M+H]^+$ calcd 449.15295, found 449.15265.

B) NMR and HRMS spectra of products.

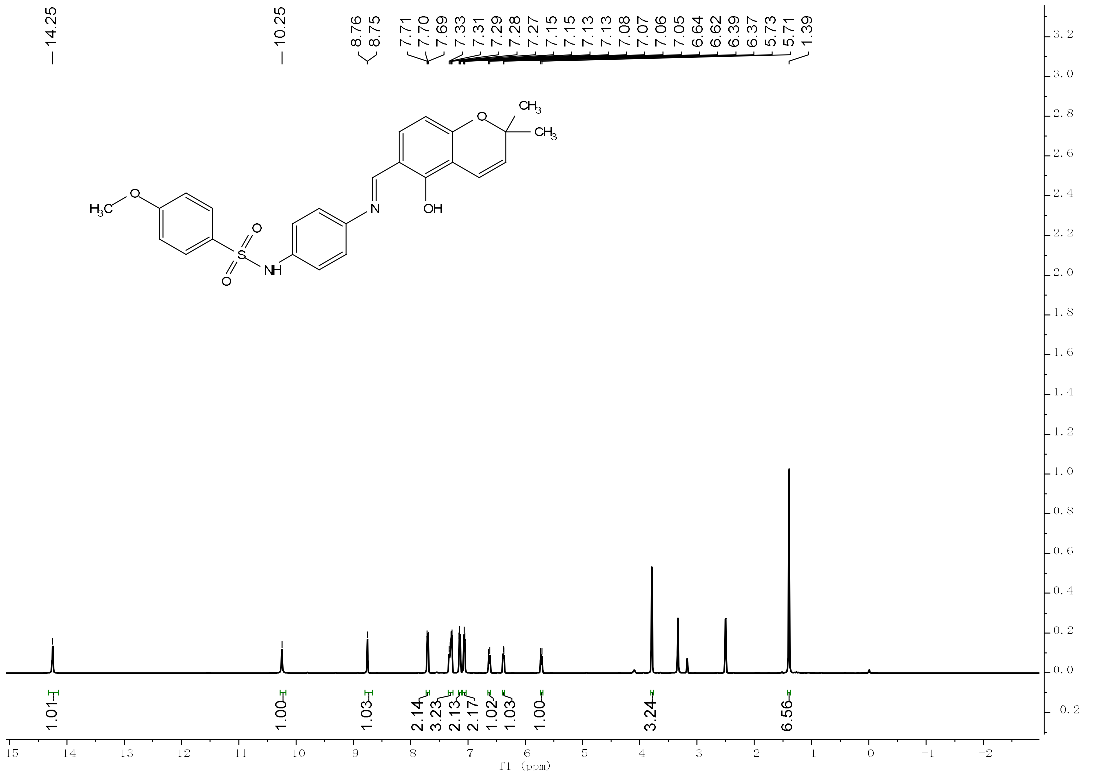

^{1}H NMR (500 MHz, DMSO-d_6) spectrum of compound **C1**.

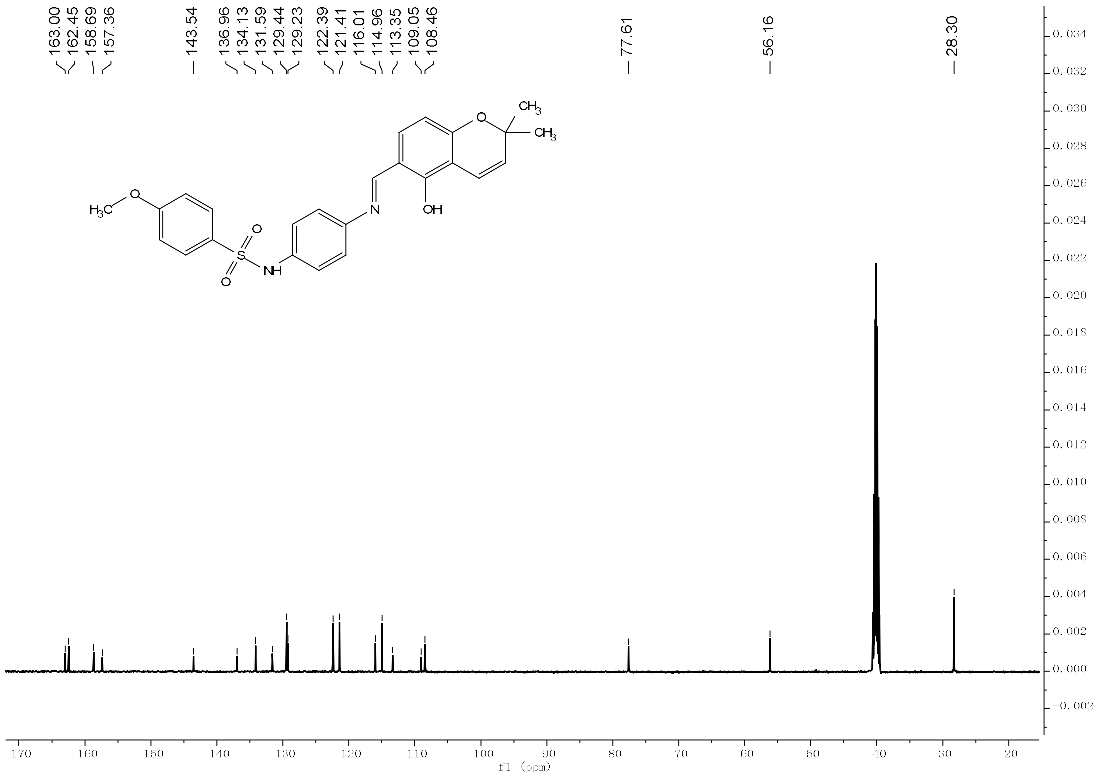

^{13}C NMR (126 MHz, DMSO-d_6) spectrum of compound **C1**.

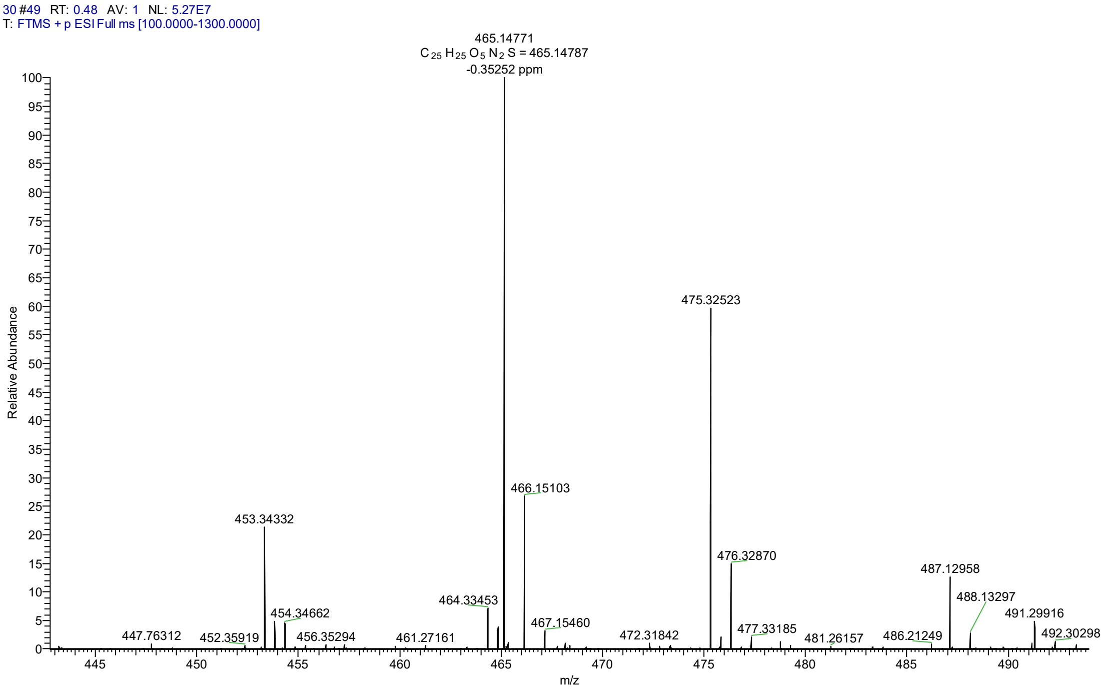

HRMS of compound **C1**.

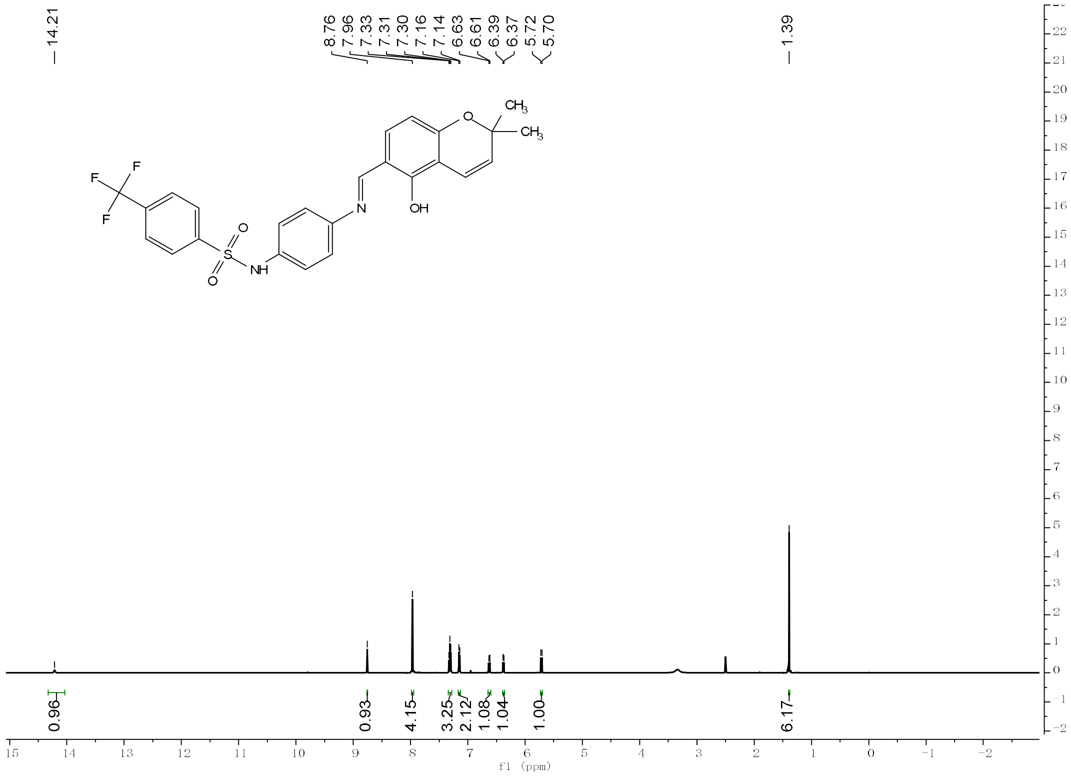

^{1}H NMR (500 MHz, DMSO-d_6) spectrum of compound **C2**.

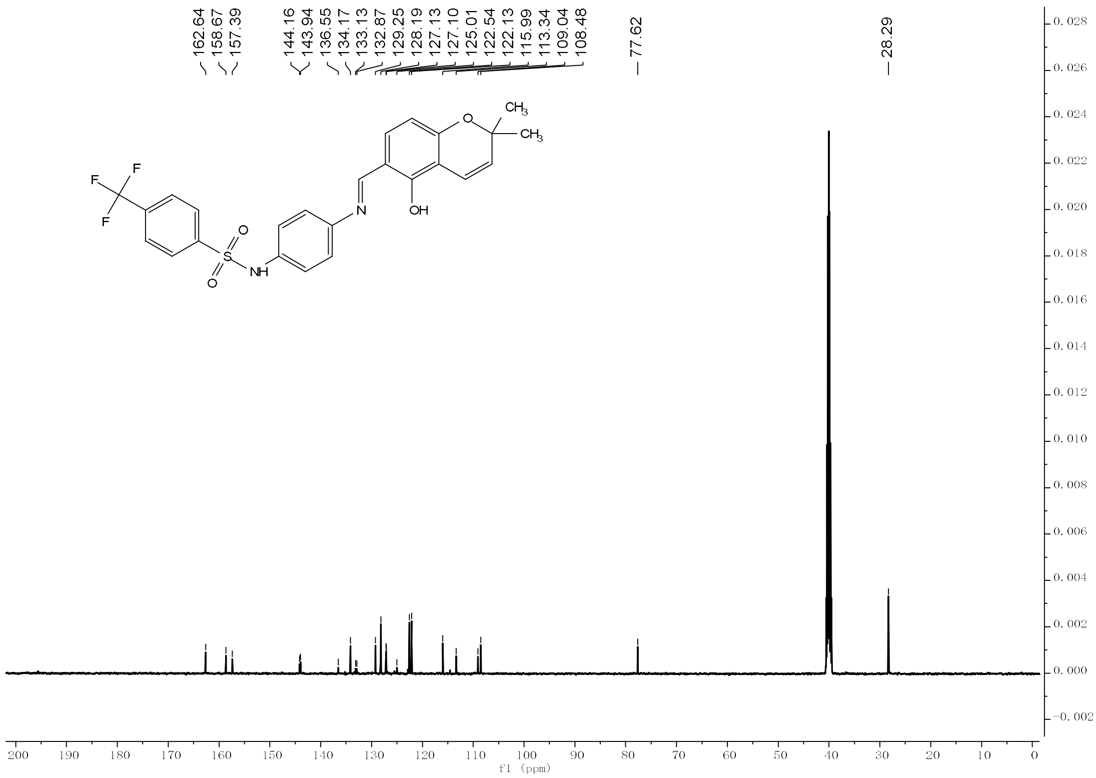

^{13}C NMR (126 MHz, DMSO-d_6) spectrum of compound **C2**.

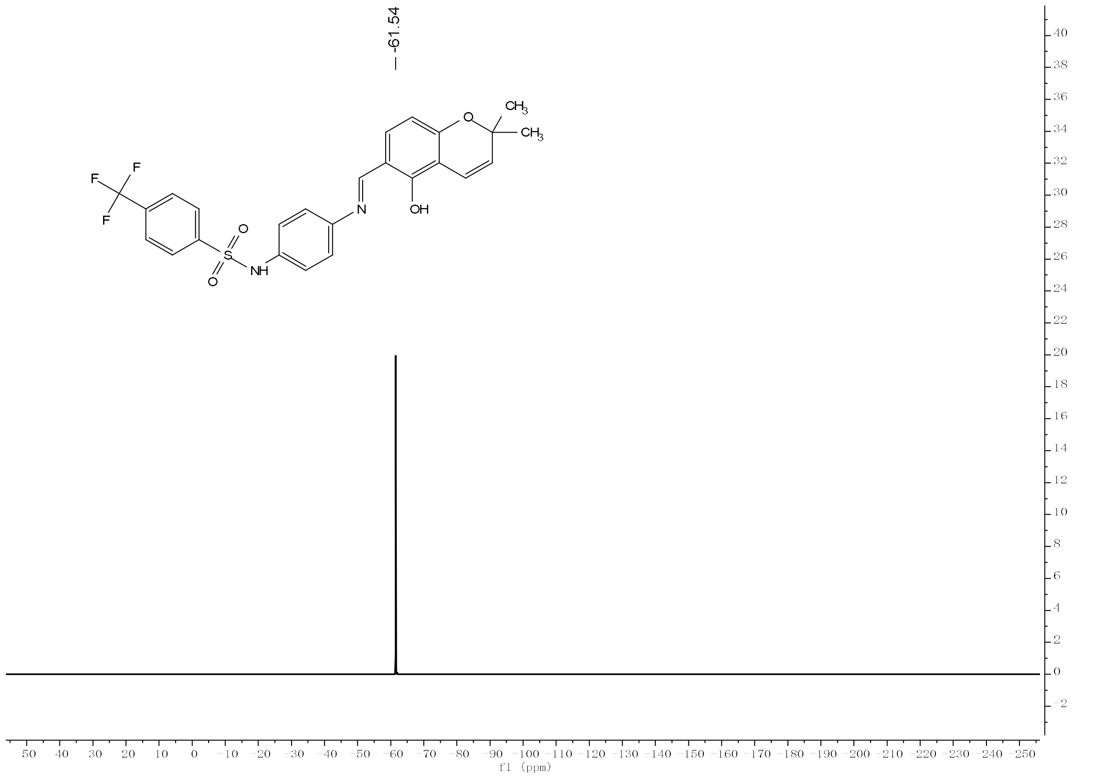

^{19}F NMR (471 MHz, DMSO-d_6) spectrum of compound **C2**.

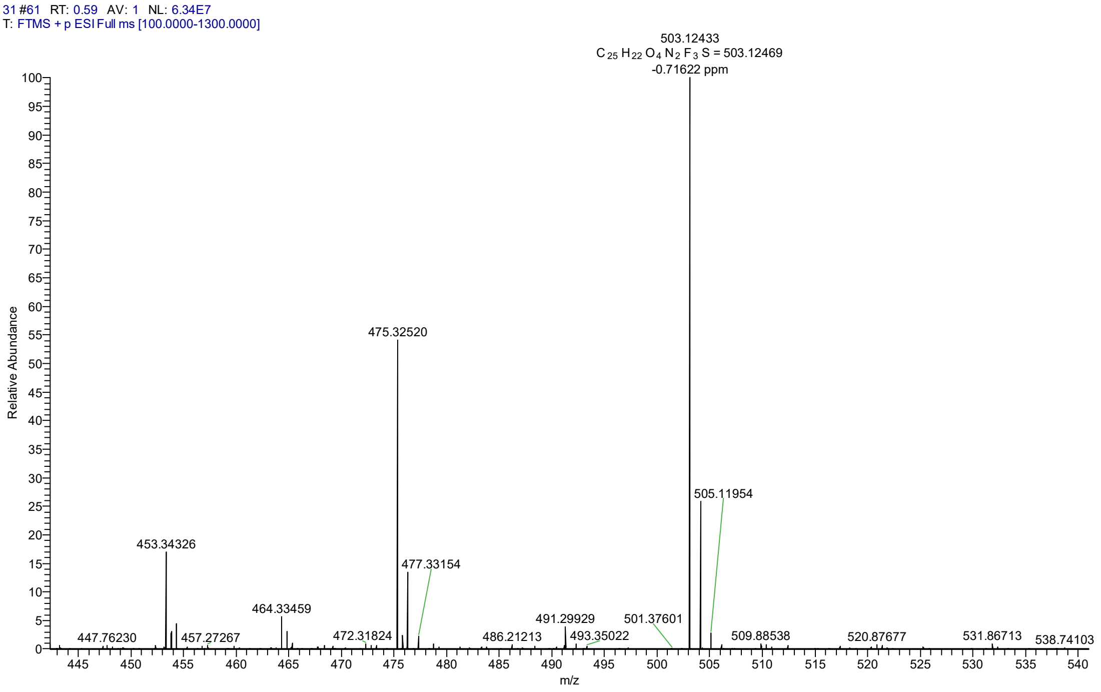

HRMS of compound **C2**.

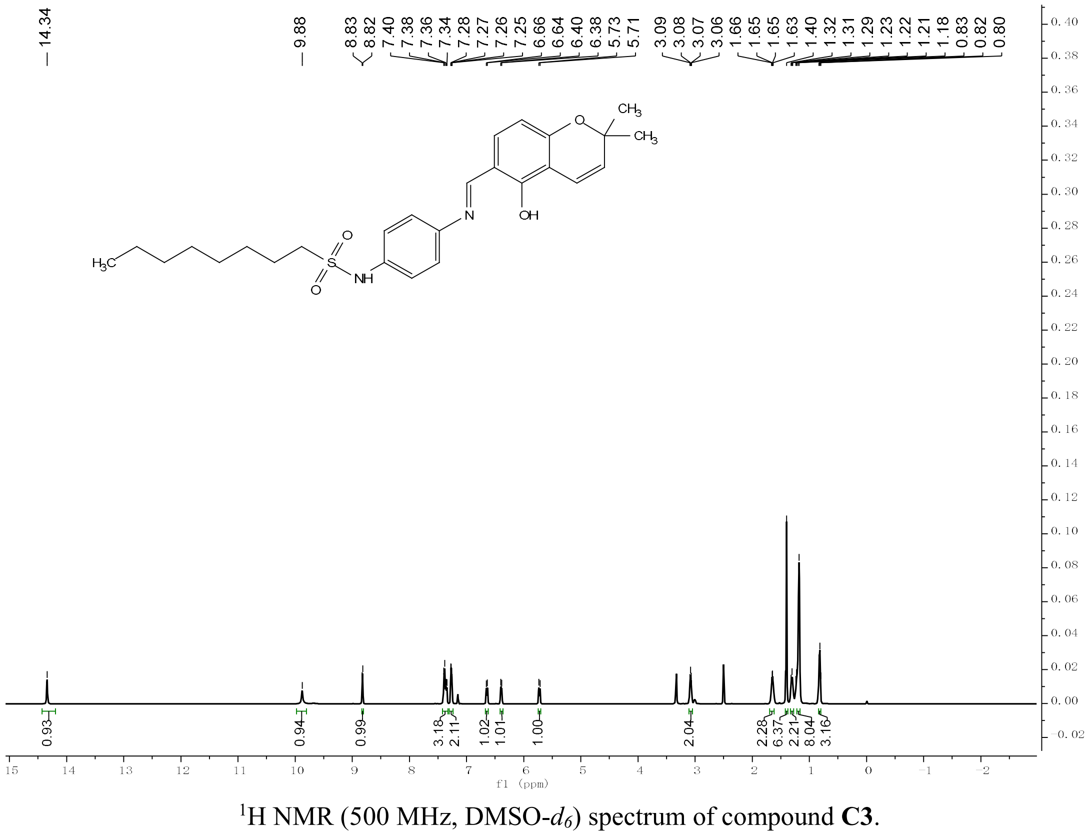

^{1}H NMR (500 MHz, DMSO-d_6) spectrum of compound **C3**.

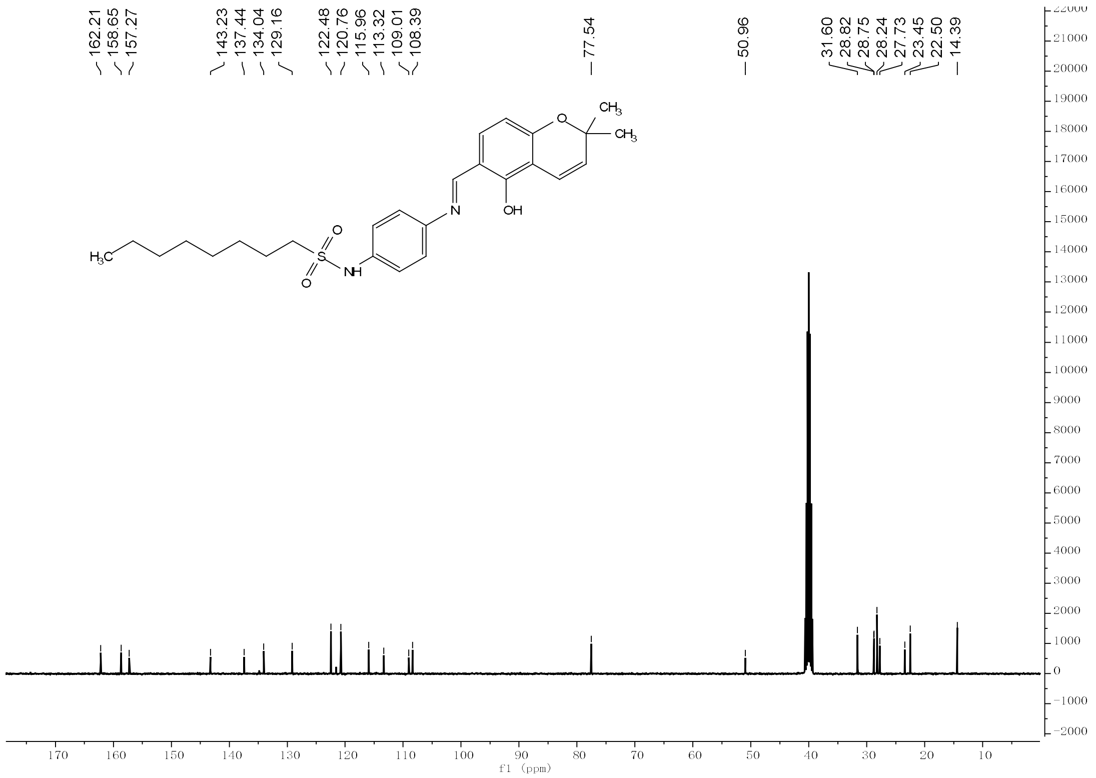

^{13}C NMR (101 MHz, DMSO-d_6) spectrum of compound **C3**.

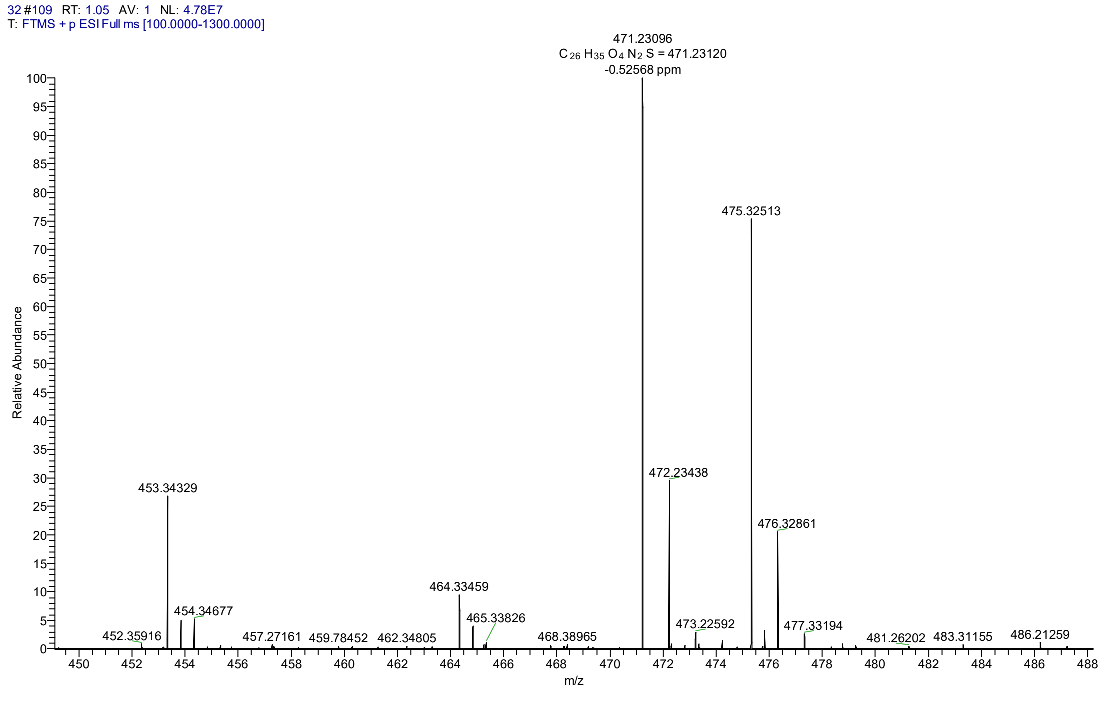

HRMS of compound **C3**.

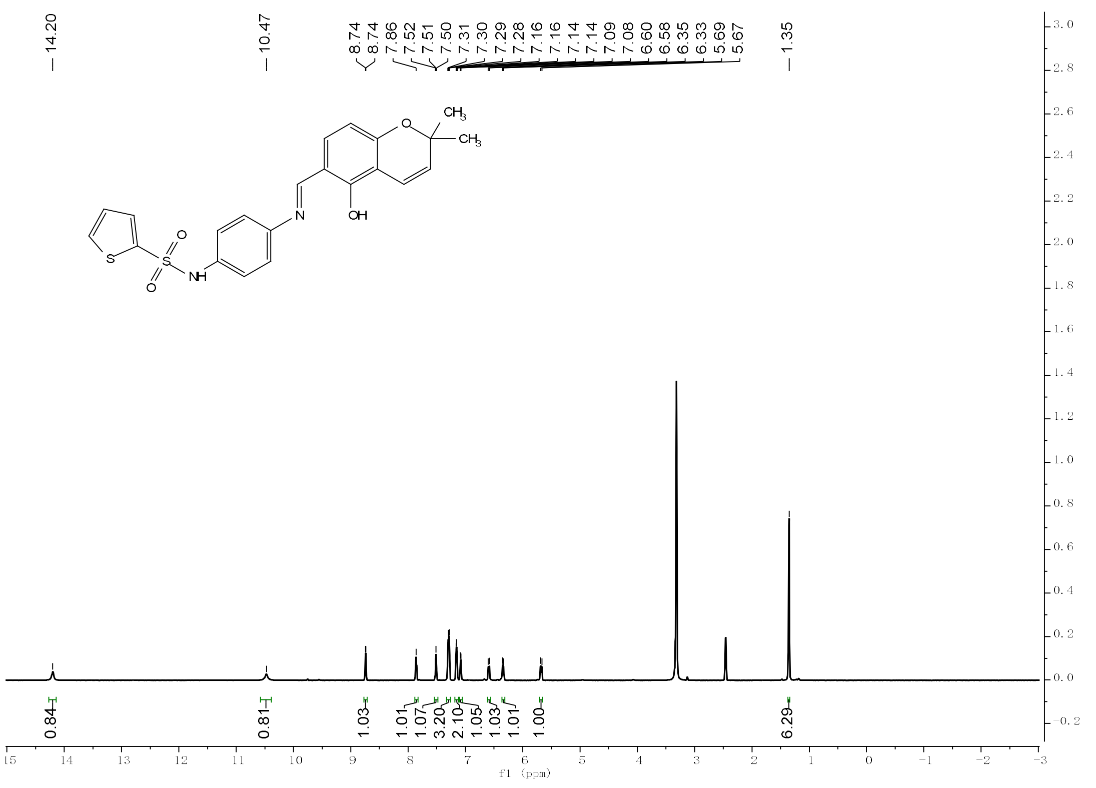

^{1}H NMR (500 MHz, DMSO-d_6) spectrum of compound **C4**.

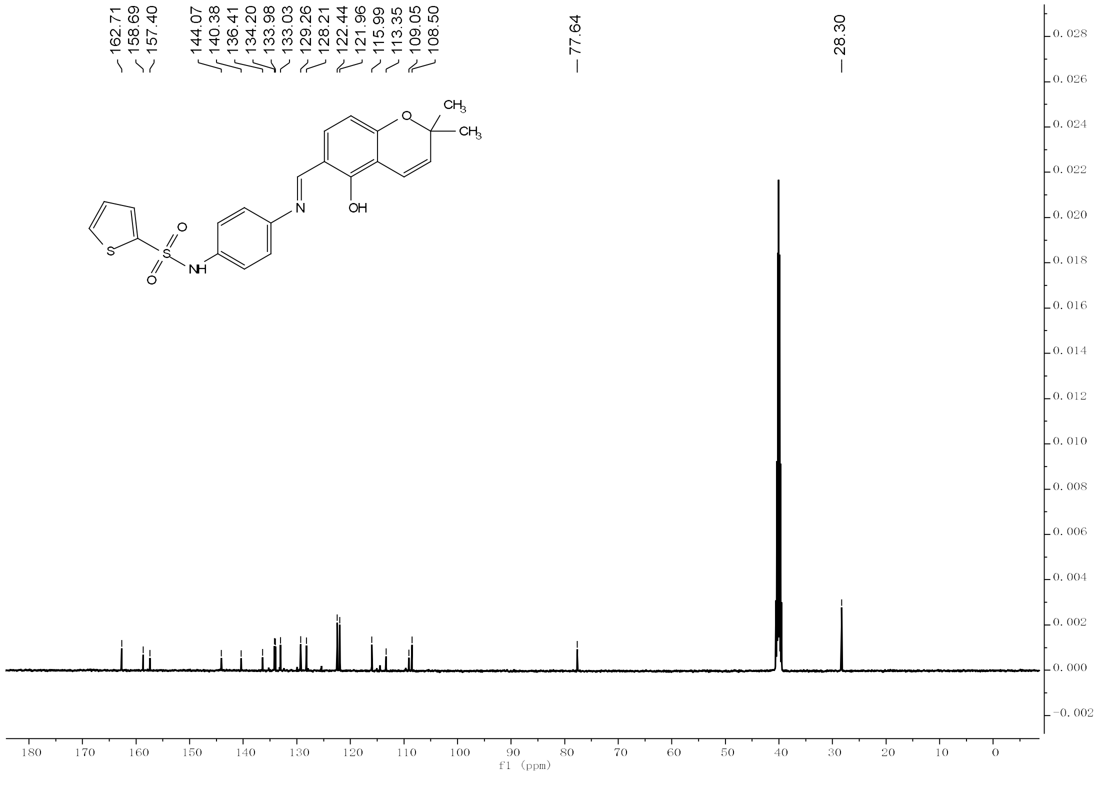

^{13}C NMR (126 MHz, DMSO-d_6) spectrum of compound **C4**.

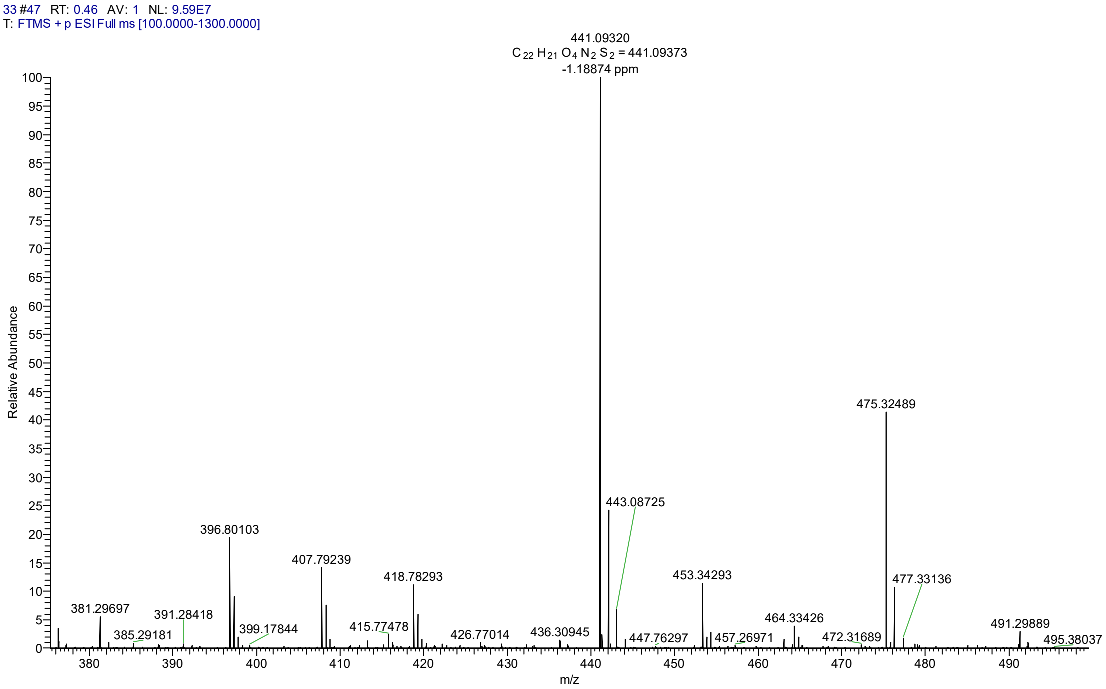

HRMS of compound **C4**.

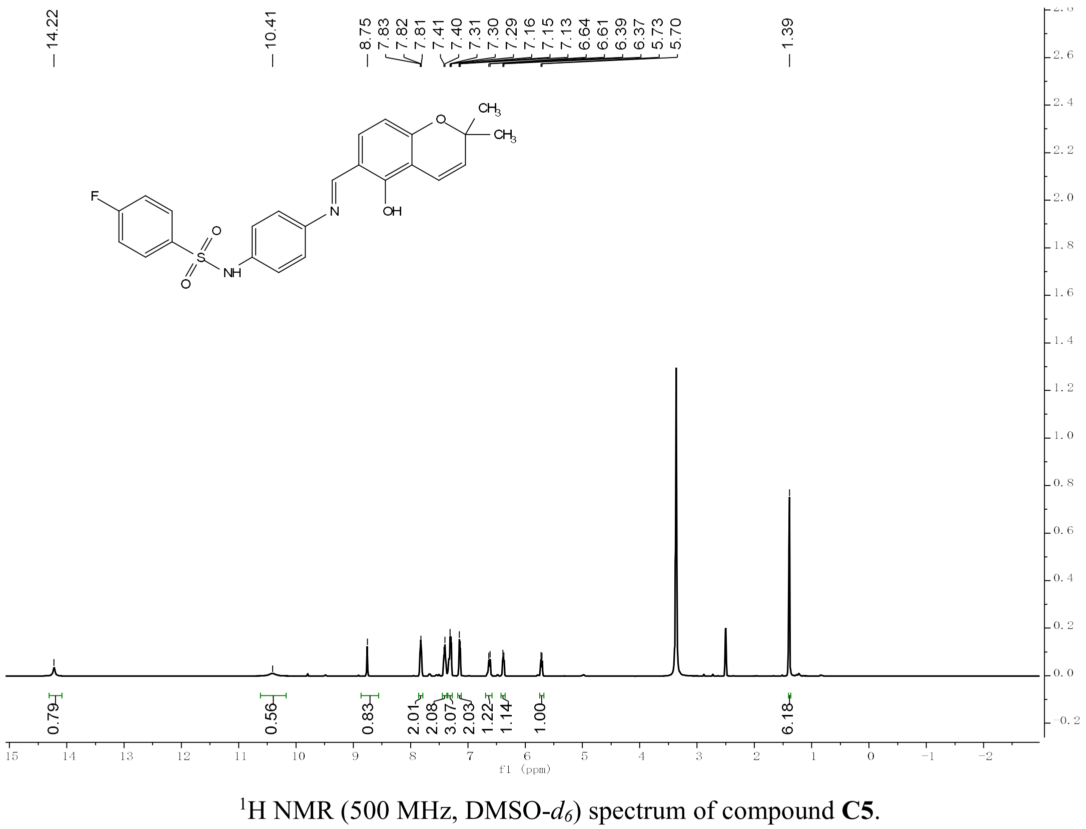

¹H NMR (500 MHz, DMSO-d_6) spectrum of compound **C5**.

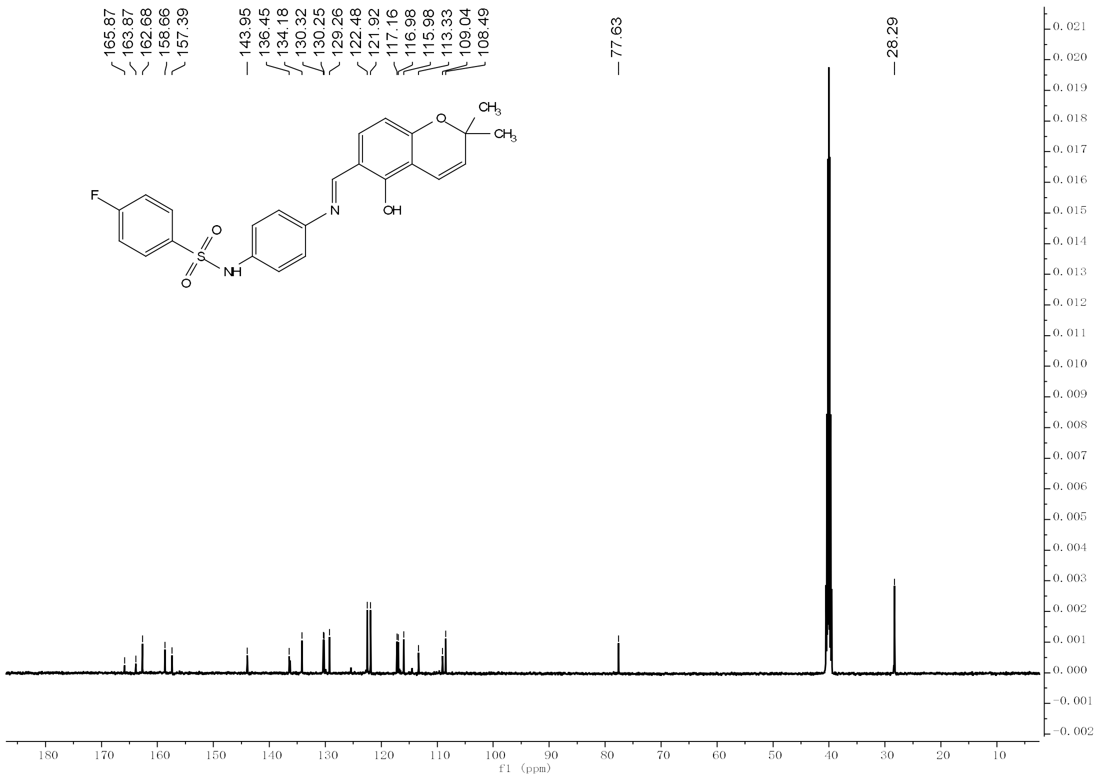

^{13}C NMR (126 MHz, DMSO-d_6) spectrum of compound **C5**.

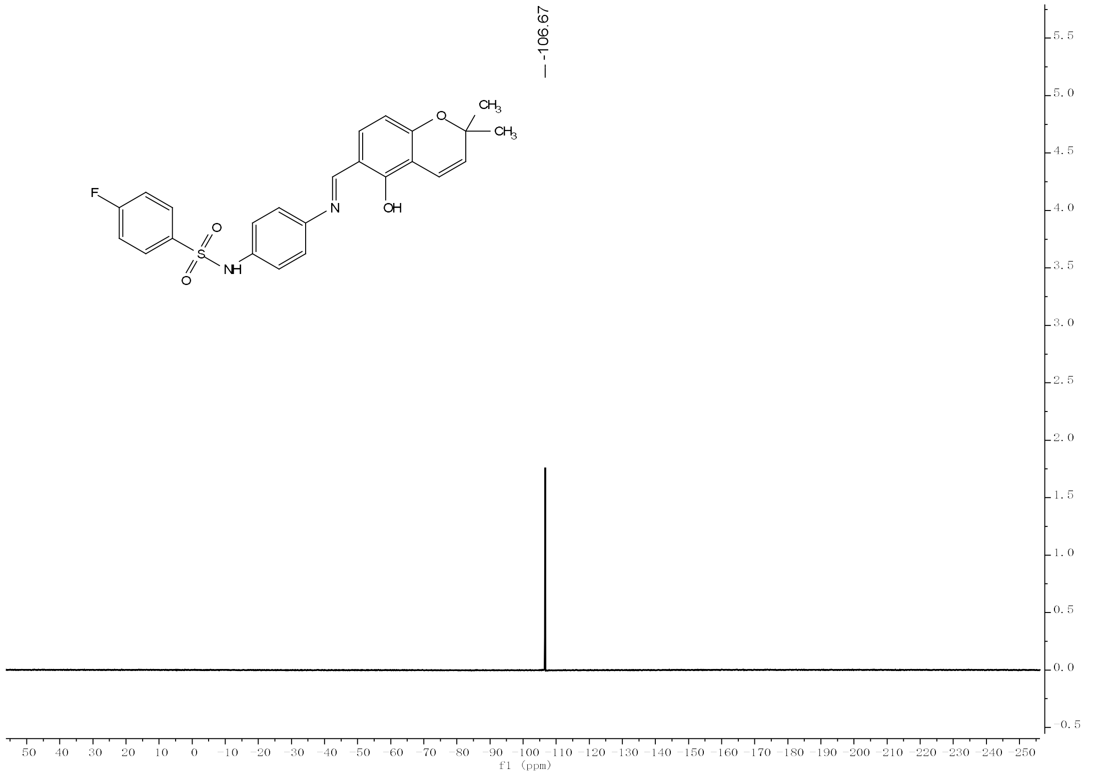

^{19}F NMR (471 MHz, DMSO-d_6) spectrum of compound **C5**.

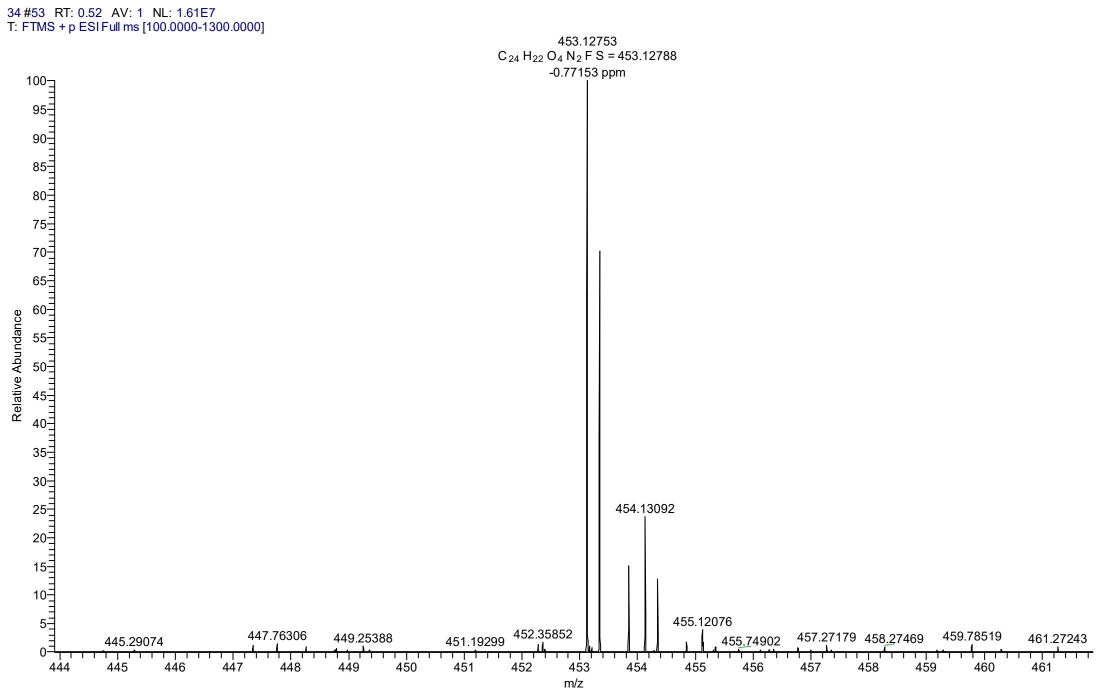

HRMS of compound **C5**.

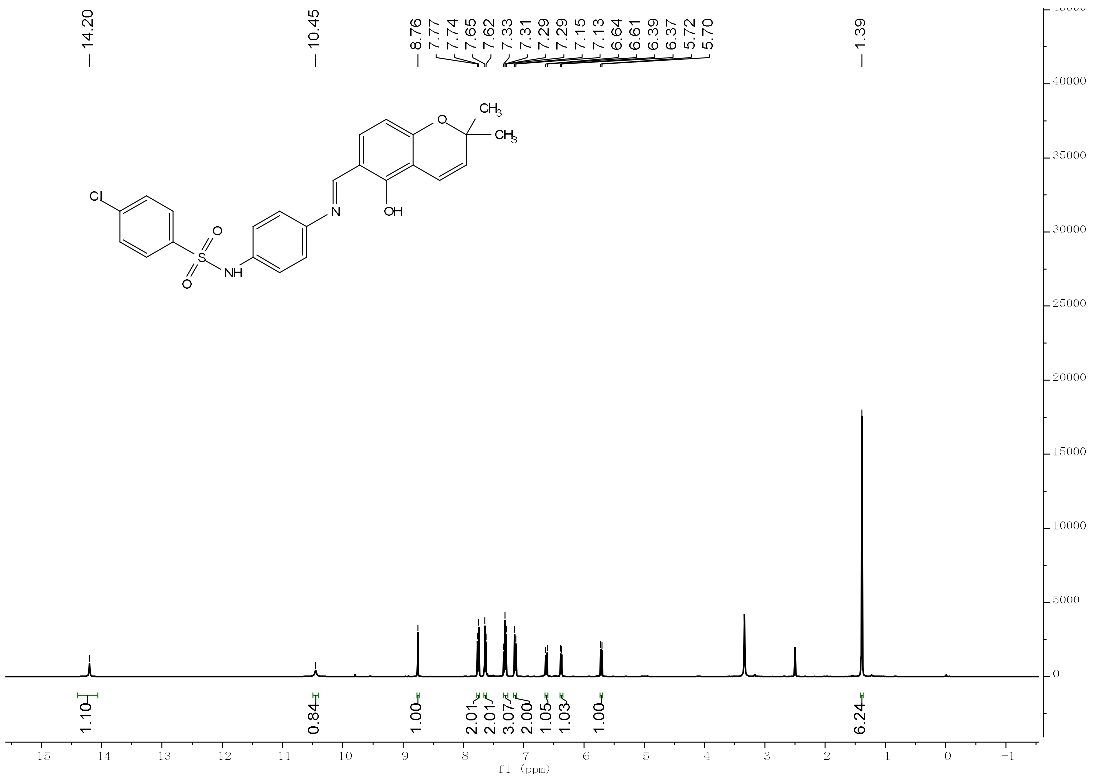

^{1}H NMR (400 MHz, DMSO-d_6) spectrum of compound C**6**.

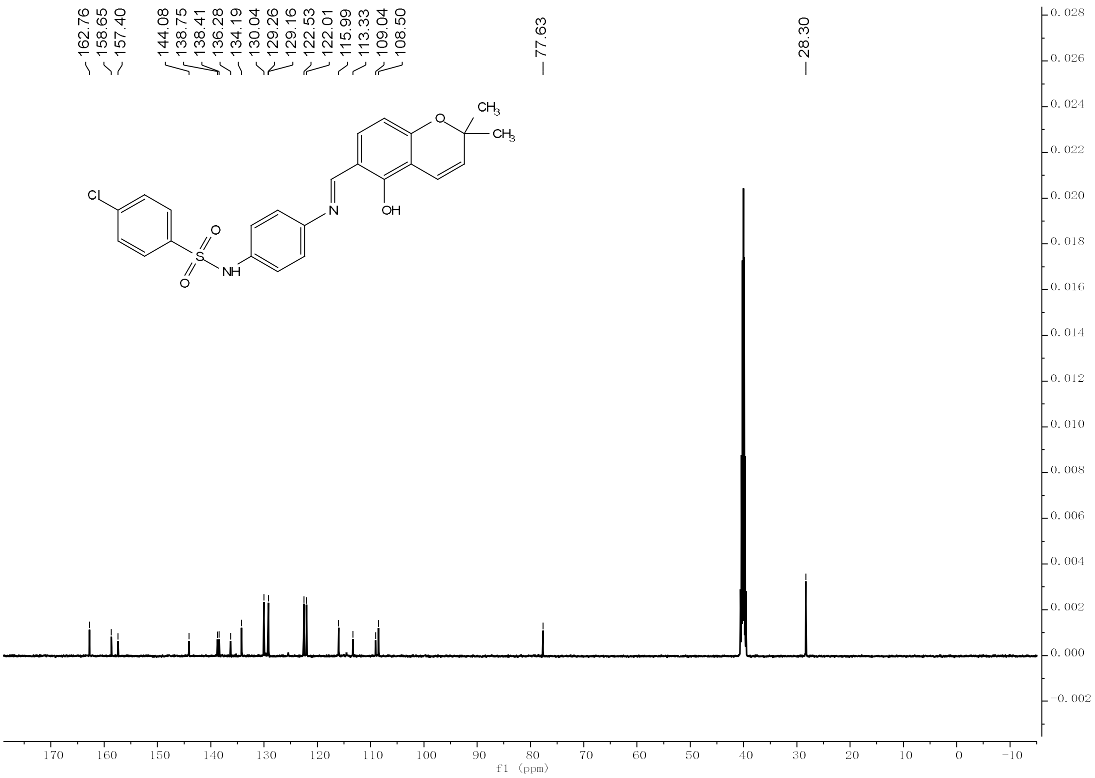

^{13}C NMR (126 MHz, DMSO-d_6) spectrum of compound **C6**.

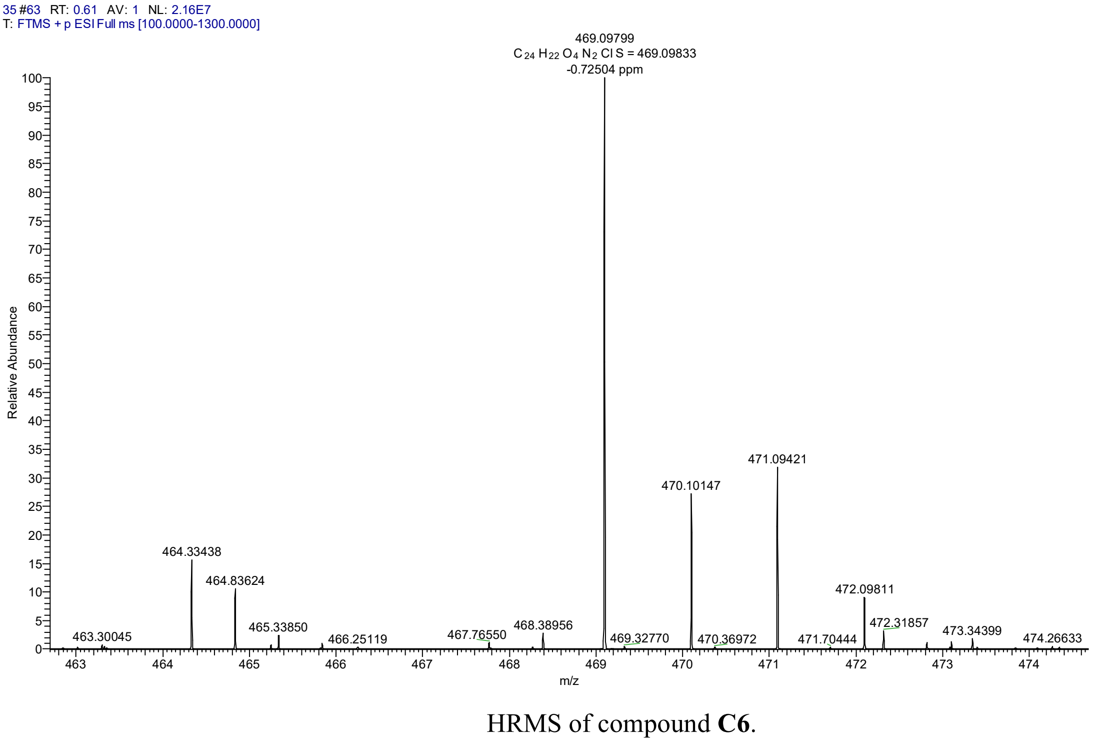

HRMS of compound **C6**.

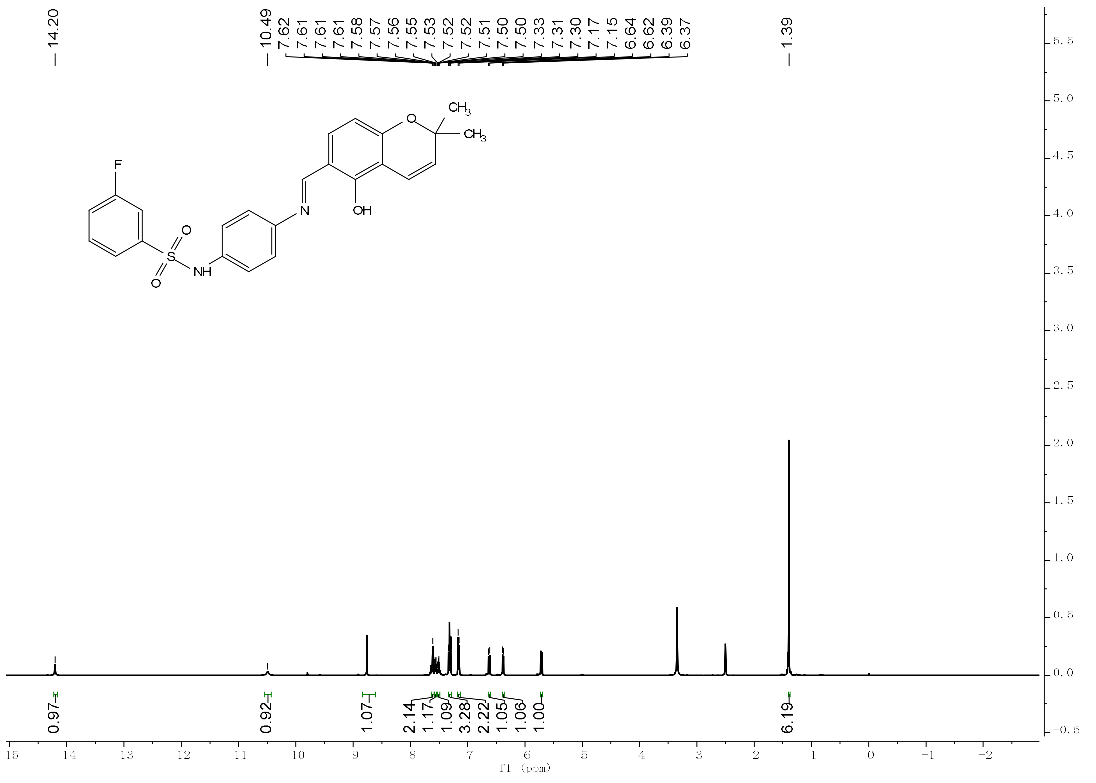

^{1}H NMR (500 MHz, DMSO-d_6) spectrum of compound **C7**.

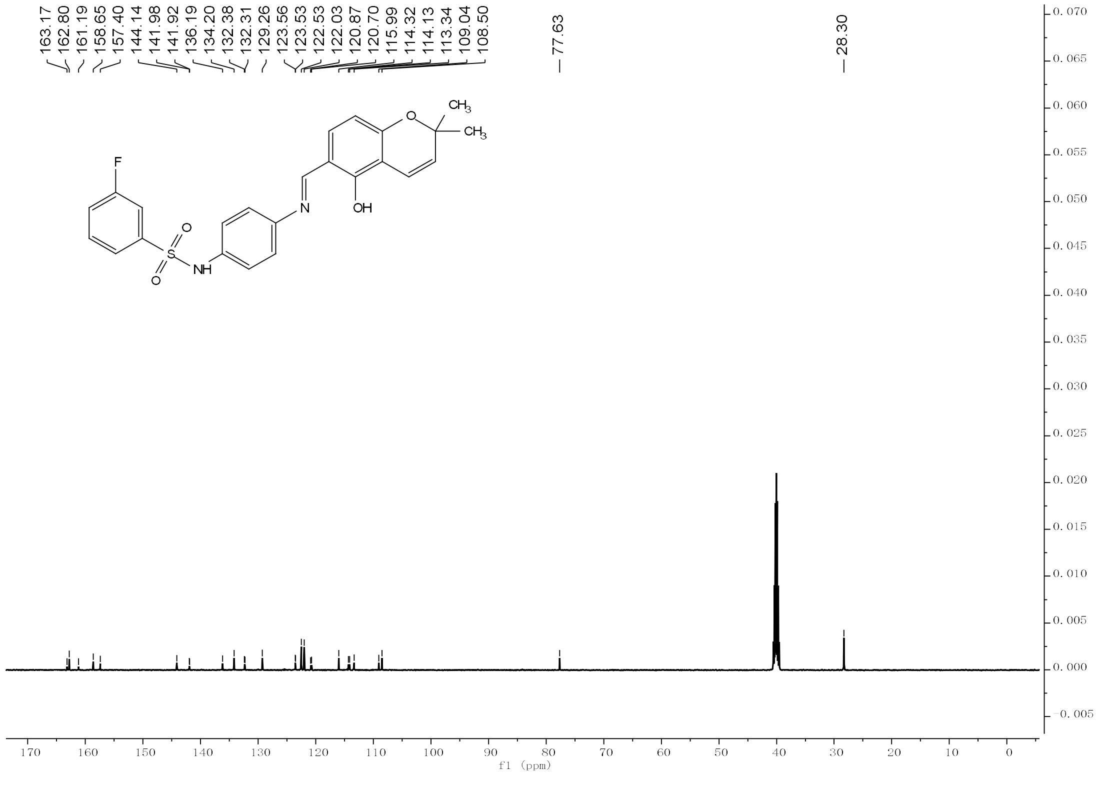

^{13}C NMR (126 MHz, DMSO-d_6) spectrum of compound **C7**.

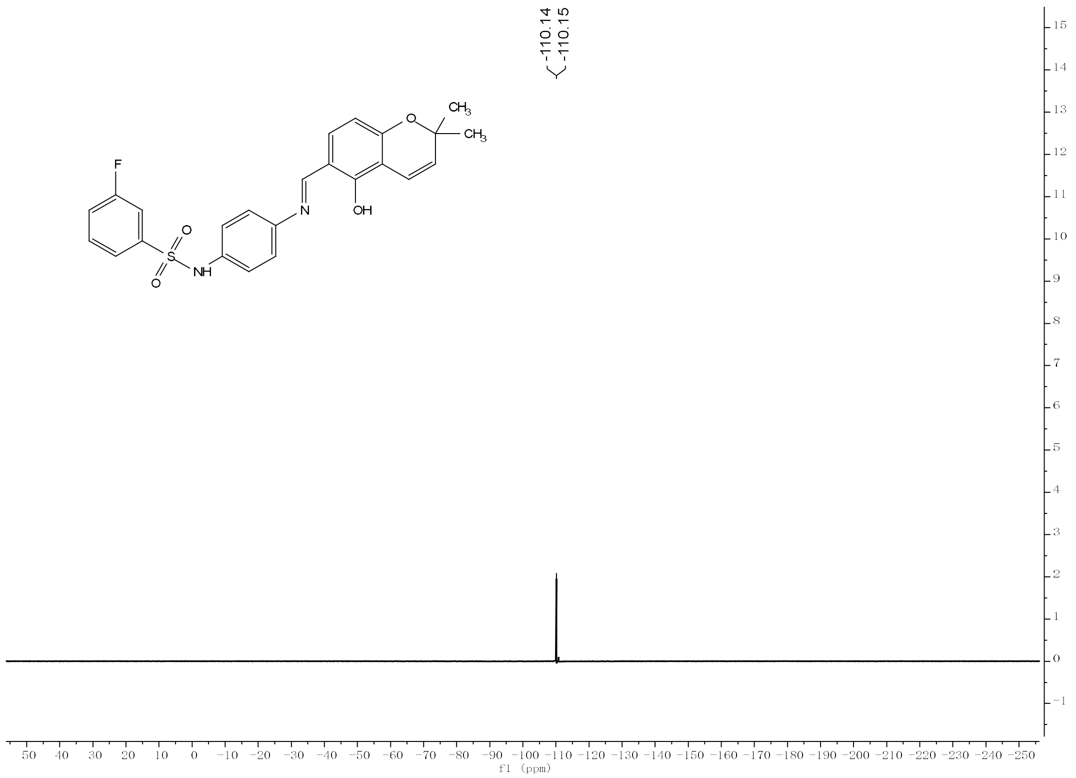

^{19}F NMR (471 MHz, DMSO-d_6) spectrum of compound **C7**.

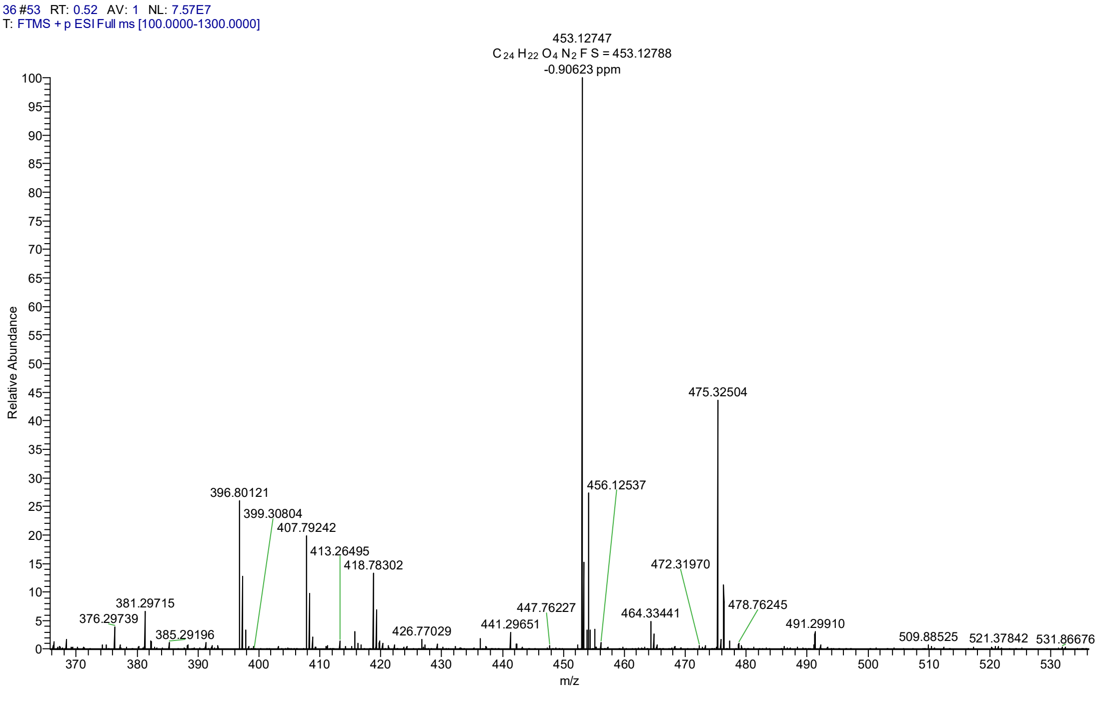

HRMS of compound **C7**.

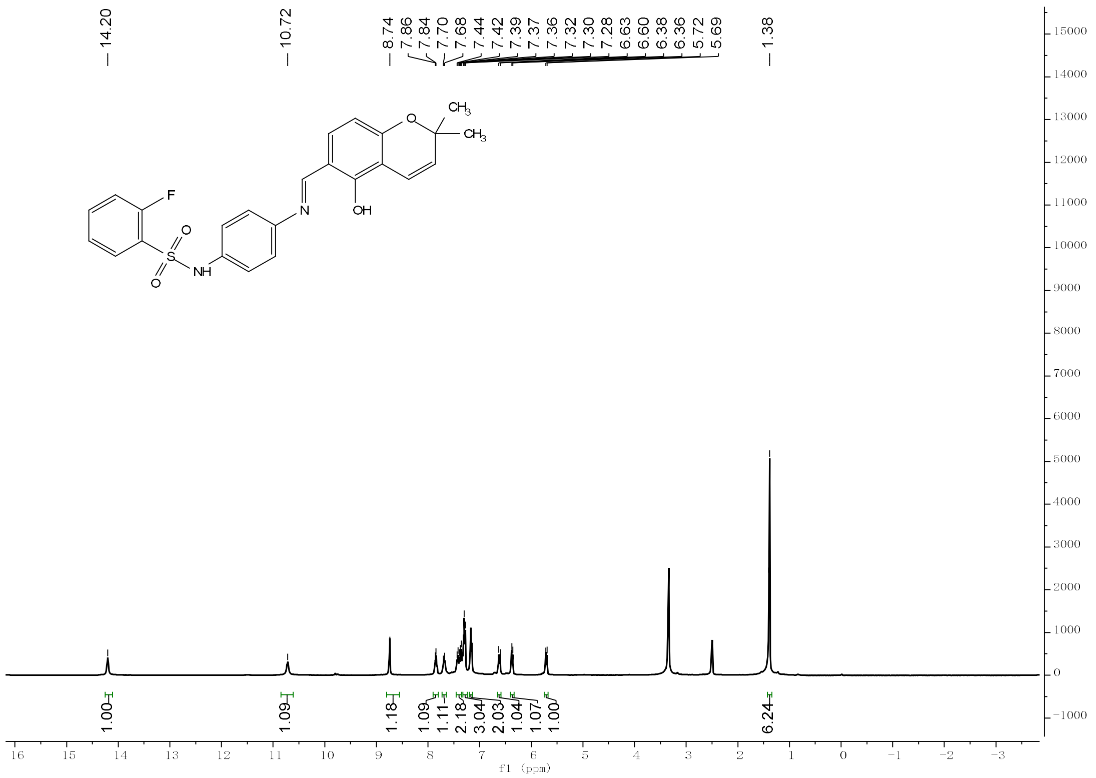

^{1}H NMR (400 MHz, DMSO-d_6) spectrum of compound **C8**.

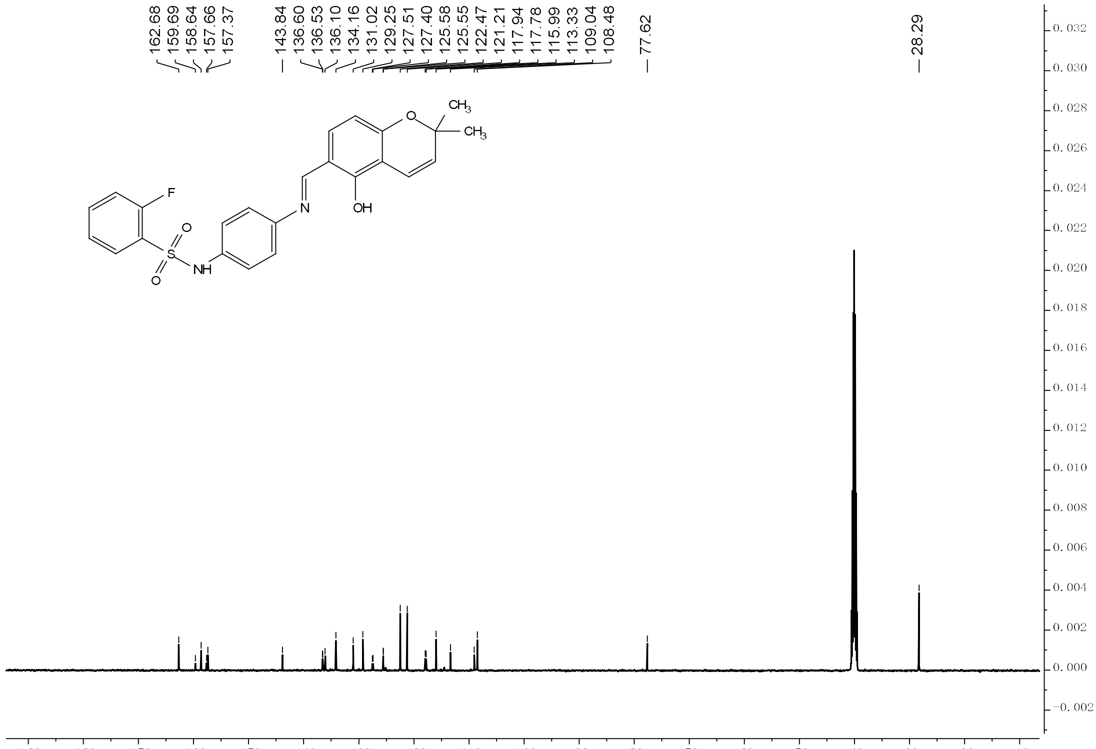

^{13}C NMR (126 MHz, DMSO-d_6) spectrum of compound **C8**.

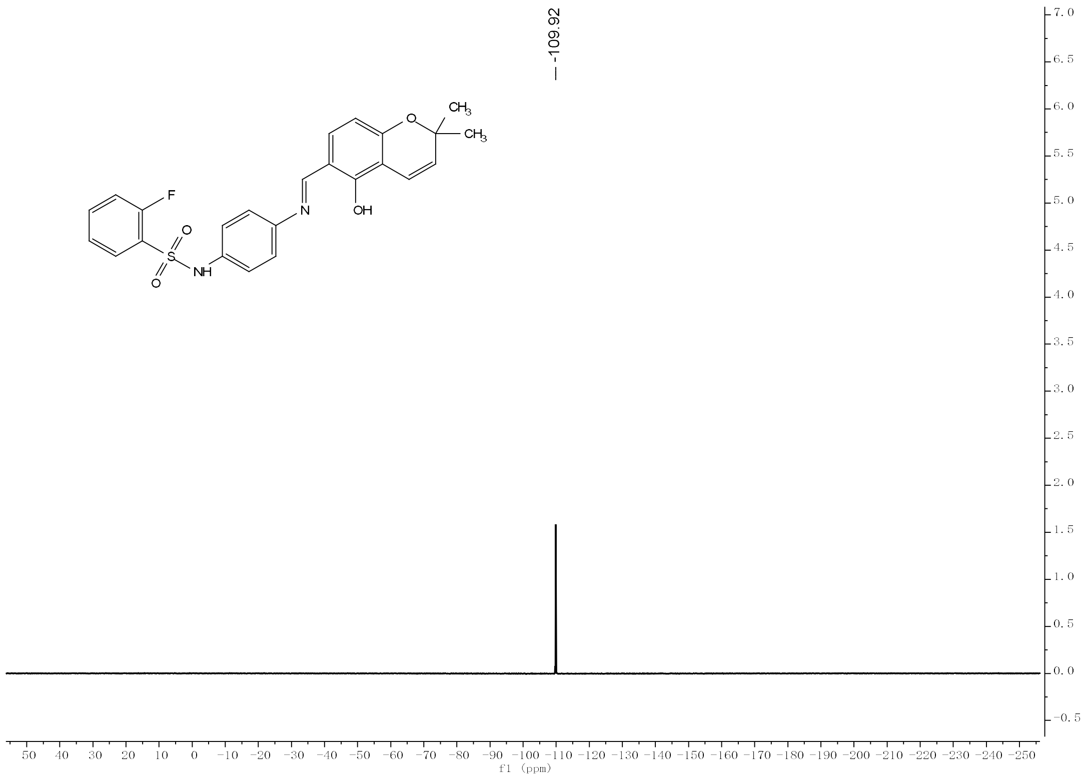

^{19}F NMR (471 MHz, DMSO-d_6) spectrum of compound **C8**.

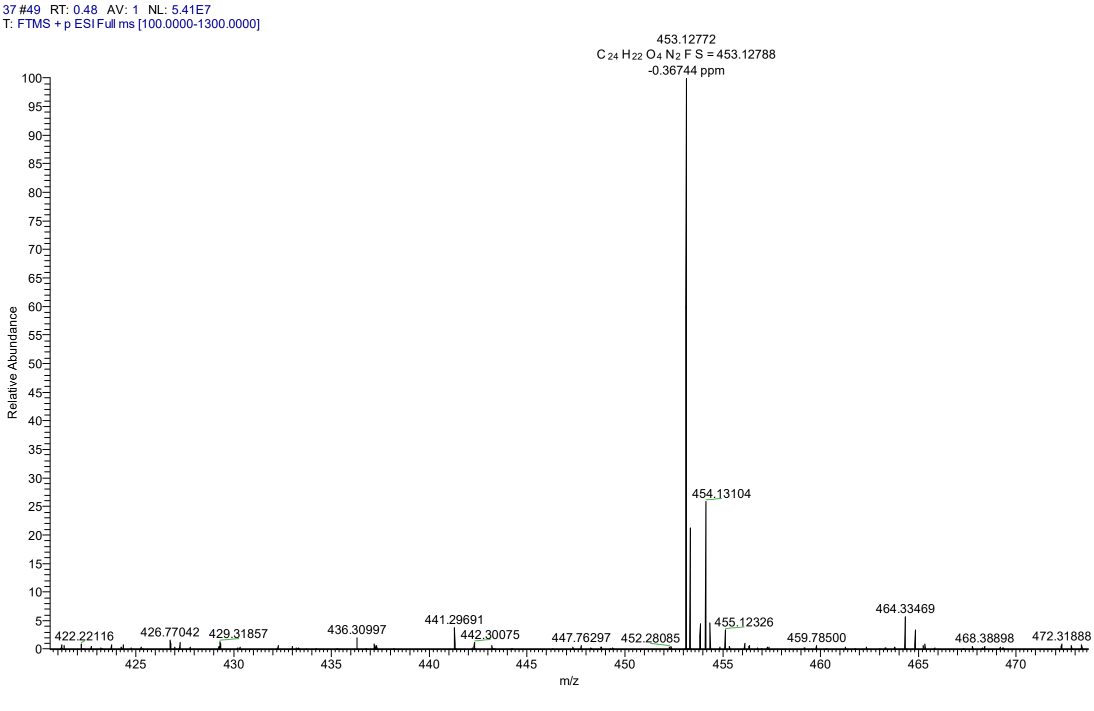

HRMS of compound **C8**.

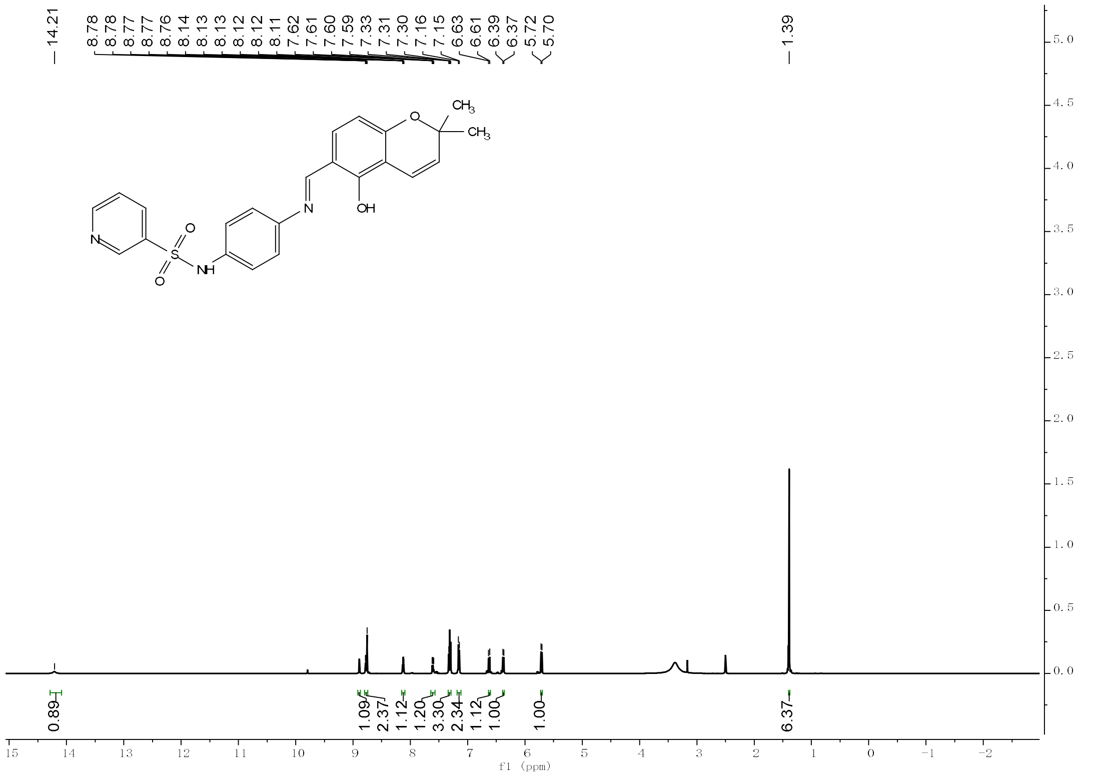

^{1}H NMR (500 MHz, DMSO-d_6) spectrum of compound **C9**.

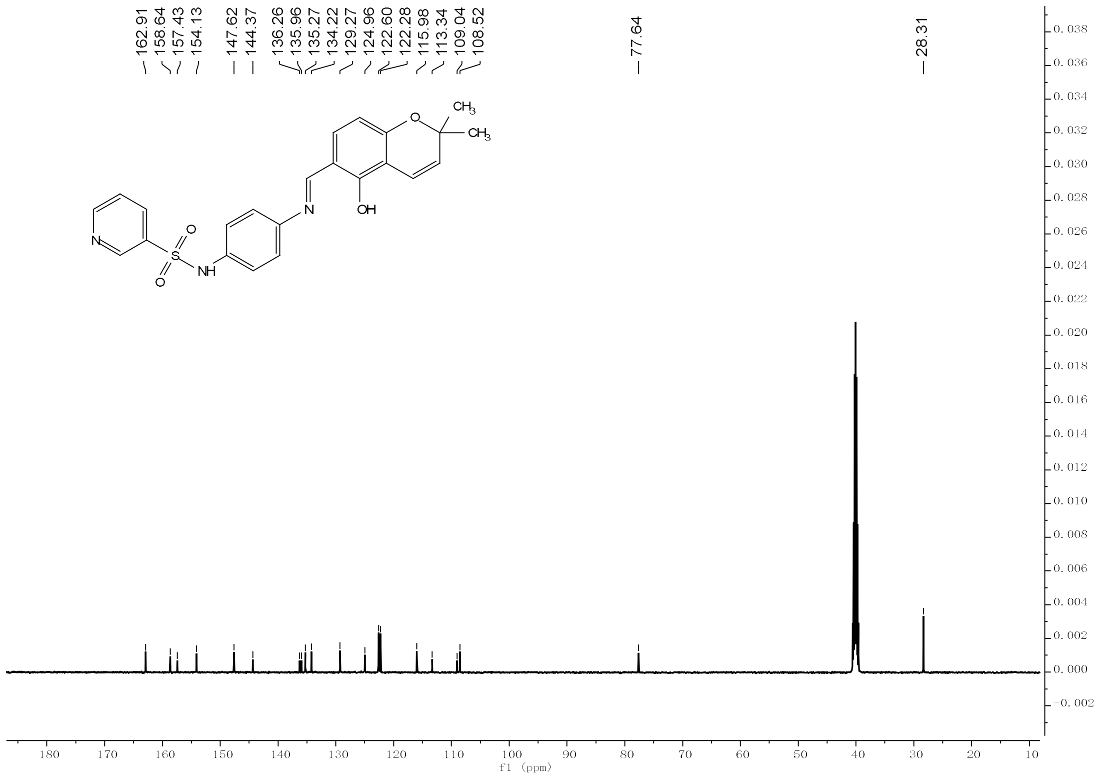

^{13}C NMR (126 MHz, DMSO-d_6) spectrum of compound **C9**.

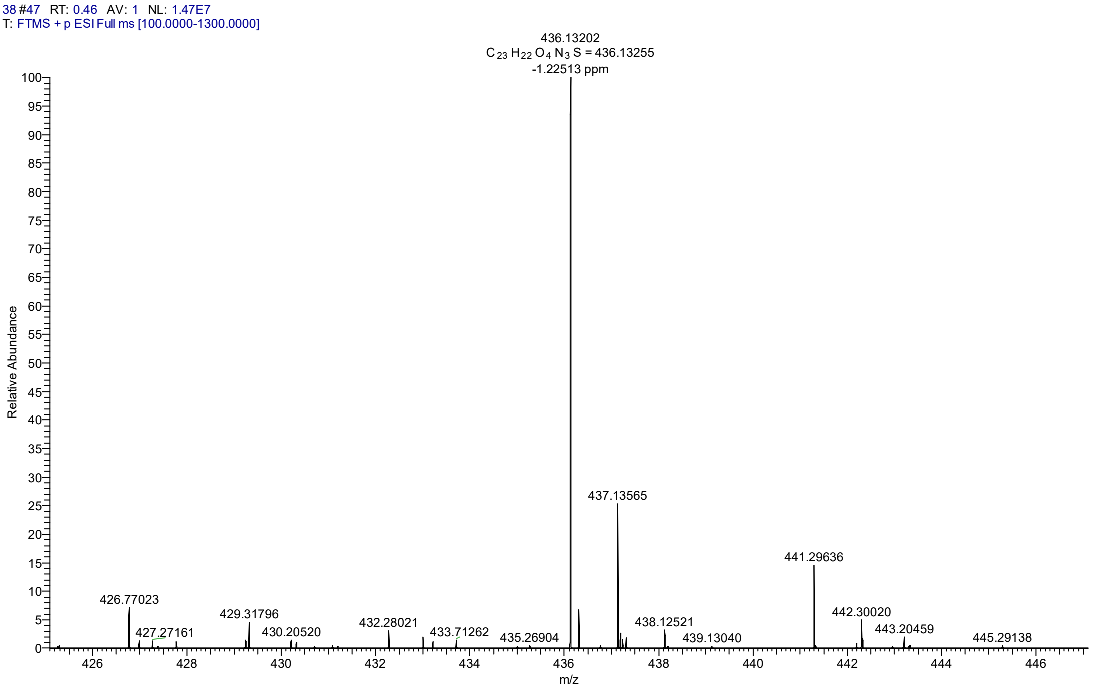

HRMS of compound **C9.**

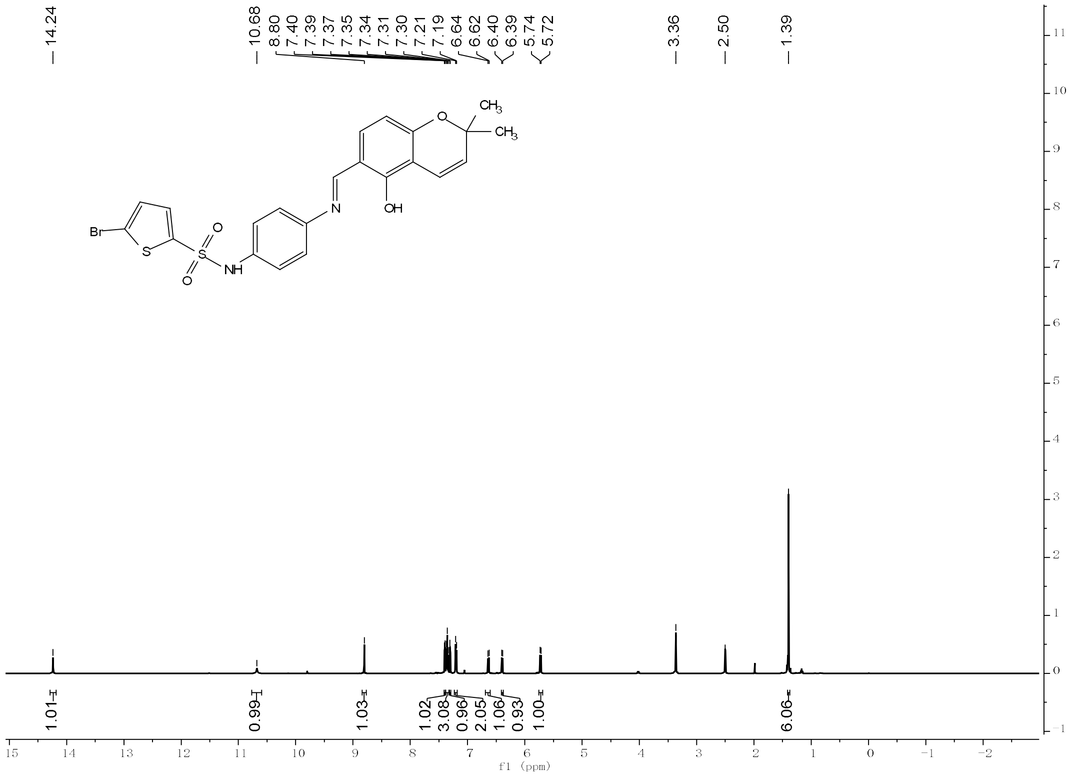

^{1}H NMR (500 MHz, DMSO-d_6) spectrum of compound **C10**.

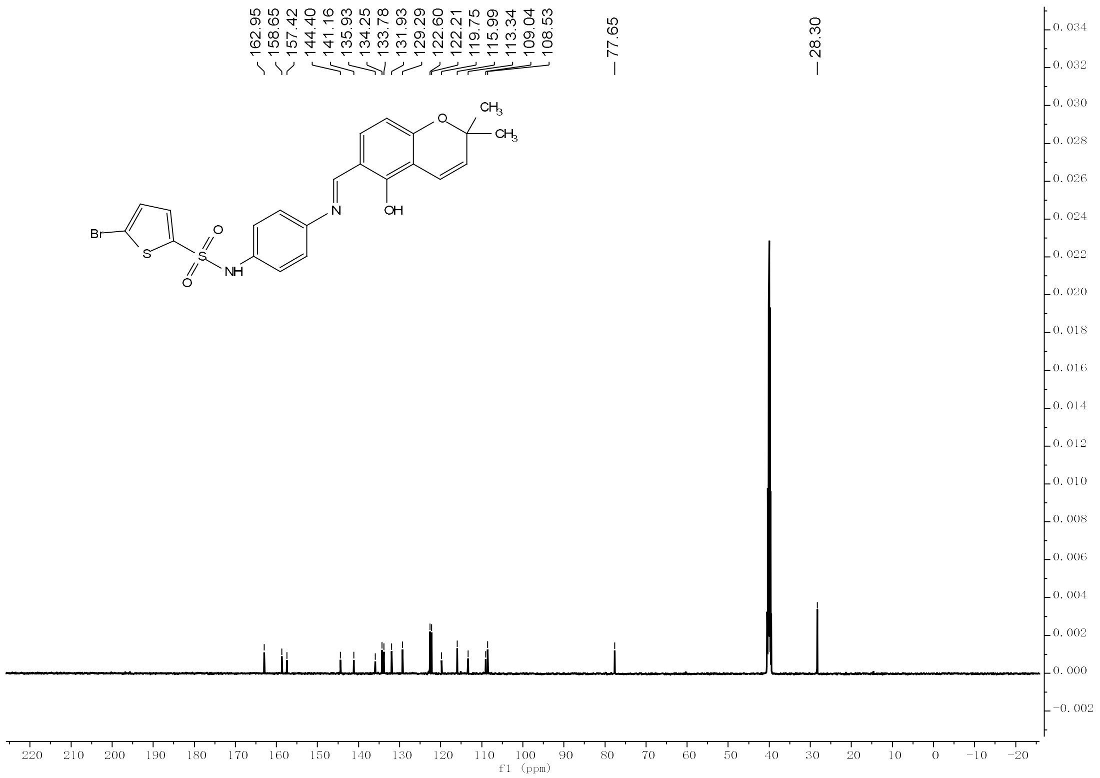

^{13}C NMR (126 MHz, DMSO-d_6) spectrum of compound **C10**.

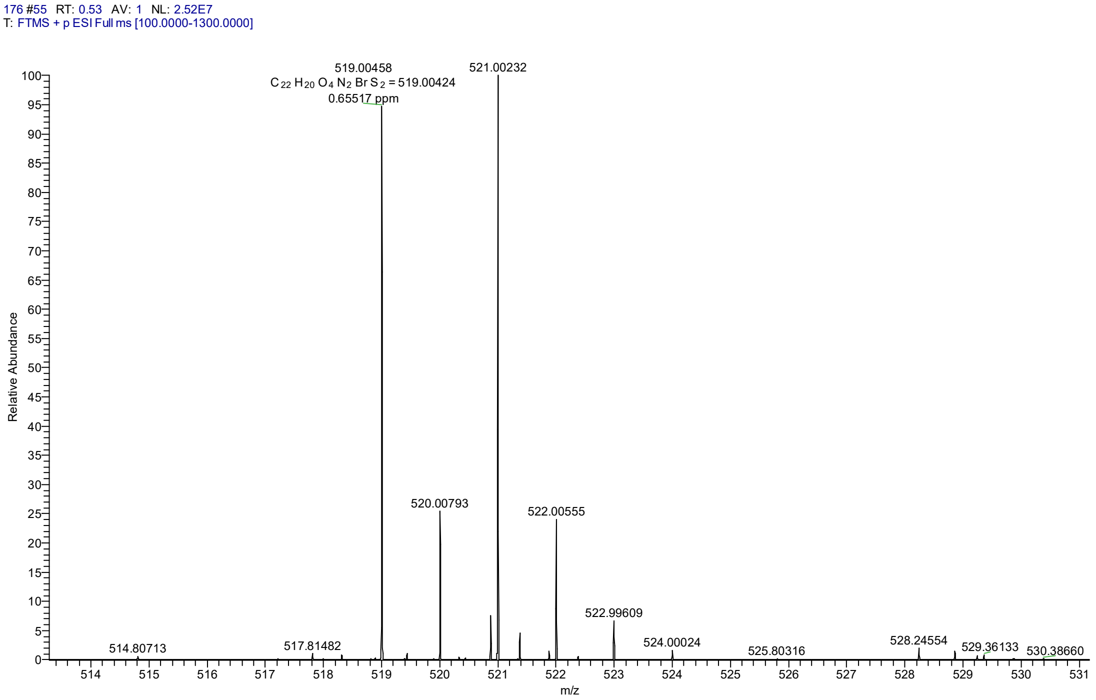

HRMS of compound **C10**.

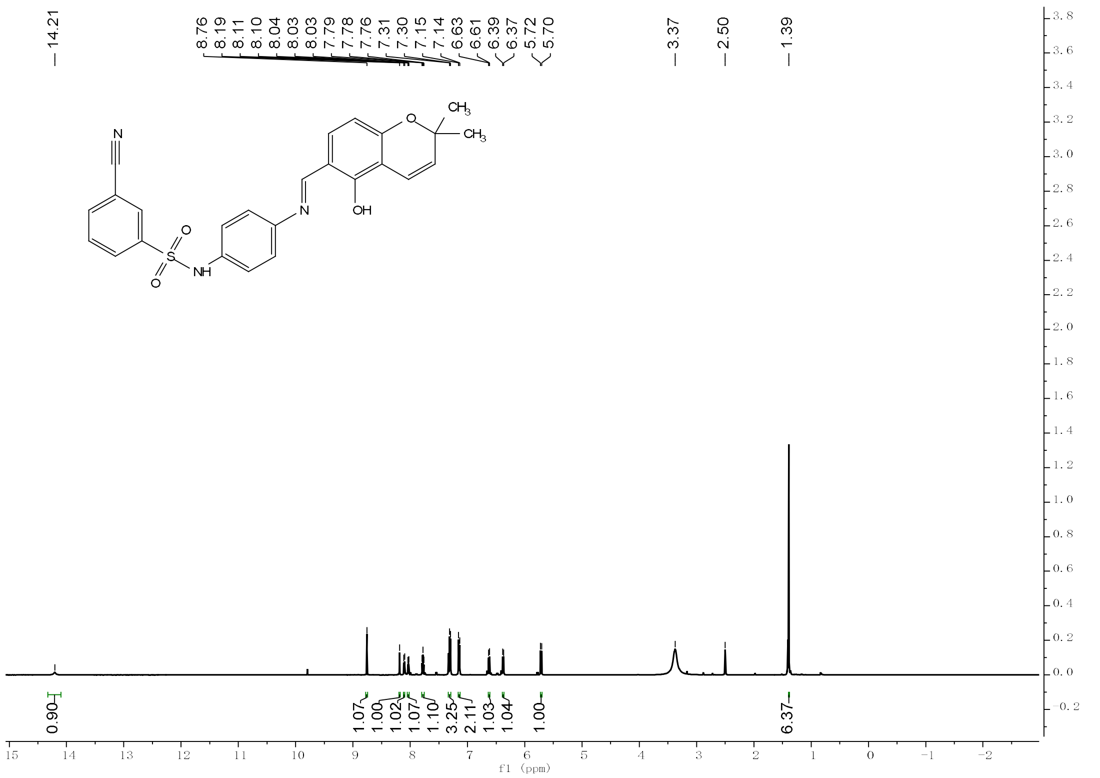

^{1}H NMR (500 MHz, DMSO-d_6) spectrum of compound **C11**.

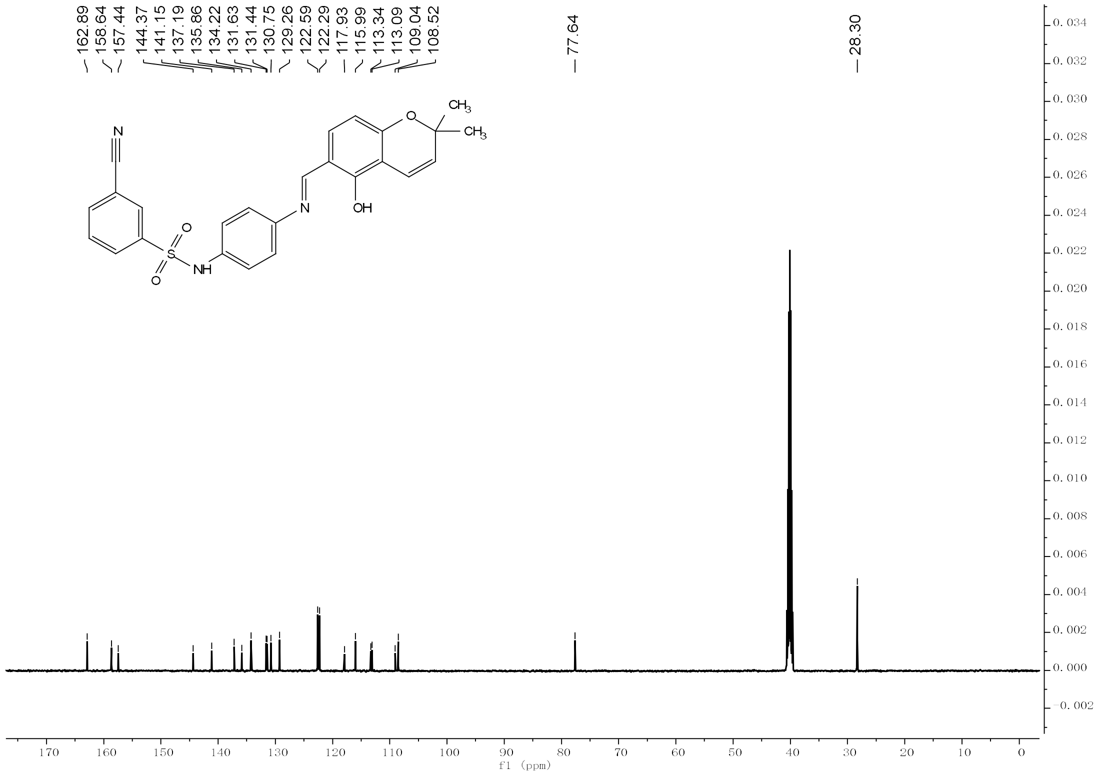

^{13}C NMR (126 MHz, DMSO-d_6) spectrum of compound **C11**.

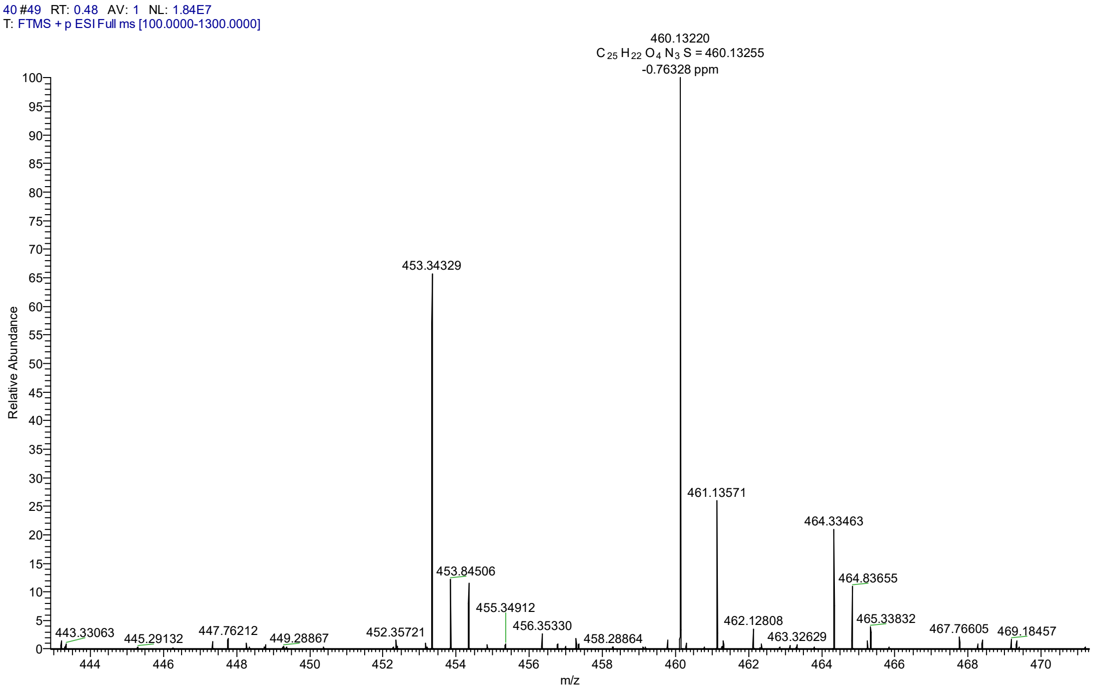

HRMS of compound **C11**.

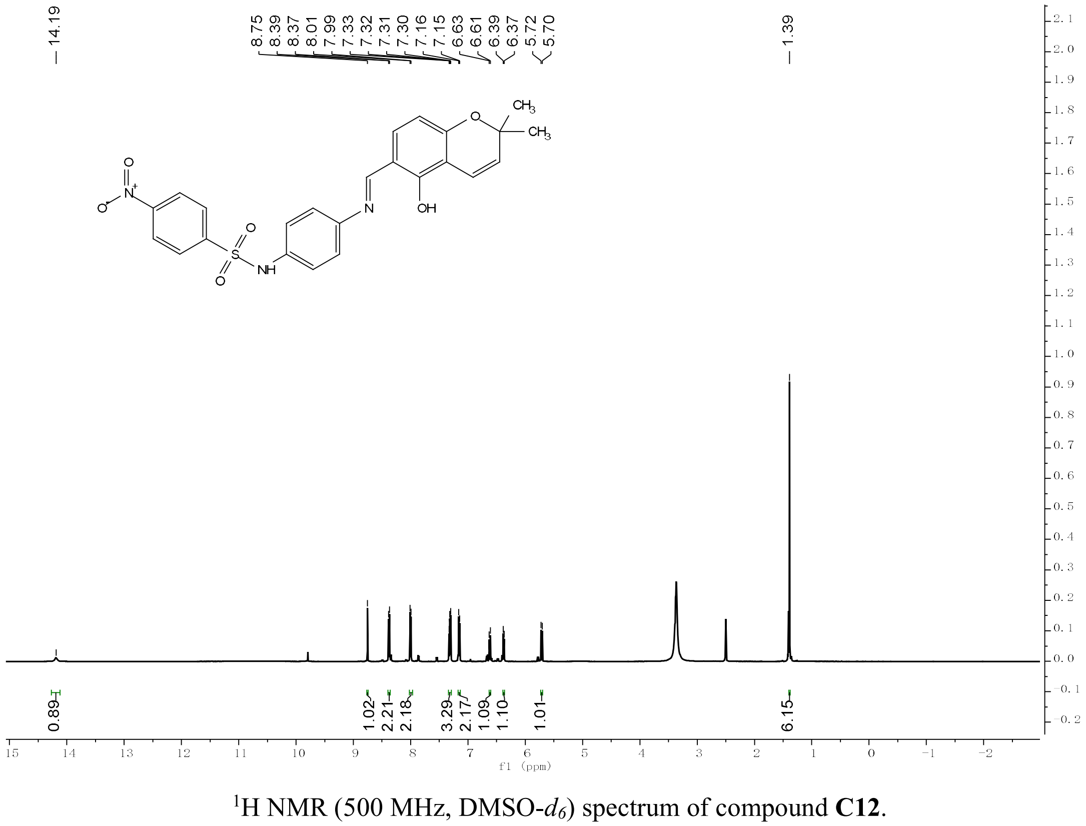

^{1}H NMR (500 MHz, DMSO-d_6) spectrum of compound **C12**.

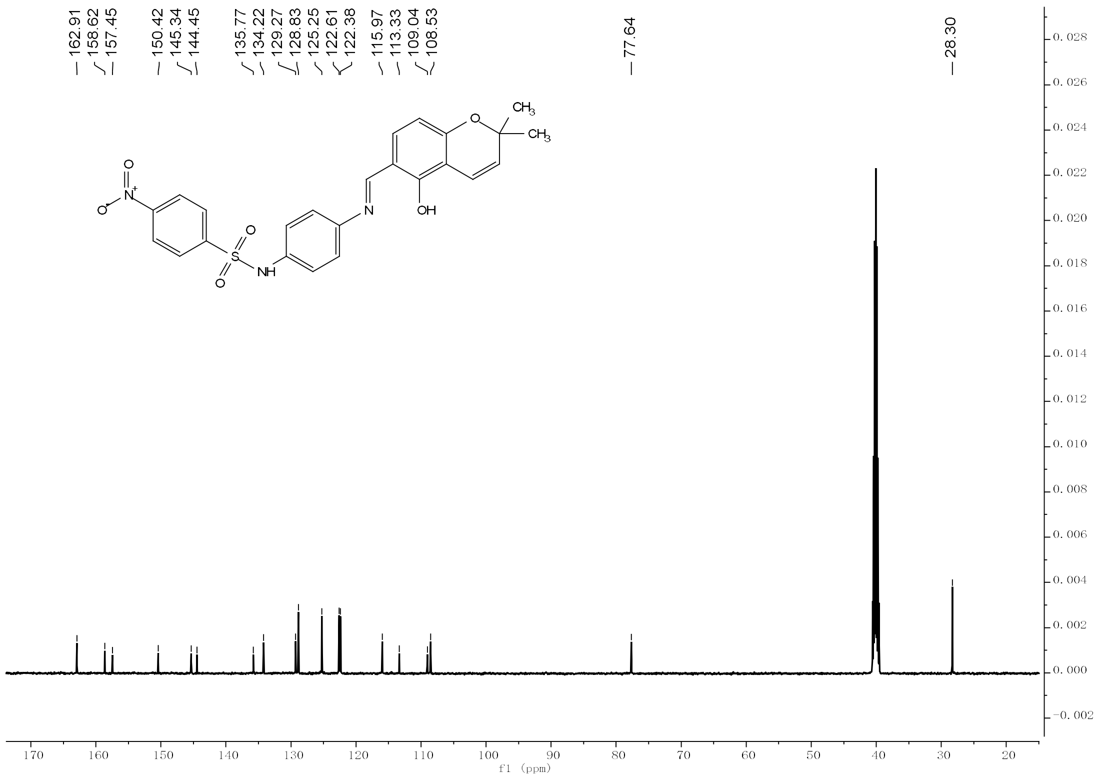

^{13}C NMR (126 MHz, DMSO-d_6) spectrum of compound **C12**.

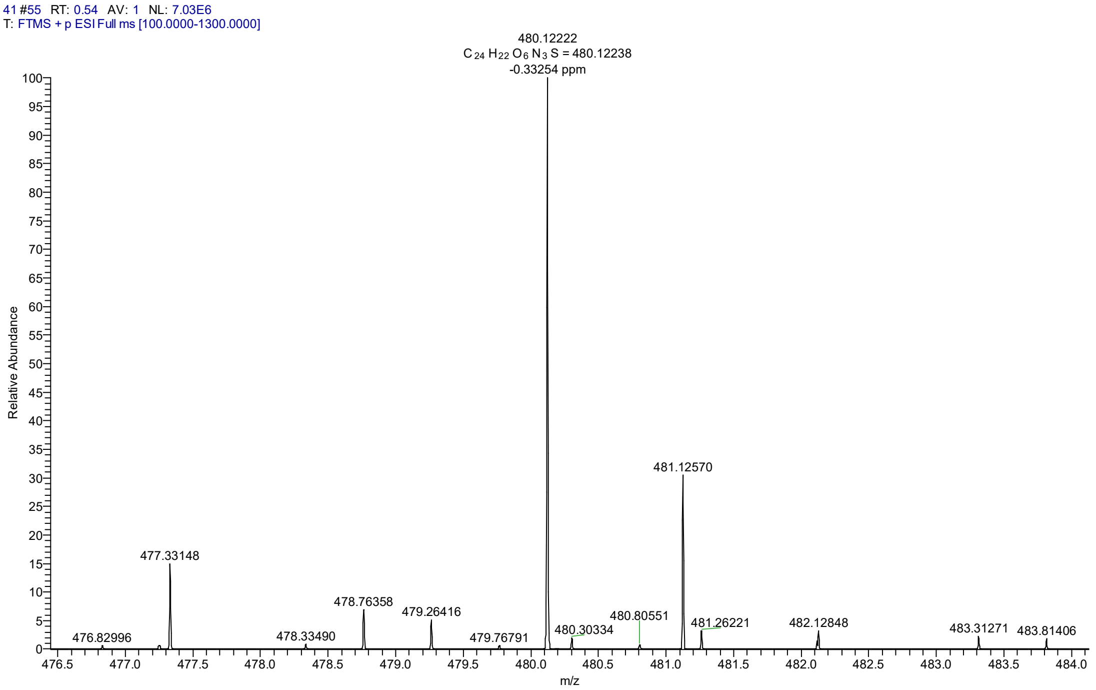

HRMS of compound **C12**.

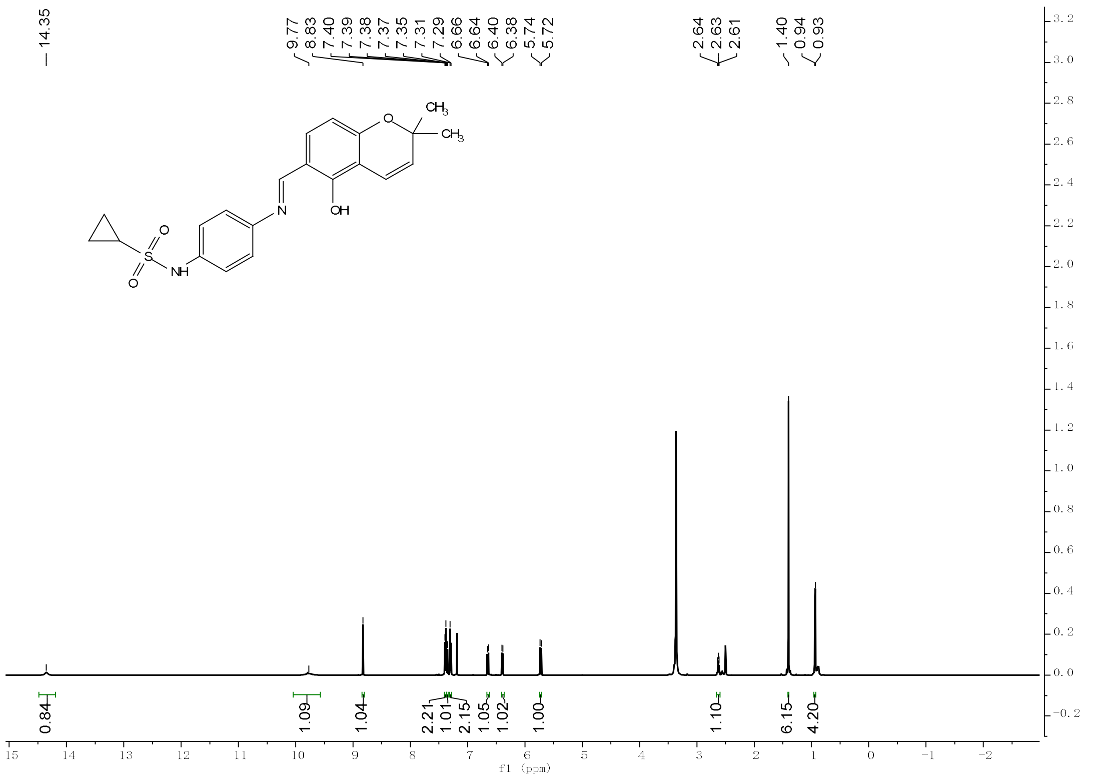

^{1}H NMR (500 MHz, DMSO-d_6) spectrum of compound **C13**.

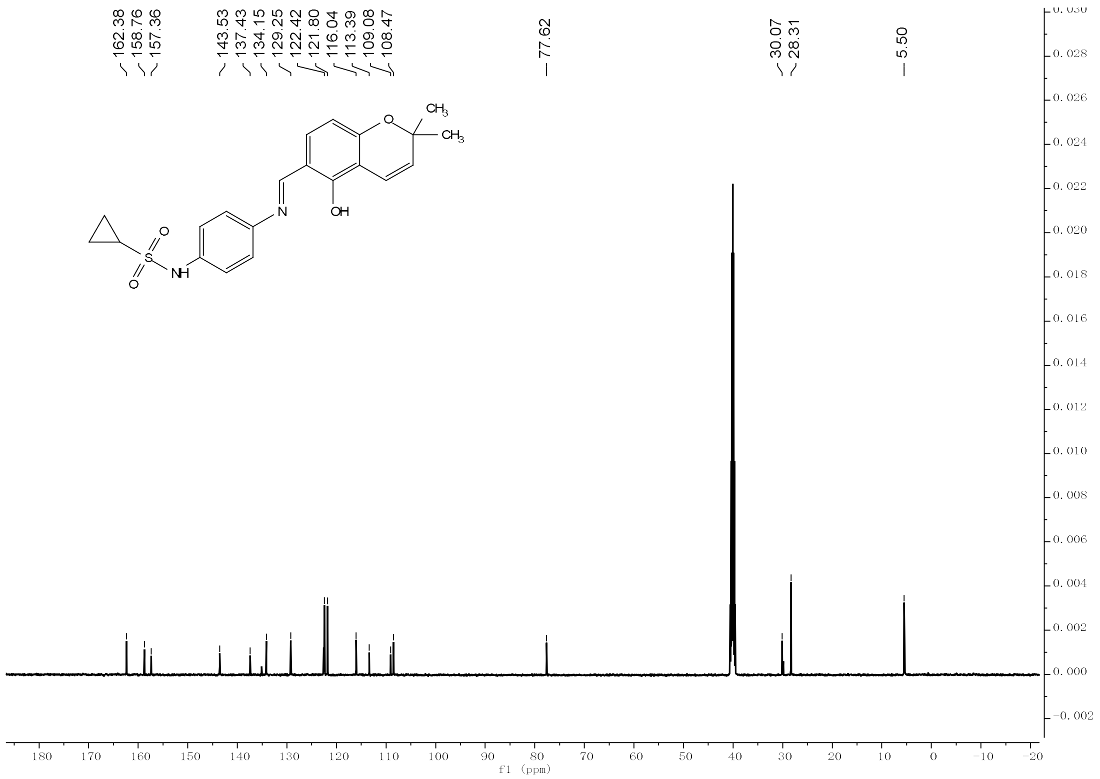

^{13}C NMR (126 MHz, DMSO-d_6) spectrum of compound **C13**.

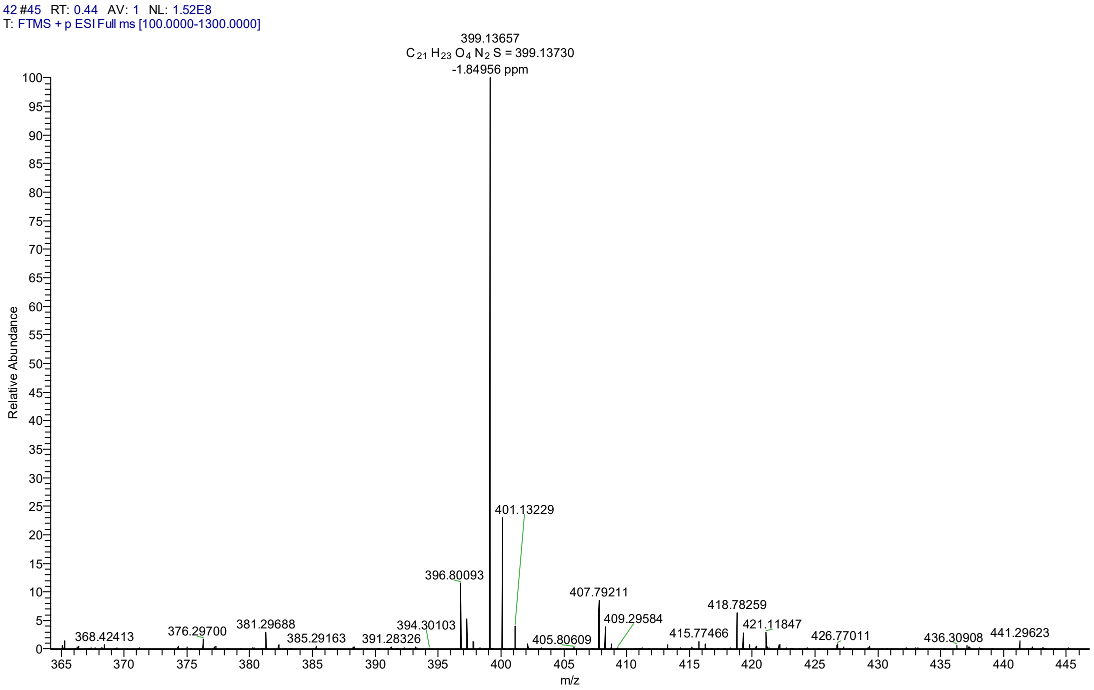

HRMS of compound **C13**.

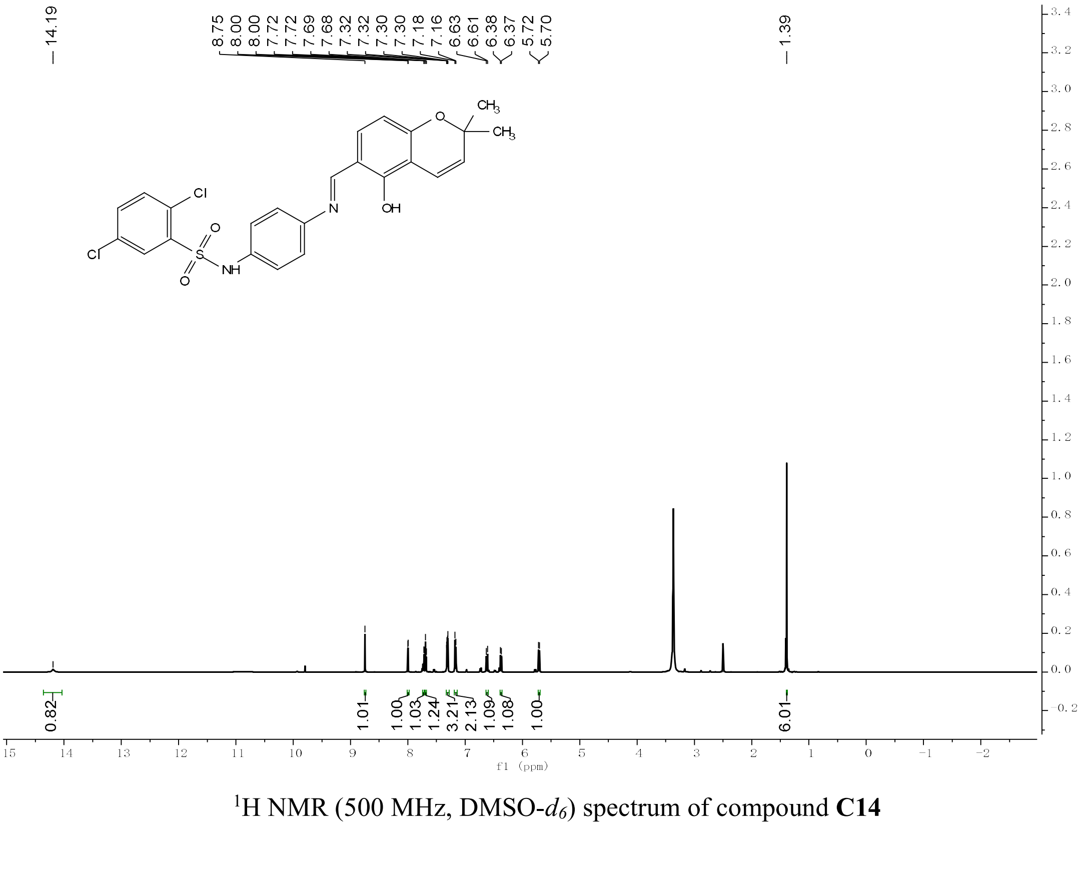

^{1}H NMR (500 MHz, DMSO-d_6) spectrum of compound **C14**

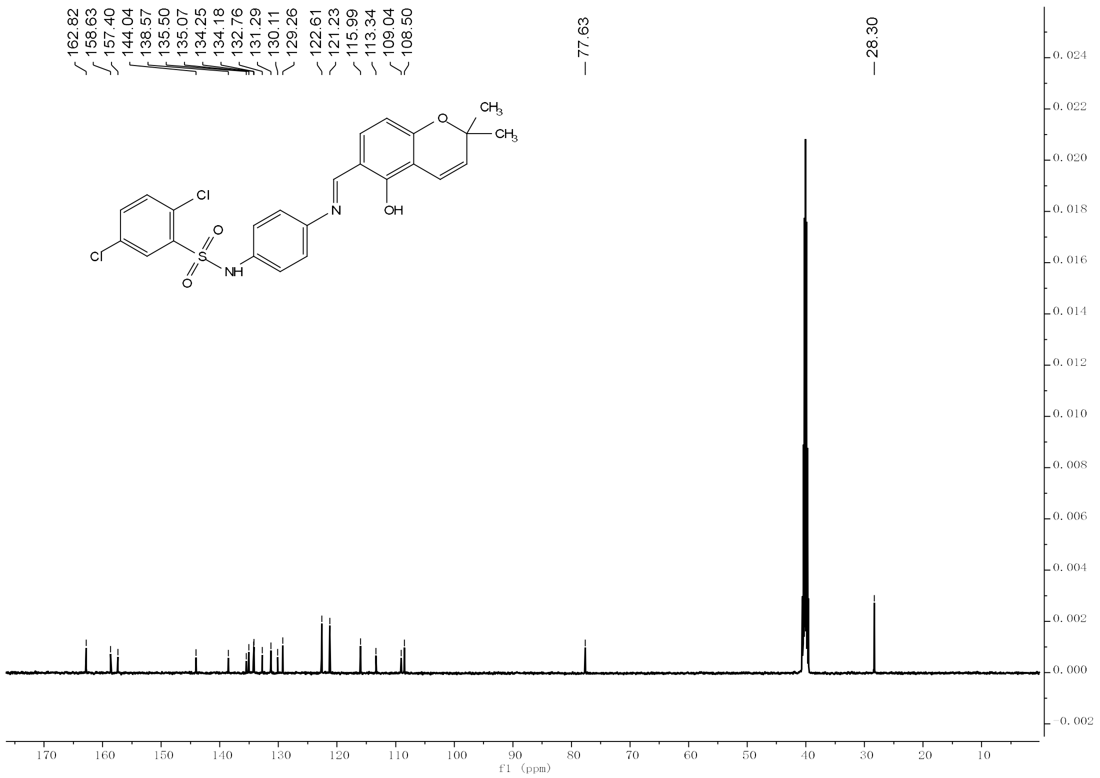

^{13}C NMR (126 MHz, DMSO-d_6) spectrum of compound **C14**.

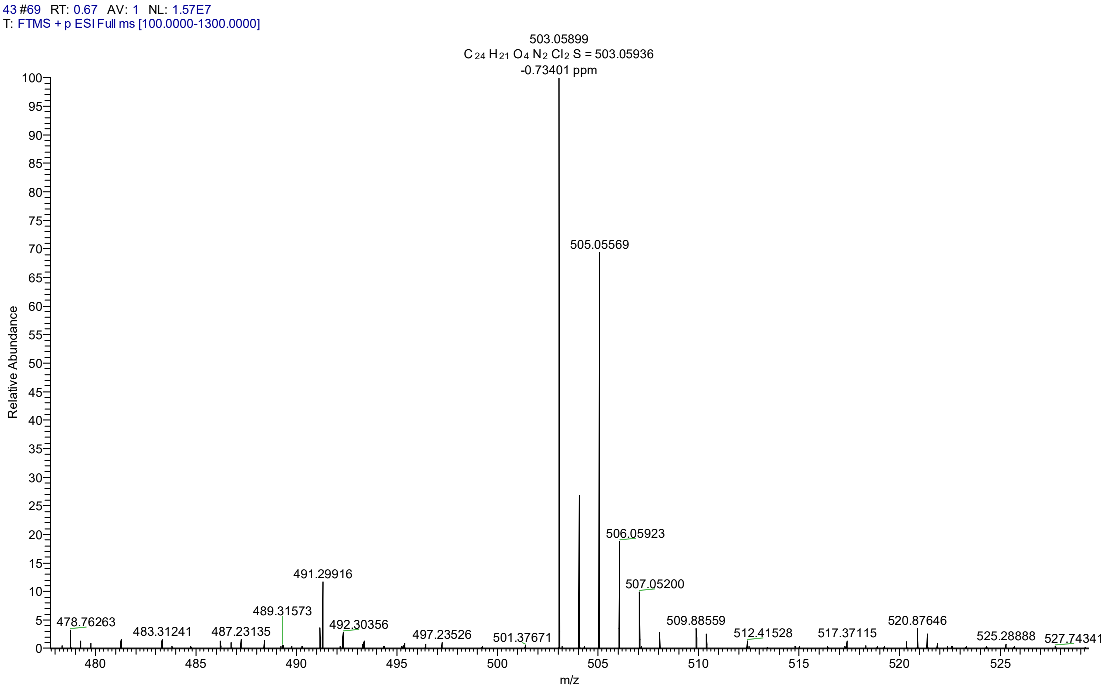

HRMS of compound **C14**.

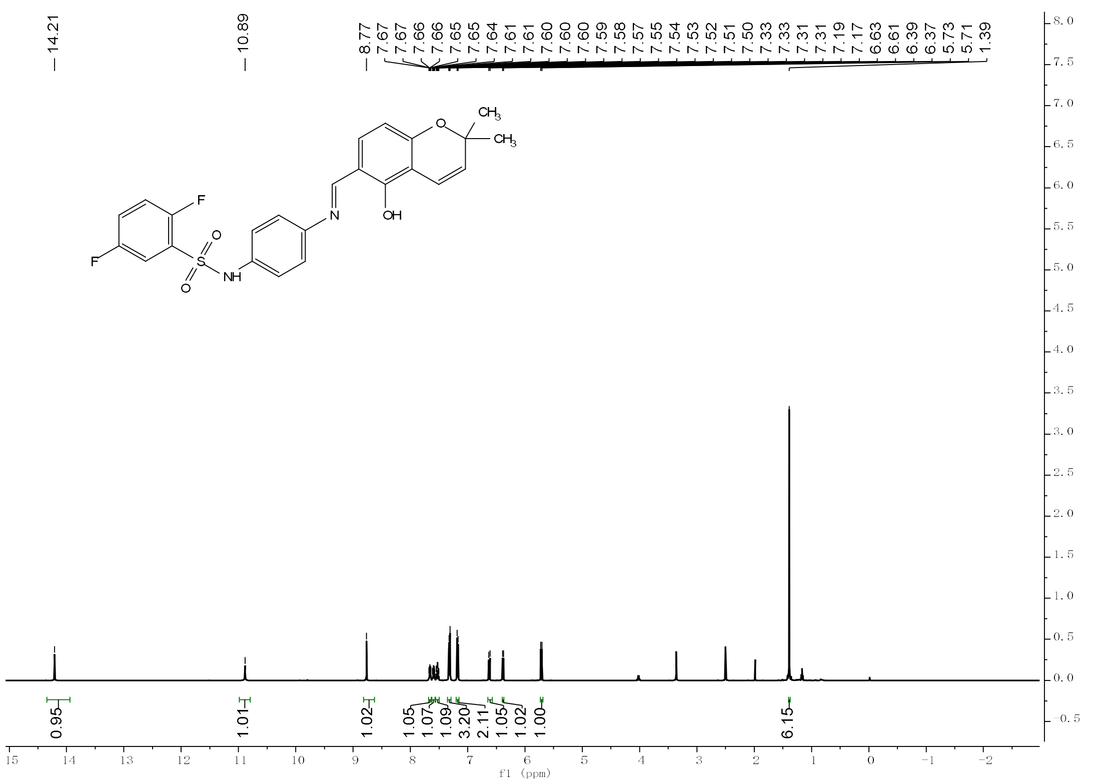

^{1}H NMR (500 MHz, DMSO-d_6) spectrum of compound **C15**.

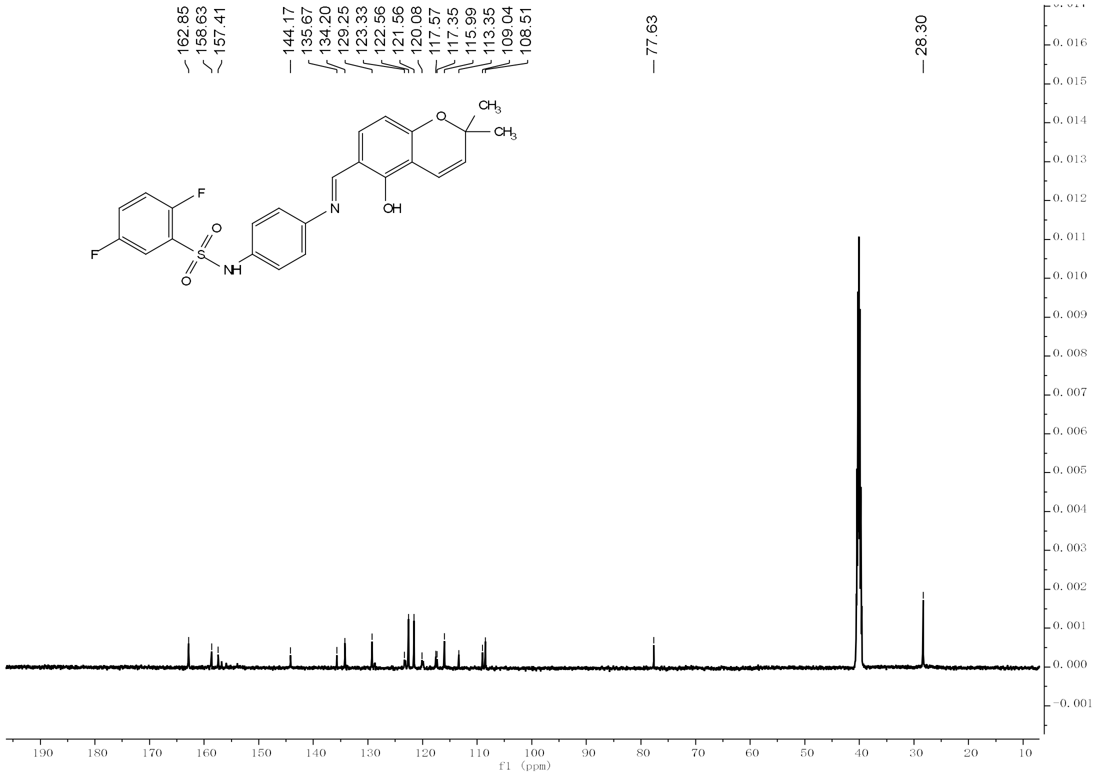

^{13}C NMR (126 MHz, DMSO-d_6) spectrum of compound **C15**.

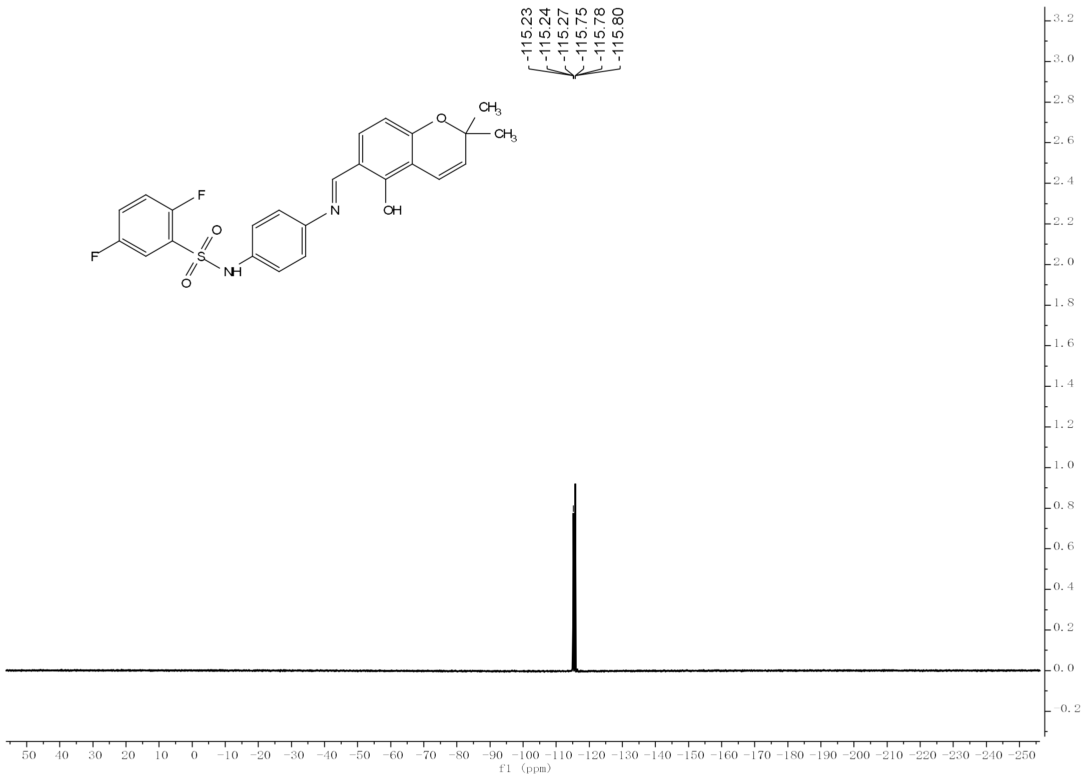

^{19}F NMR (471 MHz, DMSO-d_6) spectrum of compound **C15**.

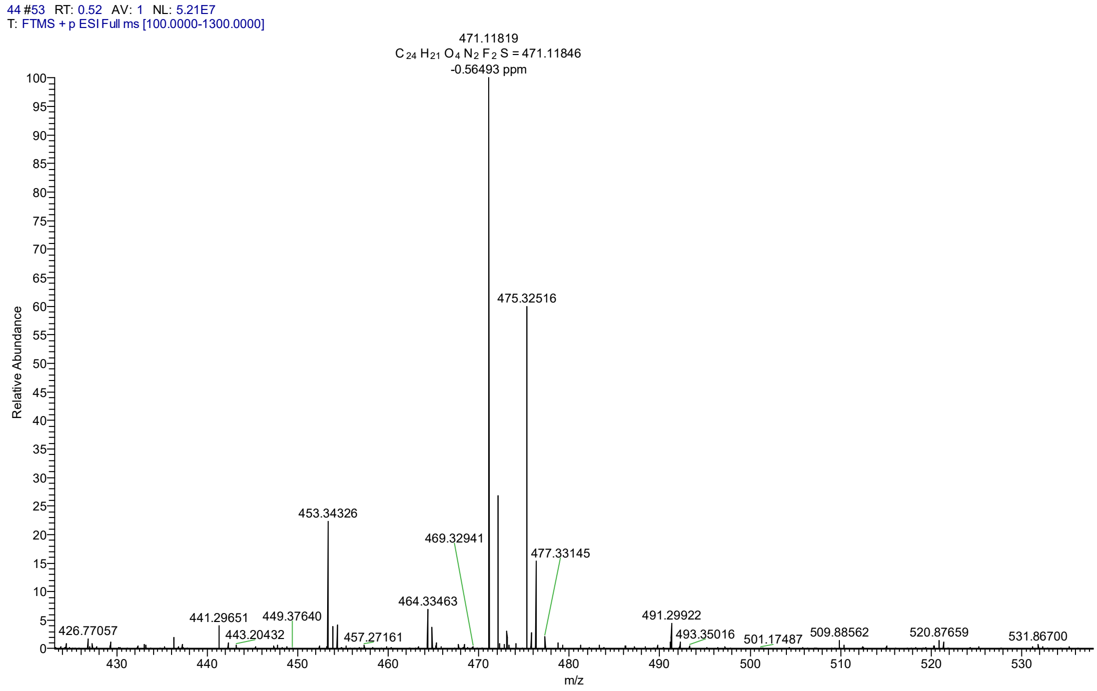

HRMS of compound **C15**.

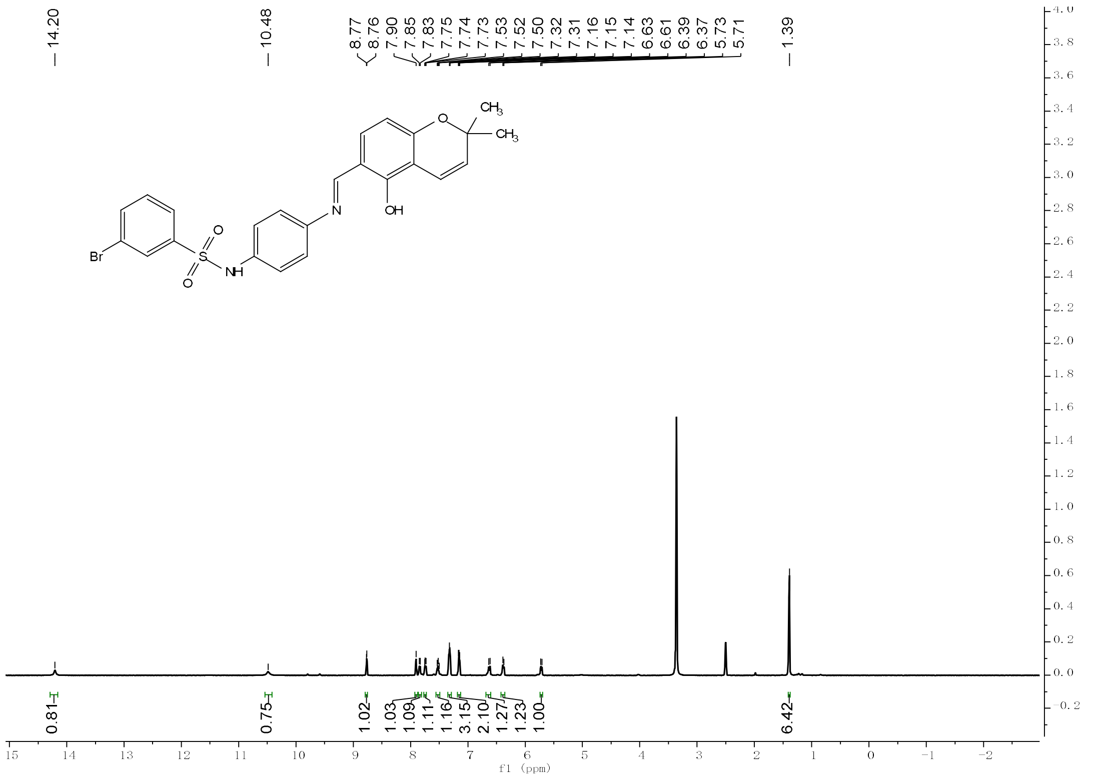

^{1}H NMR (500 MHz, DMSO-d_6) spectrum of compound **C16**.

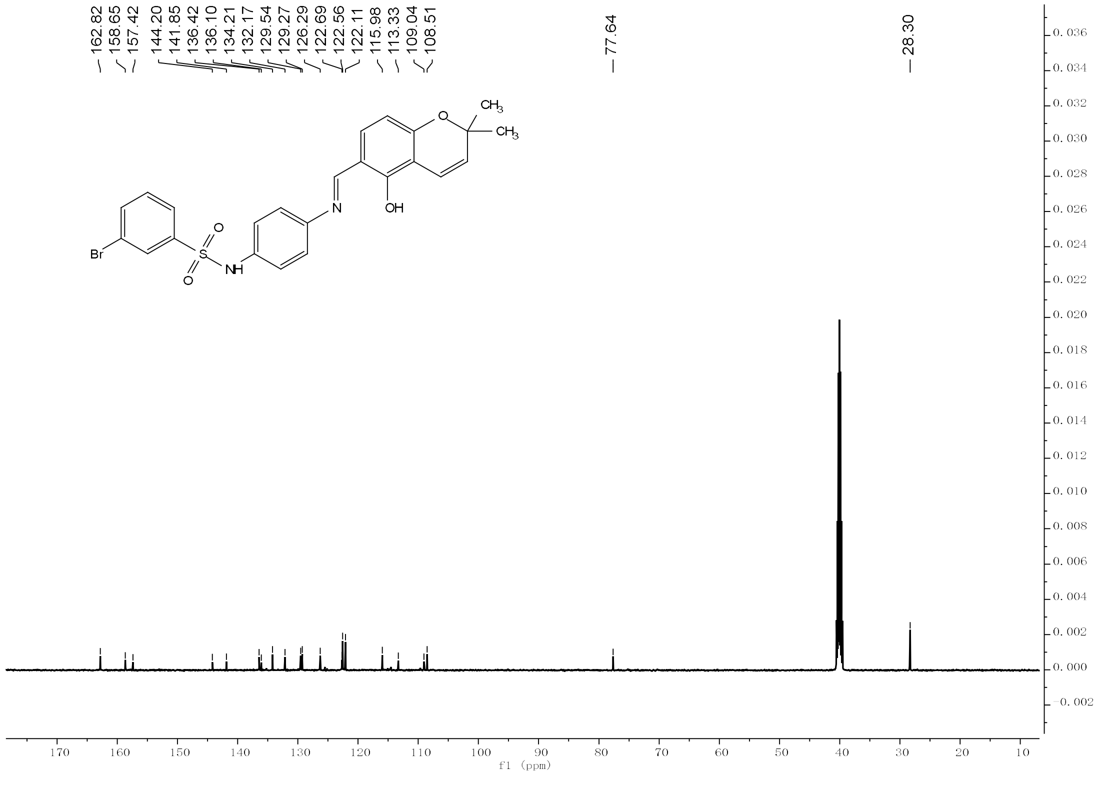

^{13}C NMR (126 MHz, DMSO-d_6) spectrum of compound **C16**.

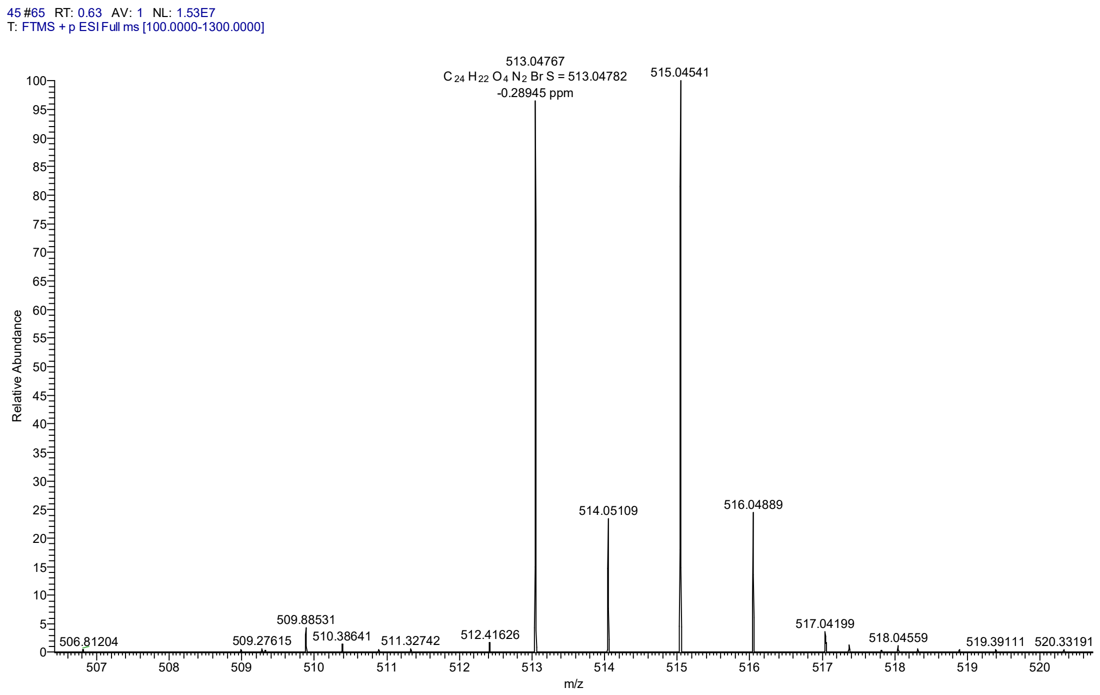

HRMS of compound **C16**.

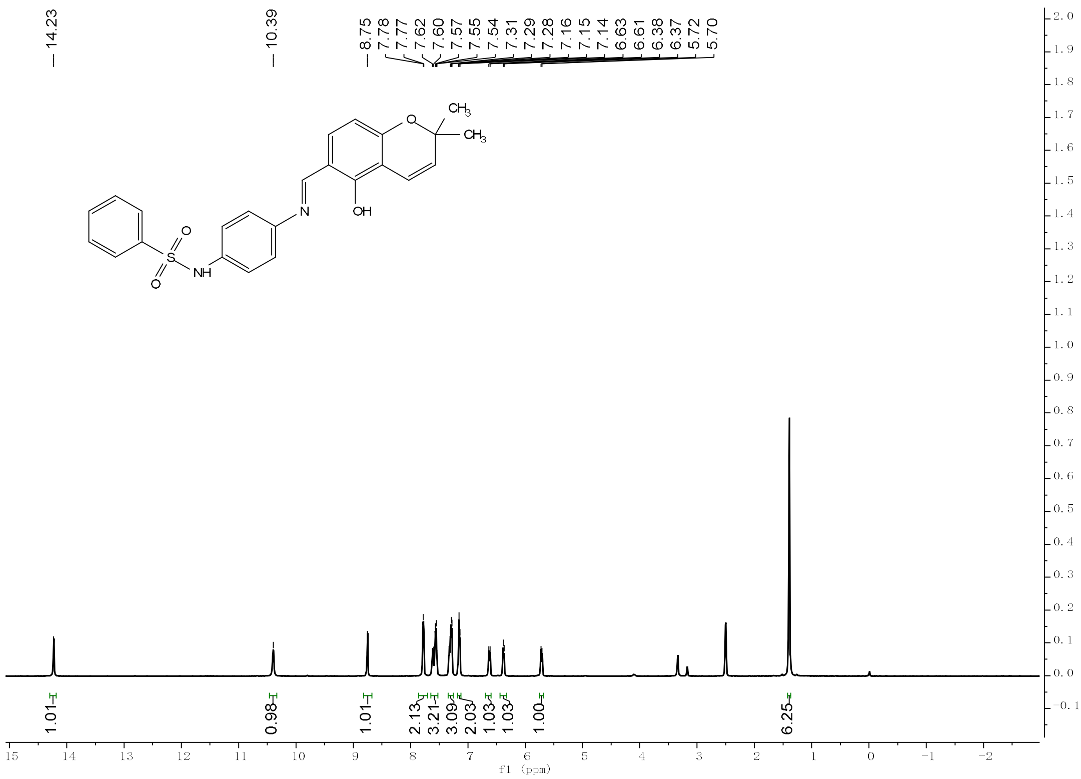

^{1}H NMR (500 MHz, DMSO-d_6) spectrum of compound **C17**.

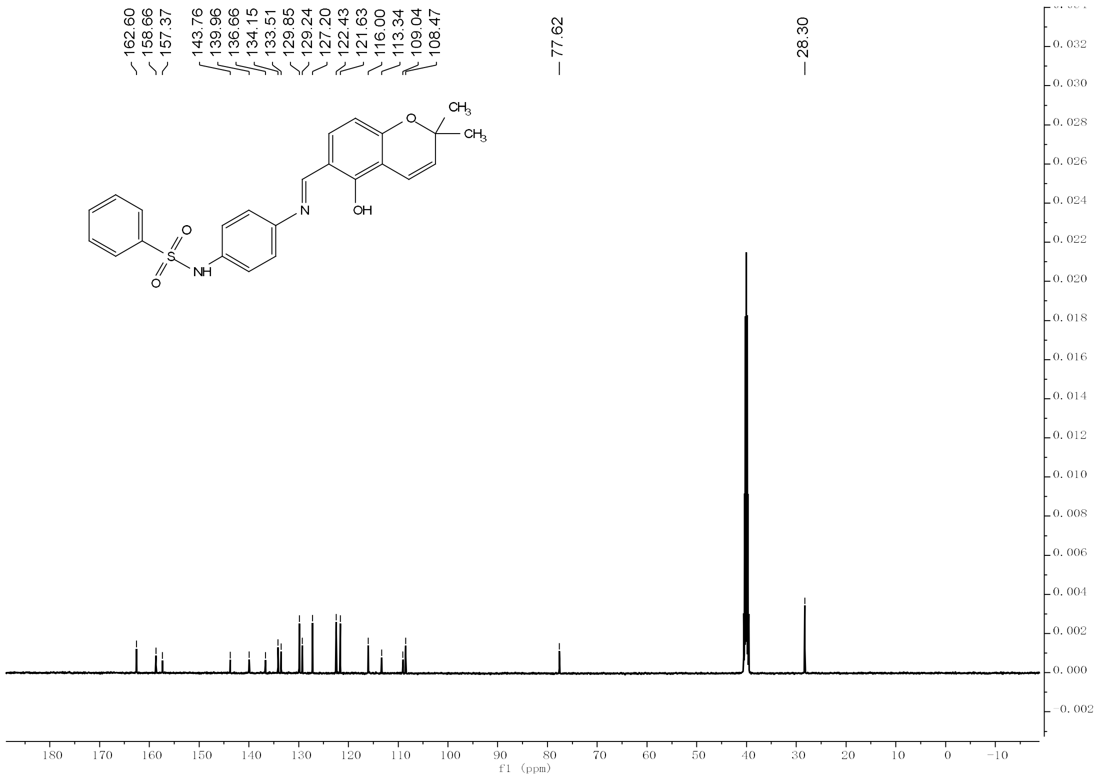

^{13}C NMR (126 MHz, DMSO-d_6) spectrum of compound **C17**.

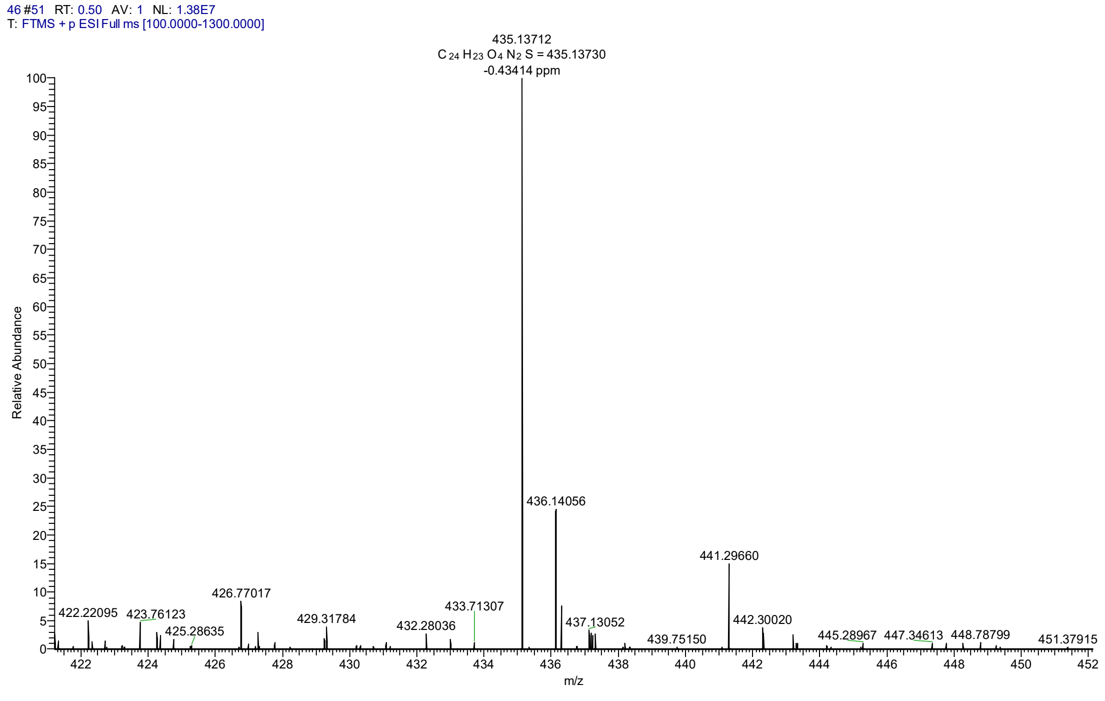

HRMS of compound **C17**.

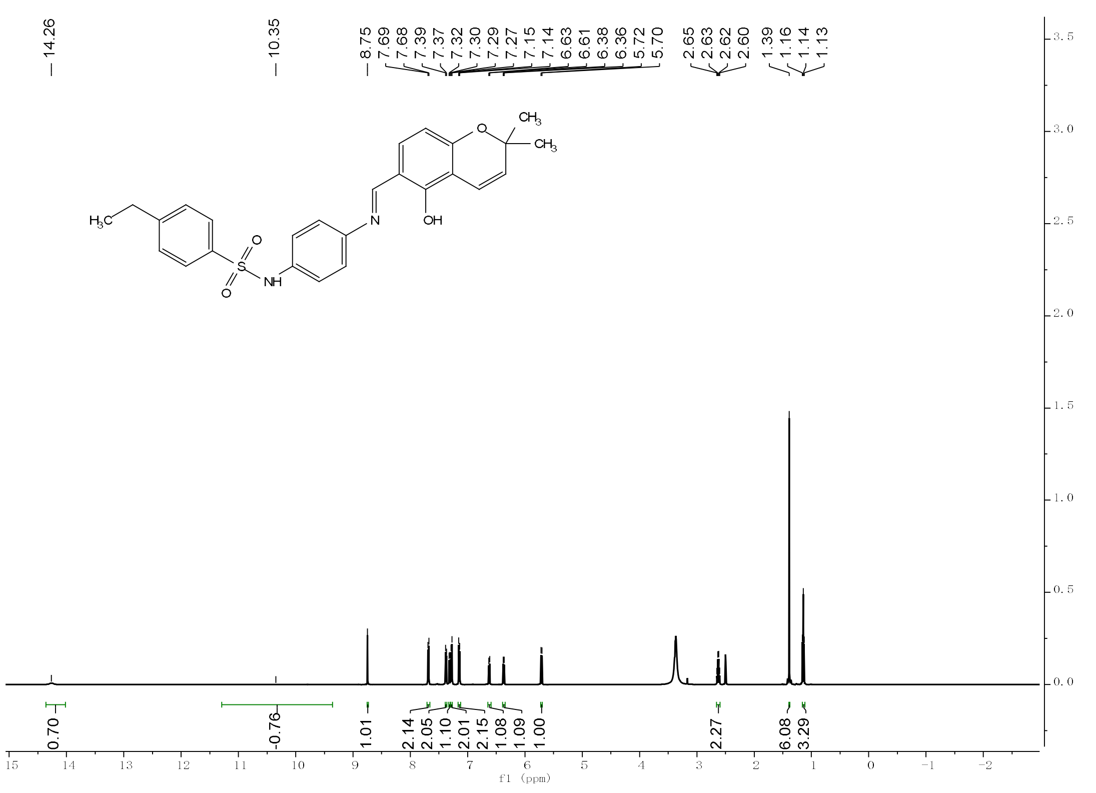

^{1}H NMR (500 MHz, DMSO-d_6) spectrum of compound **C18**.

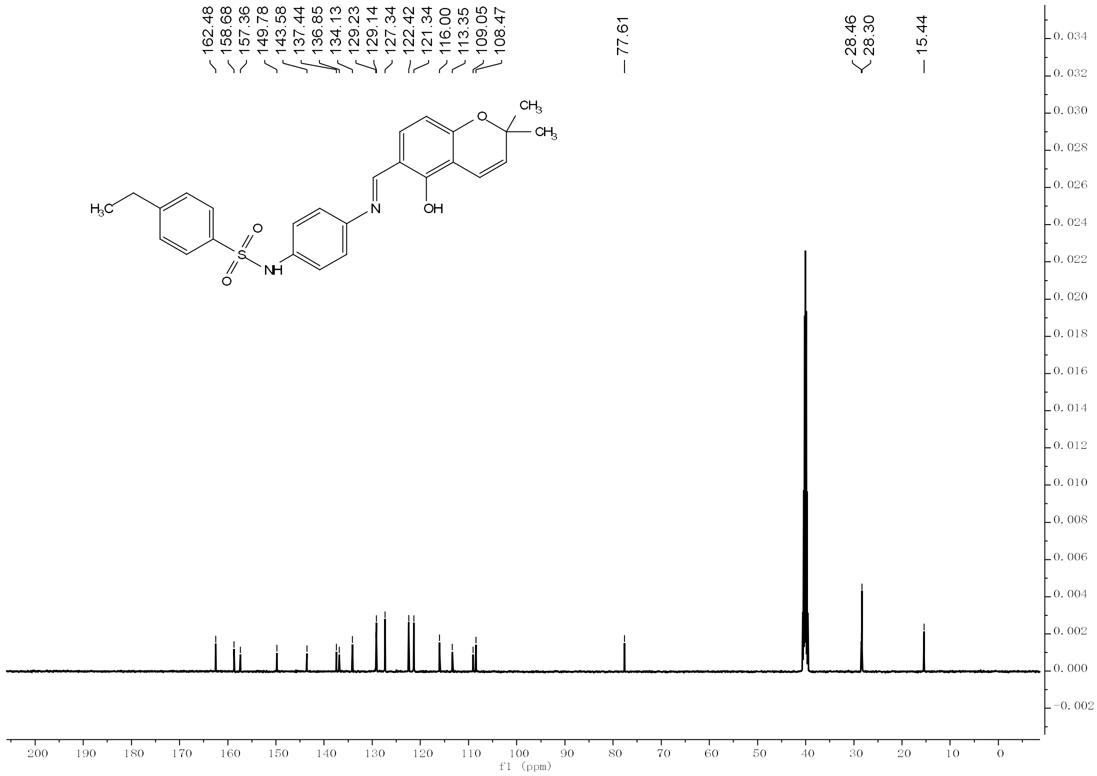

^{13}C NMR (126 MHz, DMSO-d_6) spectrum of compound **C18**.

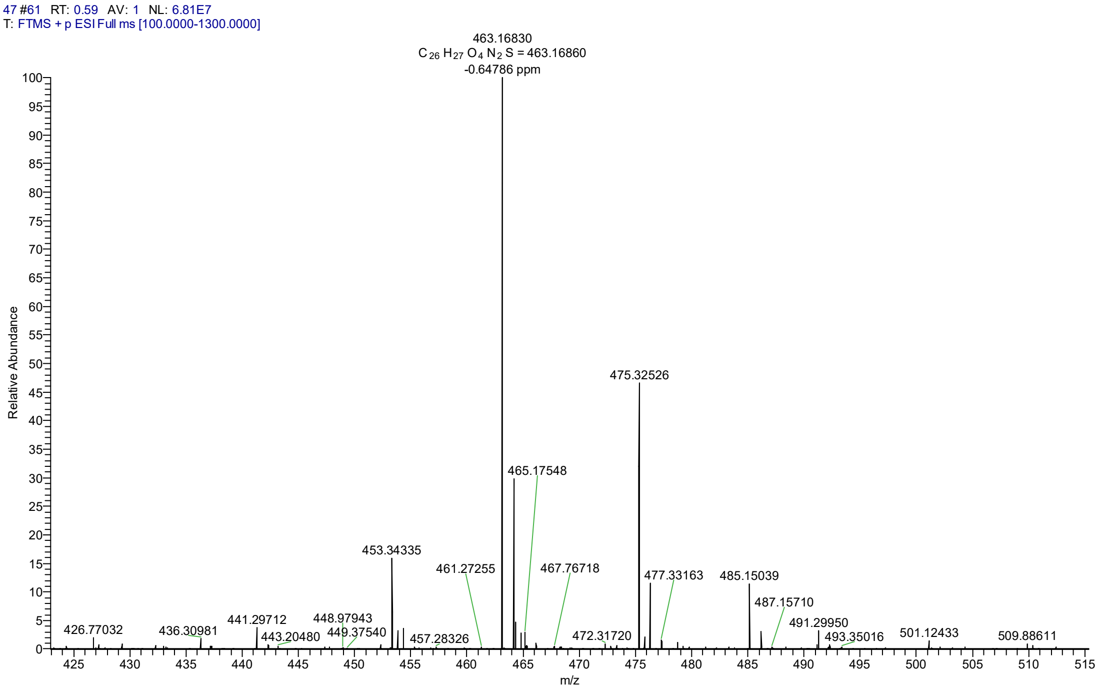

HRMS of compound **C18**.

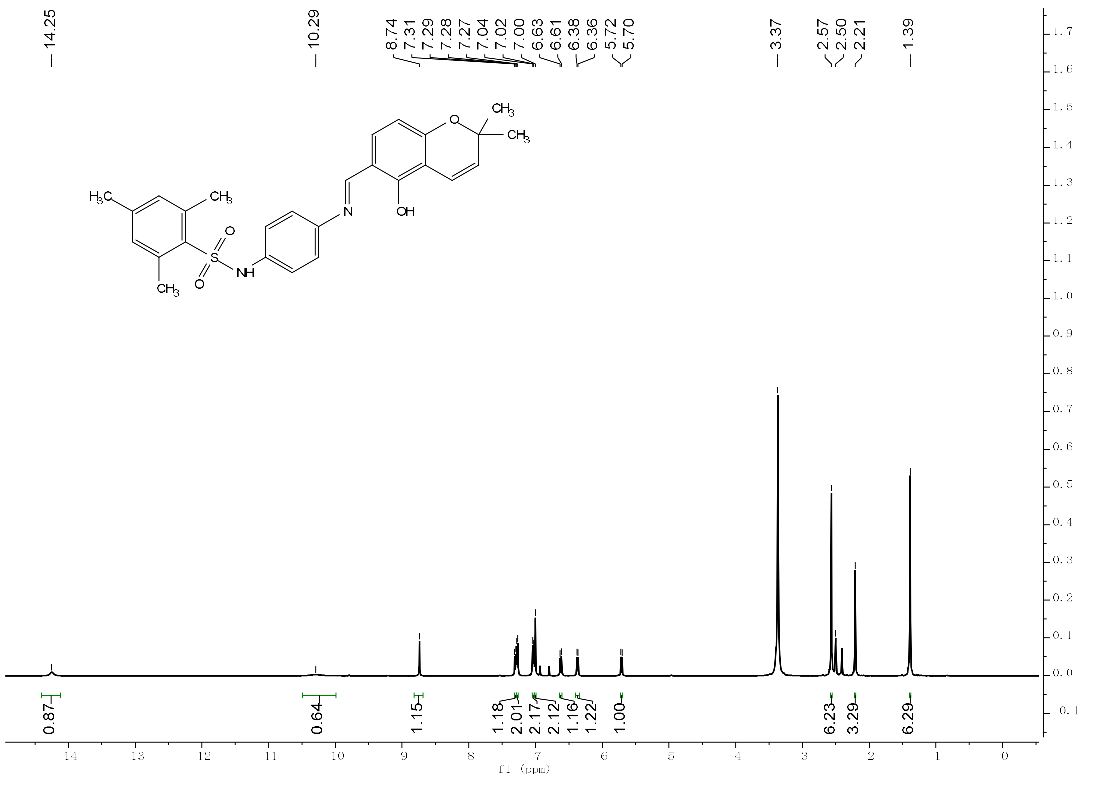

^{1}H NMR (500 MHz, DMSO-d_6) spectrum of compound **C19**.

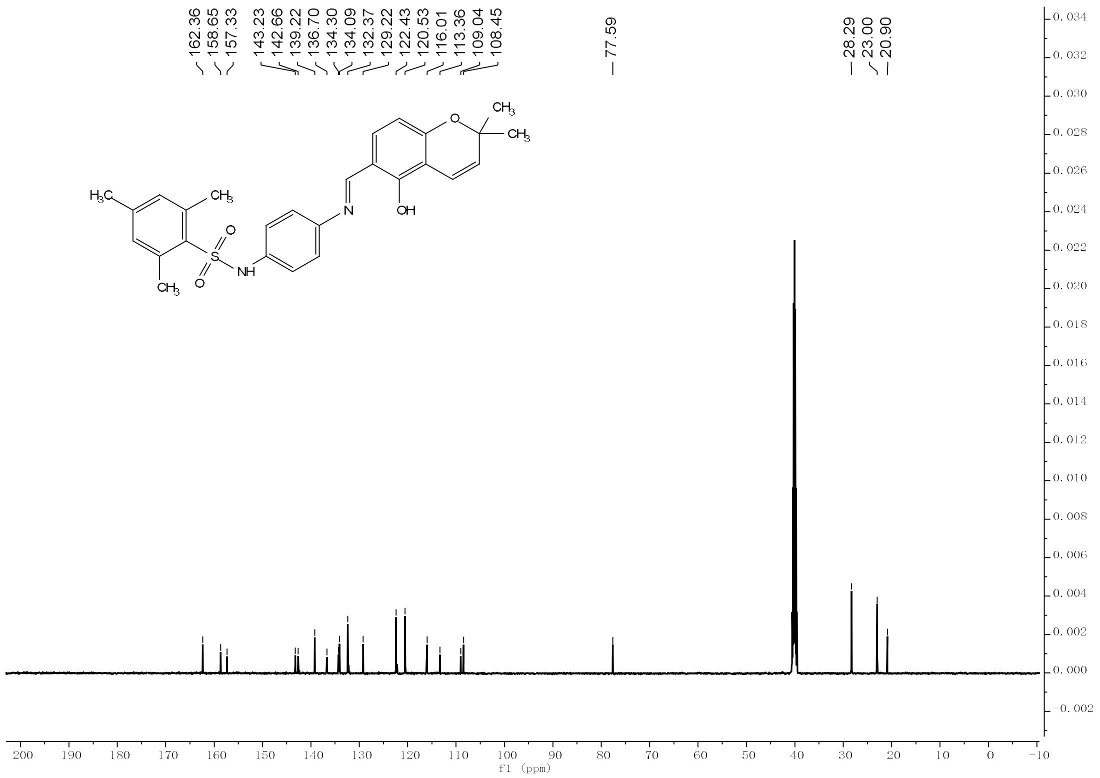

^{13}C NMR (126 MHz, DMSO-d_6) spectrum of compound **C19**.

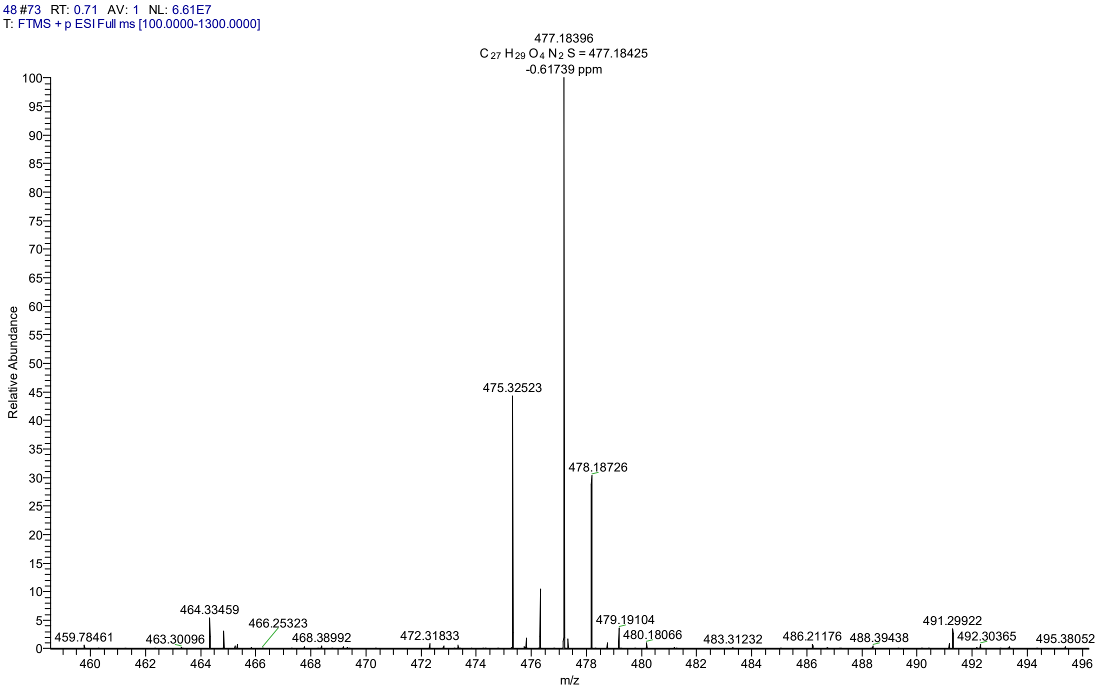

HRMS of compound **C19**.

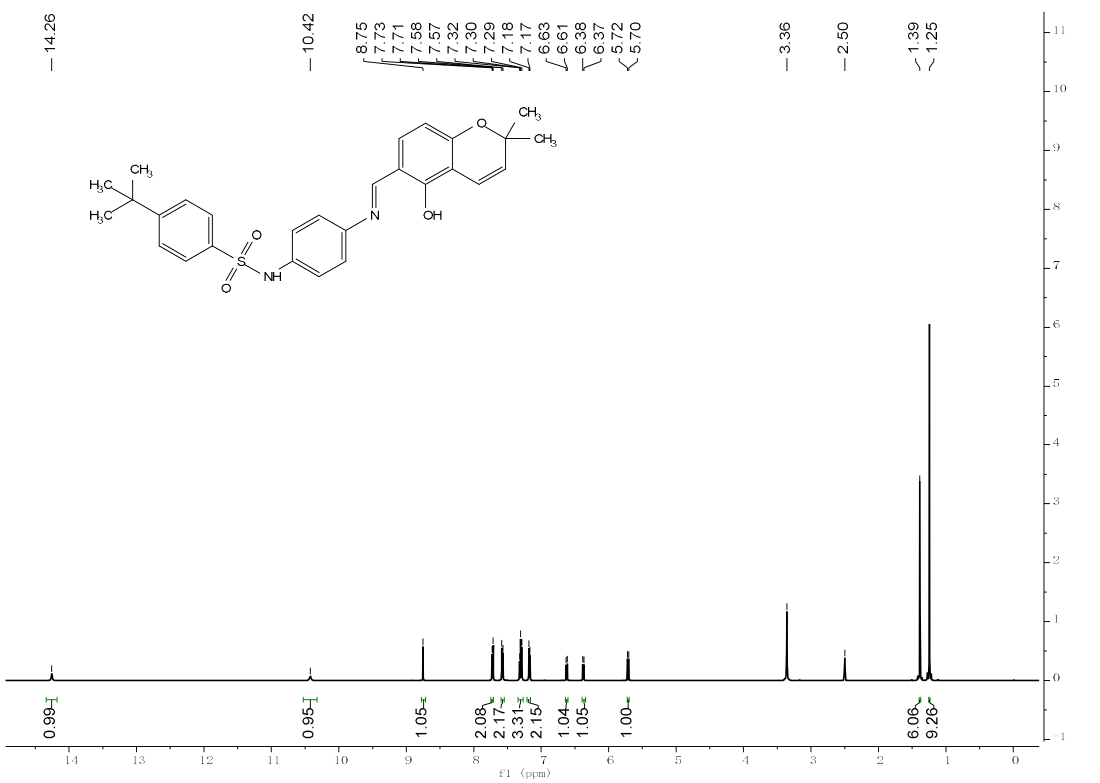

^{1}H NMR (500 MHz, DMSO-d_6) spectrum of compound **C20**.

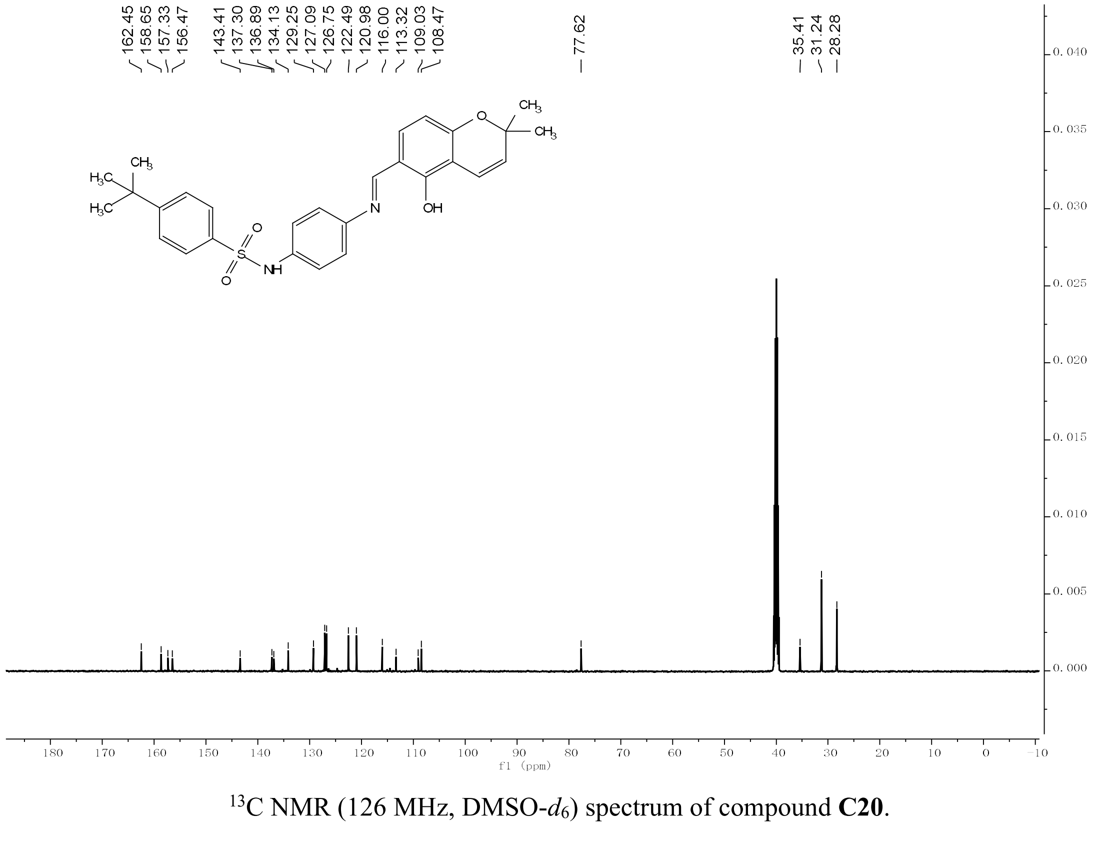

^{13}C NMR (126 MHz, DMSO-d_6) spectrum of compound **C20**.

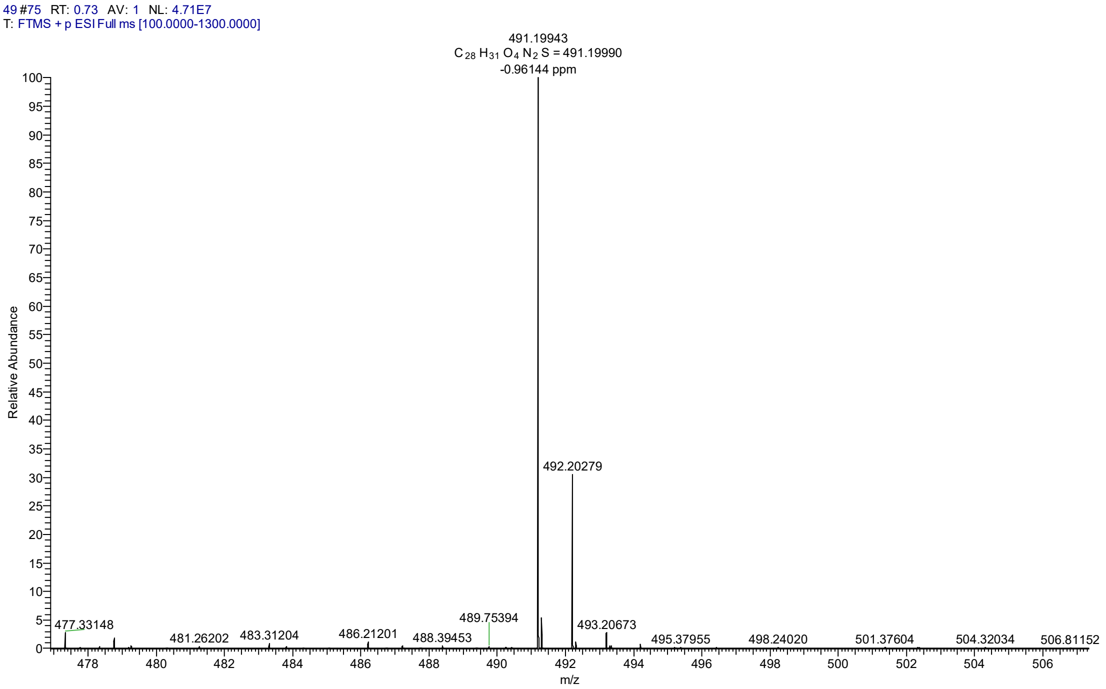

HRMS of compound **C20**.

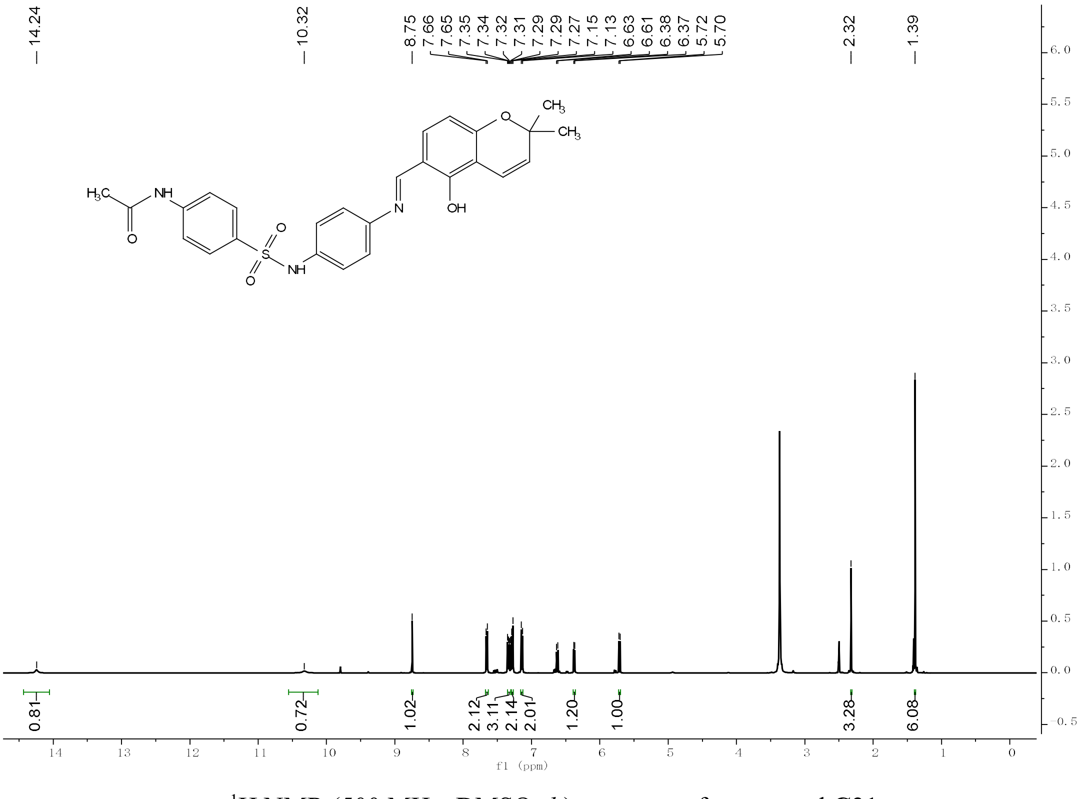

^{1}H NMR (500 MHz, DMSO-d_6) spectrum of compound **C21**.

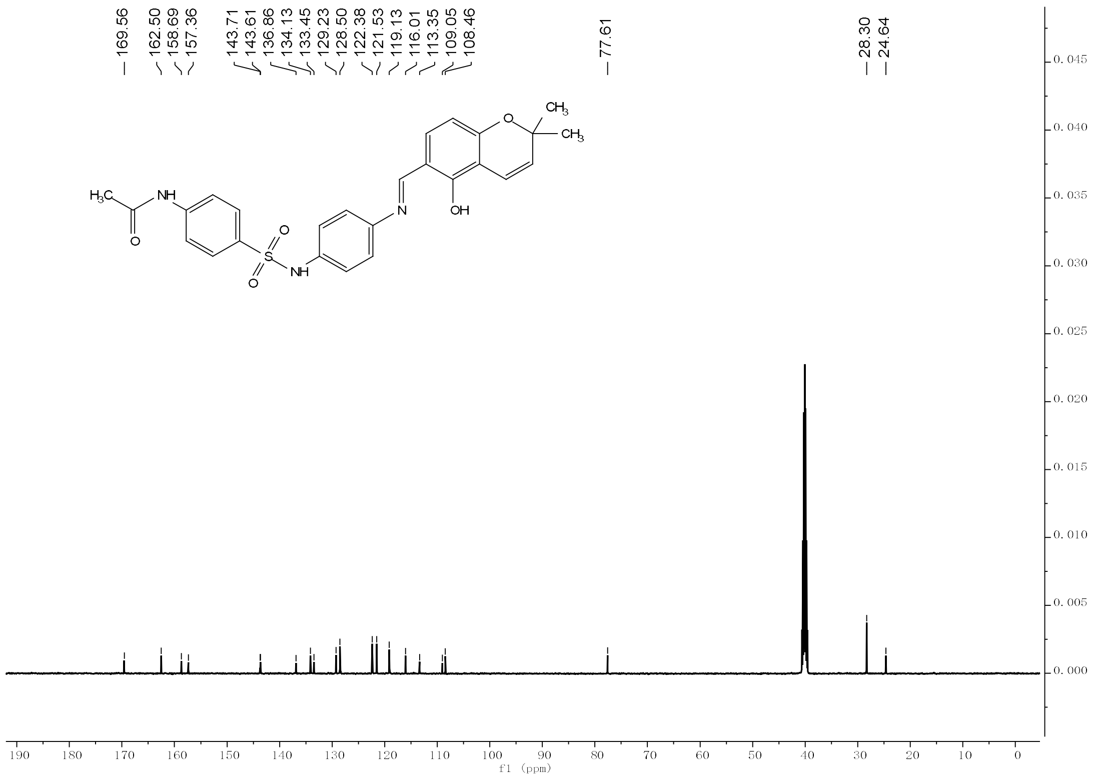

^{13}C NMR (126 MHz, DMSO-d_6) spectrum of compound **C21**.

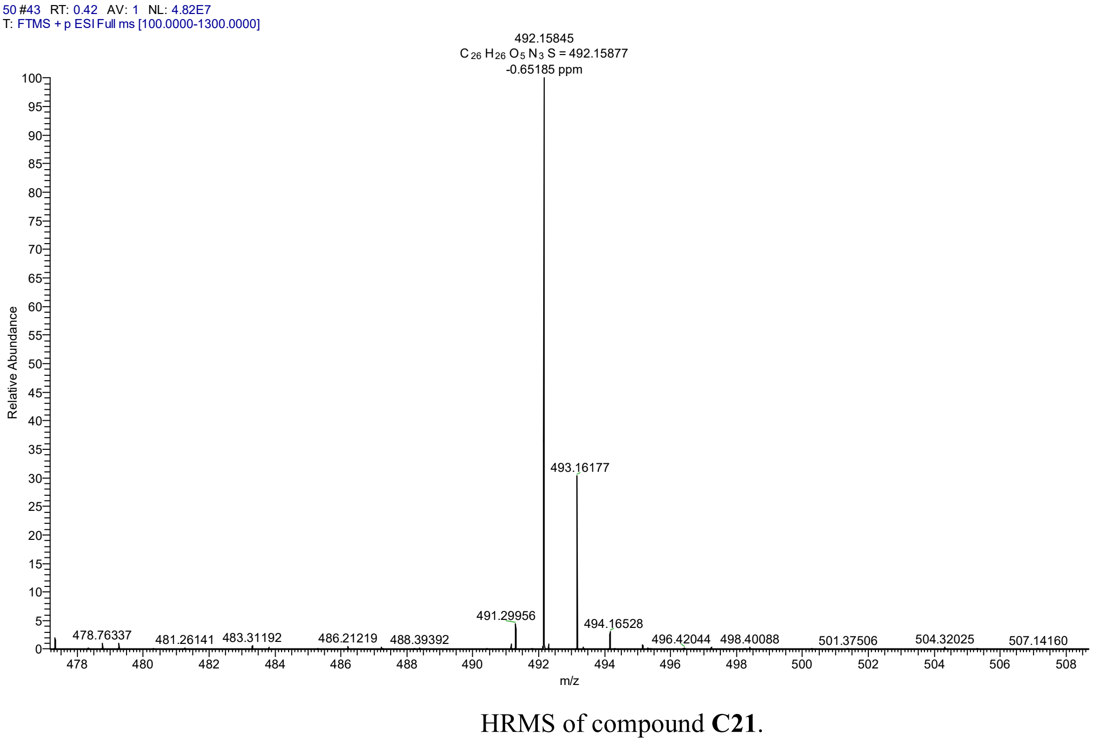

HRMS of compound **C21**.

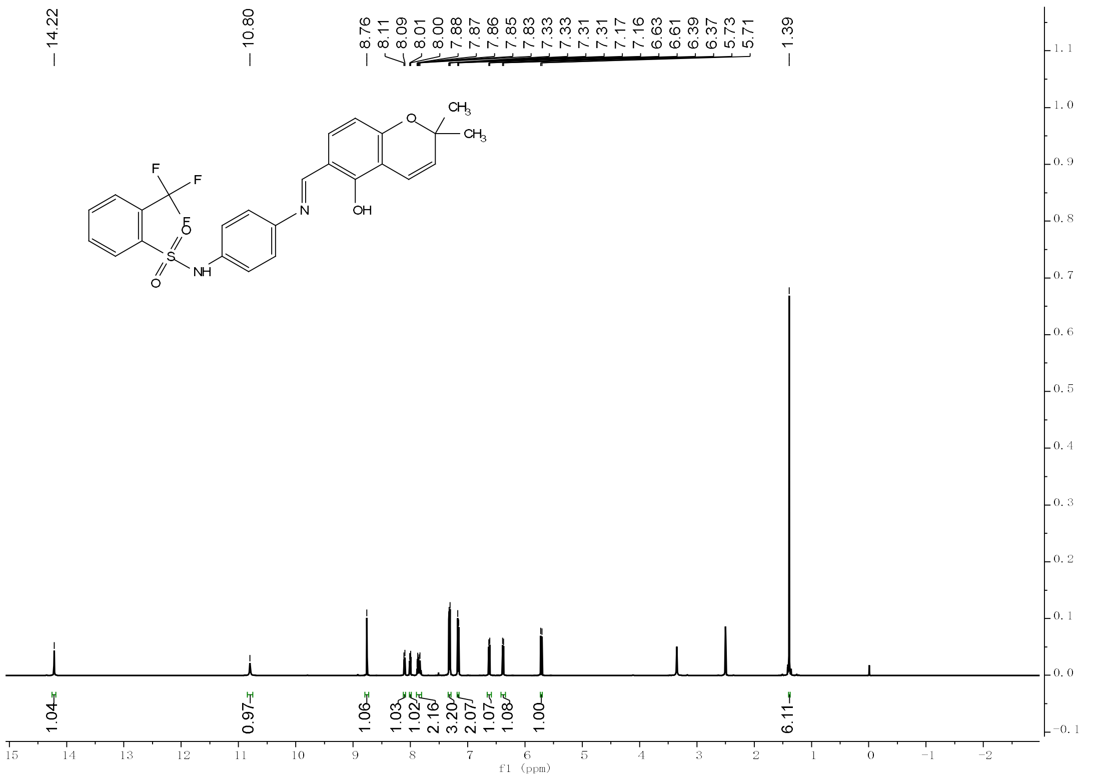

^{1}H NMR (500 MHz, DMSO-d_6) spectrum of compound **C22**.

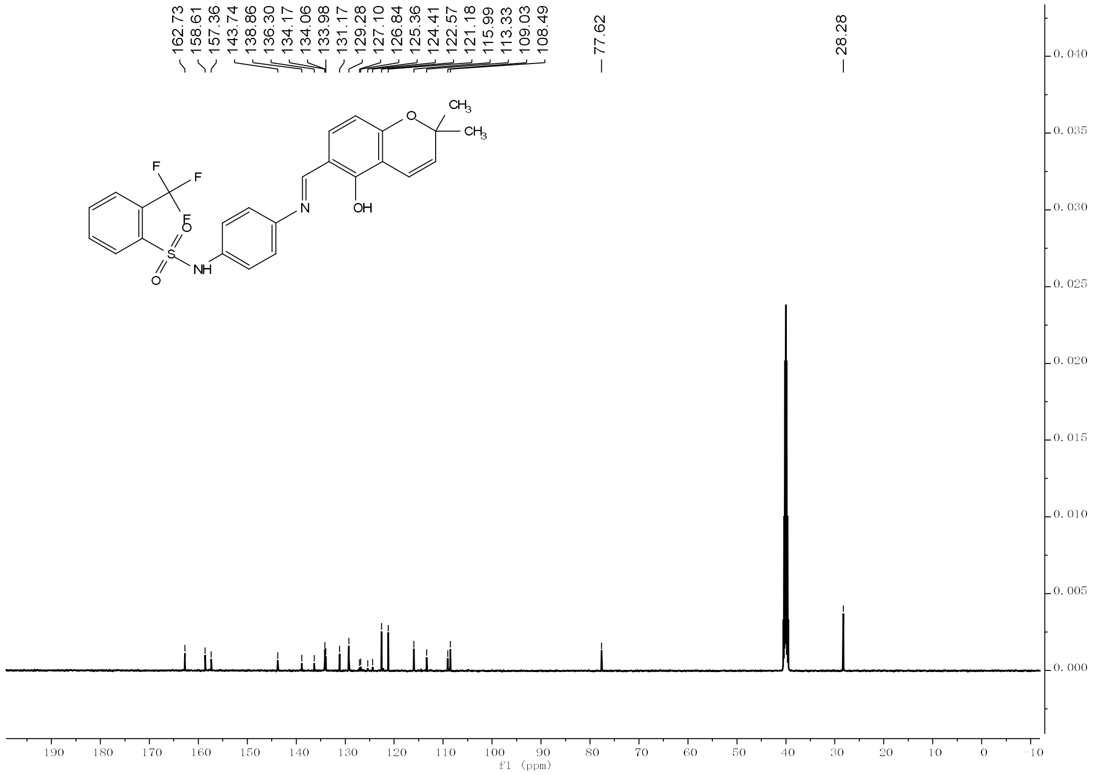

^{13}C NMR (126 MHz, DMSO-d_6) spectrum of compound **C22**.

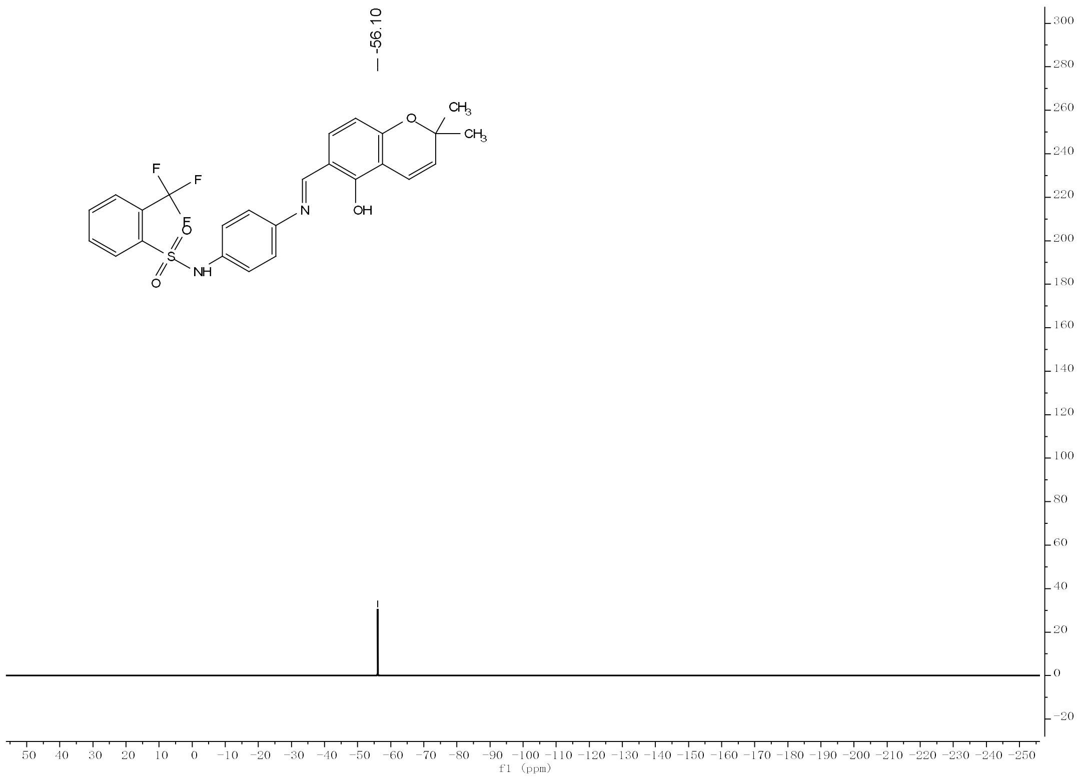

^{19}F NMR (500 MHz, DMSO-d_6) spectrum of compound **C22**.

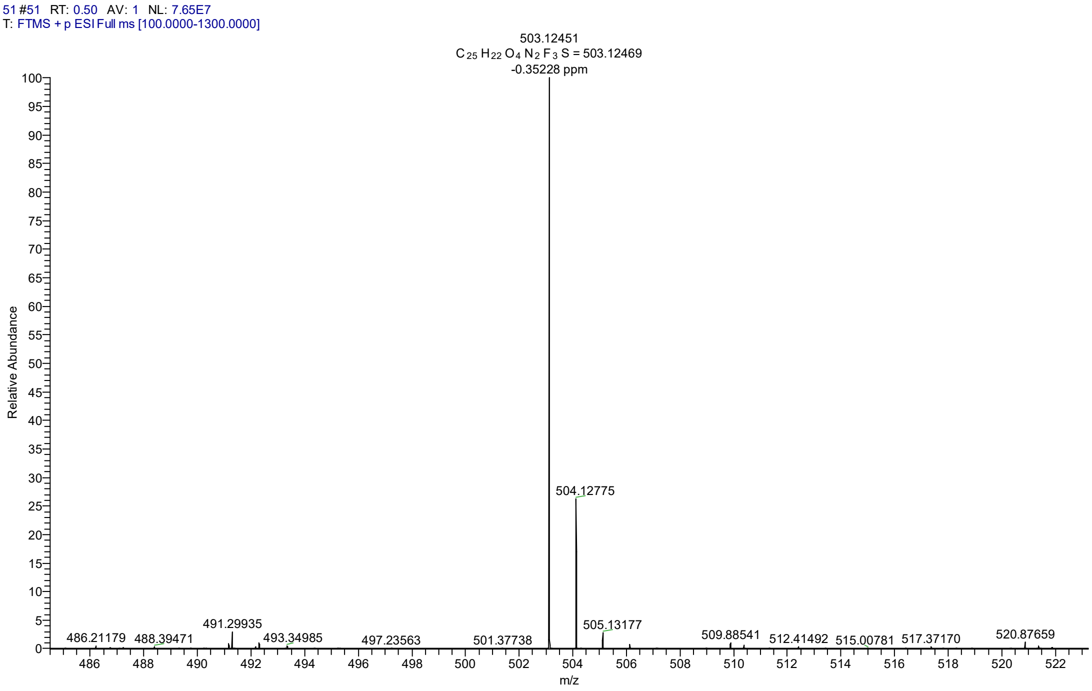

HRMS of compound **C22**.

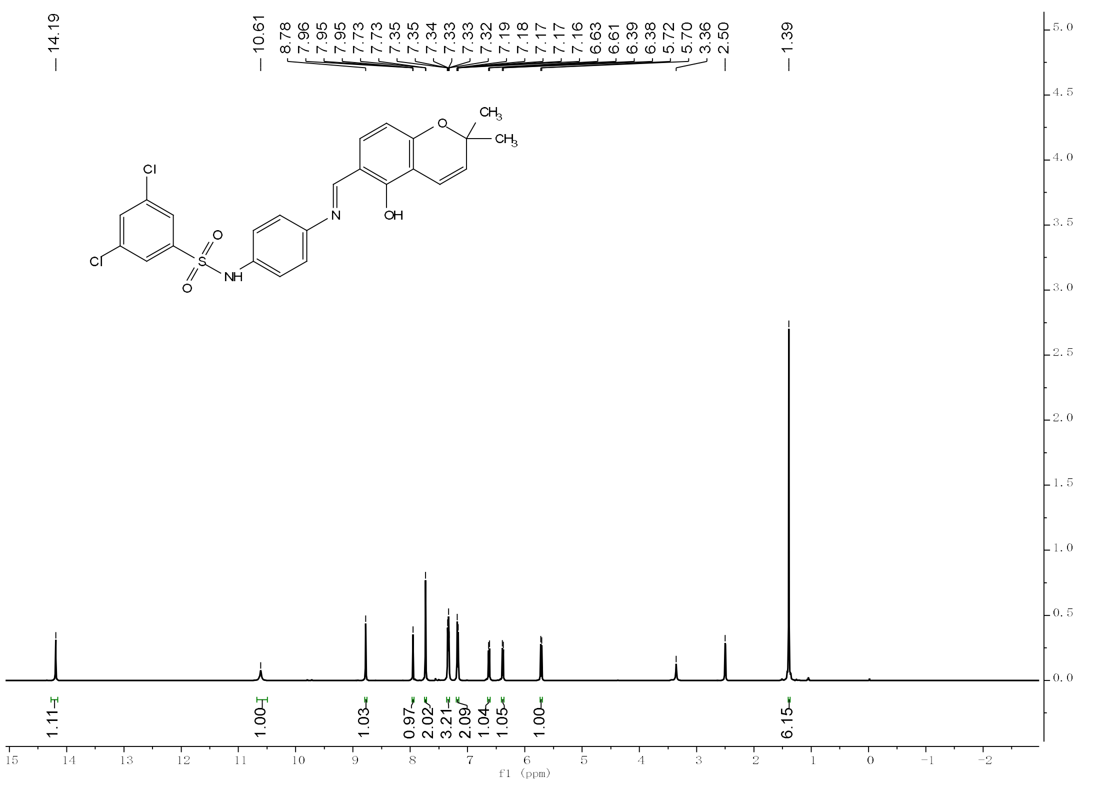

^{1}H NMR (500 MHz, DMSO-d_6) spectrum of compound **C23**.

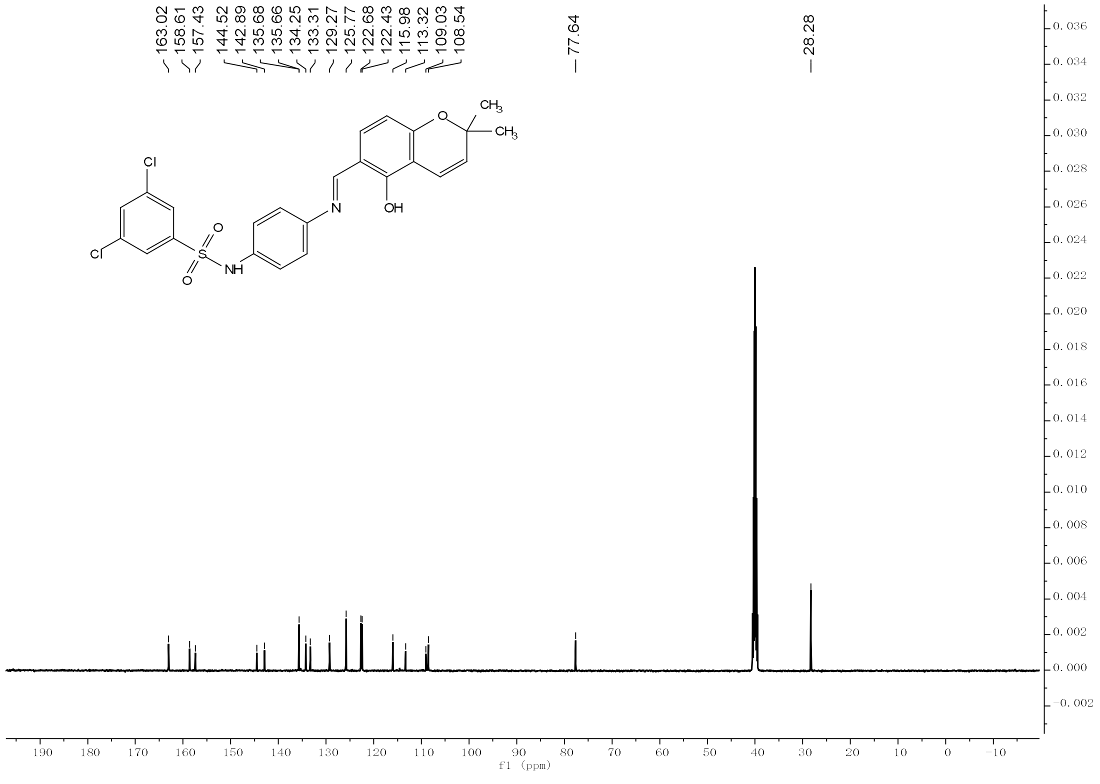

^{13}C NMR (126 MHz, DMSO-d_6) spectrum of compound **C23**.

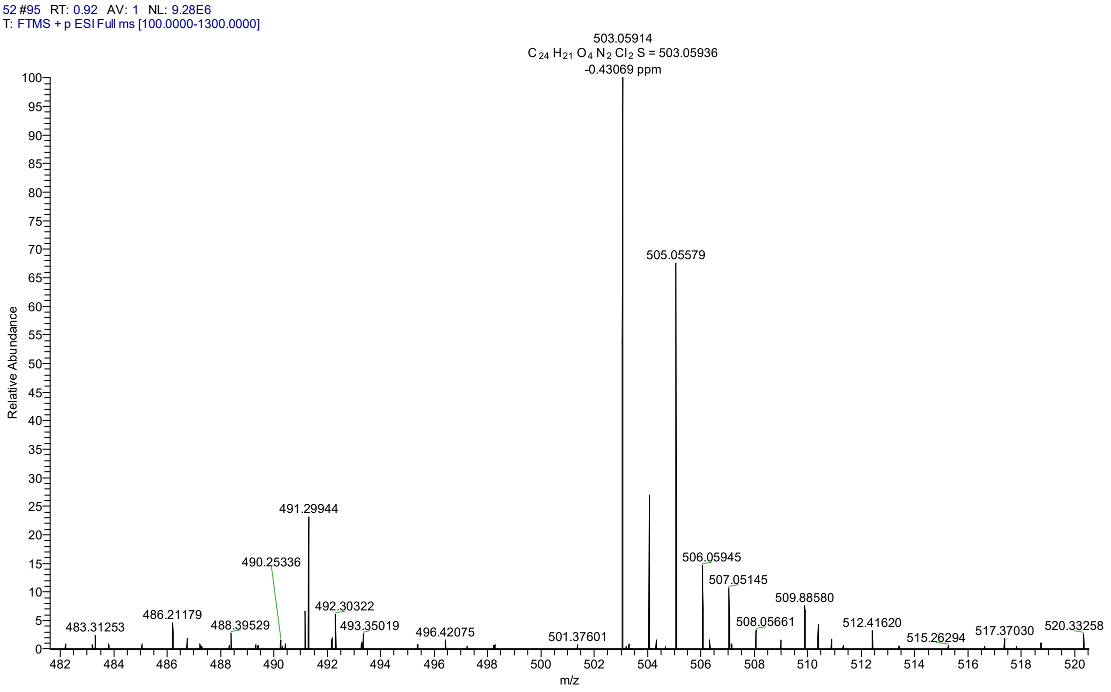

HRMS of compound **C23**.

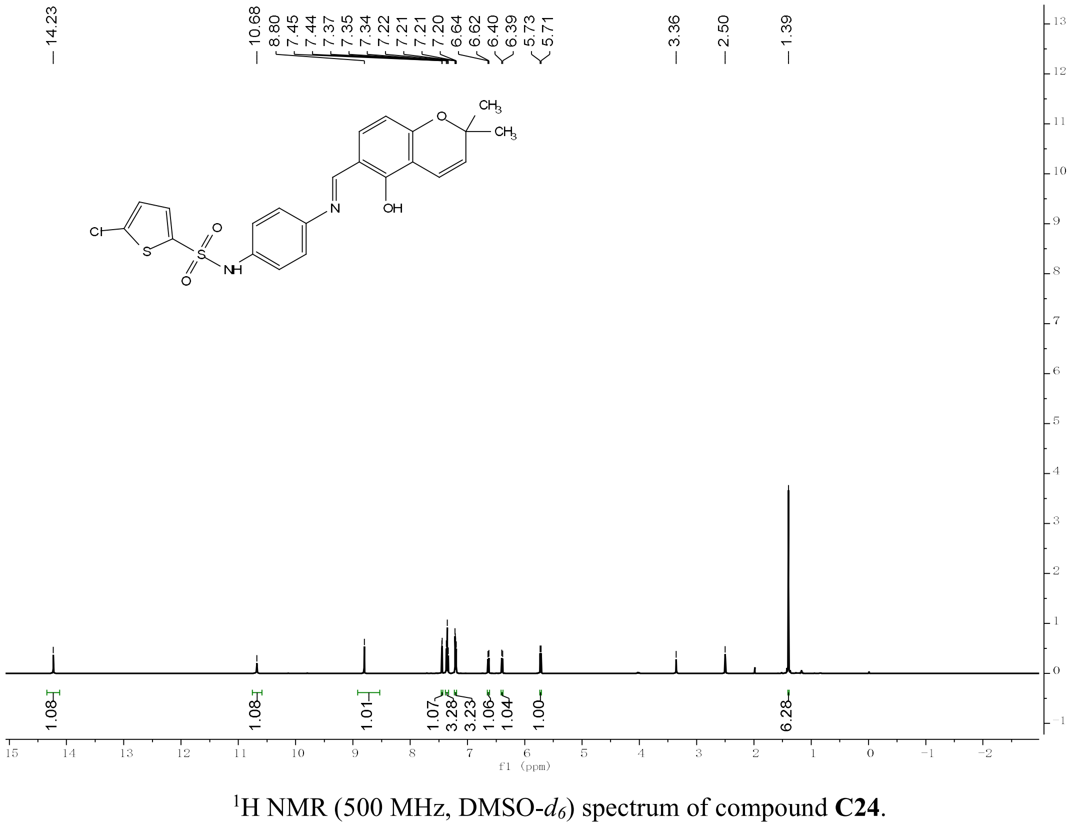

^{1}H NMR (500 MHz, DMSO-d_6) spectrum of compound **C24**.

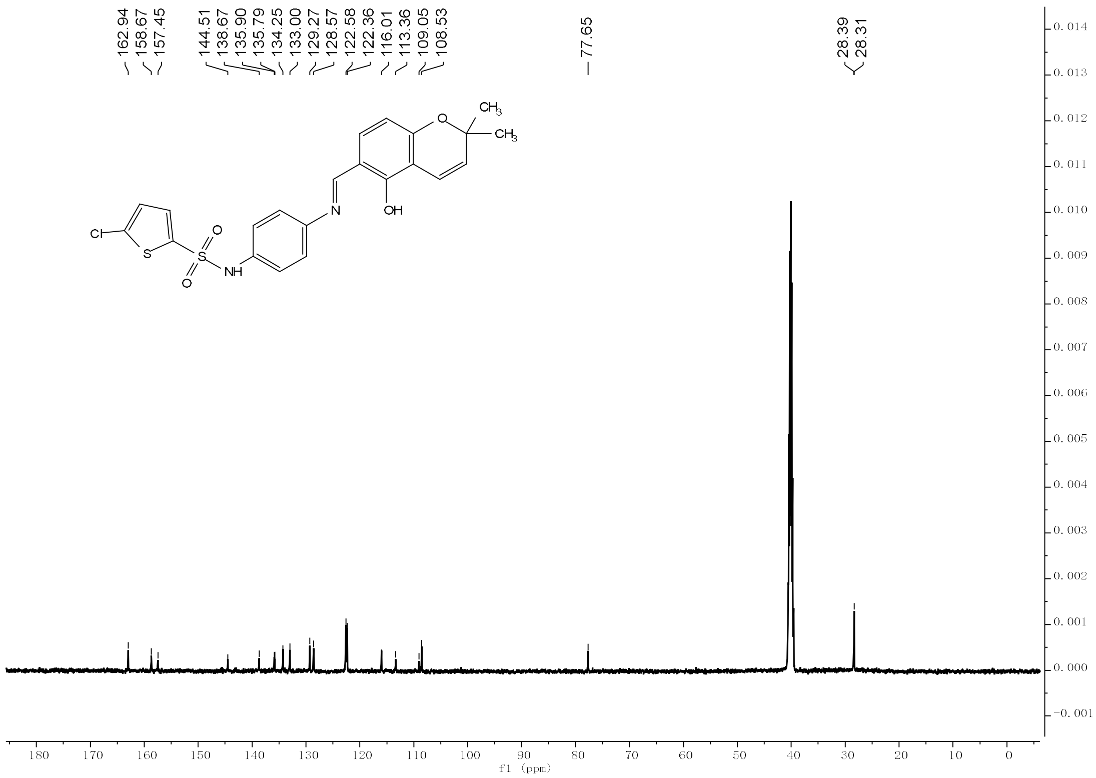

^{13}C NMR (126 MHz, DMSO-d_6) spectrum of compound **C24**.

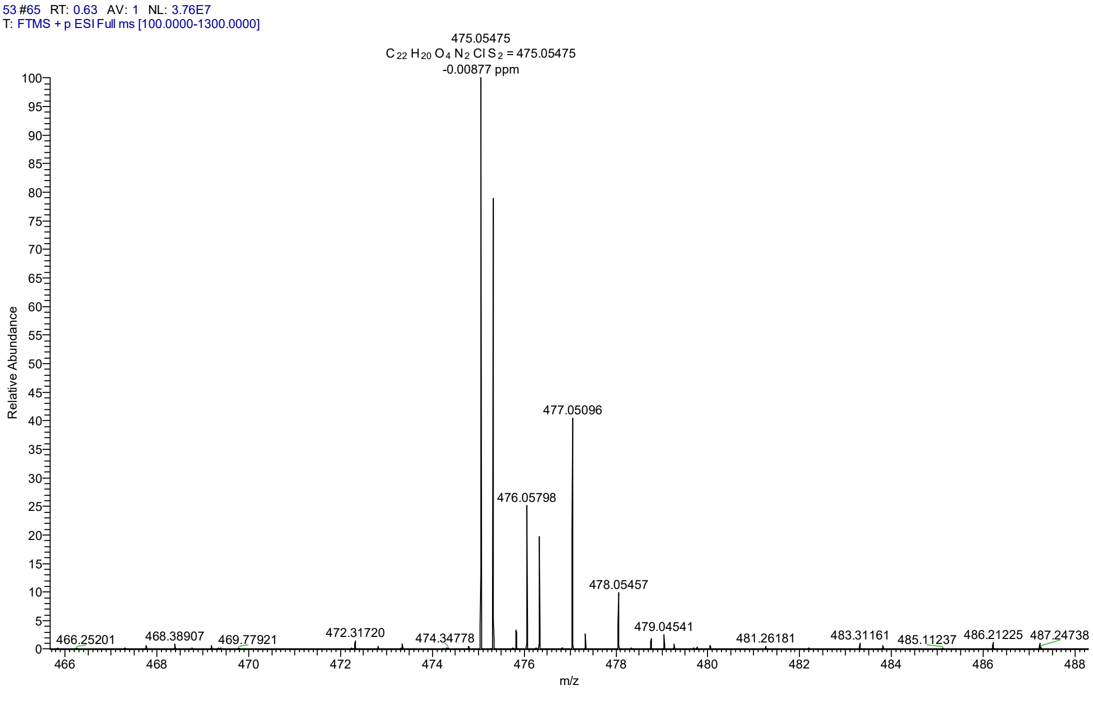

HRMS of compound **C24**.

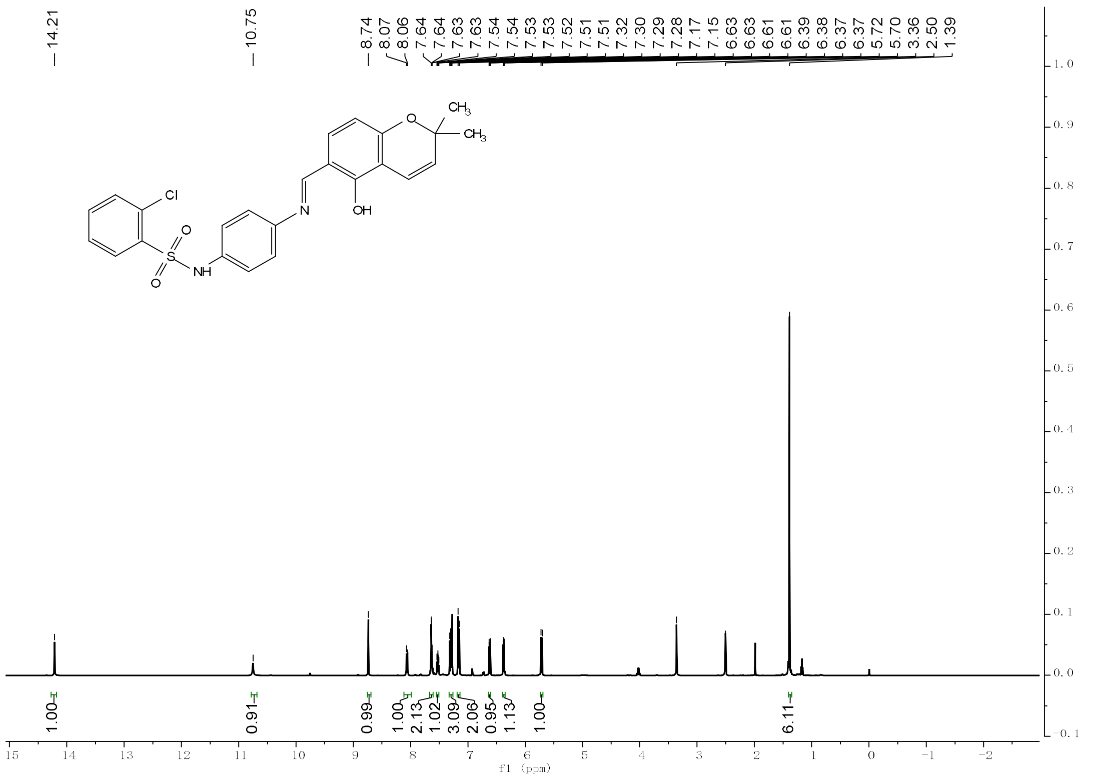

^{1}H NMR (500 MHz, DMSO-d_6) spectrum of compound **C25**.

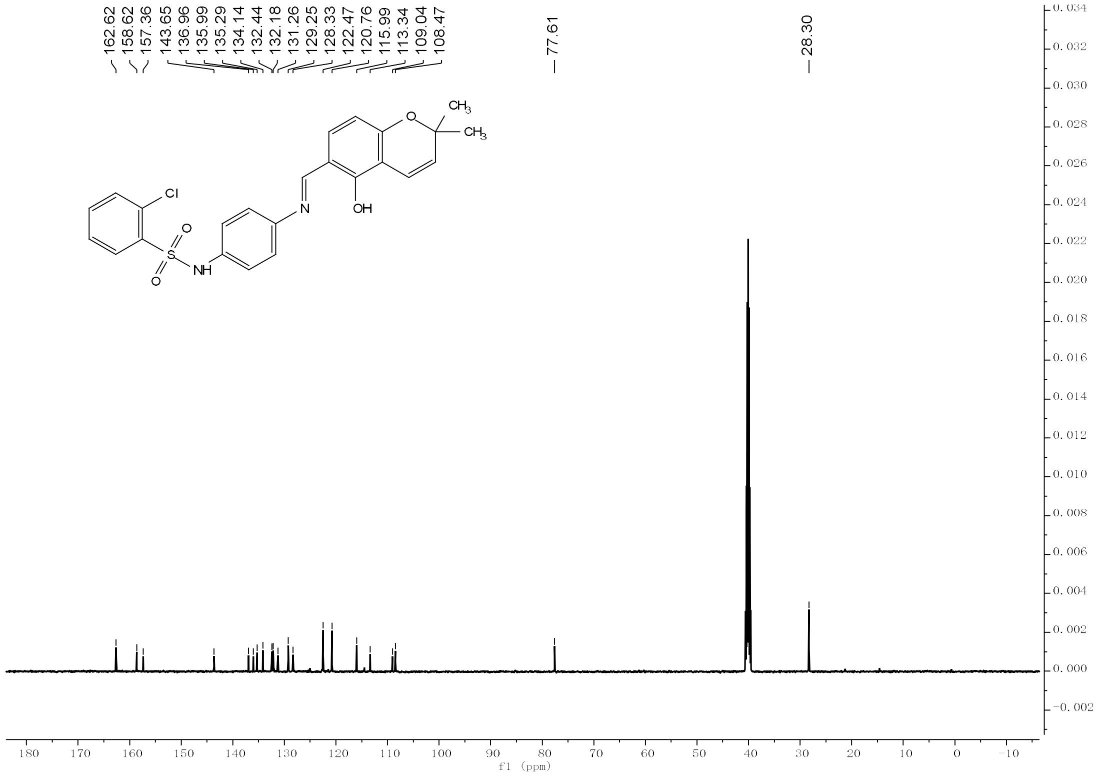

^{13}C NMR (126 MHz, DMSO-d_6) spectrum of compound **C25**.

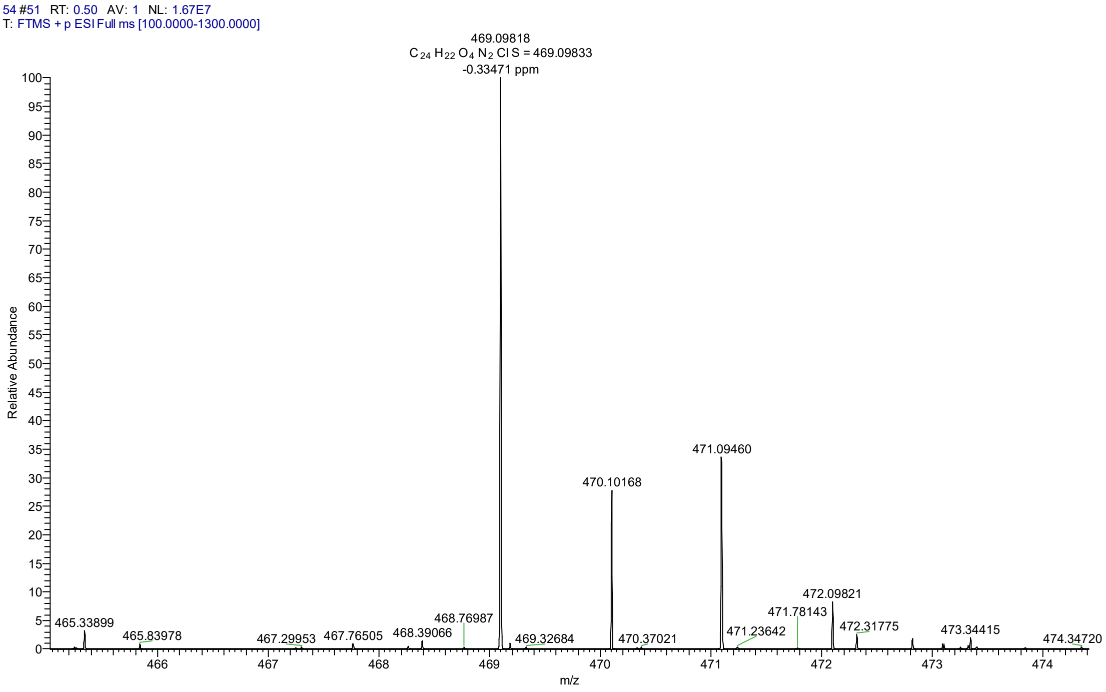

HRMS of compound **C25**.

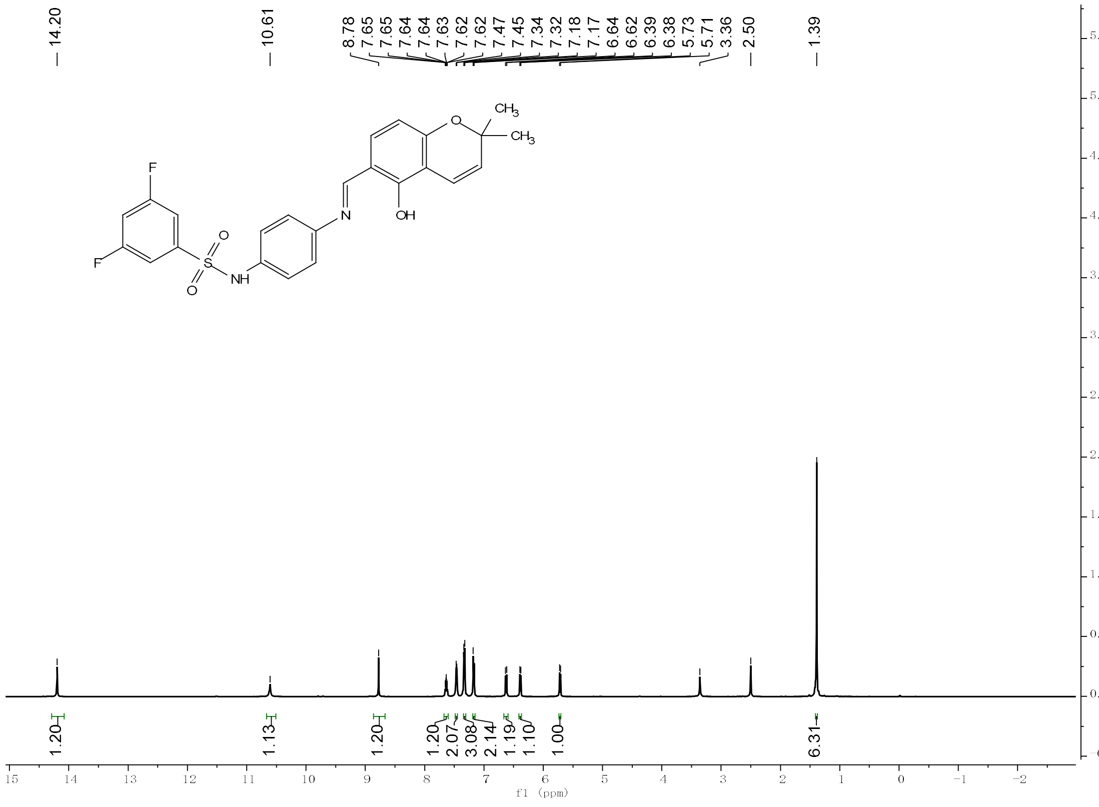

^{1}H NMR (500 MHz, DMSO-d_6) spectrum of compound **C26**.

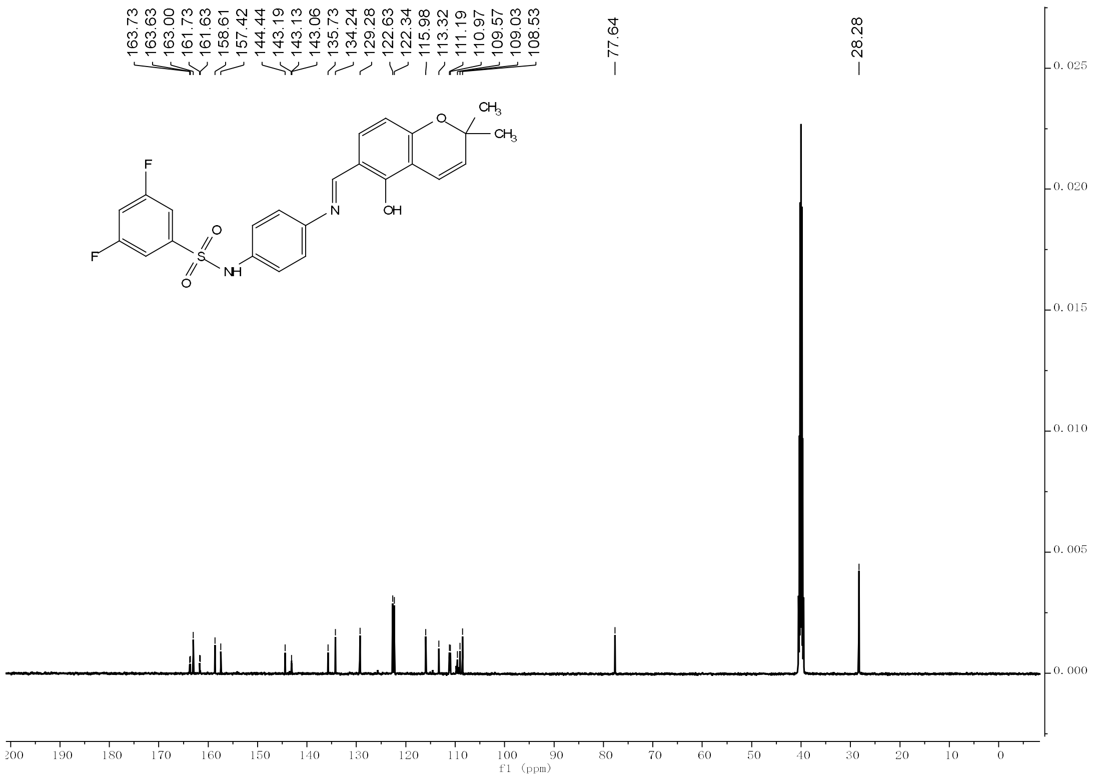

^{13}C NMR (126 MHz, DMSO-d_6) spectrum of compound **C26**.

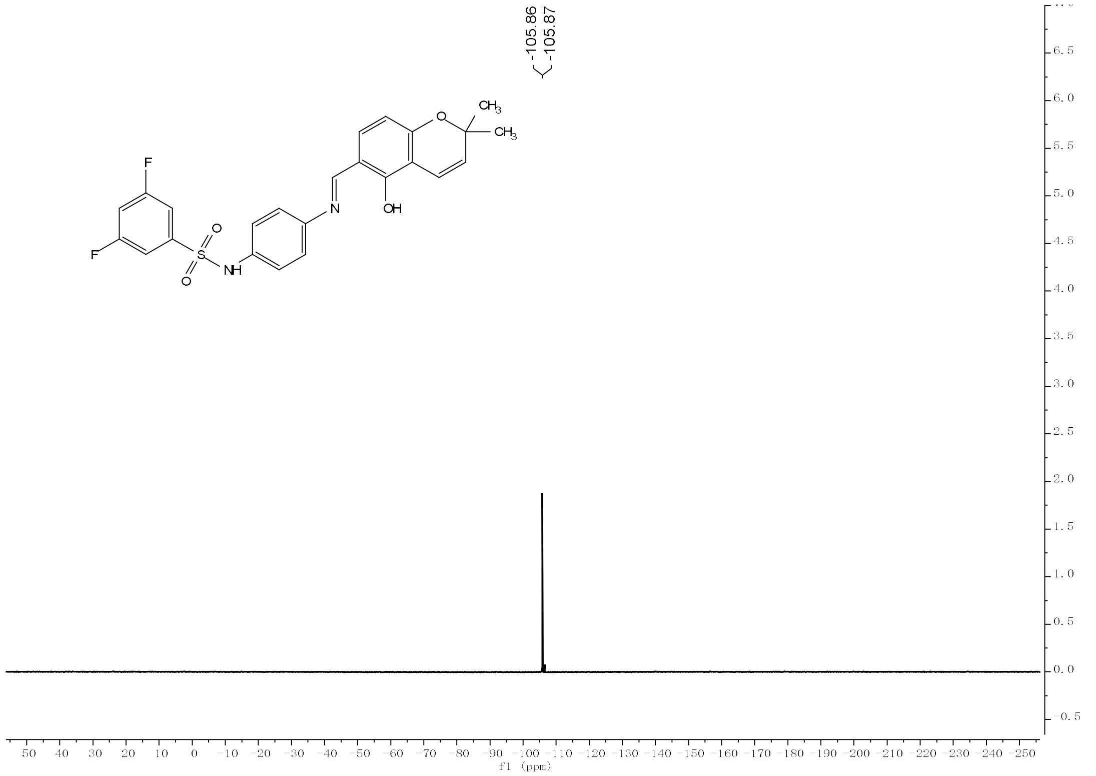

^{19}F NMR (471 MHz, DMSO-d_6) spectrum of compound **C26**.

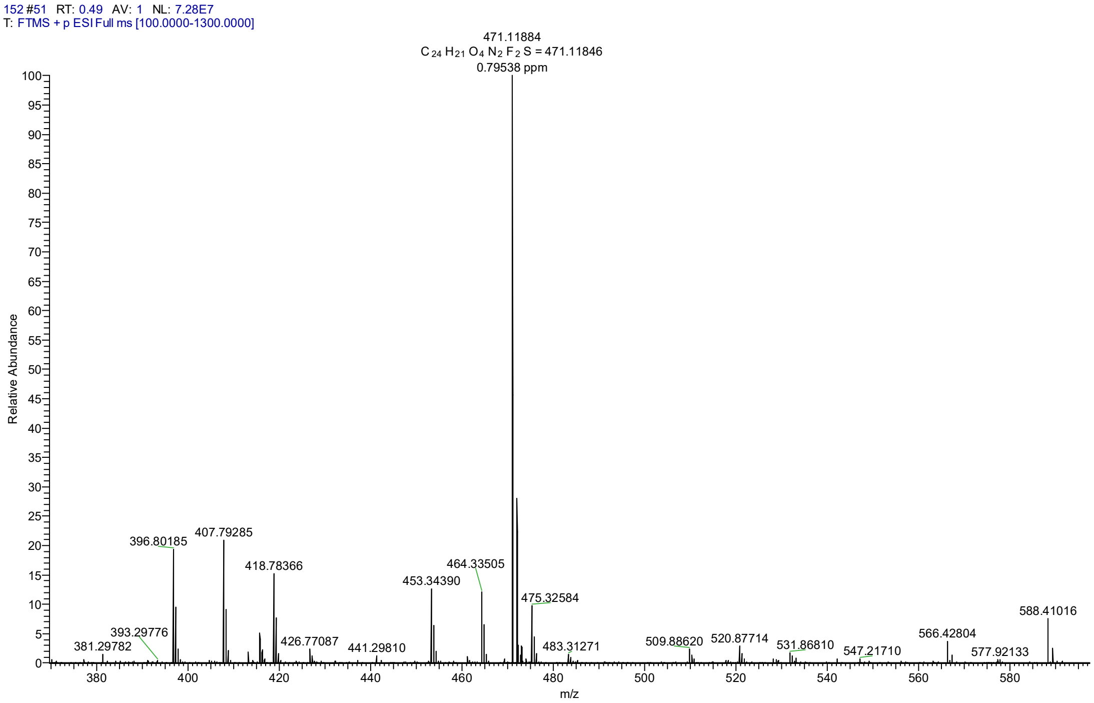

HRMS of compound **C26**.

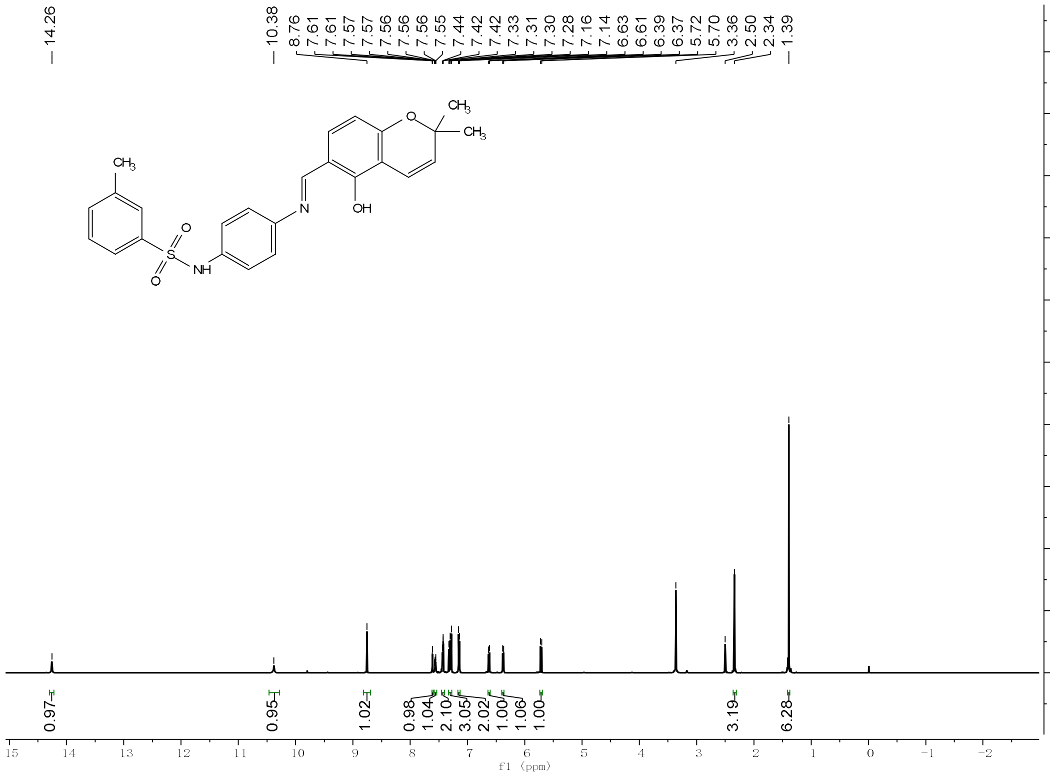

^{1}H NMR (500 MHz, DMSO-d_6) spectrum of compound **C27**.

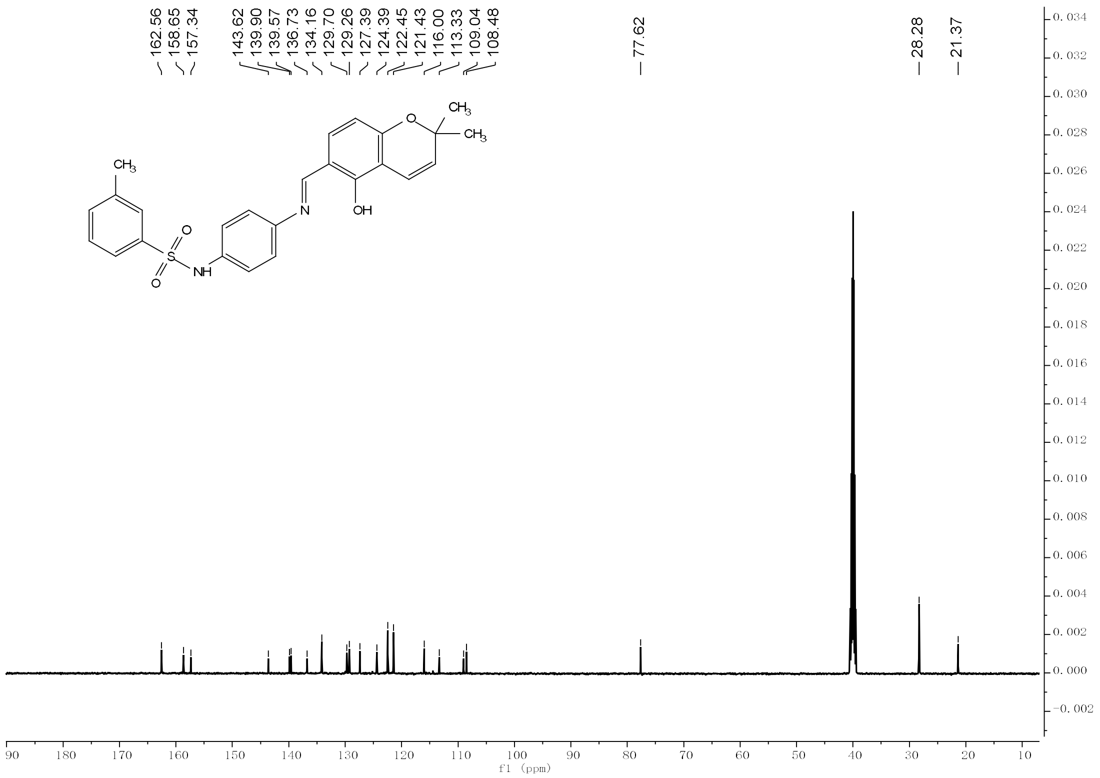

^{13}C NMR (126 MHz, DMSO-d_6) spectrum of compound **C27**.

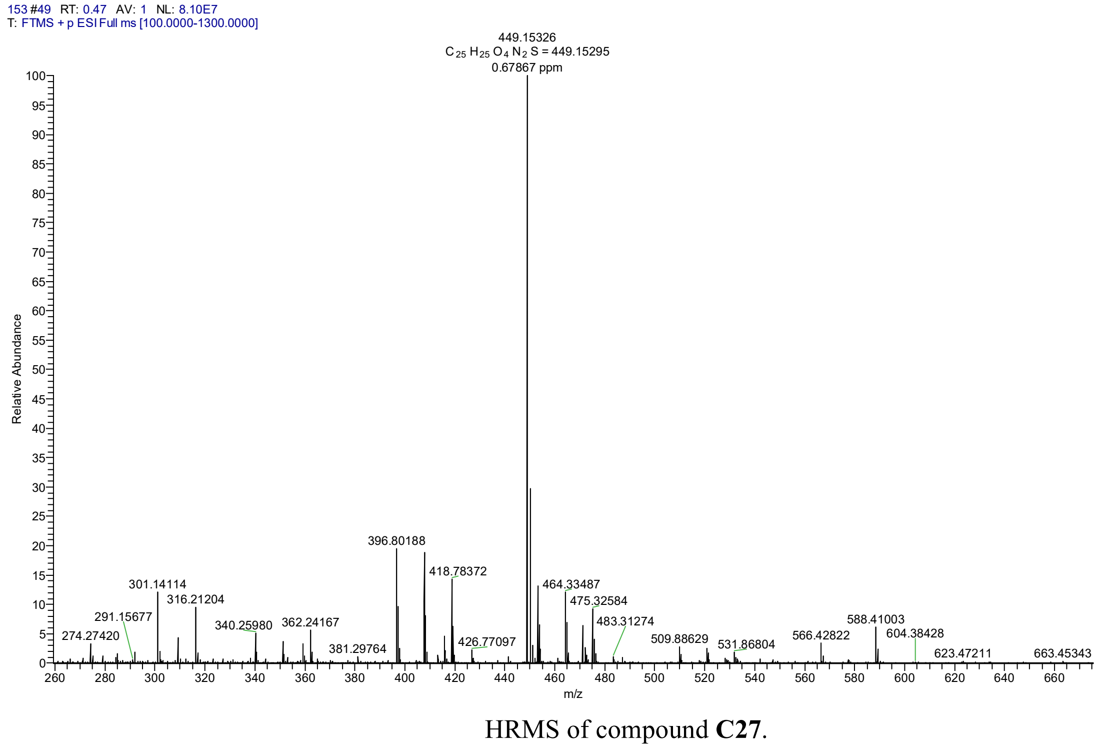

HRMS of compound **C27**.

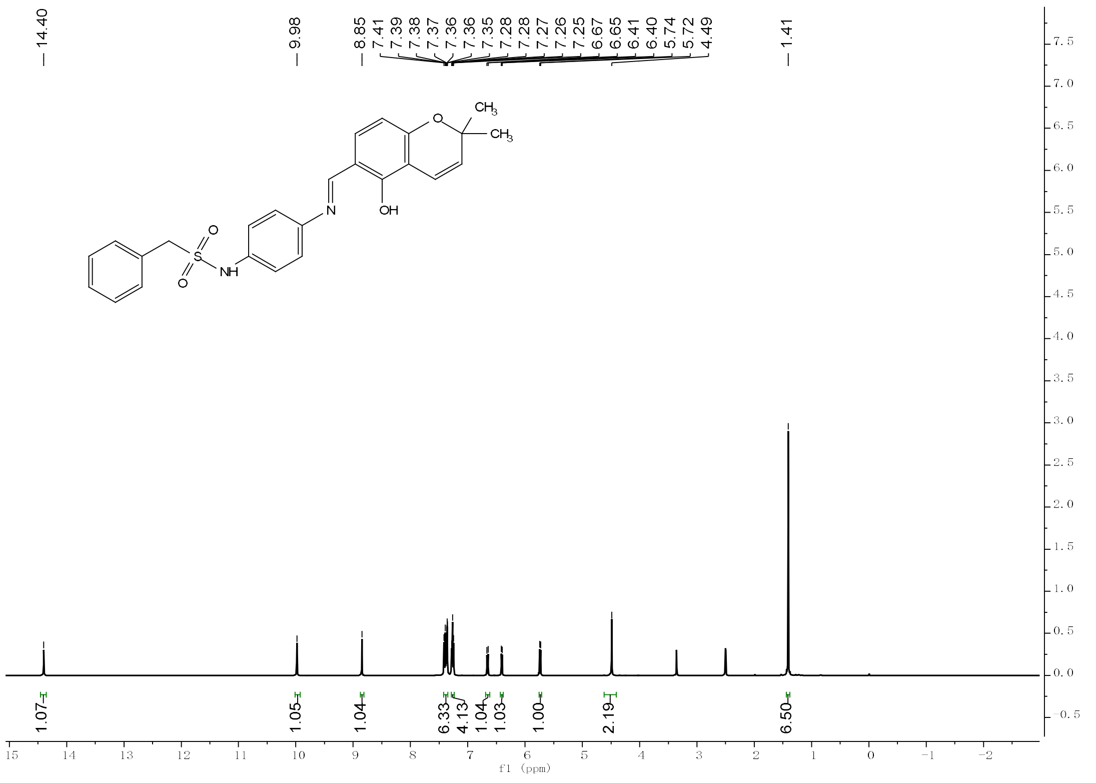

^{1}H NMR (500 MHz, DMSO-d_6) spectrum of compound **C28**.

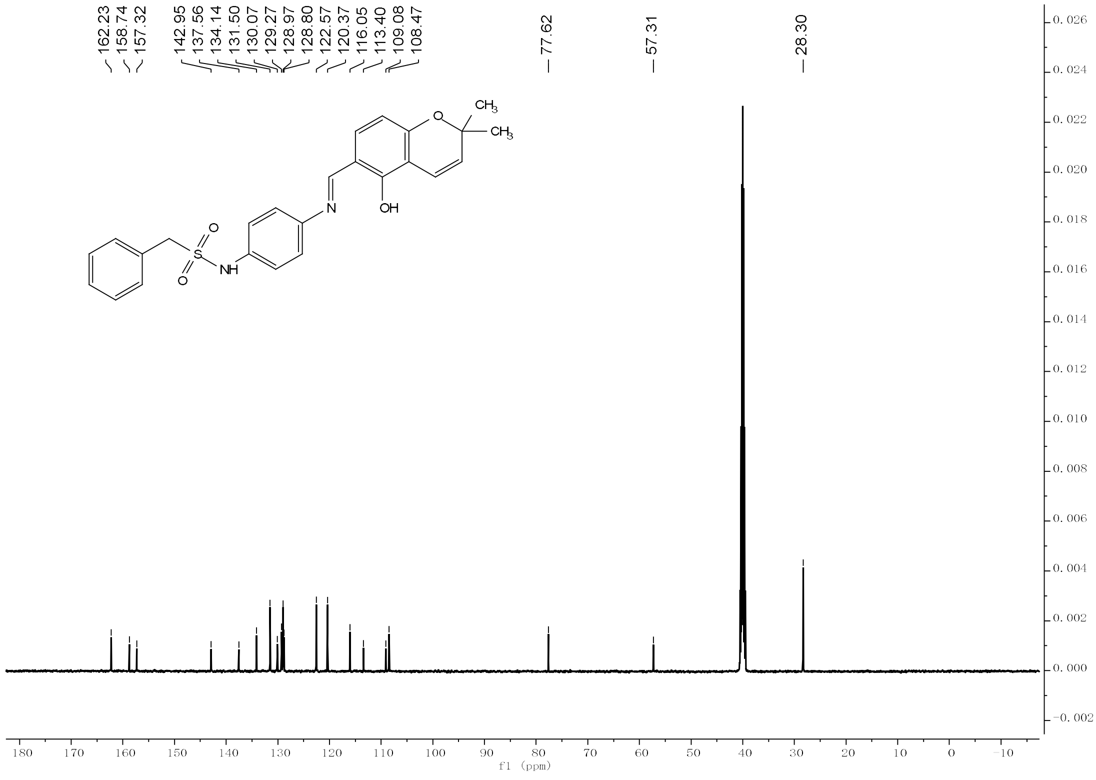

^{13}C NMR (126 MHz, DMSO-d_6) spectrum of compound **C28**.

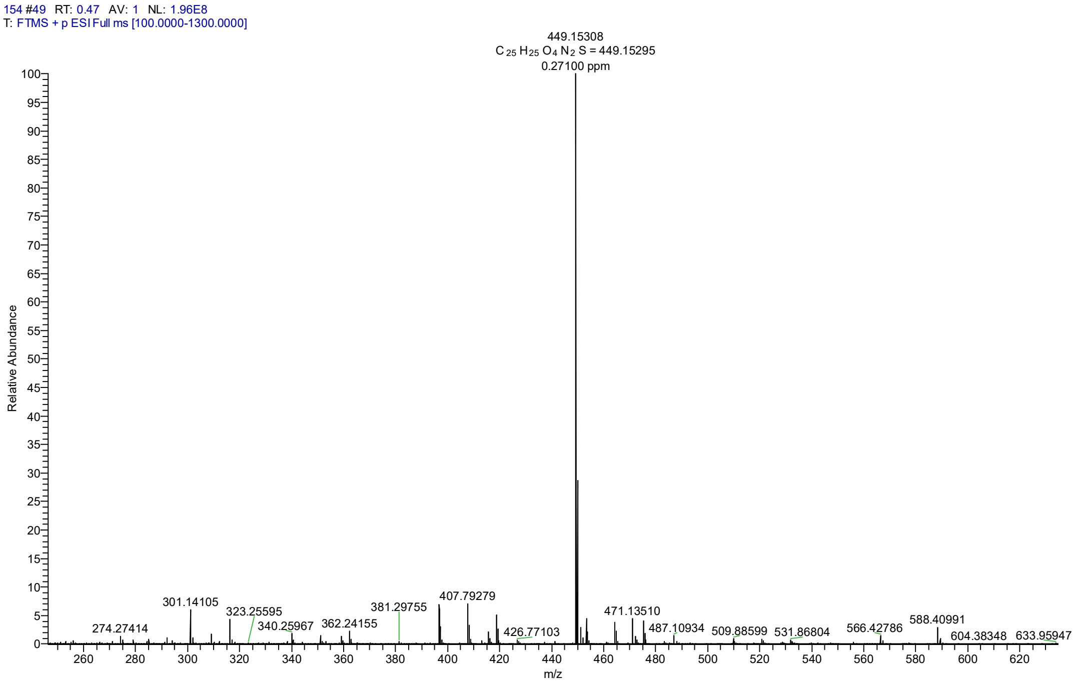

HRMS of compound **C28.**

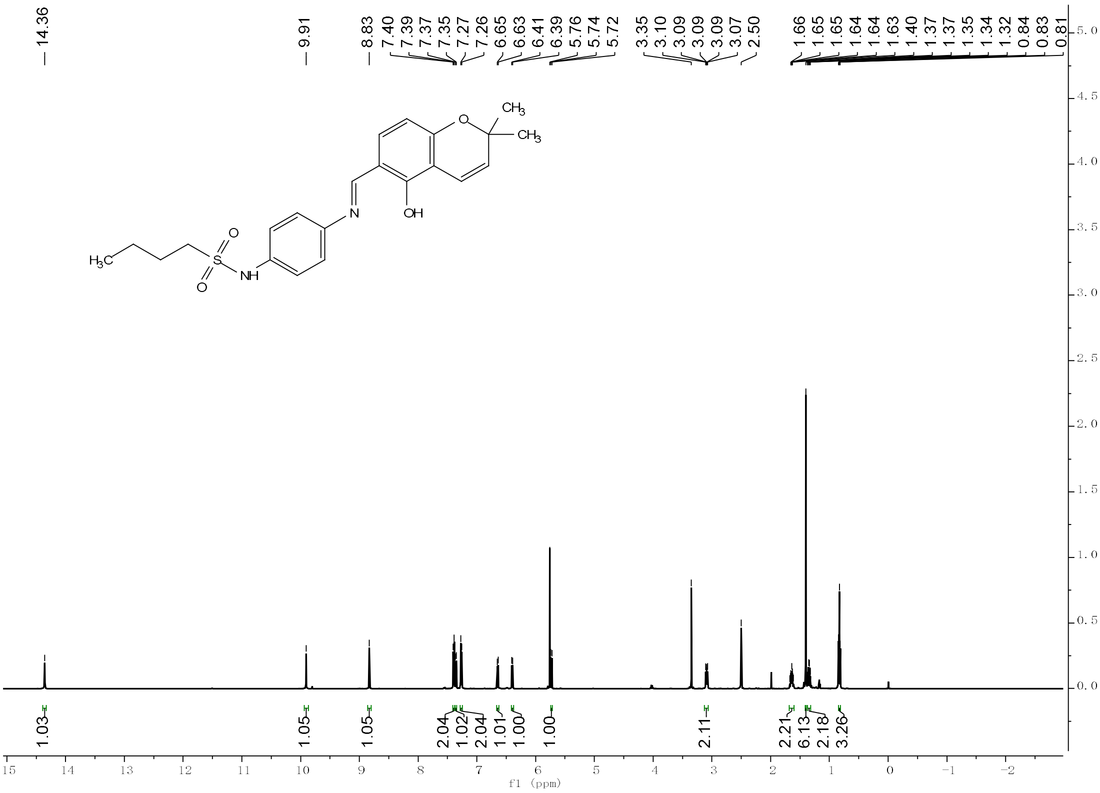

^{1}H NMR (500 MHz, DMSO-d_6) spectrum of compound **C29**.

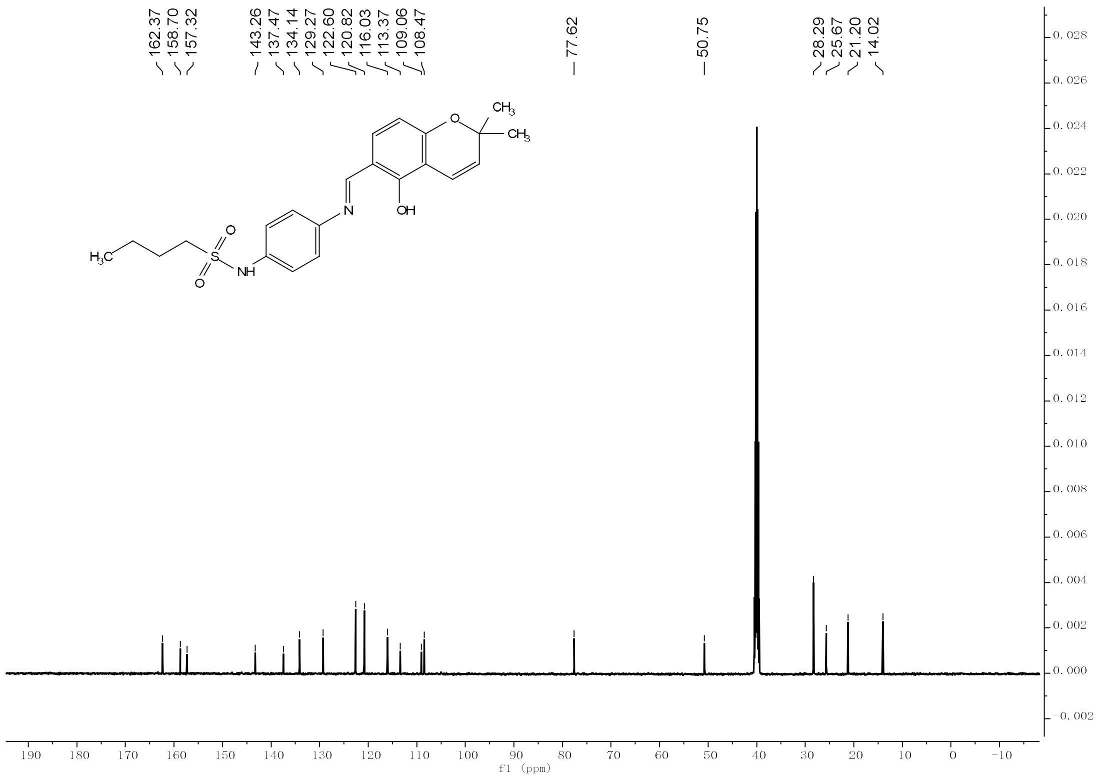

^{13}C NMR (126 MHz, DMSO-d_6) spectrum of compound **C29**.

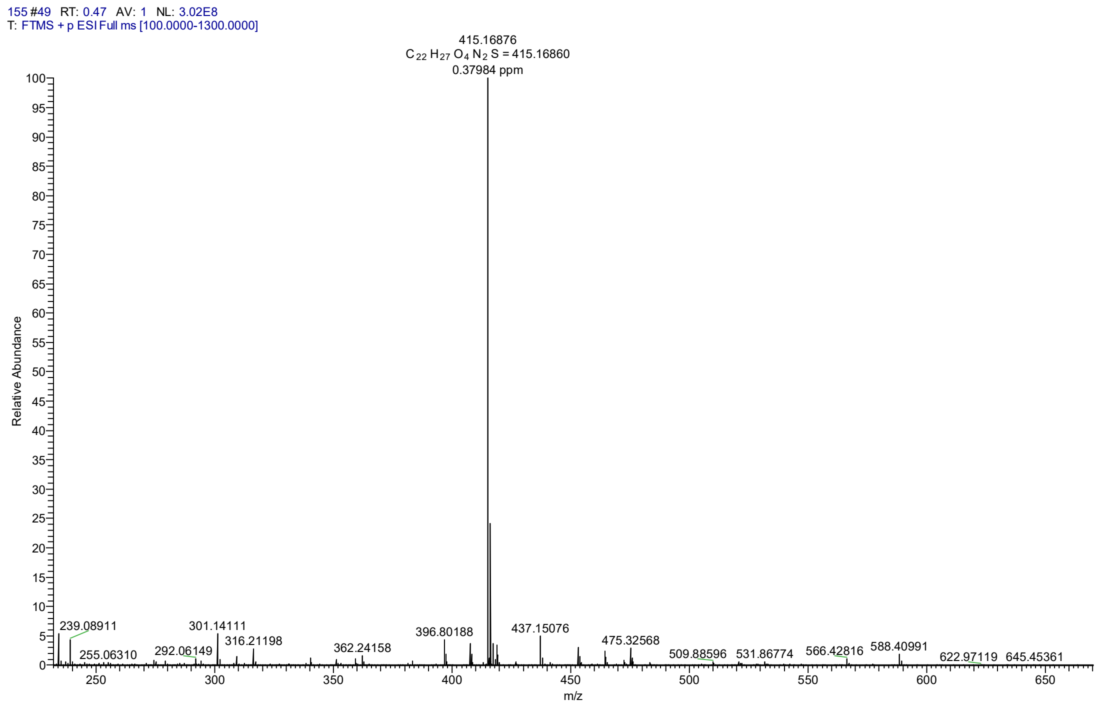

HRMS of compound **C29**.

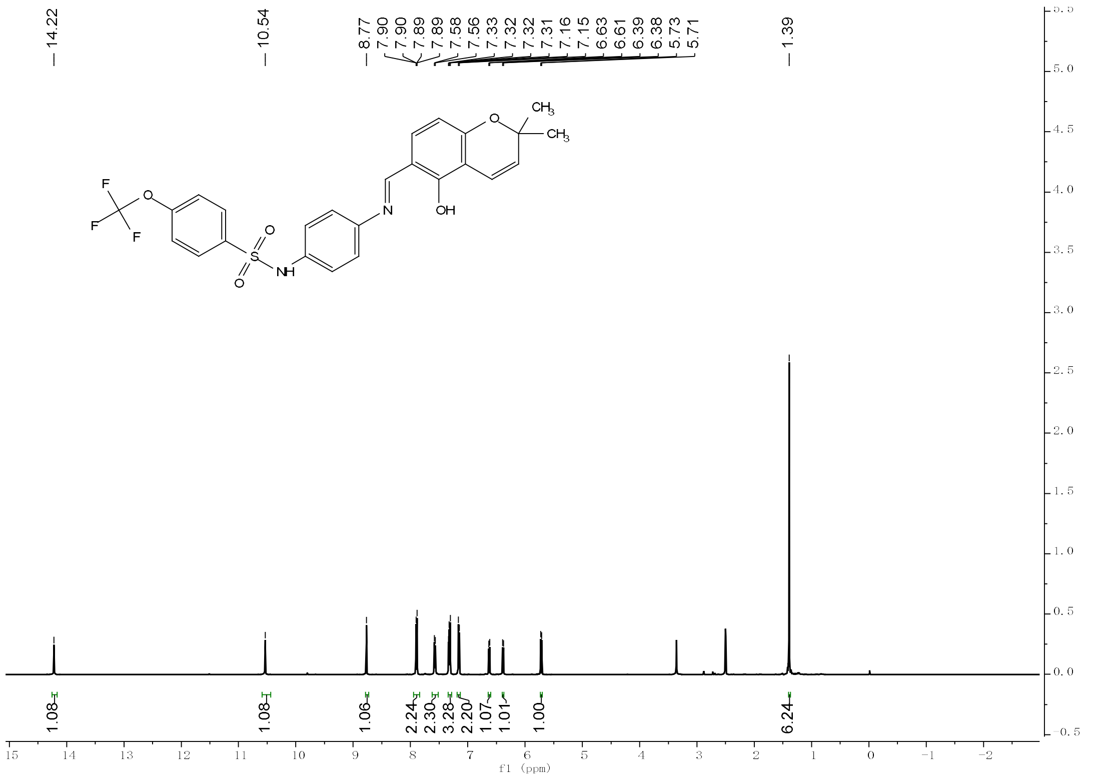

^{1}H NMR (500 MHz, DMSO-d_6) spectrum of compound **C30**.

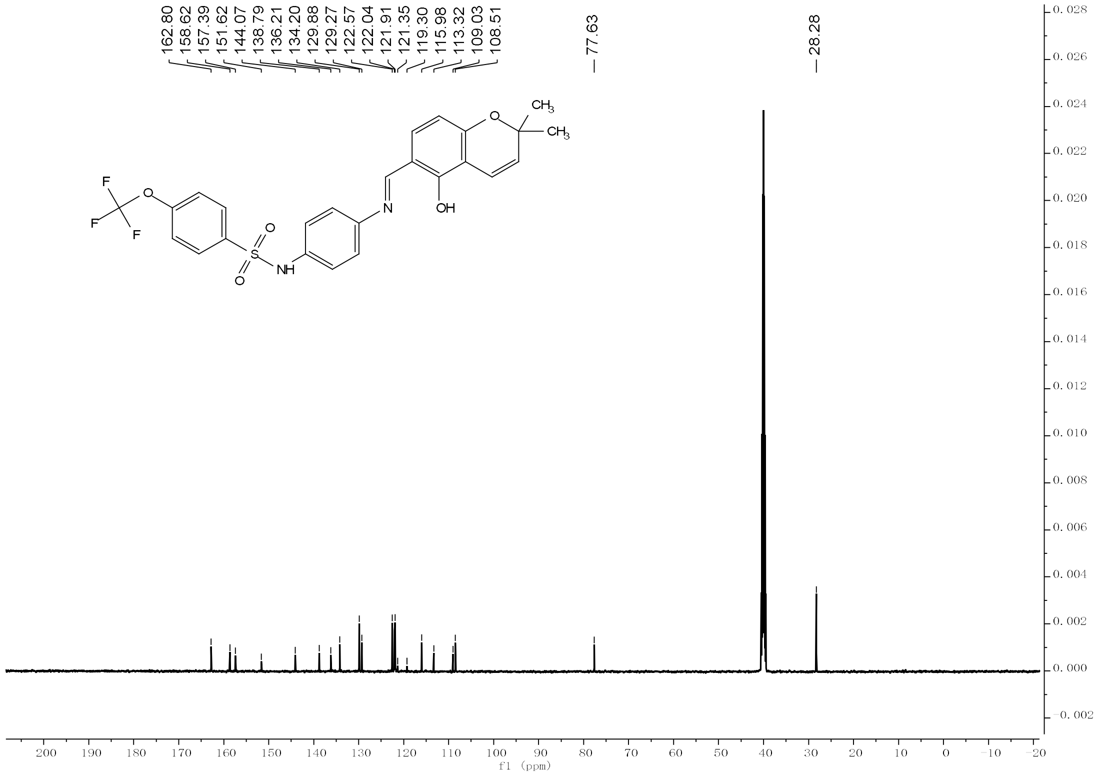

^{13}C NMR (126 MHz, DMSO-d_6) spectrum of compound **C30**.

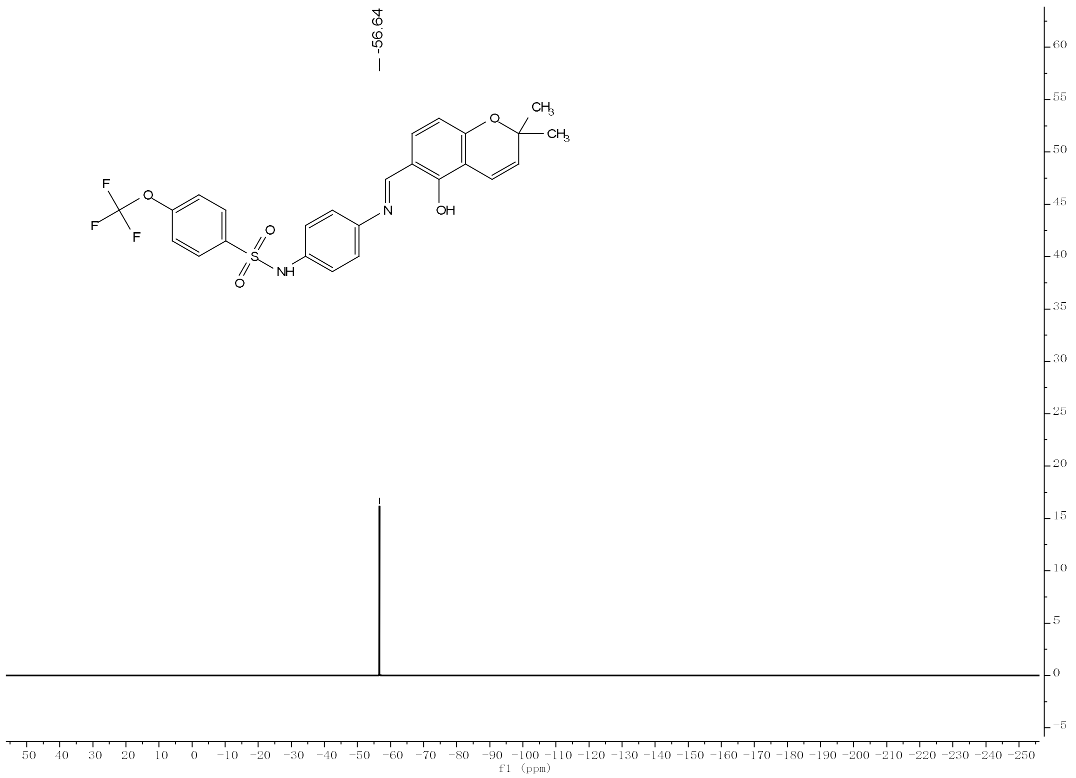

^{19}F NMR (471 MHz, DMSO-d_6) spectrum of compound C**30**.

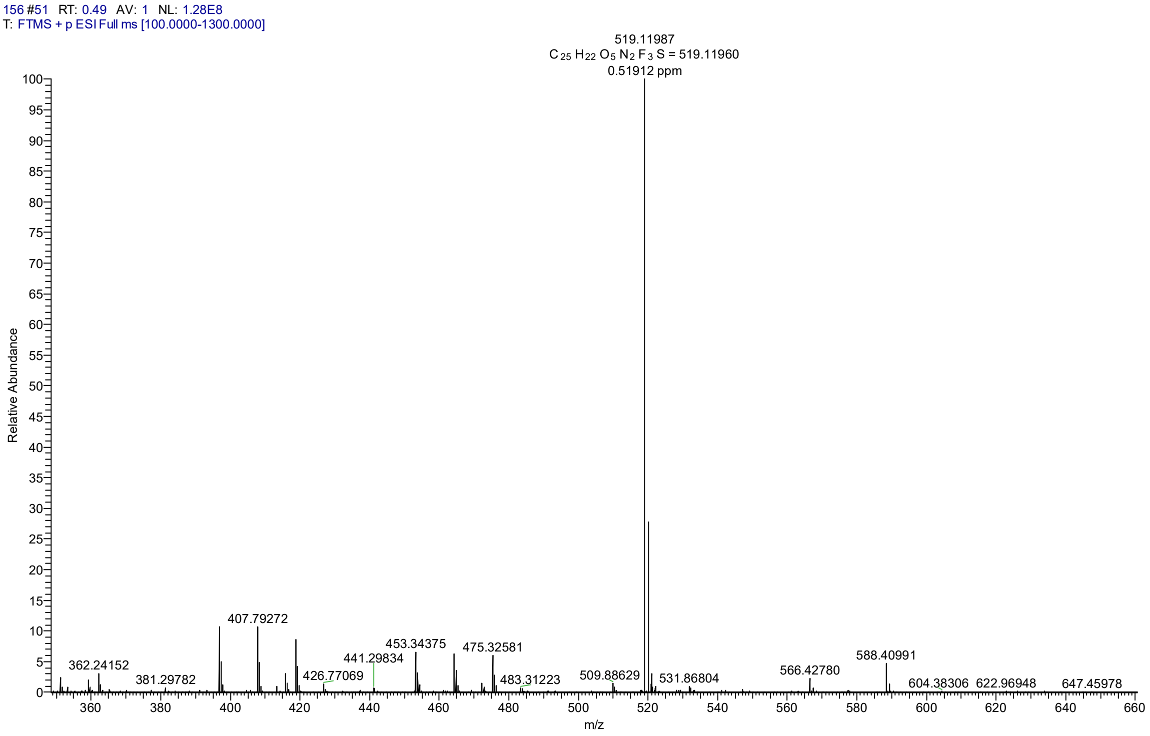

HRMS of compound **C30**.

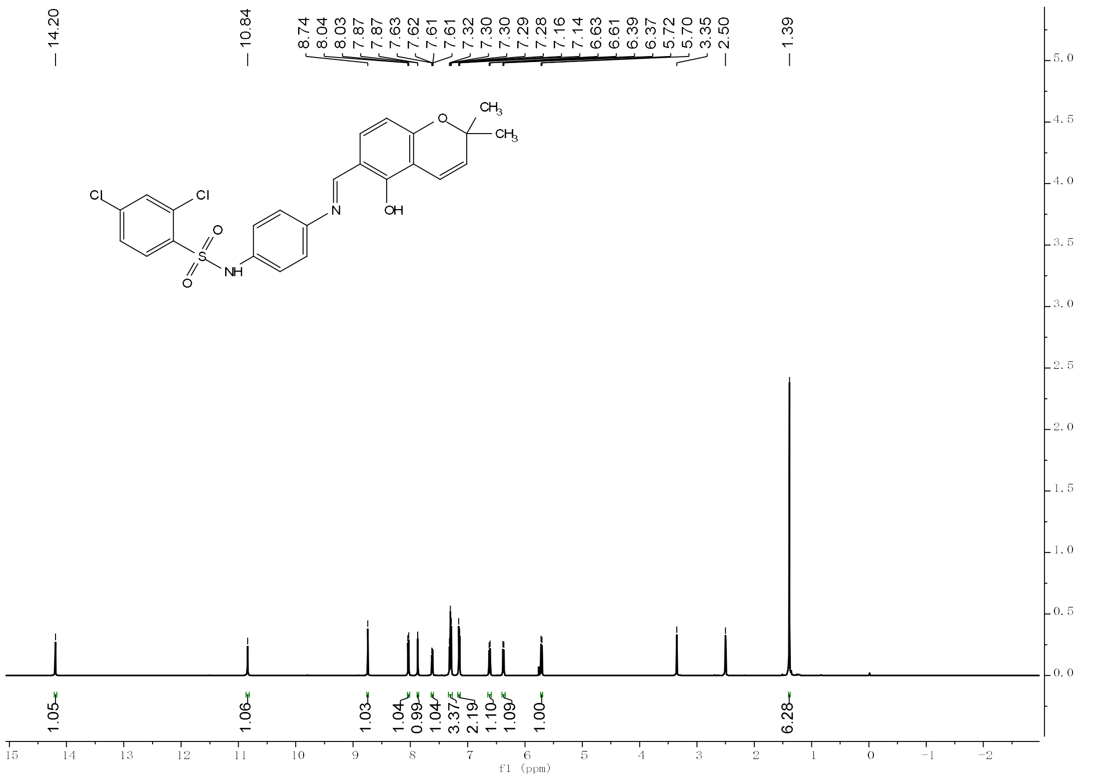

^{1}H NMR (500 MHz, DMSO-d_6) spectrum of compound **C31**.

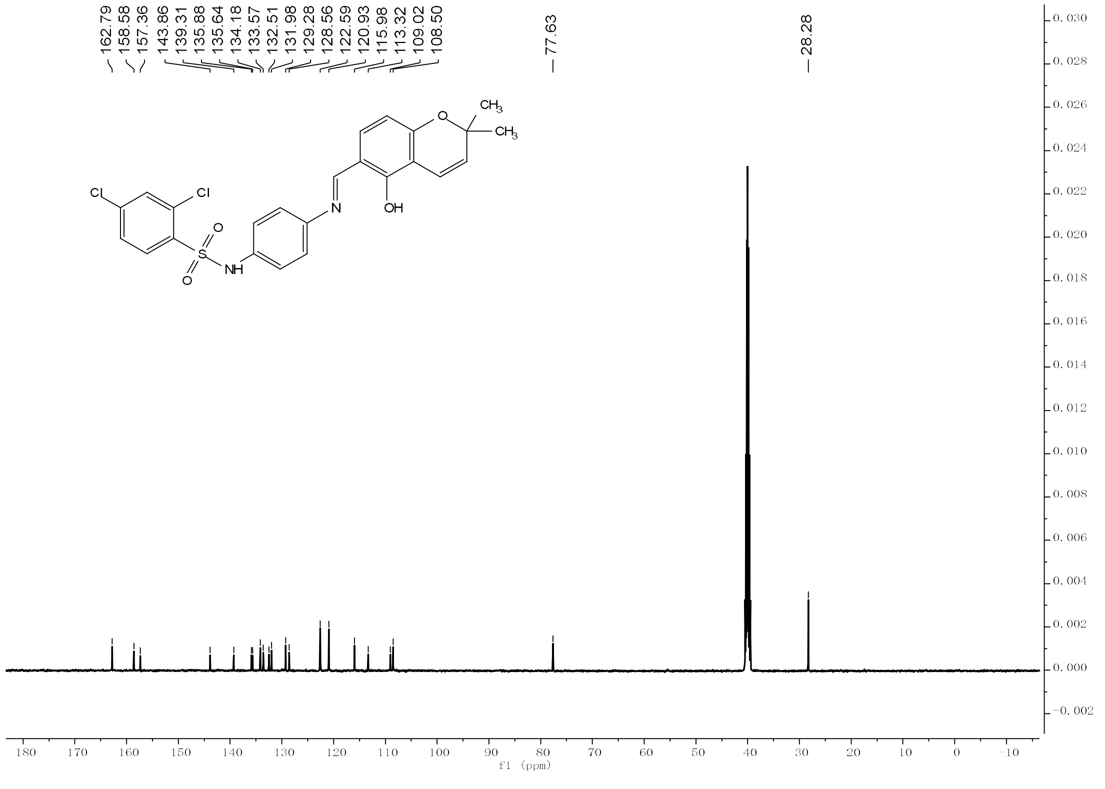

^{13}C NMR (126 MHz, DMSO-d_6) spectrum of compound **C31**.

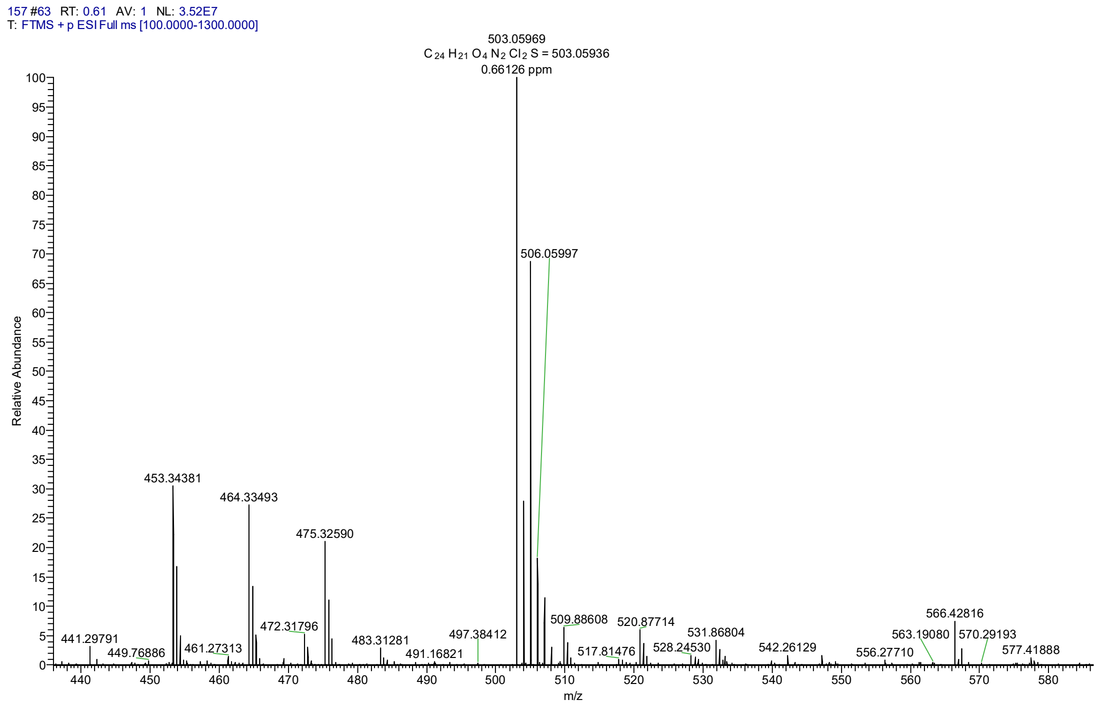

HRMS of compound **C31**

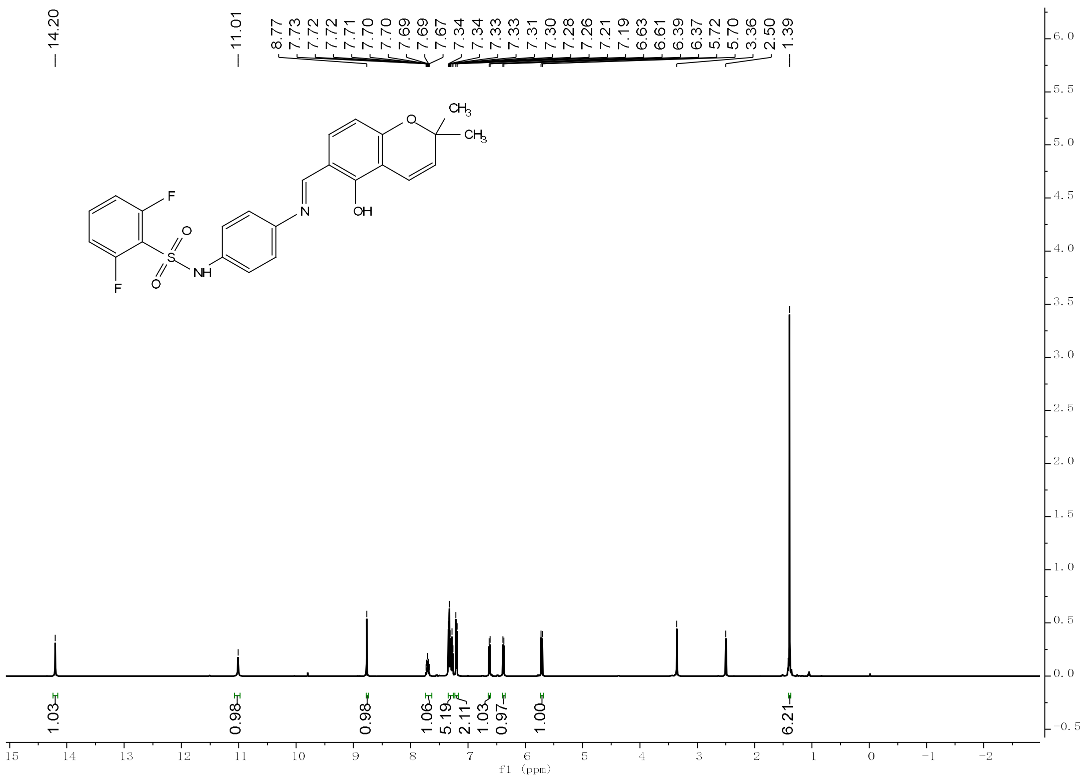

^{1}H NMR (500 MHz, DMSO-d_6) spectrum of compound **C32**.

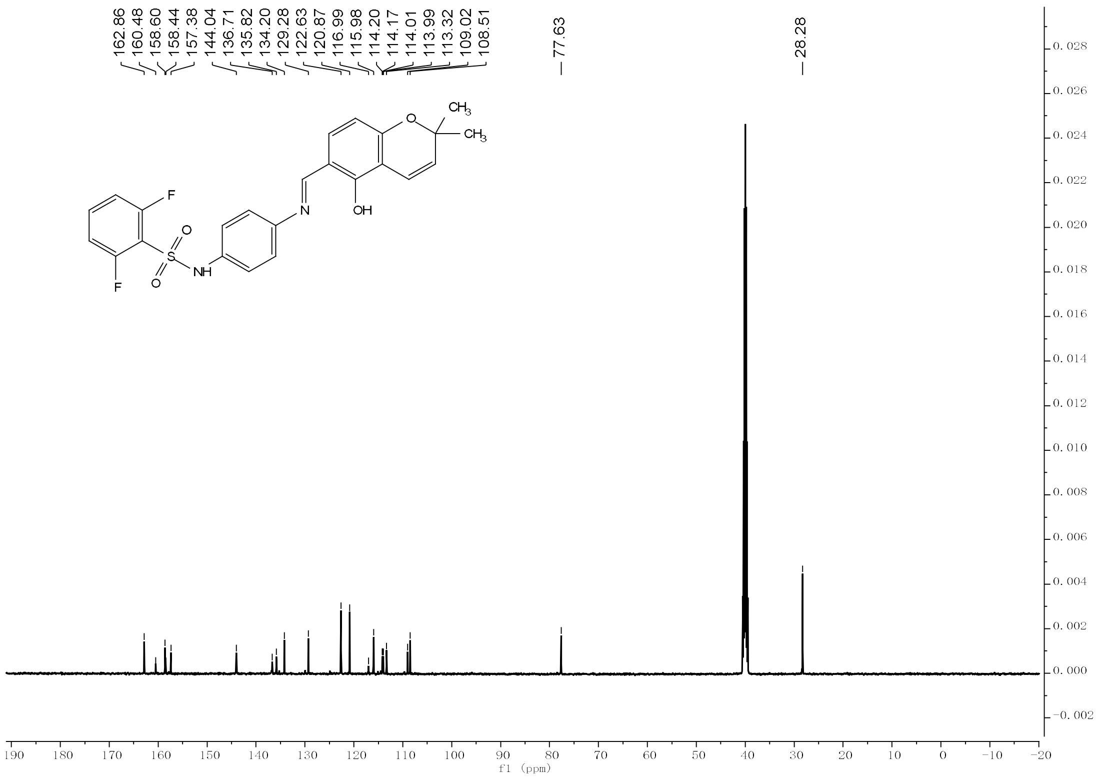

^{13}C NMR (126 MHz, DMSO-d_6) spectrum of compound **C32**.

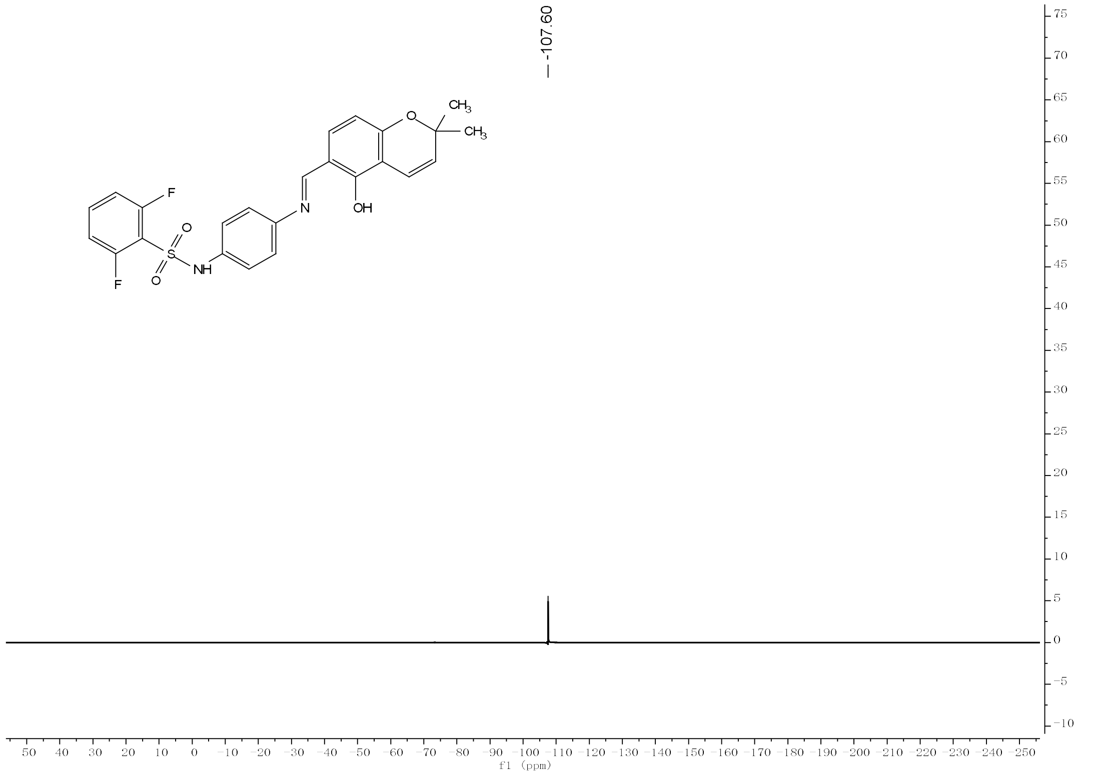

^{19}F NMR (471 MHz, DMSO-d_6) spectrum of compound C**32**.

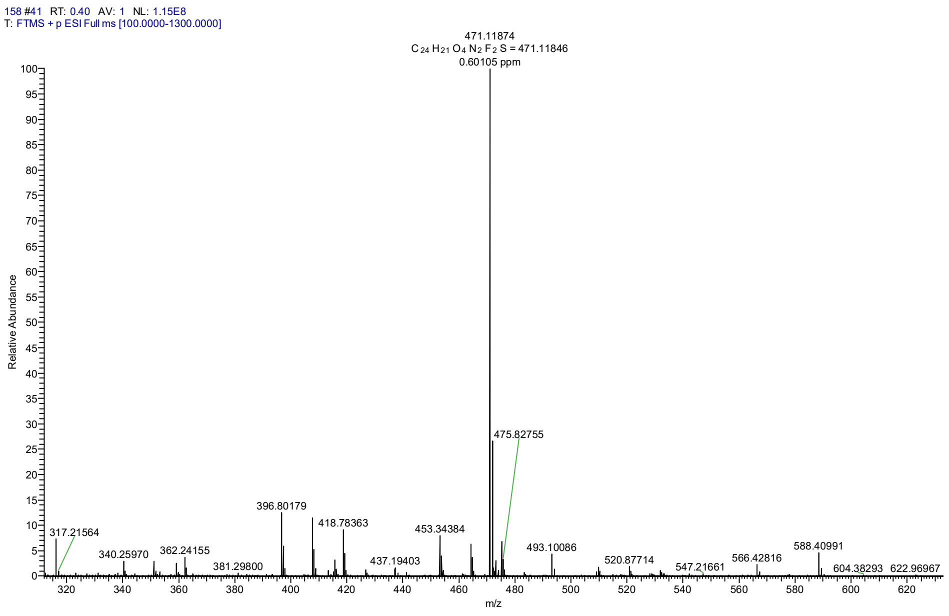

HRMS of compound **C32**.

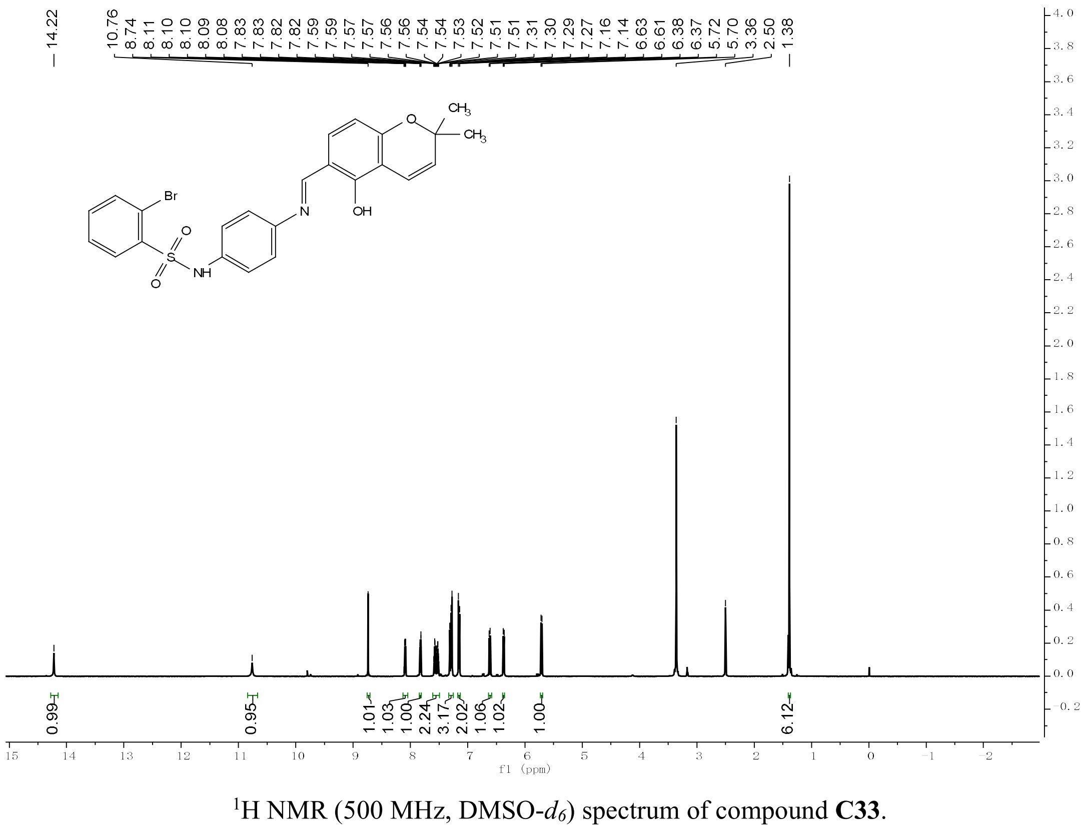

^{1}H NMR (500 MHz, DMSO-d_6) spectrum of compound **C33**.

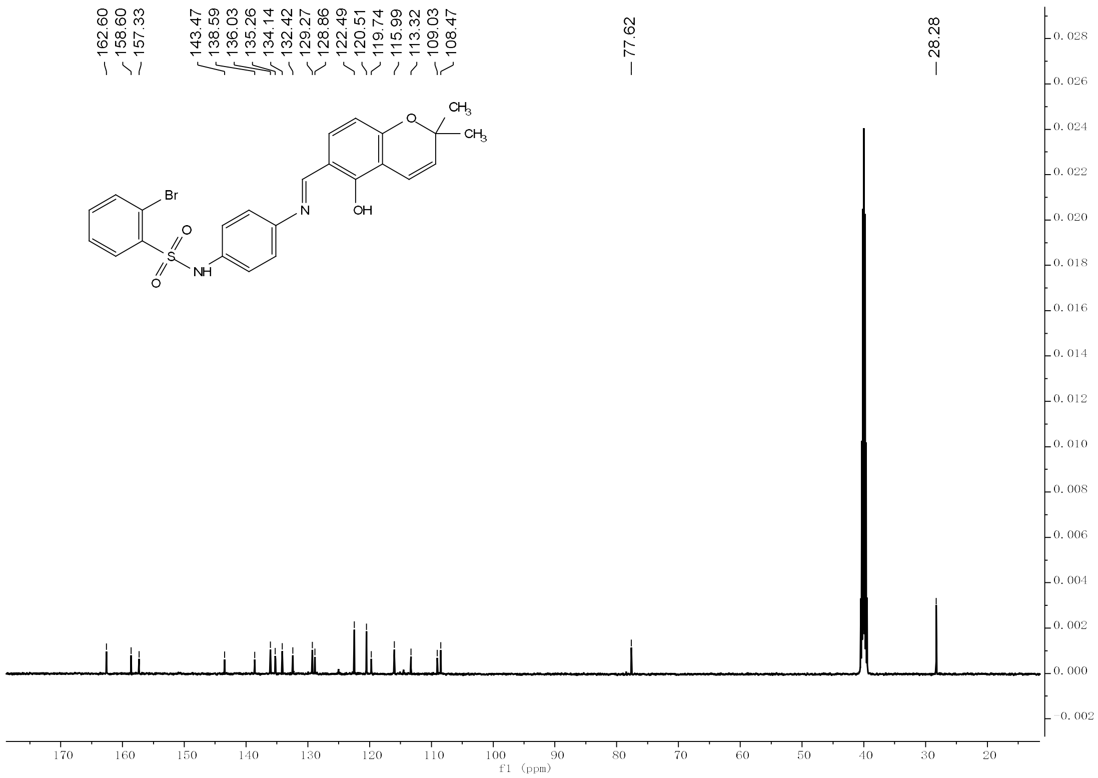

^{13}C NMR (126 MHz, DMSO-d_6) spectrum of compound **C33**.

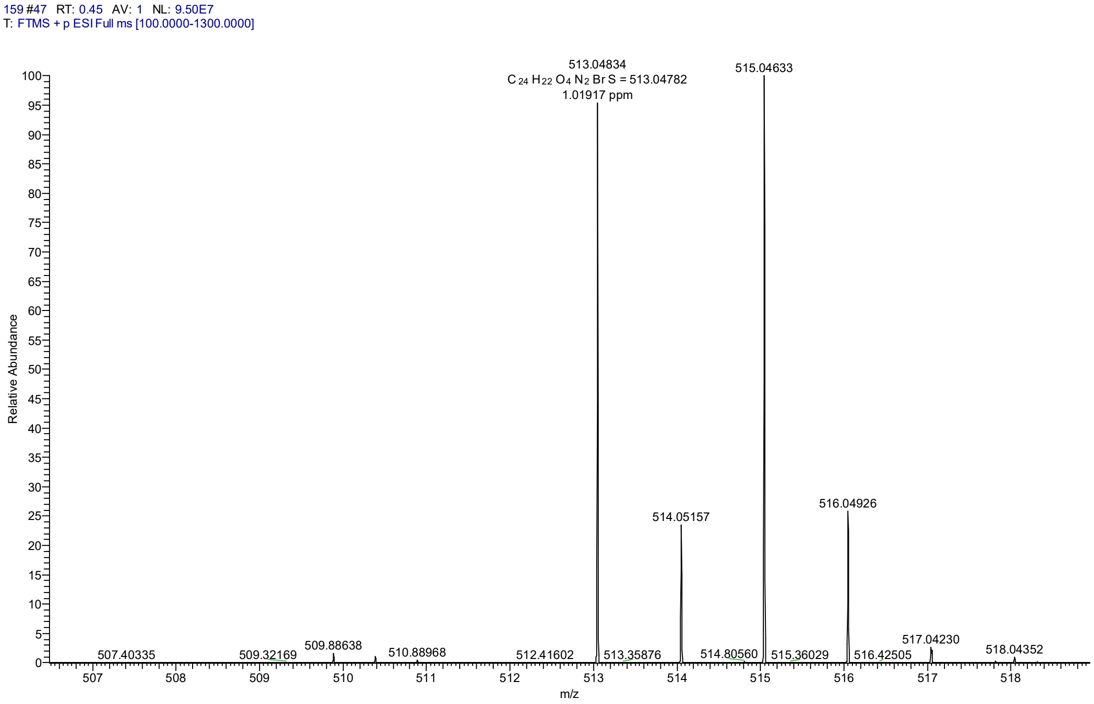

HRMS of compoun **C33**

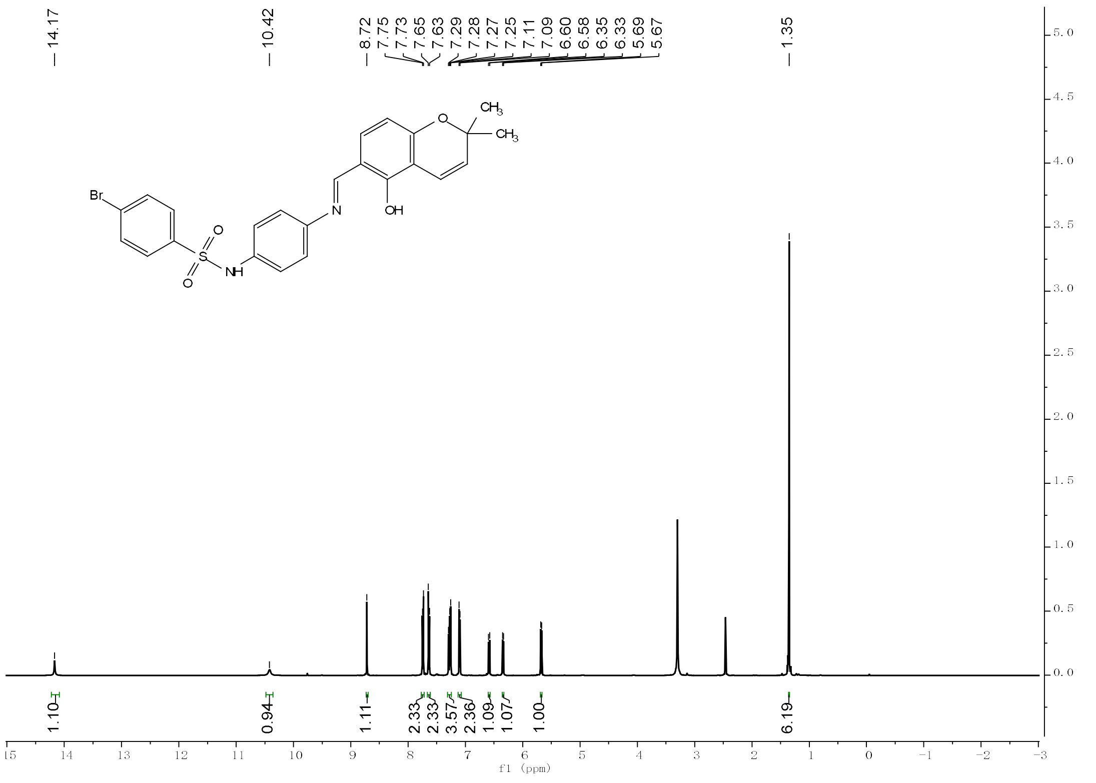

^{1}H NMR (500 MHz, DMSO-d_6) spectrum of compound **C34**.

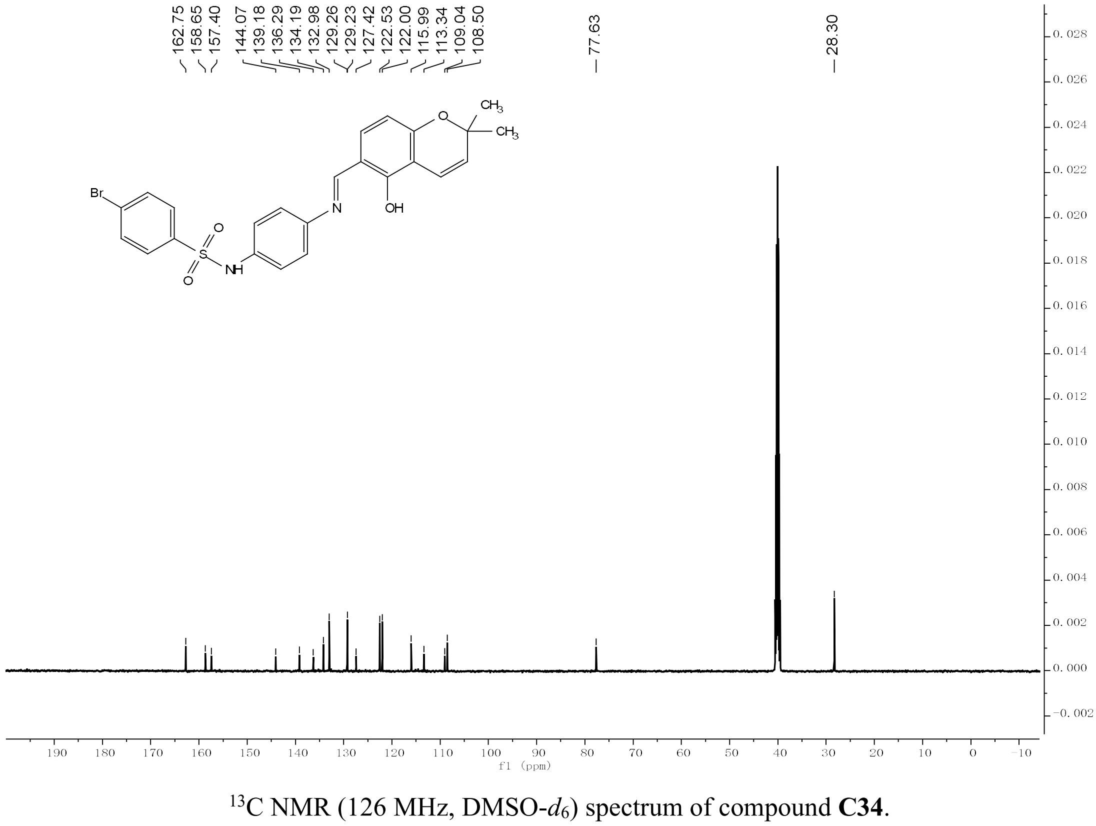

^{13}C NMR (126 MHz, DMSO-d_6) spectrum of compound **C34**.

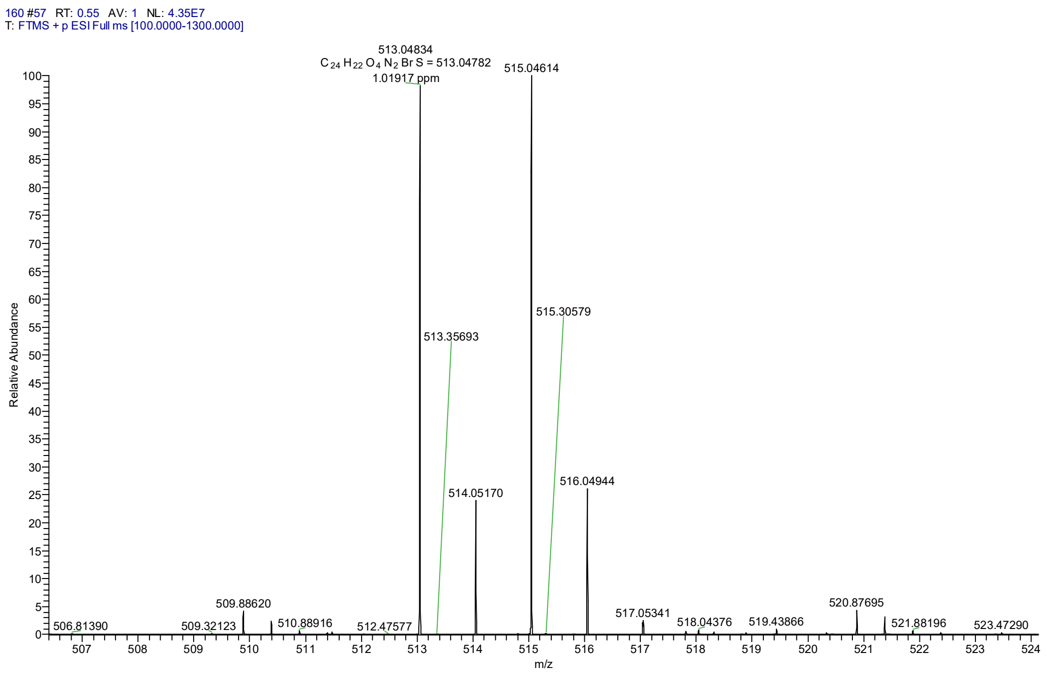

HRMS of compound **C34**

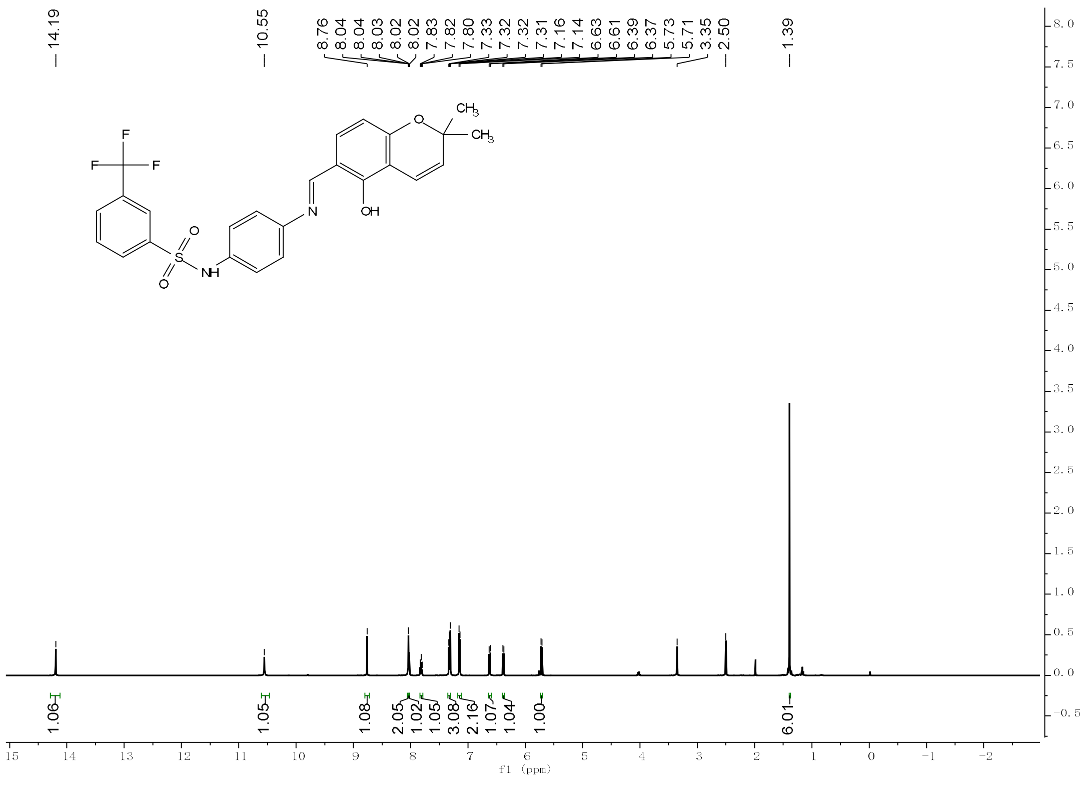

^{1}H NMR (500 MHz, DMSO-d_6) spectrum of compound **C35**.

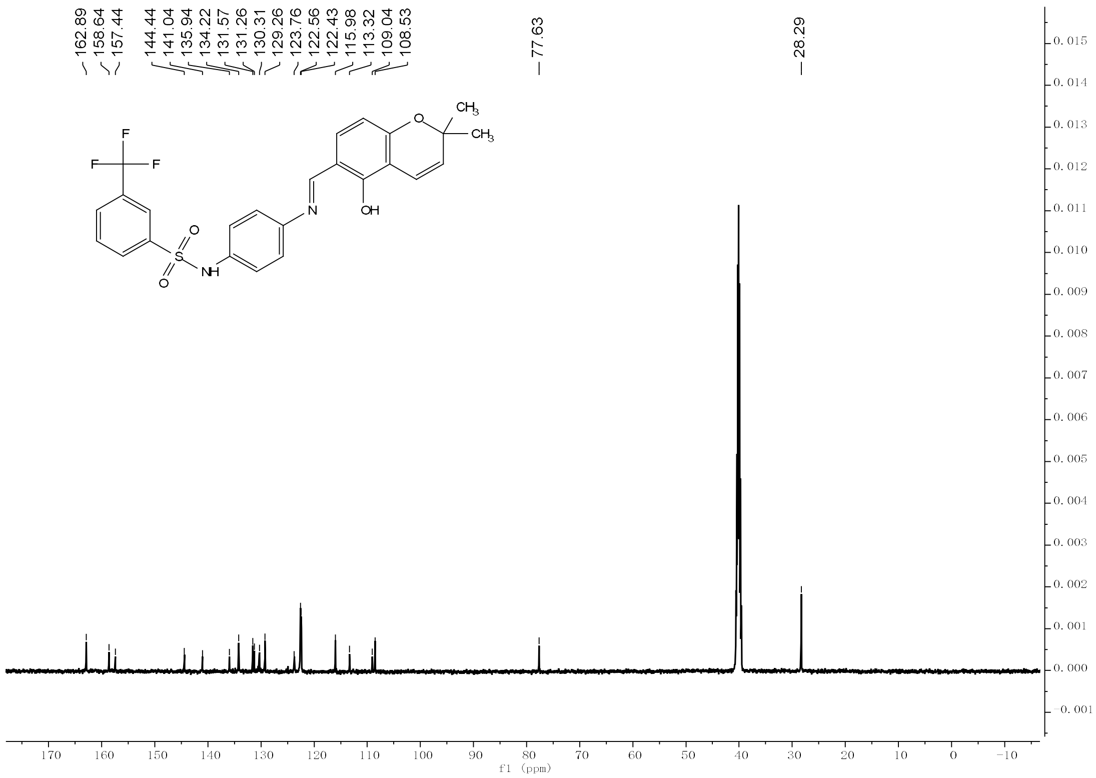

^{13}C NMR (126 MHz, DMSO-d_6) spectrum of compound **C35**.

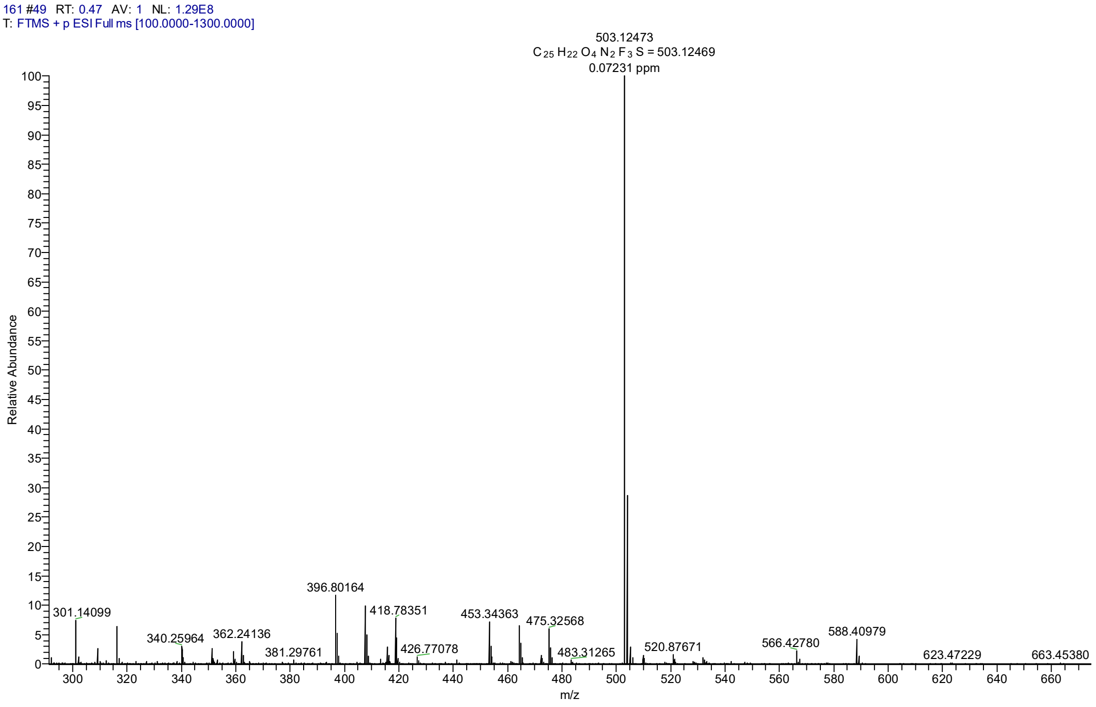

HRMS of compoun **C35**

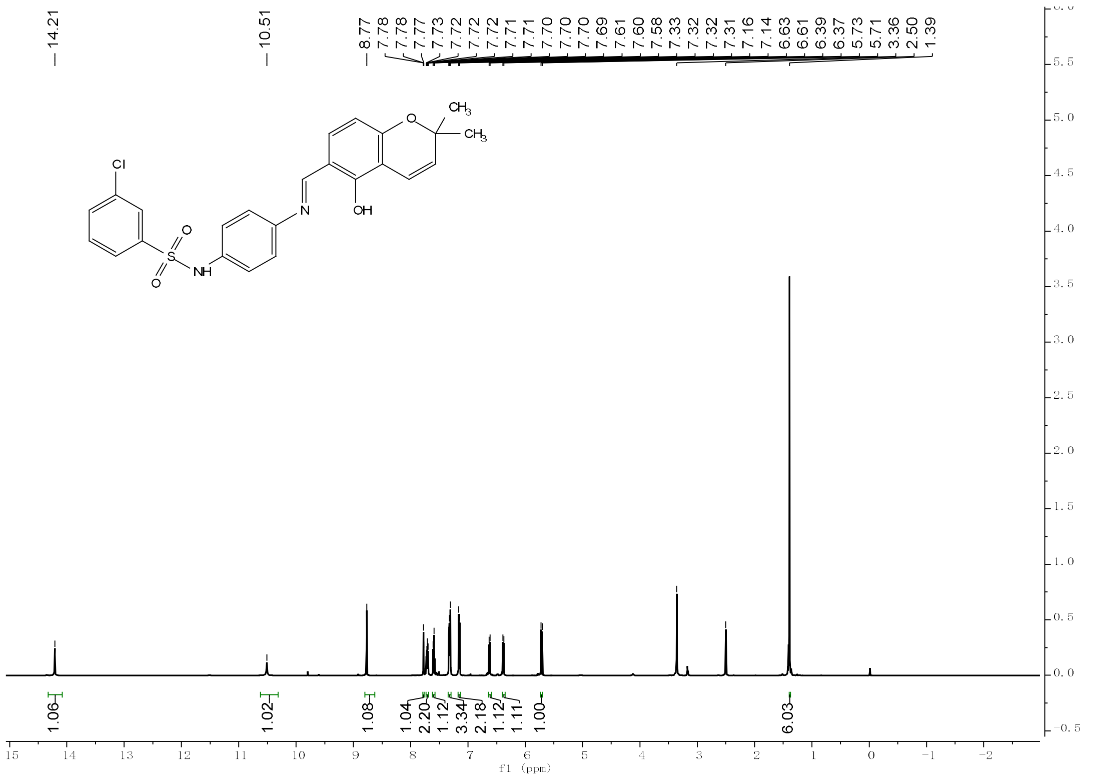

^{1}H NMR (500 MHz, DMSO-d_6) spectrum of compound **C36**.

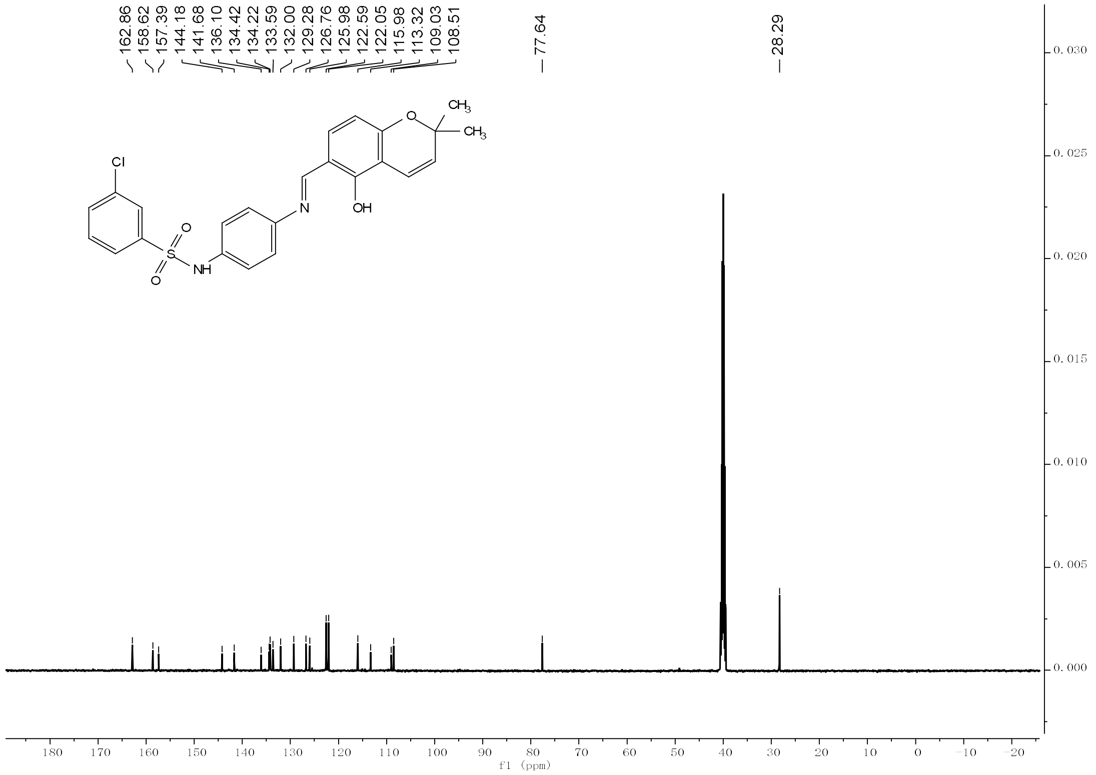

^{13}C NMR (126 MHz, DMSO-d_6) spectrum of compound **C36**.

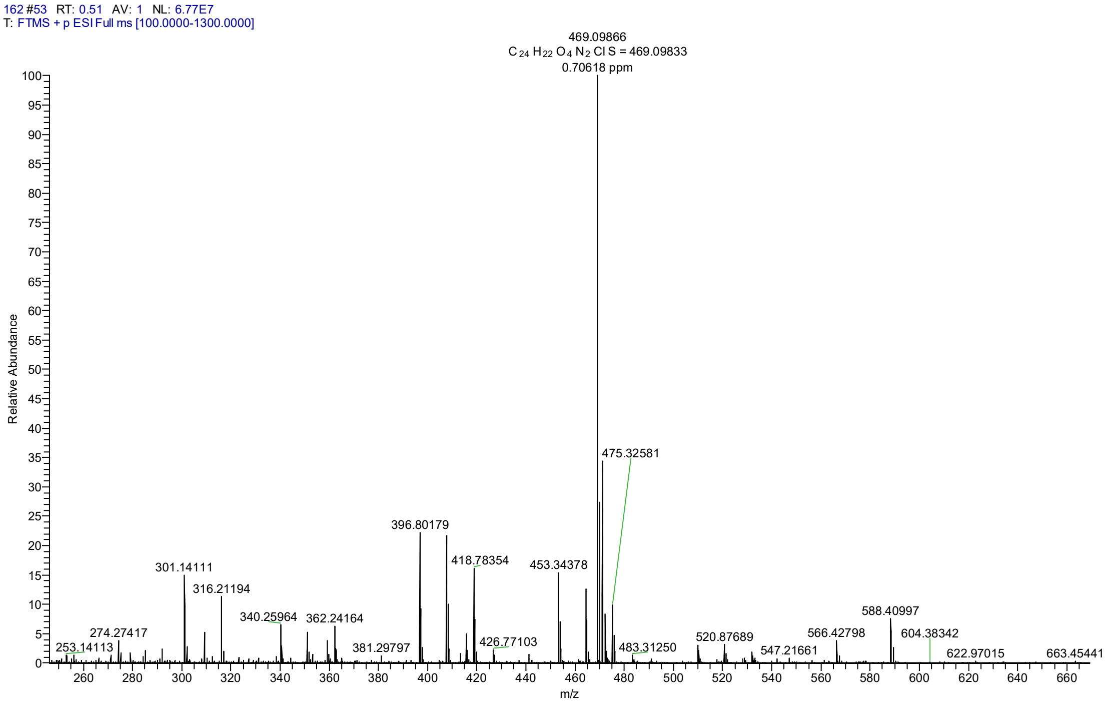

HRMS of compou **C36**

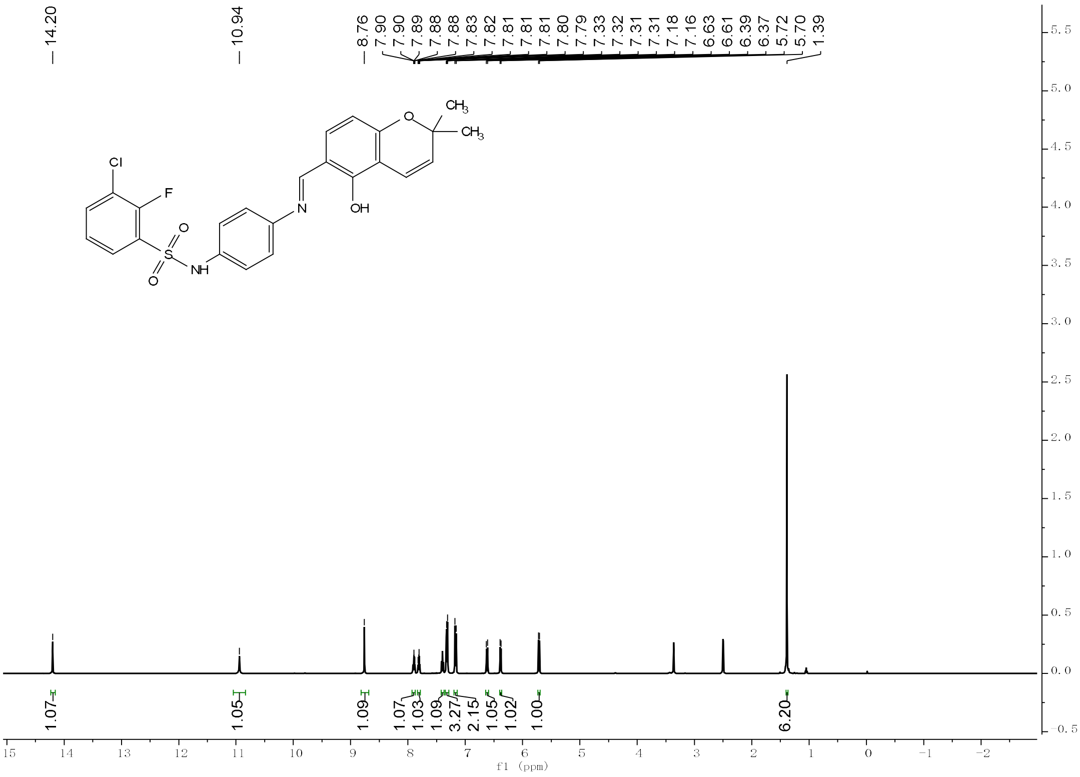

^{1}H NMR (500 MHz, DMSO-d_6) spectrum of compound **C37**.

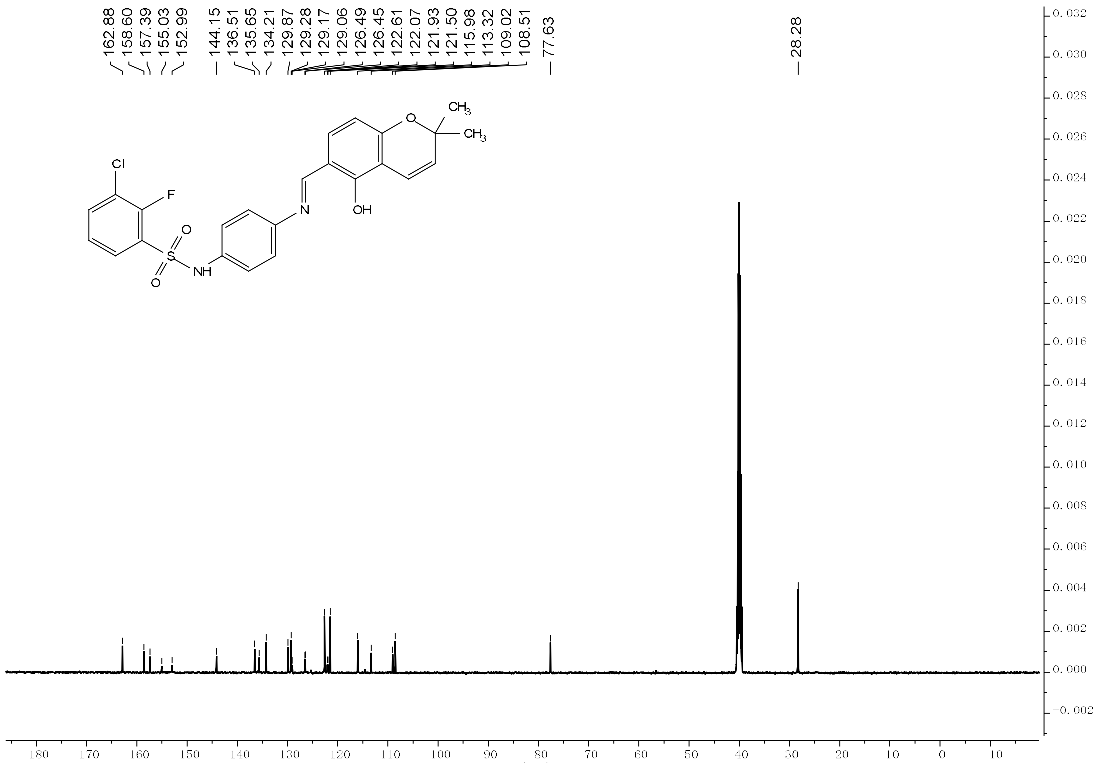

^{13}C NMR (126 MHz, DMSO-d_6) spectrum of compound **C37**.

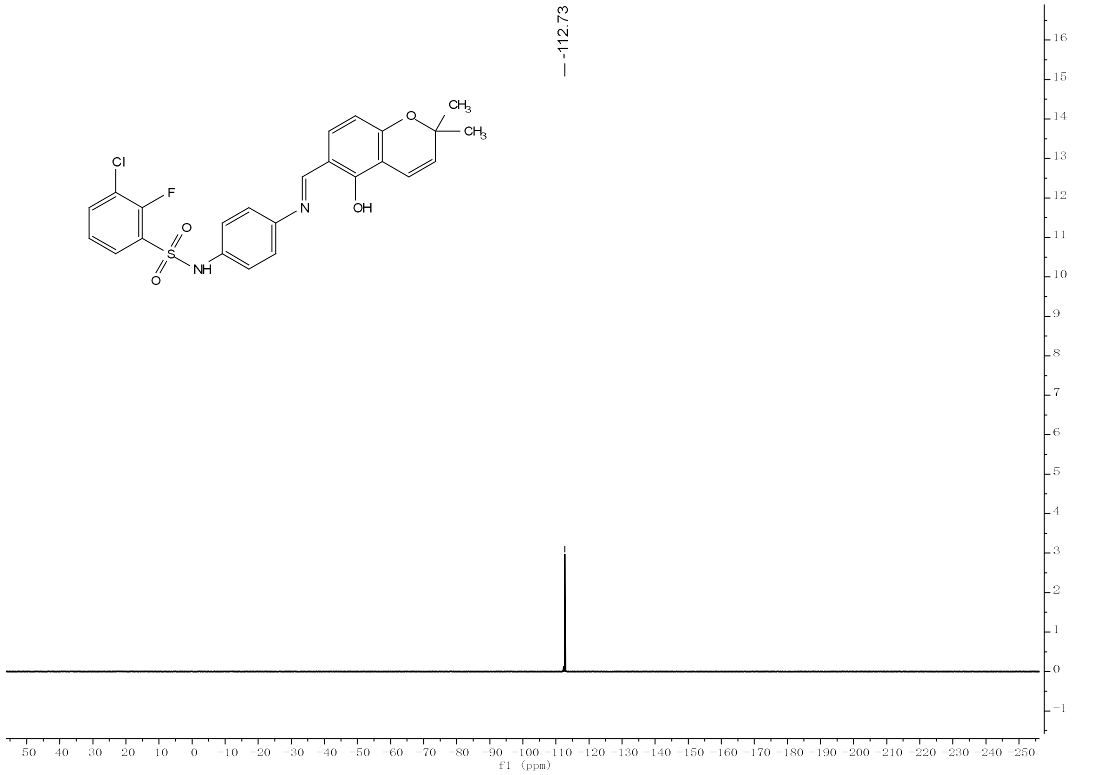

^{19}F NMR (471 MHz, DMSO-d_6) spectrum of compound **C37**.

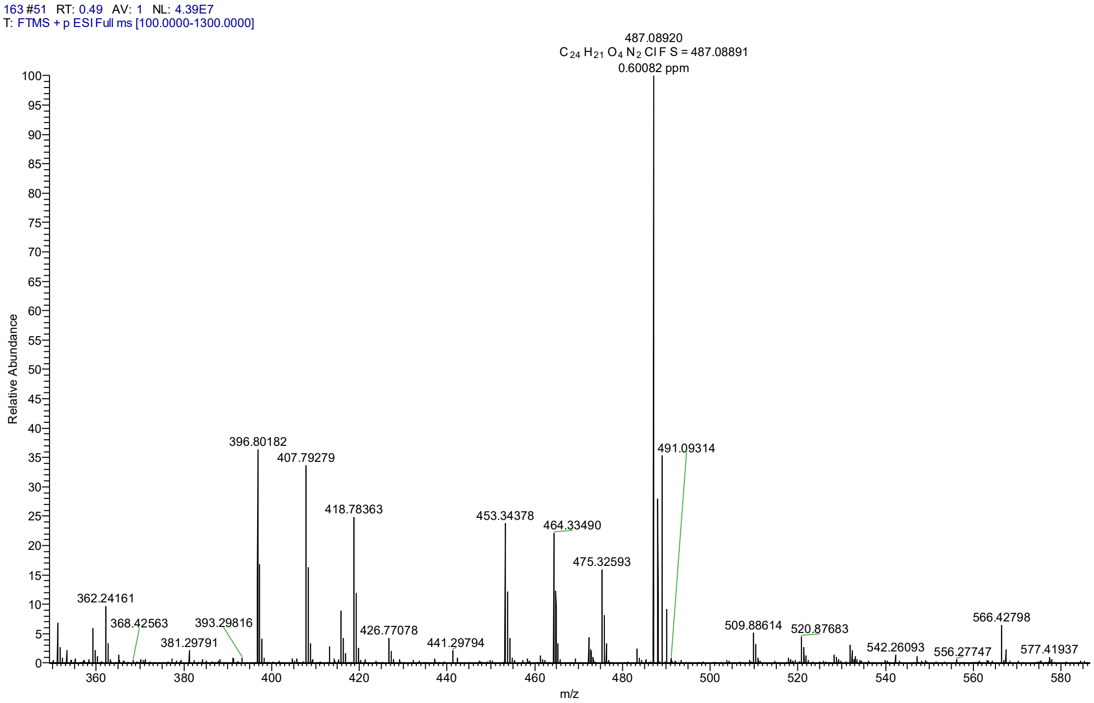

HRMS of comp **C37**

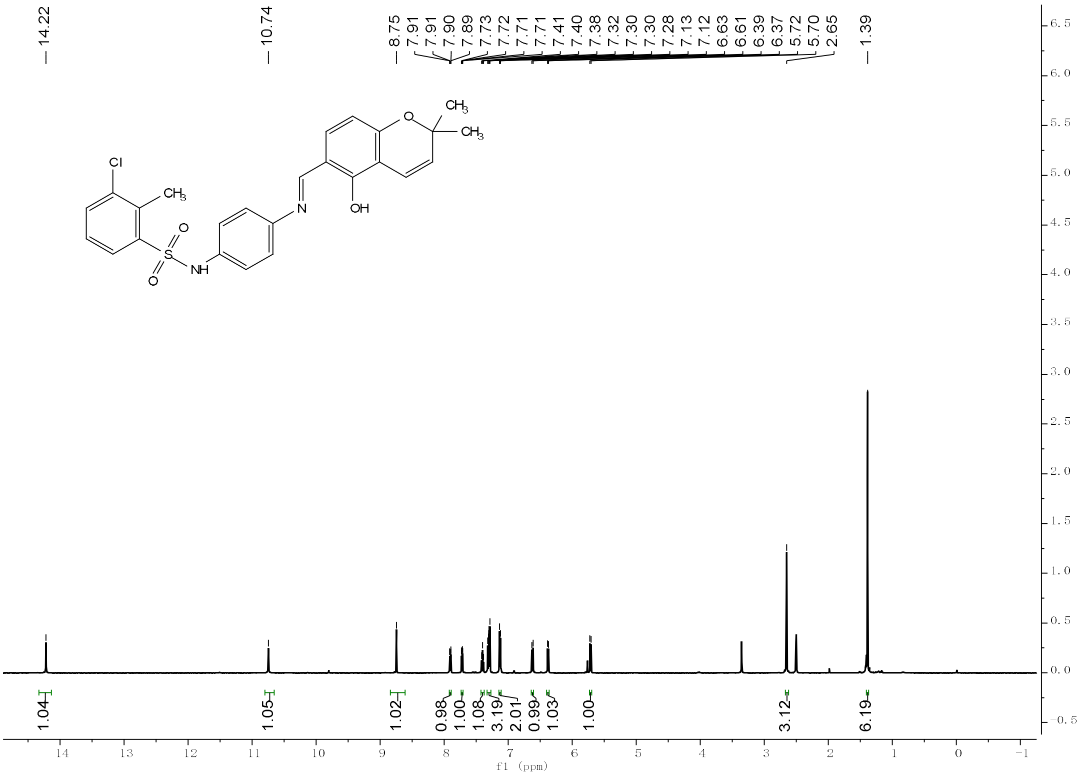

^{1}H NMR (500 MHz, DMSO-d_6) spectrum of compound **C38**.

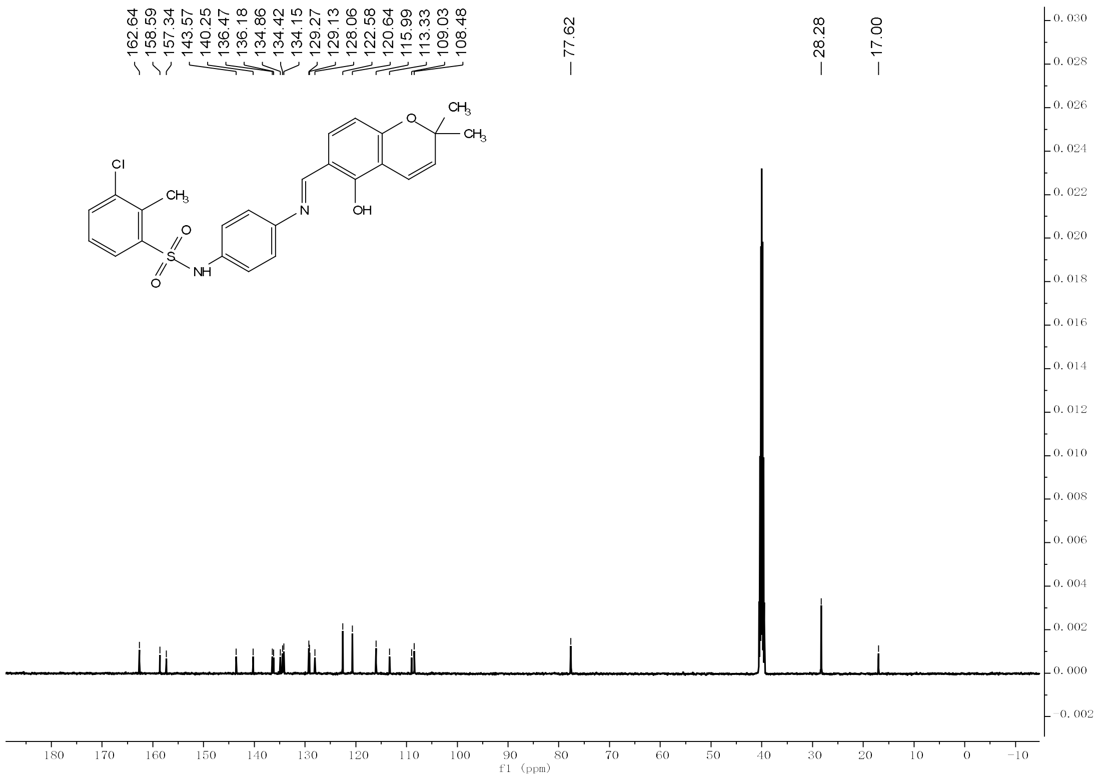

^{13}C NMR (126 MHz, DMSO-d_6) spectrum of compound **C38**.

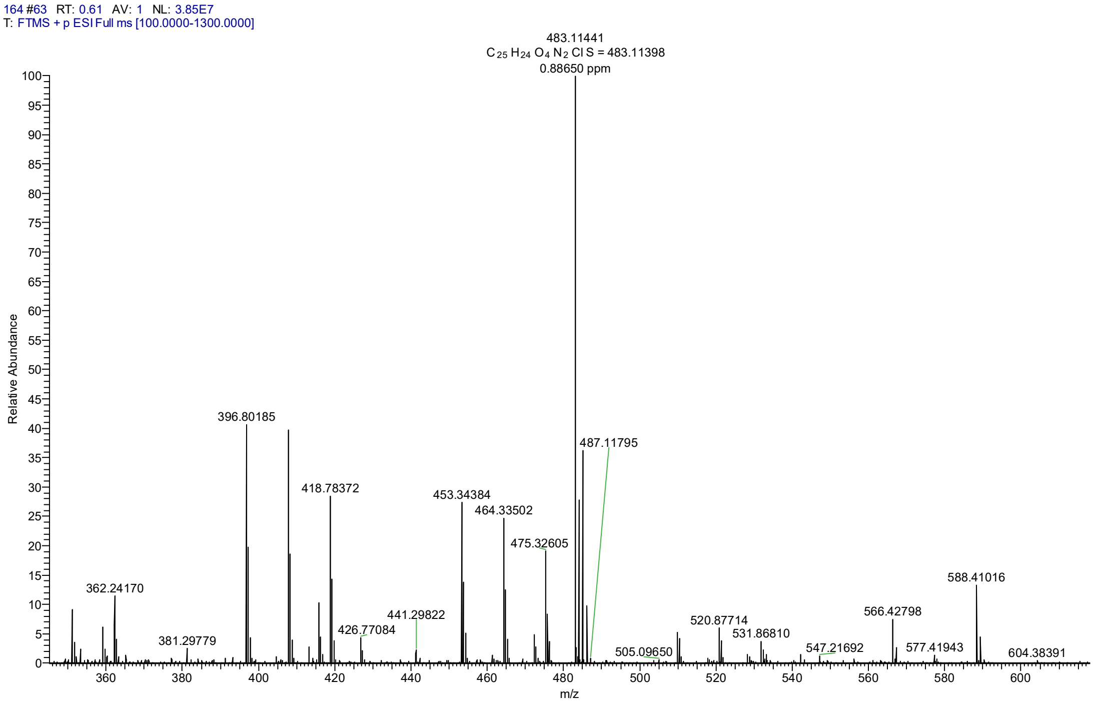

HRMS of compound **C38**.

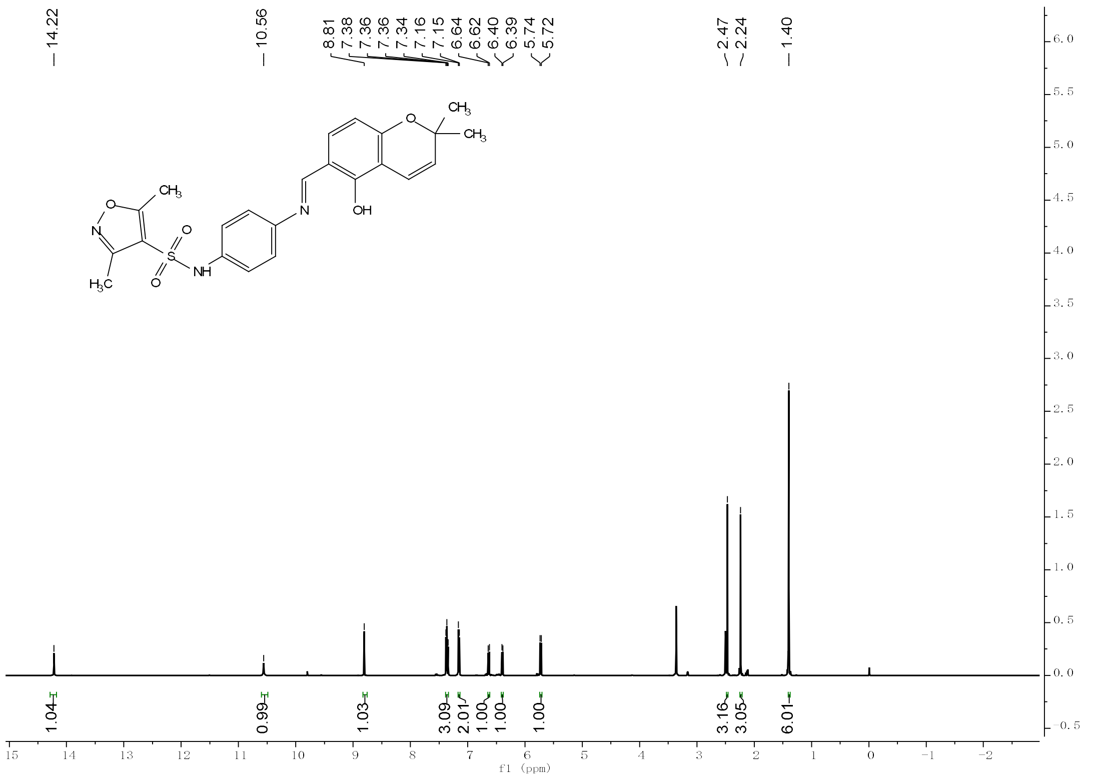

^{1}H NMR (500 MHz, DMSO-d_6) spectrum of compound **C39**.

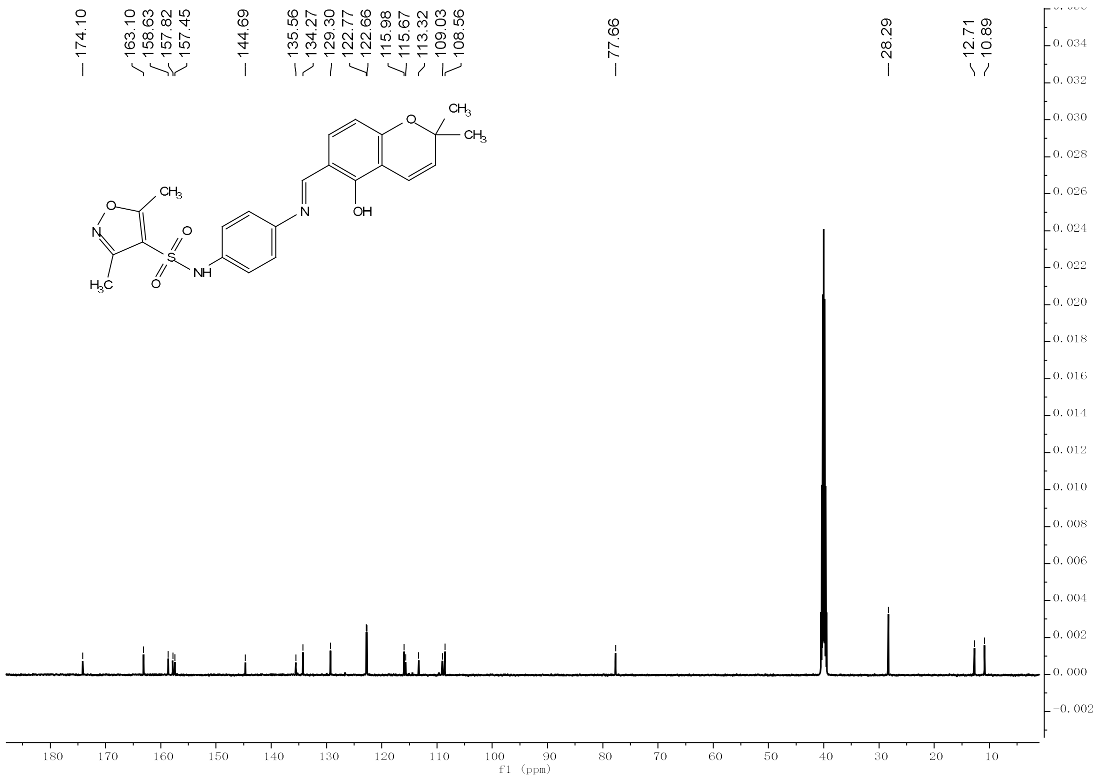

^{13}C NMR (126 MHz, DMSO-d_6) spectrum of compound **C39**.

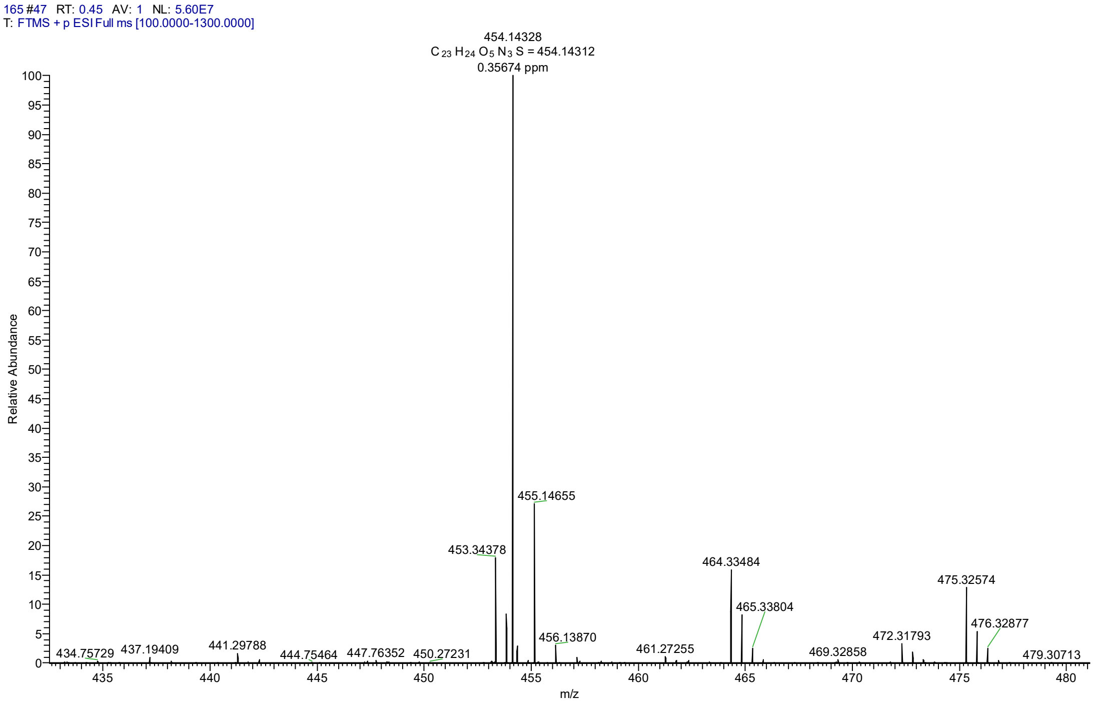

HRMS of compound **C39**

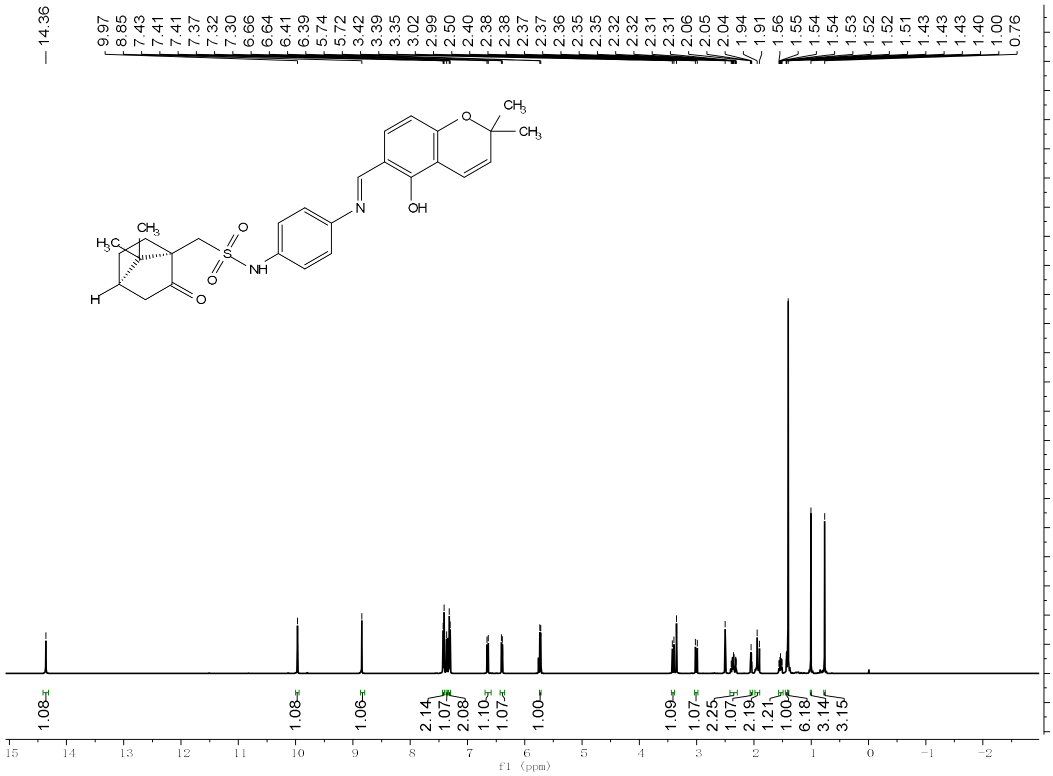

^{1}H NMR (500 MHz, DMSO-d_6) spectrum of compound **C40**.

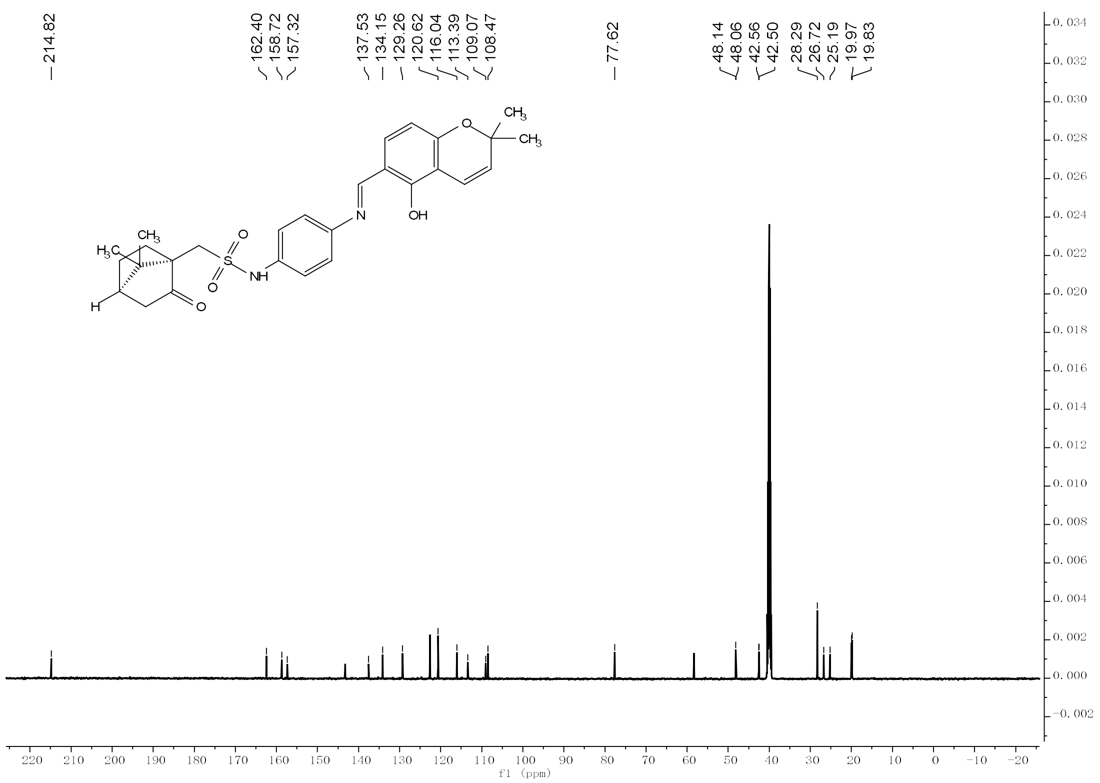

^{13}C NMR (126 MHz, DMSO-d_6) spectrum of compound **C40**.

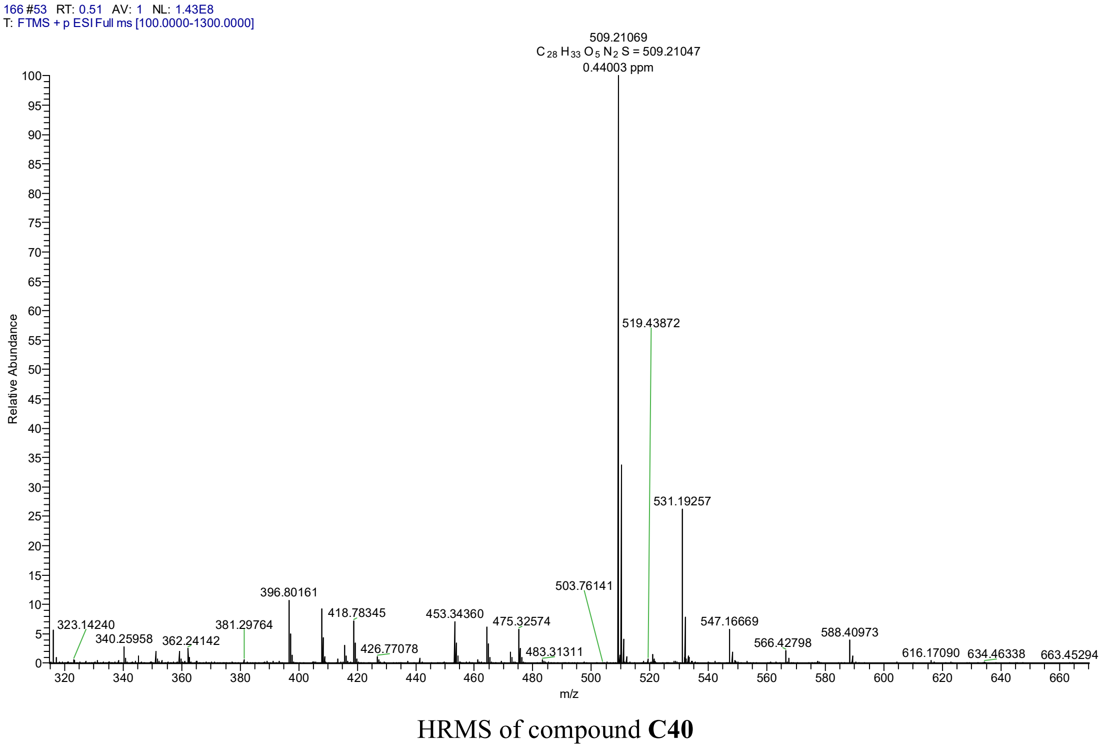

HRMS of compound **C40**

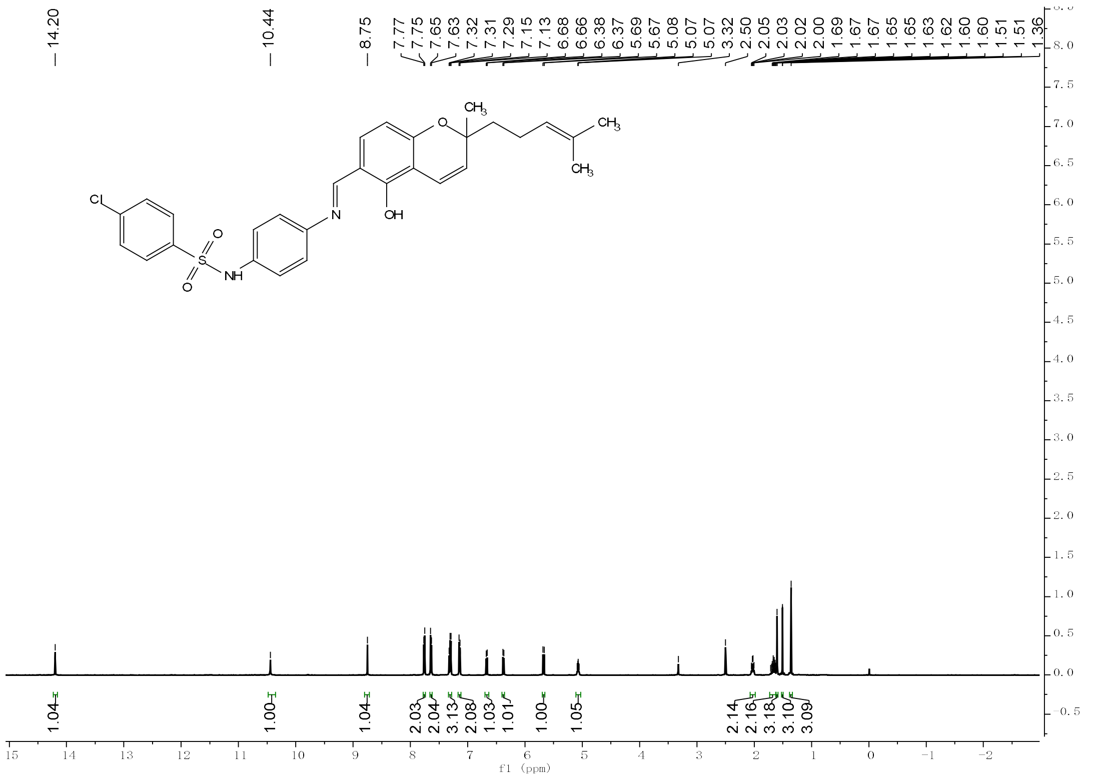

^{1}H NMR (500 MHz, DMSO-d_6) spectrum of compound **C41**.

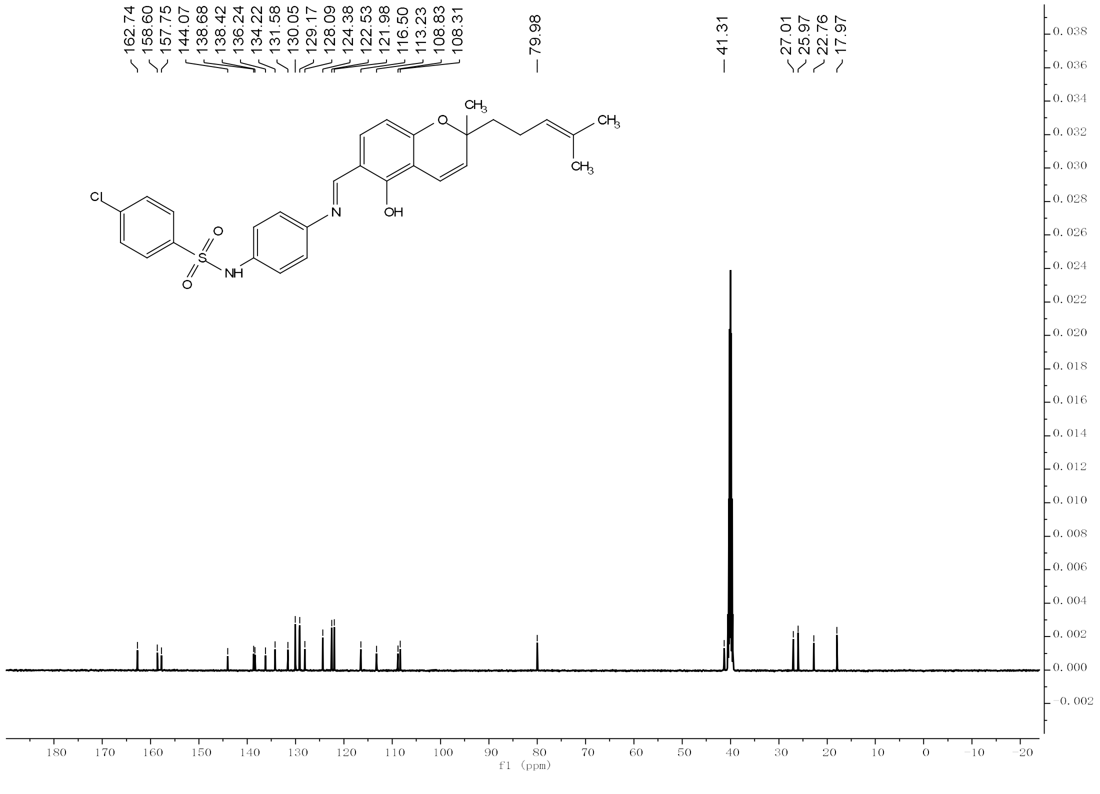

^{13}C NMR (126 MHz, DMSO-d_6) spectrum of compound **C41**.

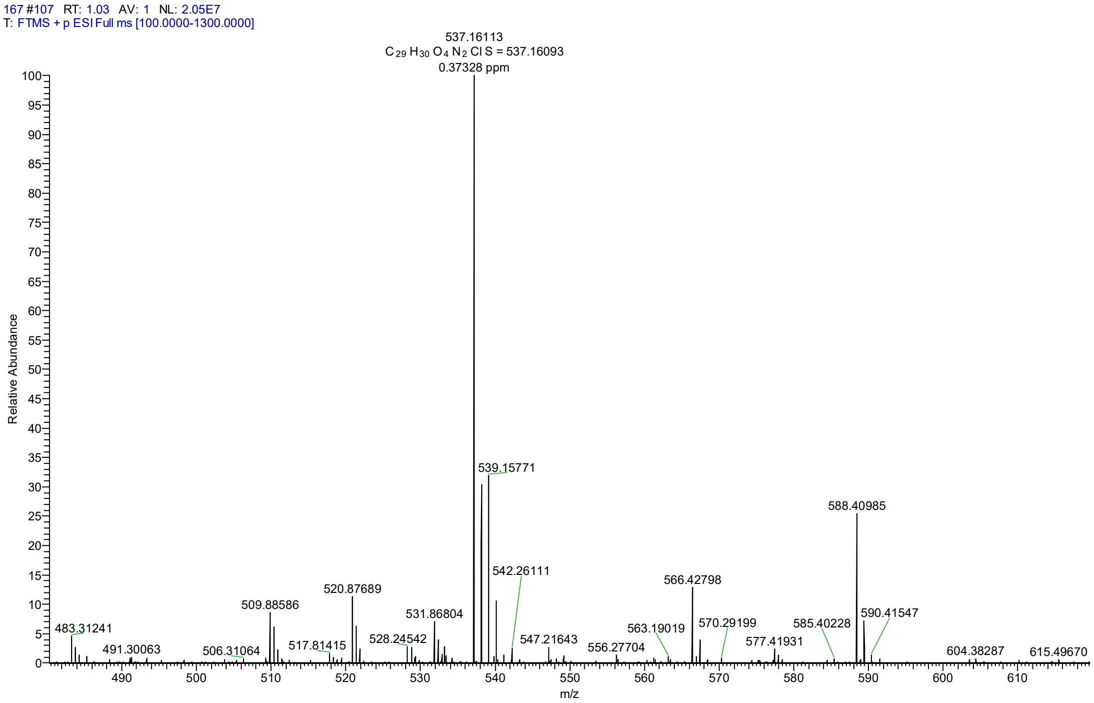

HRMS of compound **C41.**

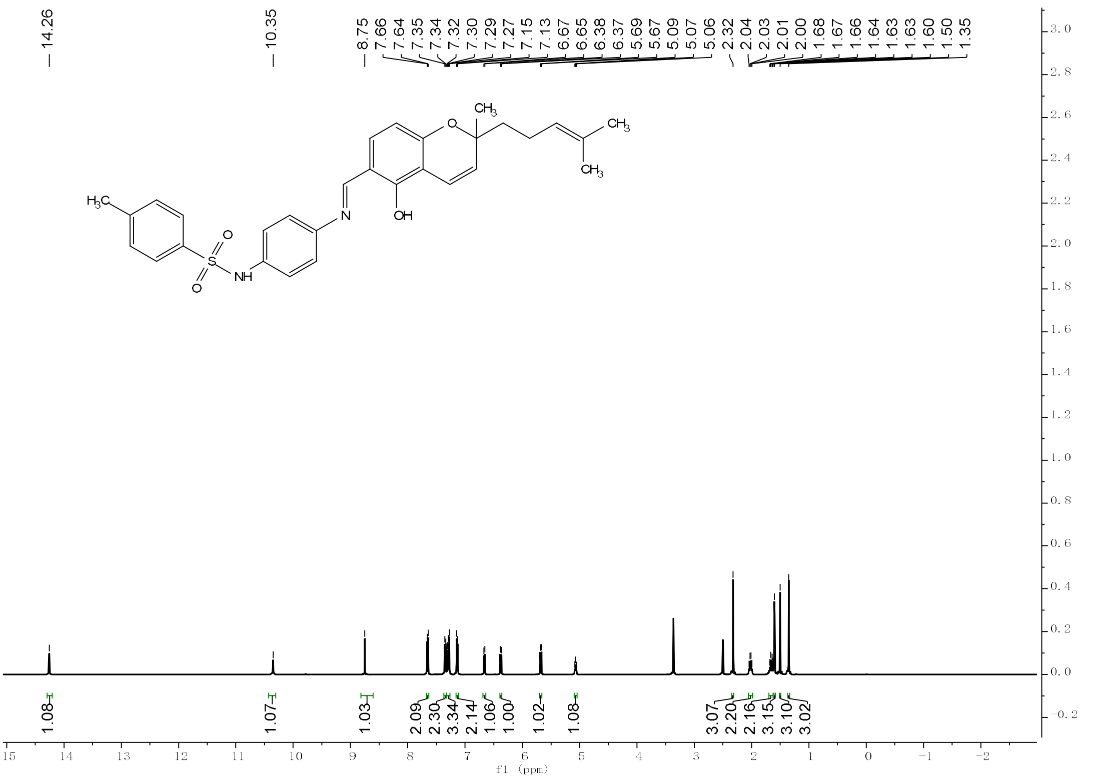

^{1}H NMR (500 MHz, DMSO-d_6) spectrum of compound **C42**.

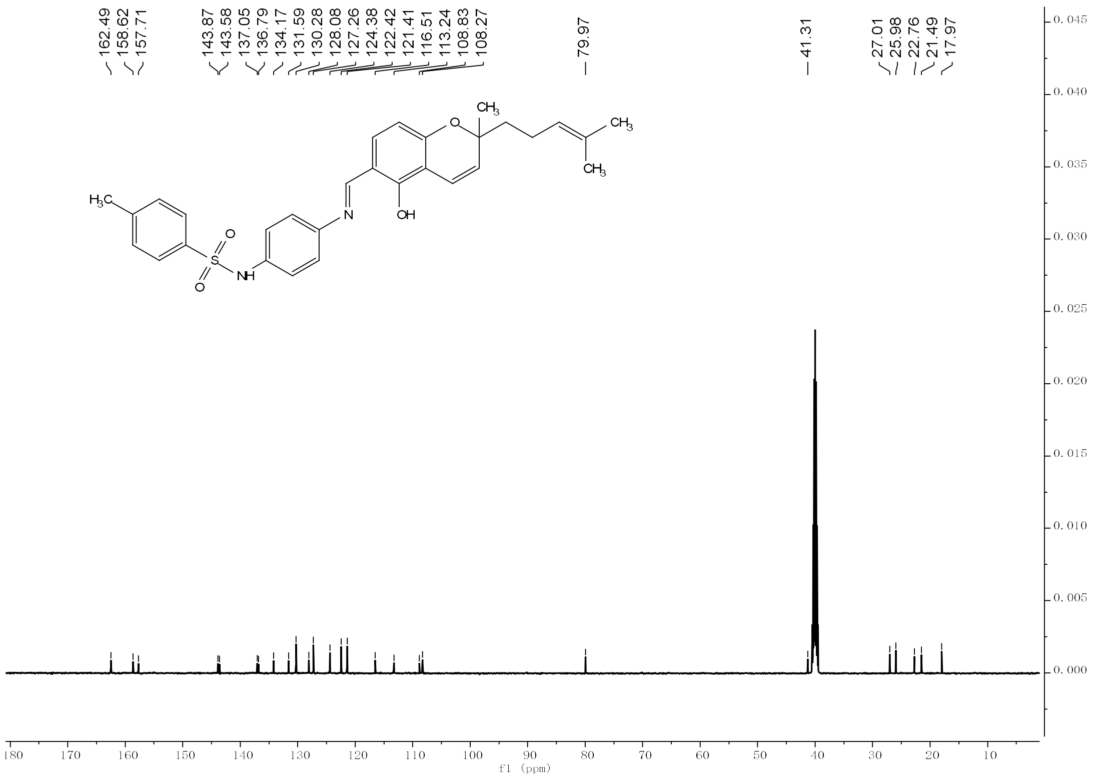

^{13}C NMR (126 MHz, DMSO-d_6) spectrum of compound **C42**.

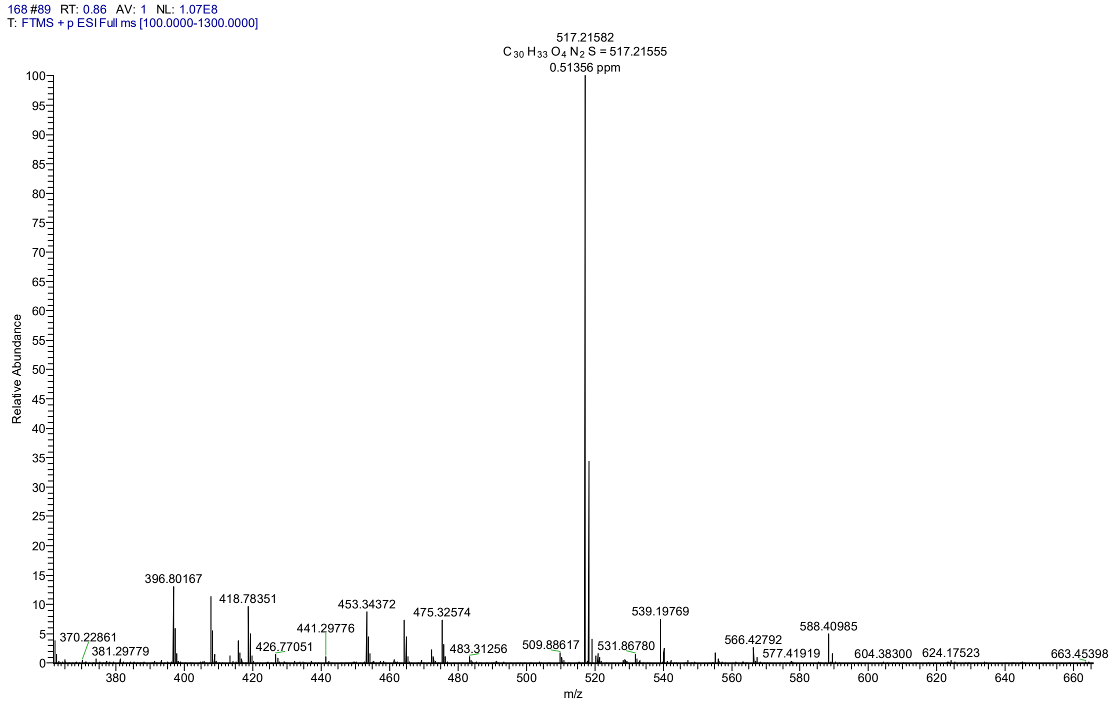

HRMS of compound **C42.**

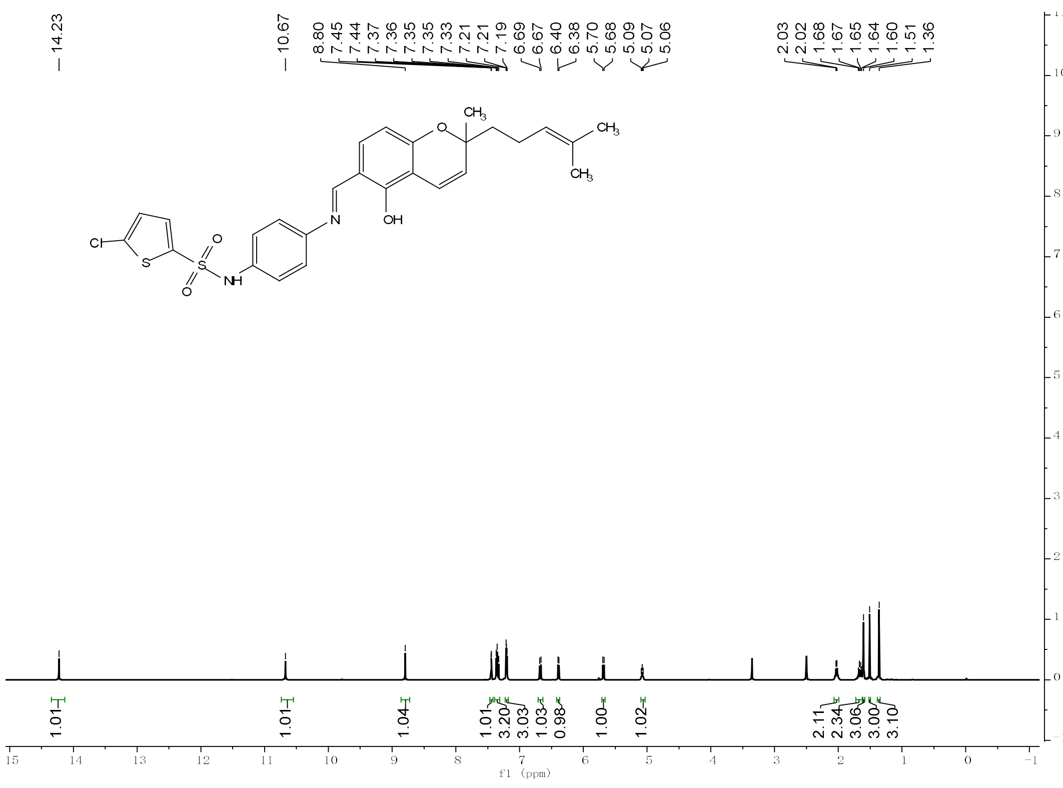

^{1}H NMR (500 MHz, DMSO-d_6) spectrum of compound **C43**.

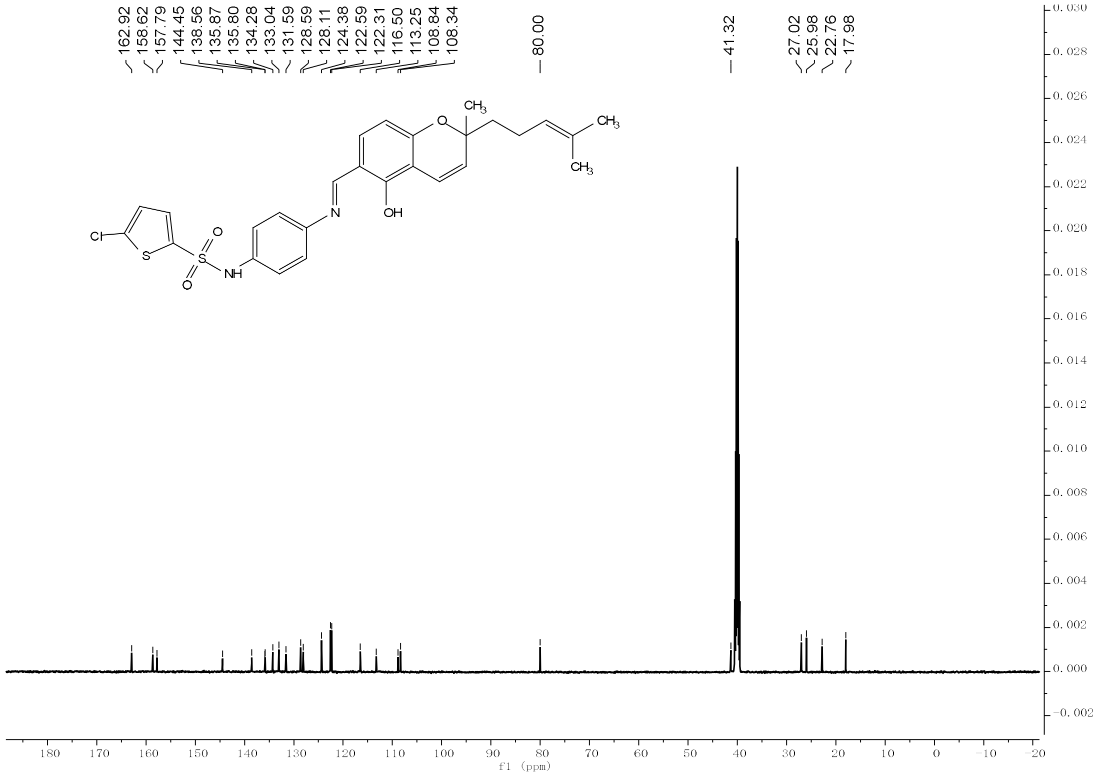

^{13}C NMR (126 MHz, DMSO-d_6) spectrum of compound **C43**.

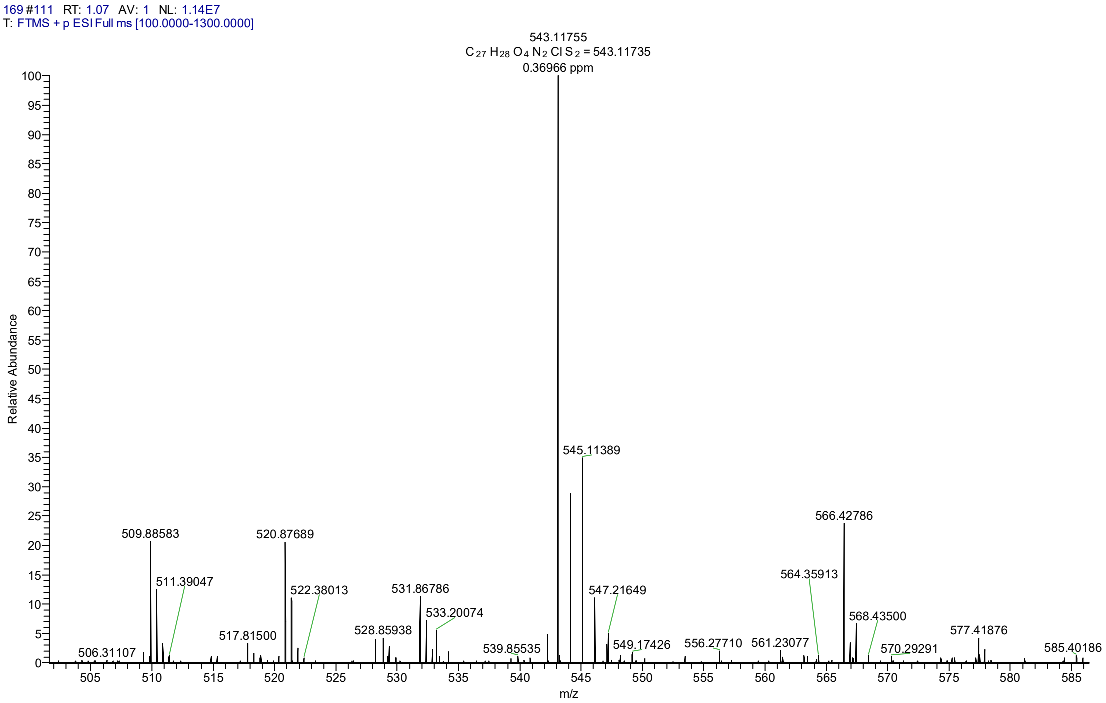

HRMS of compound **C43.**

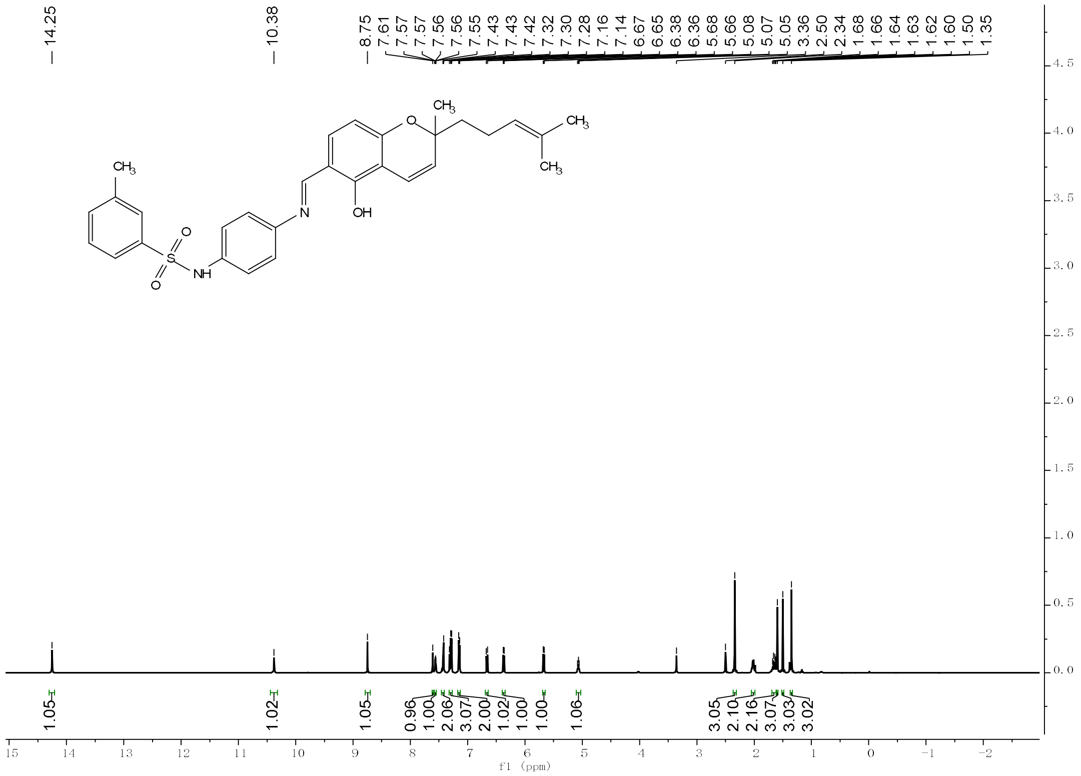

^{1}H NMR (500 MHz, DMSO-d_6) spectrum of compound **C44**.

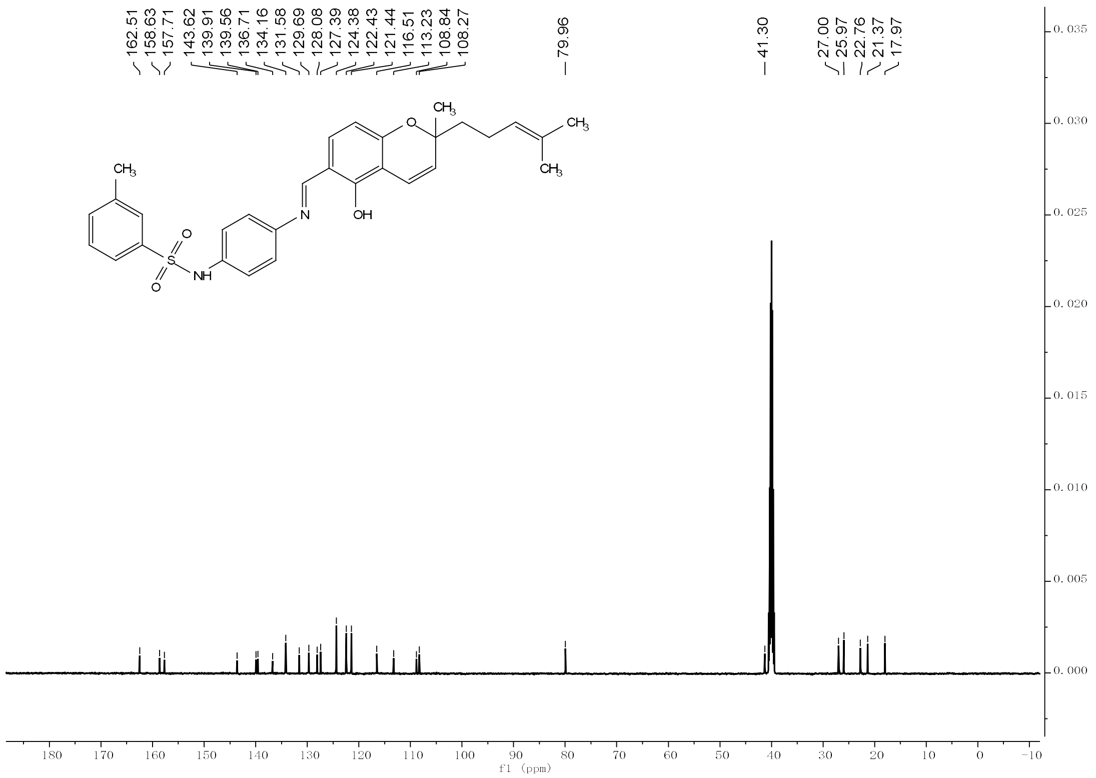

^{13}C NMR (126 MHz, DMSO-d_6) spectrum of compound **C44**.

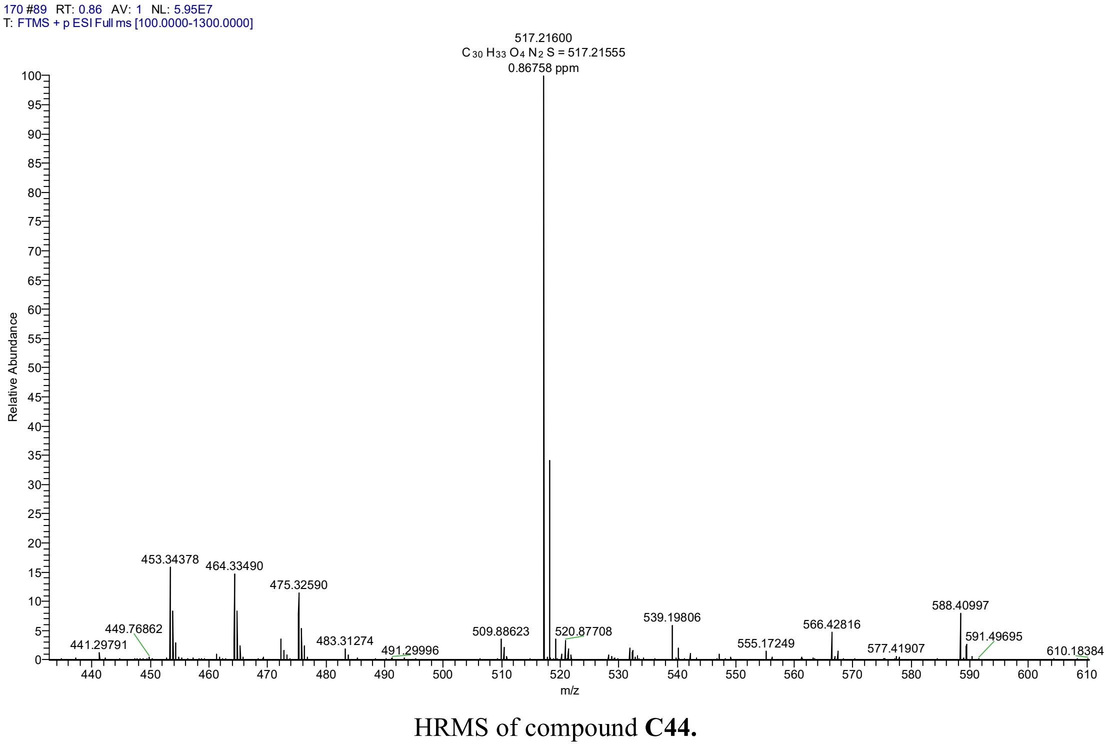

HRMS of compound **C44.**

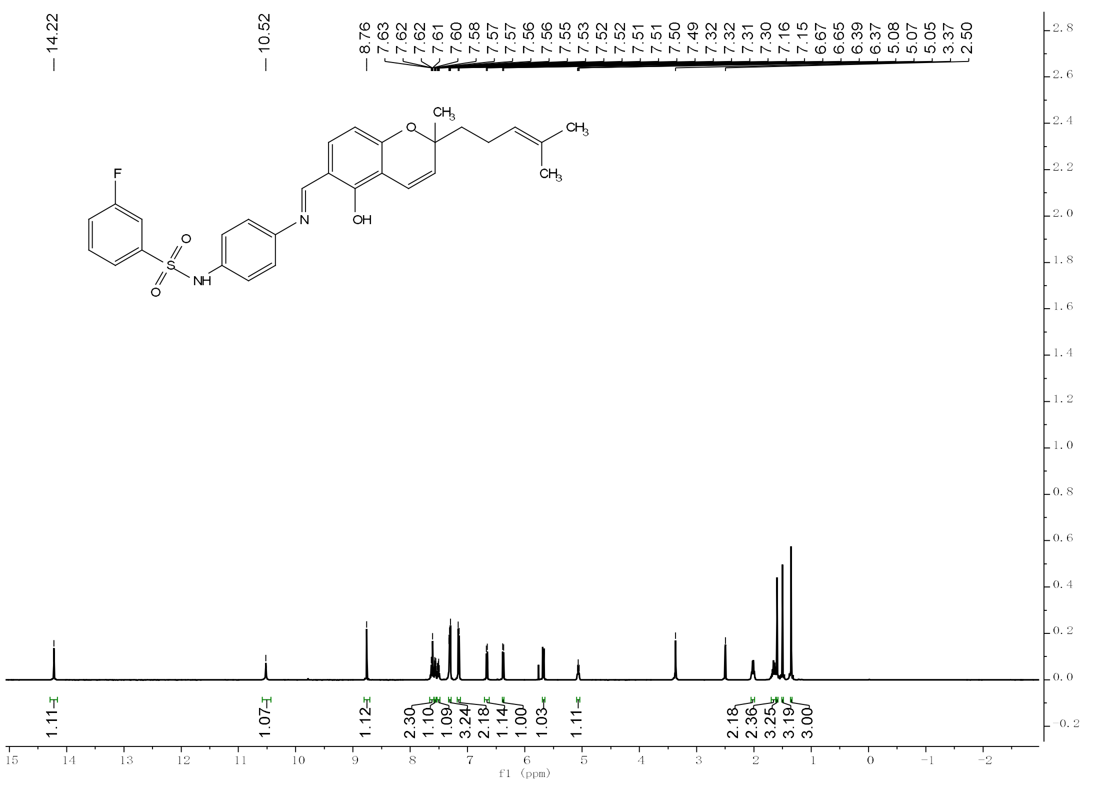

^{1}H NMR (500 MHz, DMSO-d_6) spectrum of compound **C45**.

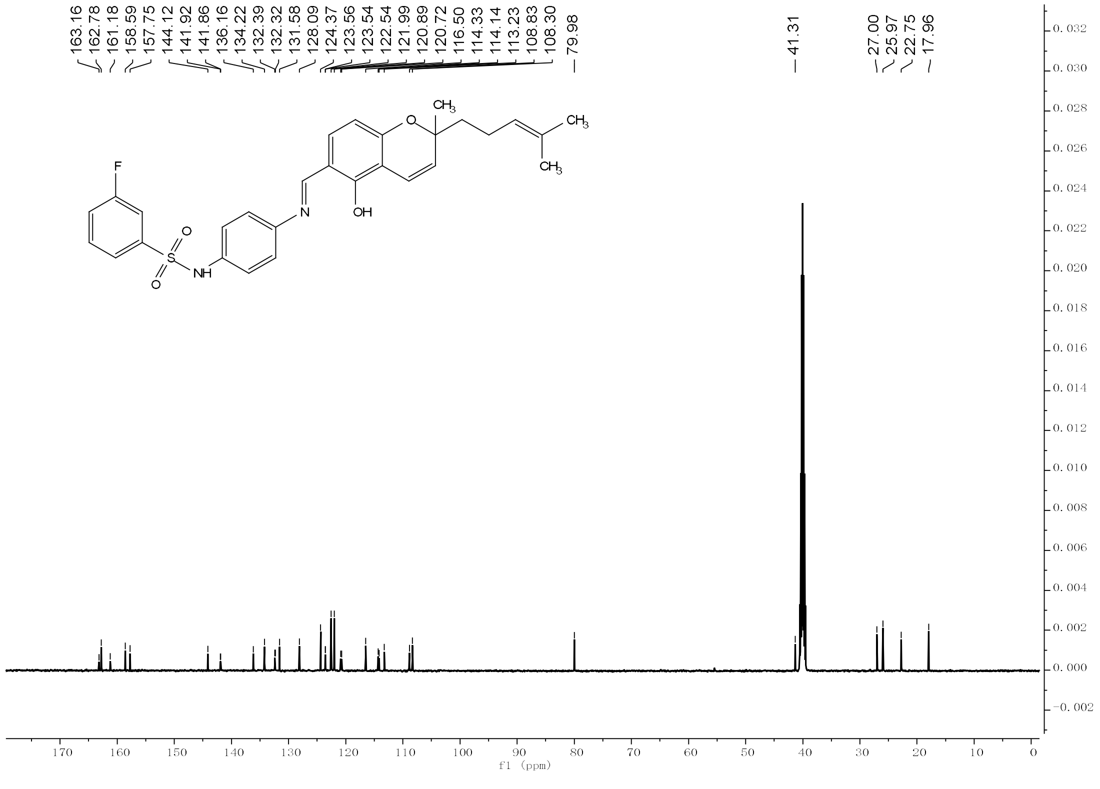

^{13}C NMR (126 MHz, DMSO-d_6) spectrum of compound **C45**.

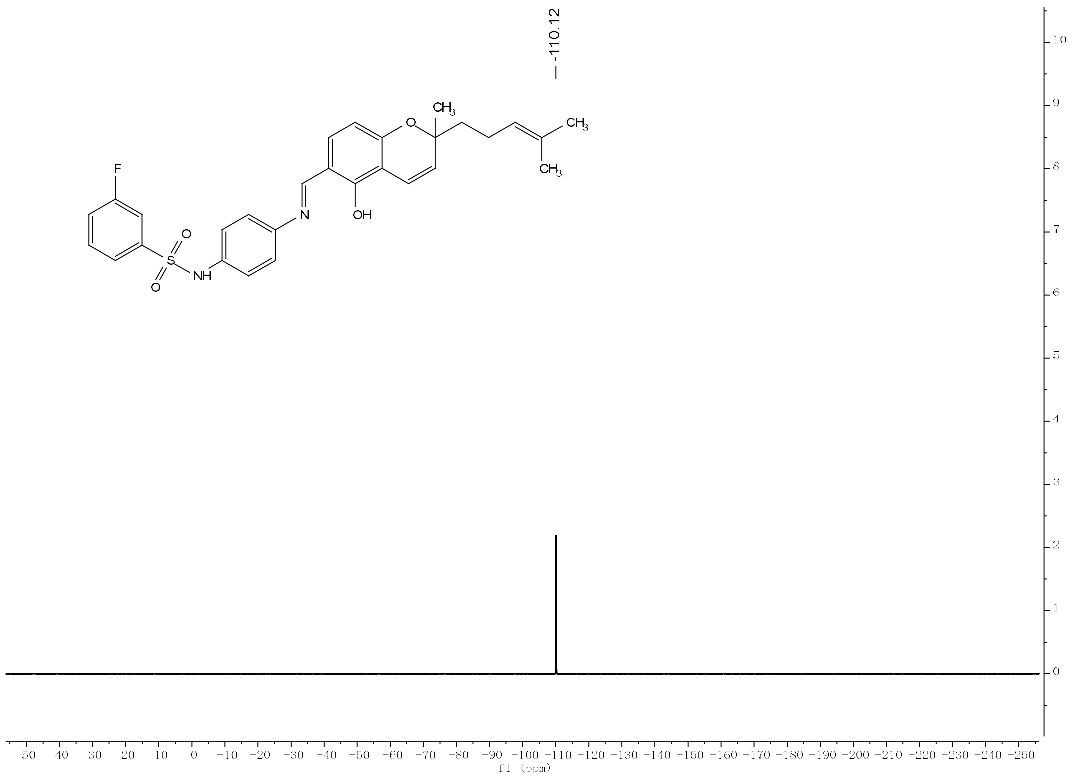

^{19}F NMR (471 MHz, DMSO-d_6) spectrum of compound **C45**.

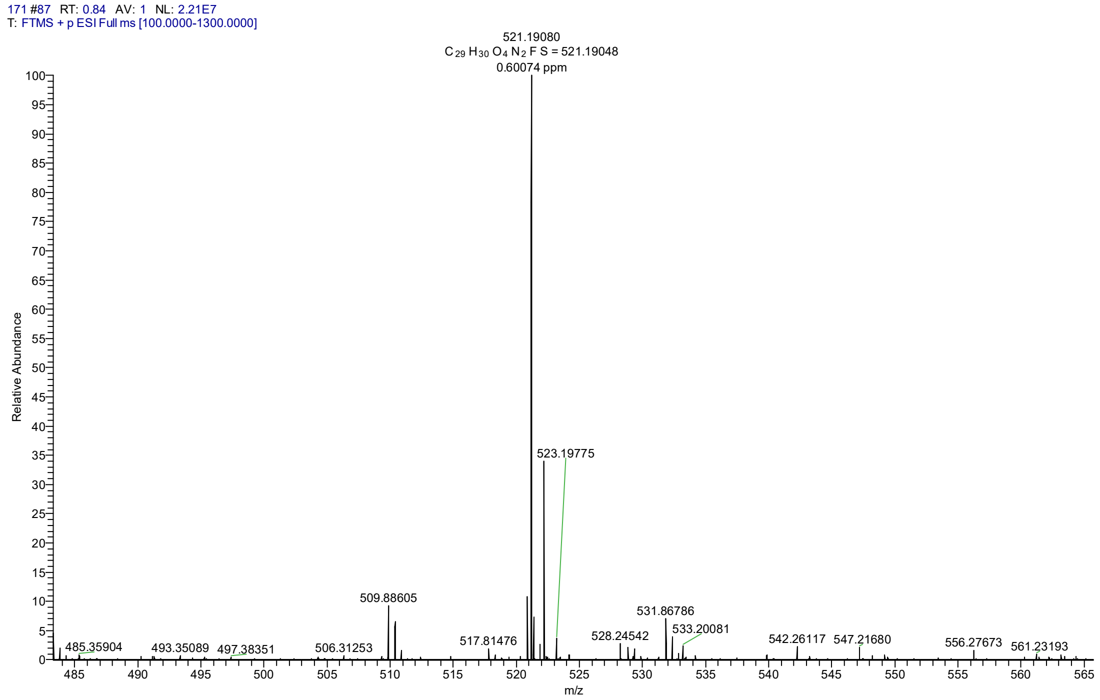

HRMS of compound **C46.**

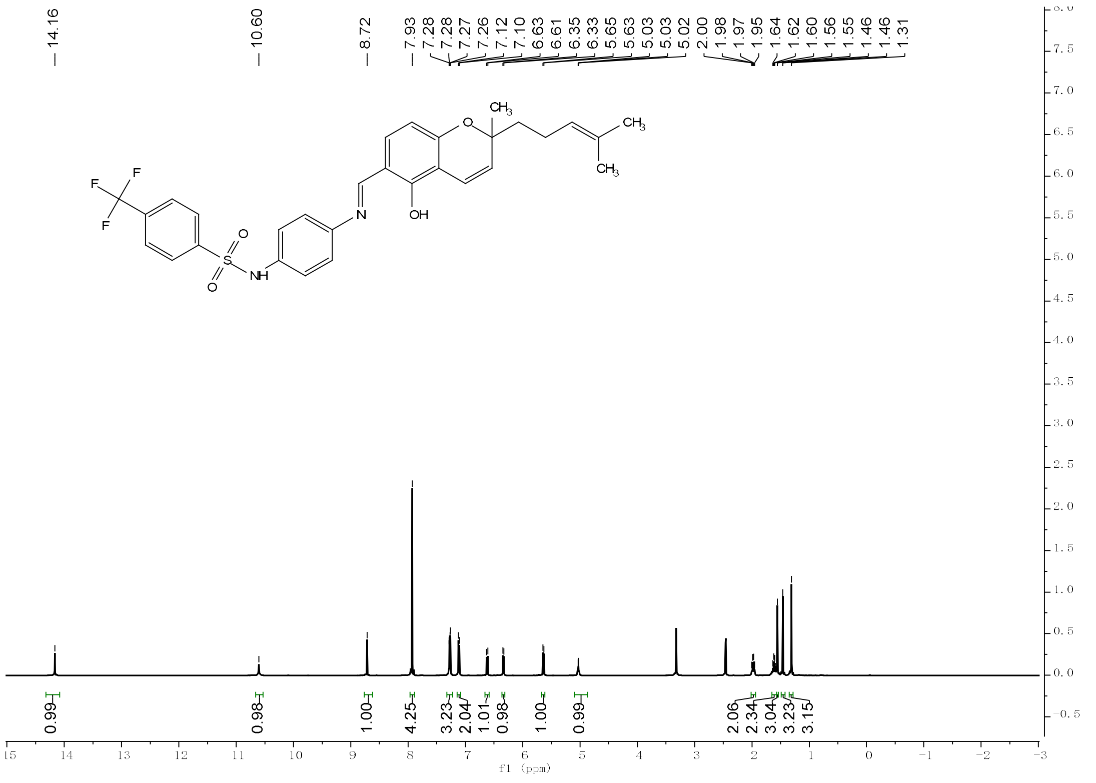

^{1}H NMR (500 MHz, DMSO-d_6) spectrum of compound **C46**.

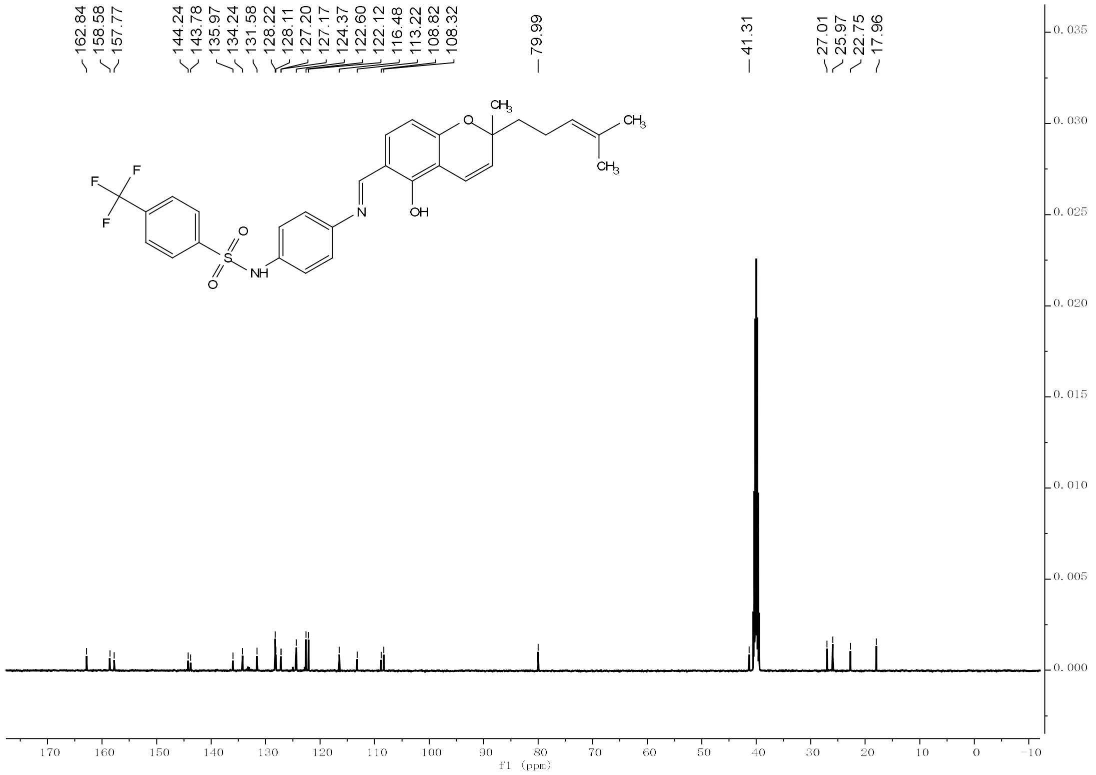

^{13}C NMR (126 MHz, DMSO-d_6) spectrum of compound **C46**.

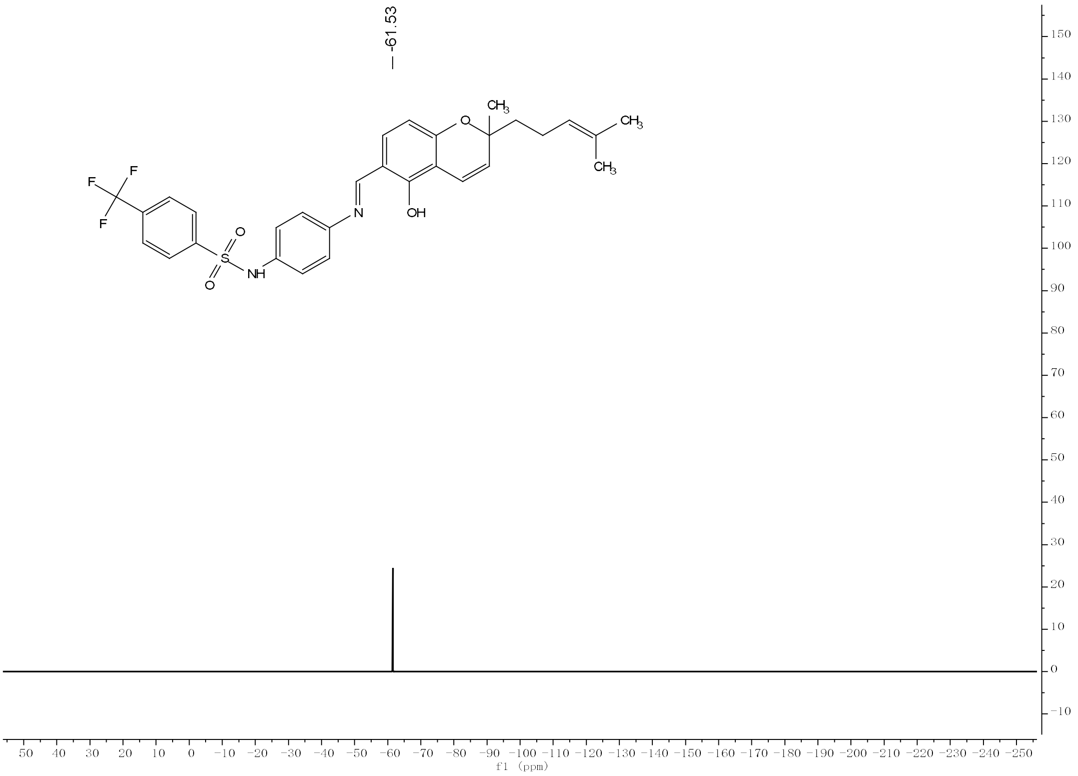

^{19}F NMR (471 MHz, DMSO-d_6) spectrum of compound **C46.**

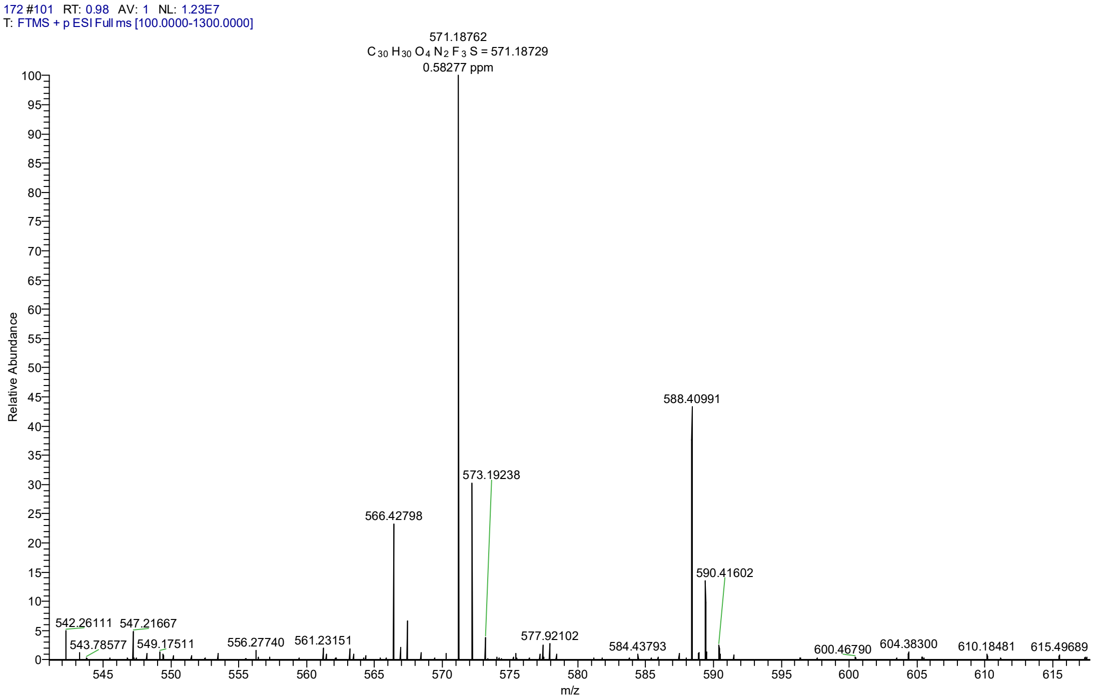

HRMS of compound **C46.**

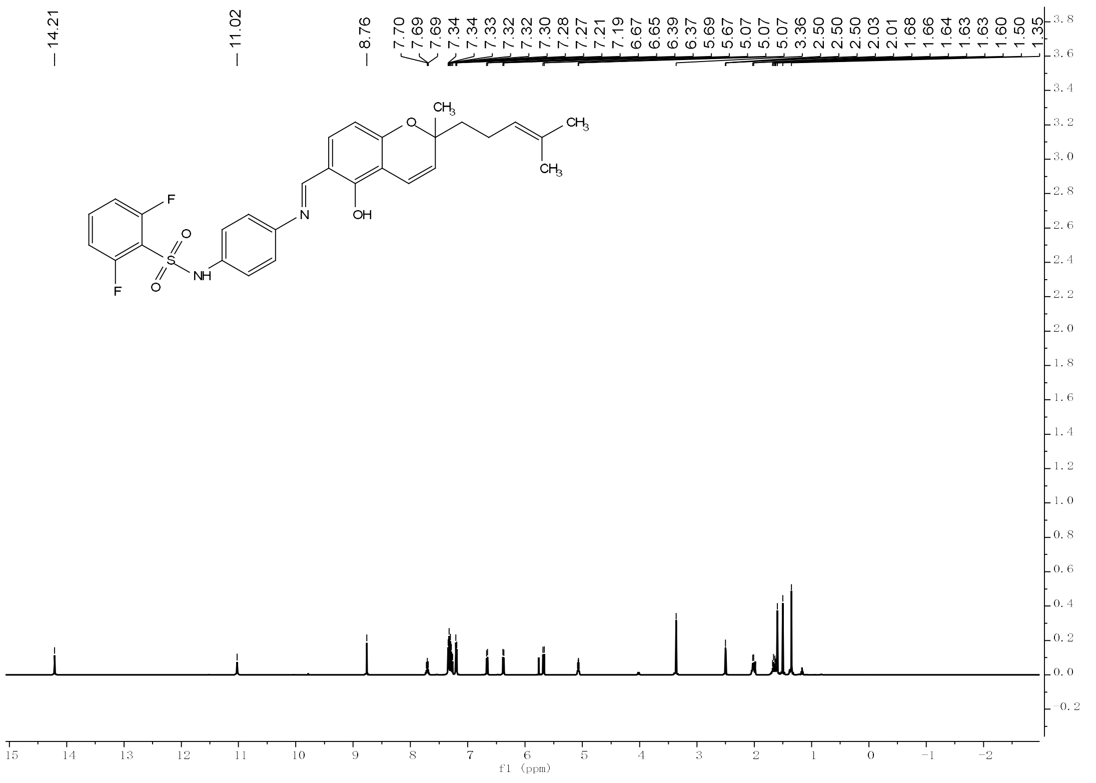

^{1}H NMR (500 MHz, DMSO-d_6) spectrum of compound **C47**.

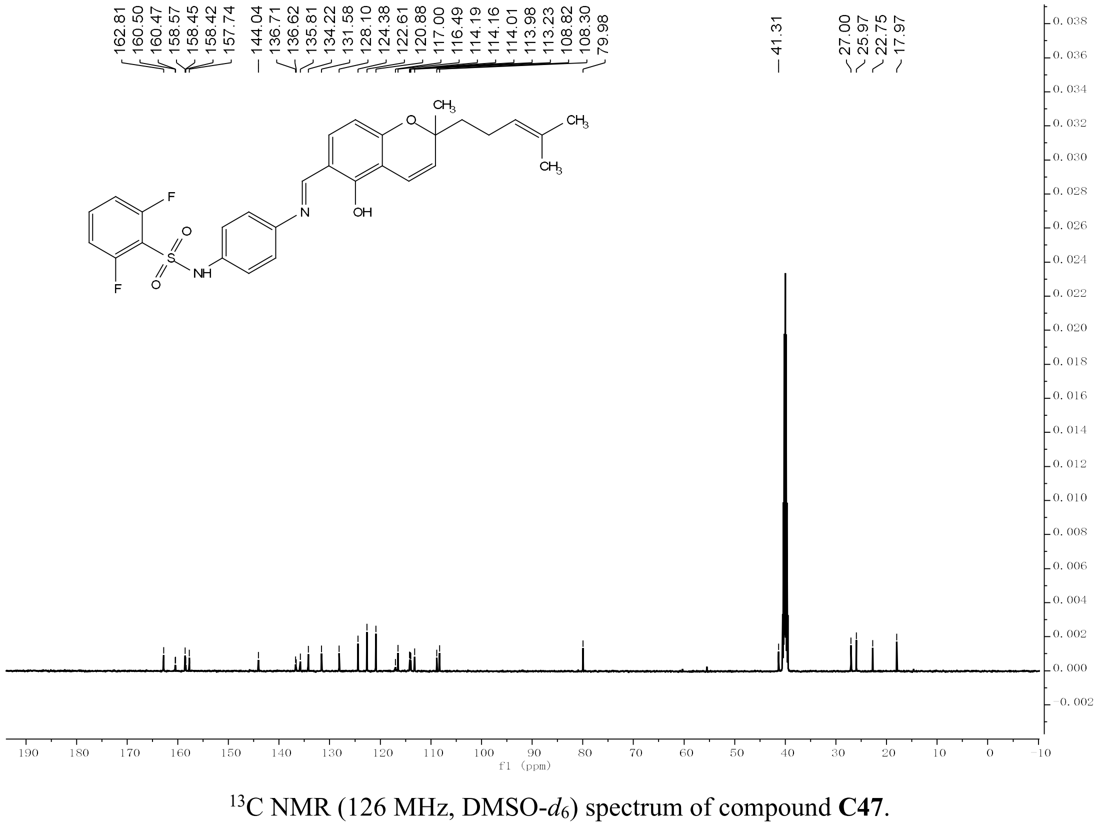

^{13}C NMR (126 MHz, DMSO-d_6) spectrum of compound **C47**.

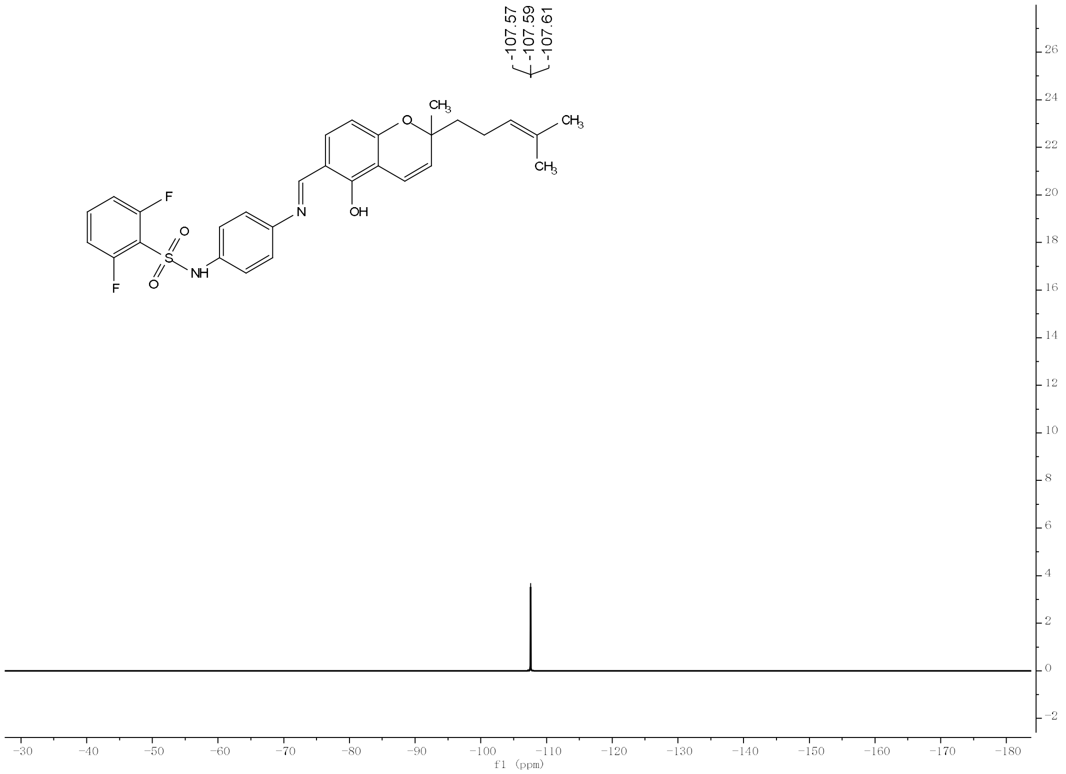

^{19}F NMR (471 MHz, DMSO-d_6) spectrum of compound **C47**.

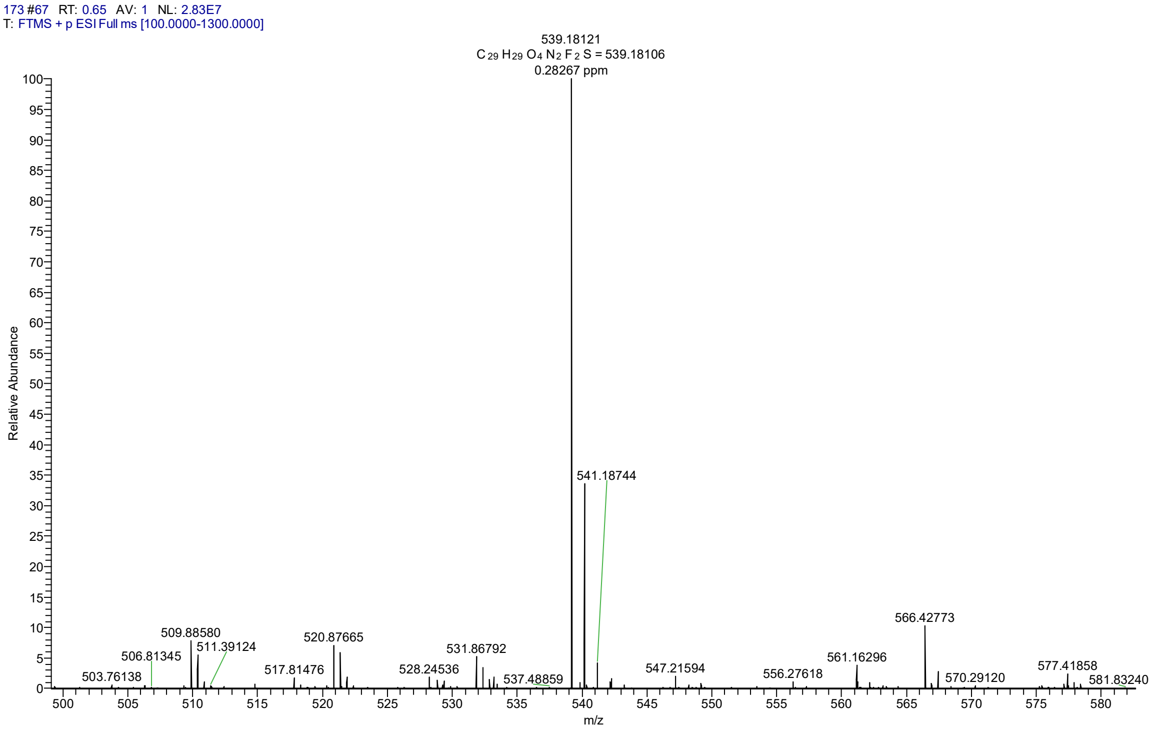

HRMS of compound **C47.**

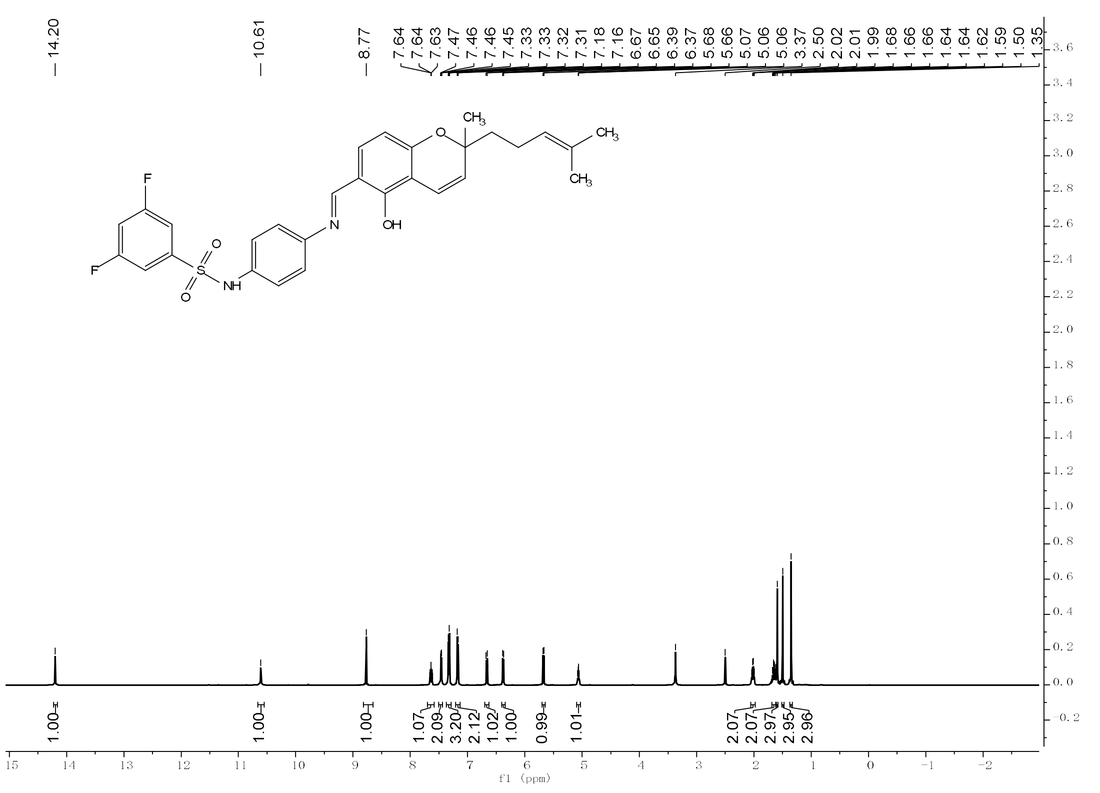

^{1}H NMR (500 MHz, DMSO-d_6) spectrum of compound **C48**.

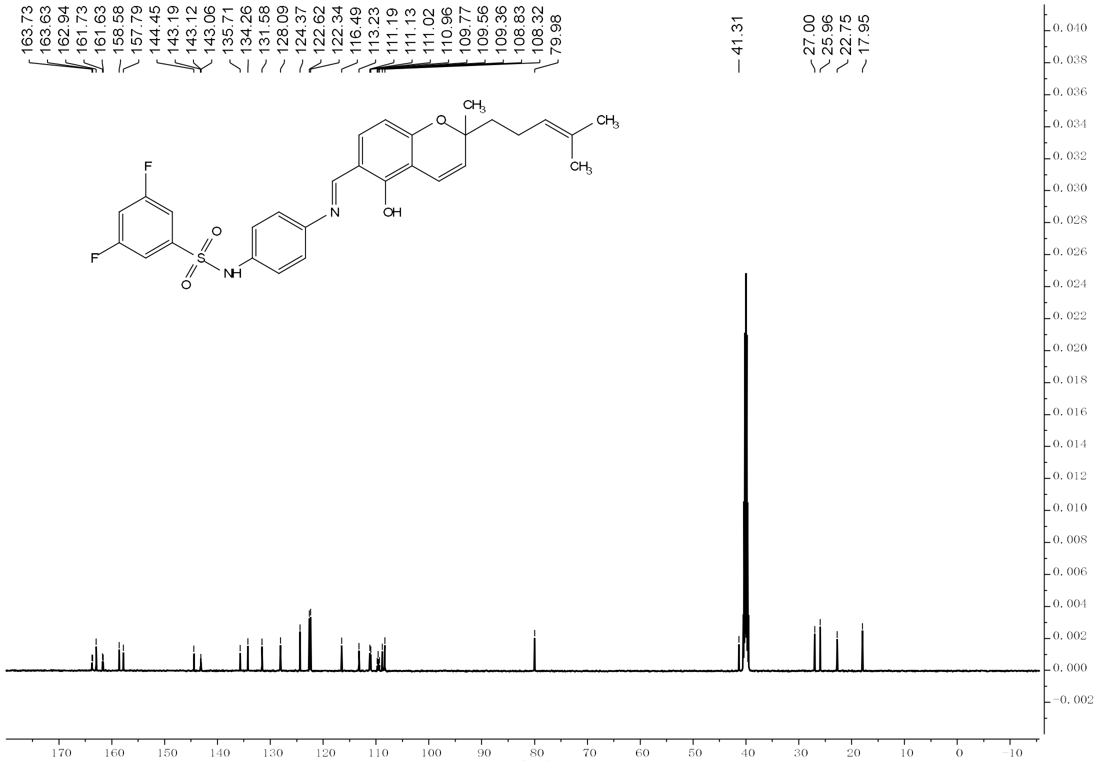

^{13}C NMR (126 MHz, DMSO-d_6) spectrum of compound **C48**.

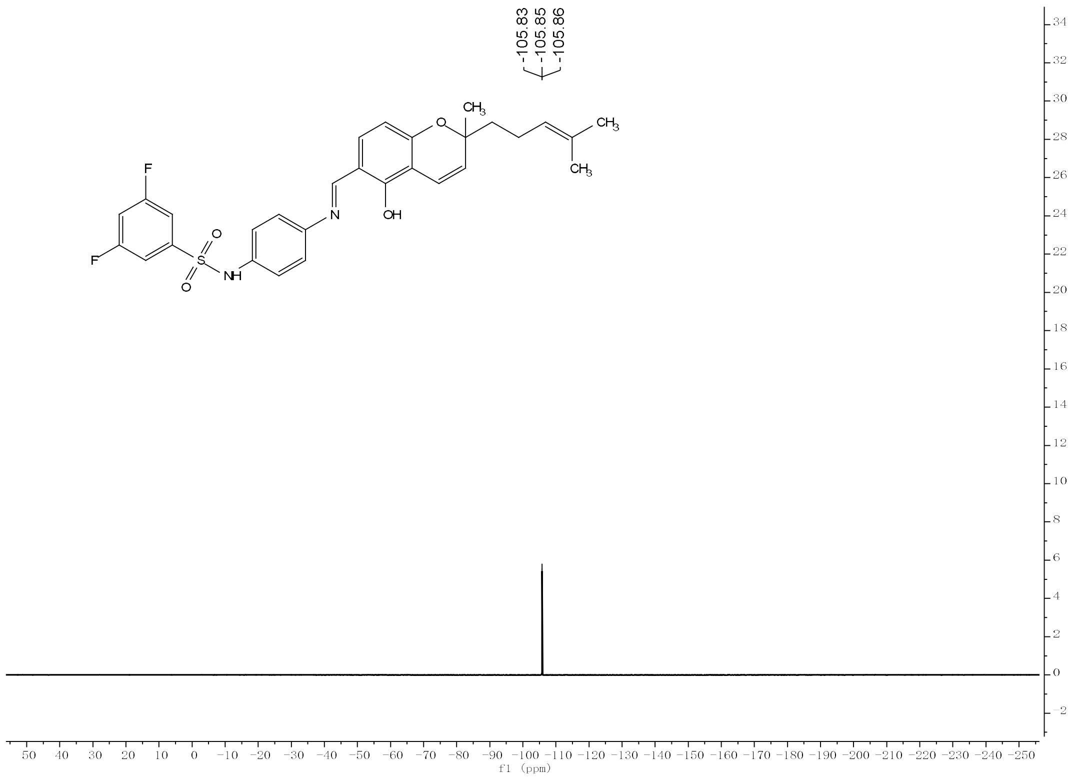

^{19}F NMR (471 MHz, DMSO-d_6) spectrum of compound **C48**.

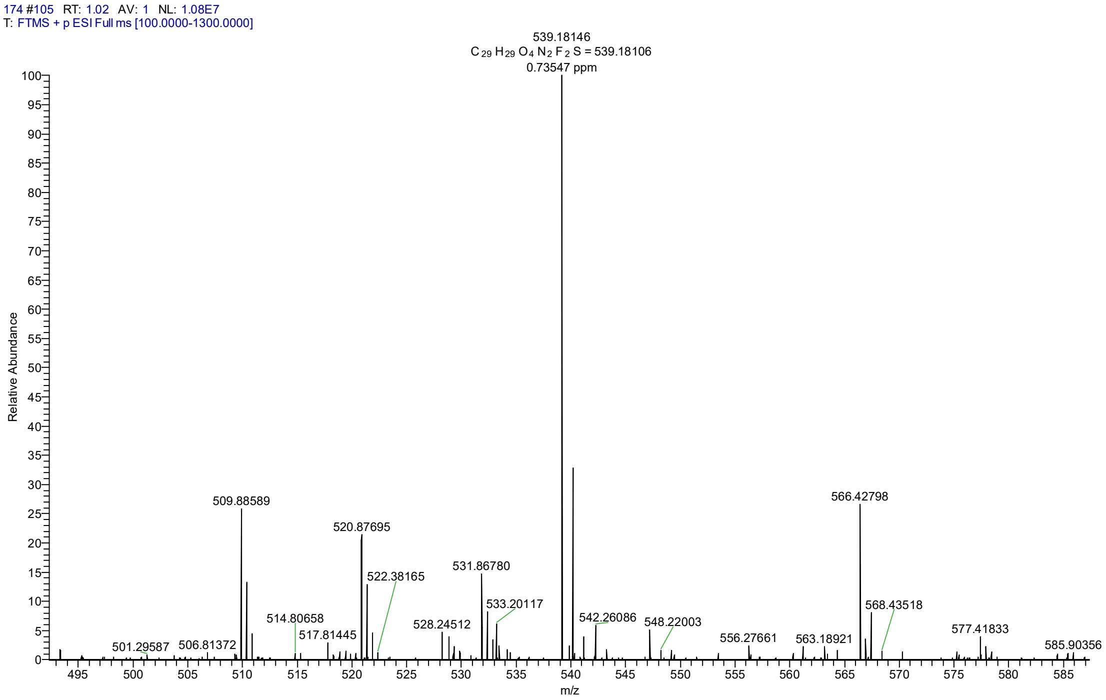

HRMS of compound **C48**.

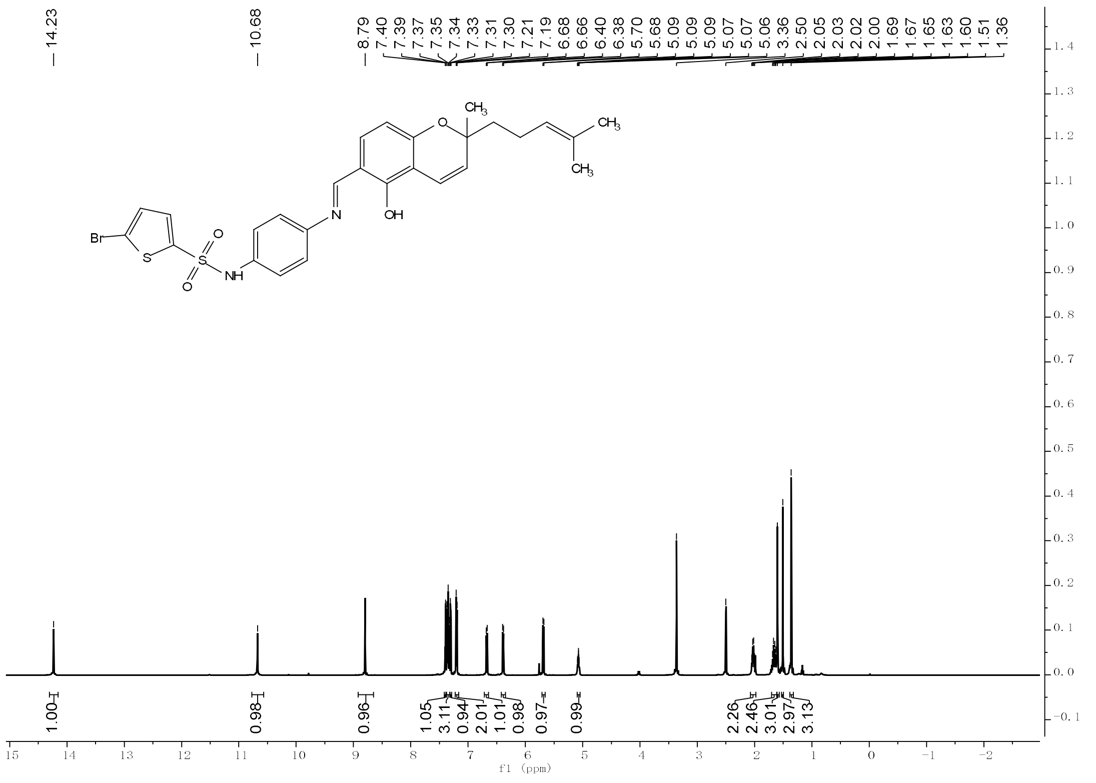

^{1}H NMR (500 MHz, DMSO-d_6) spectrum of compound **C49**.

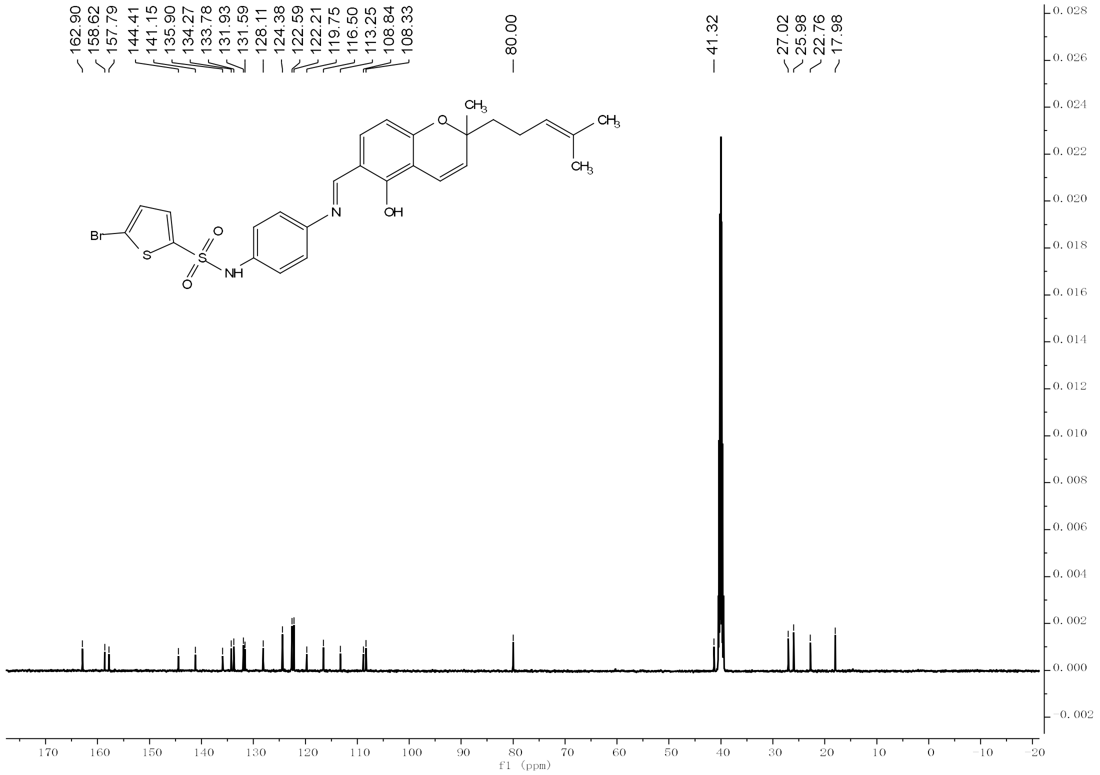

^{13}C NMR (126 MHz, DMSO-d_6) spectrum of compound **C49**.

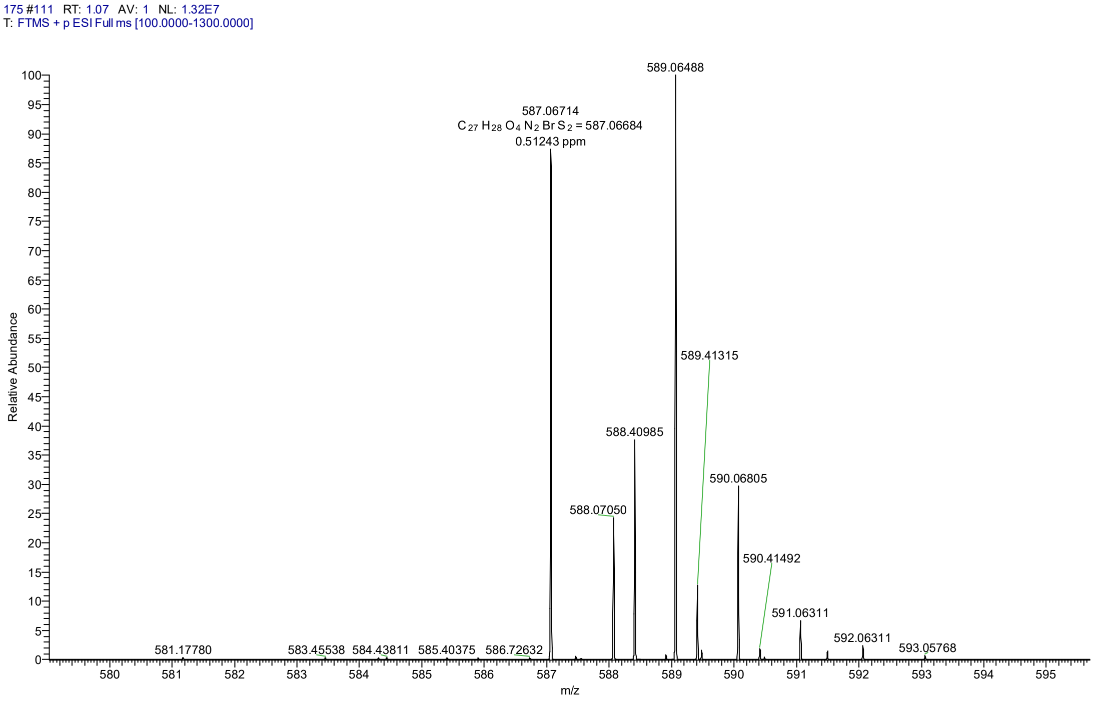

HRMS of compound **C49.**

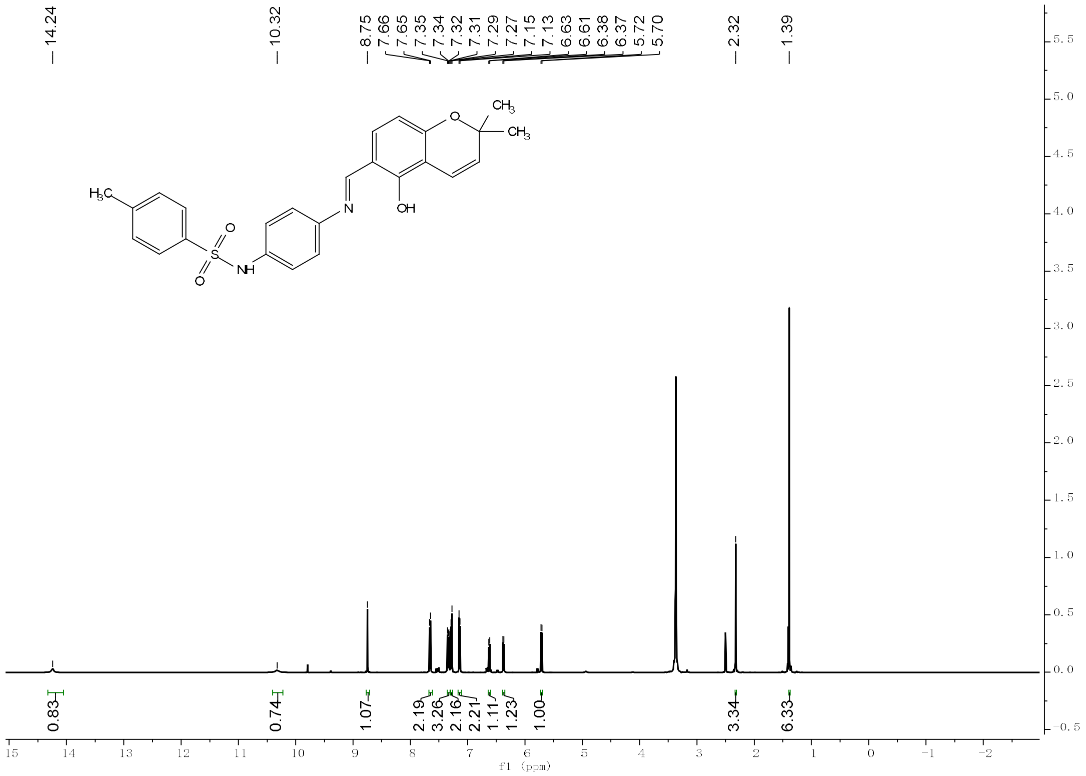

^{1}H NMR (500 MHz, DMSO-d_6) spectrum of compound **C50**.

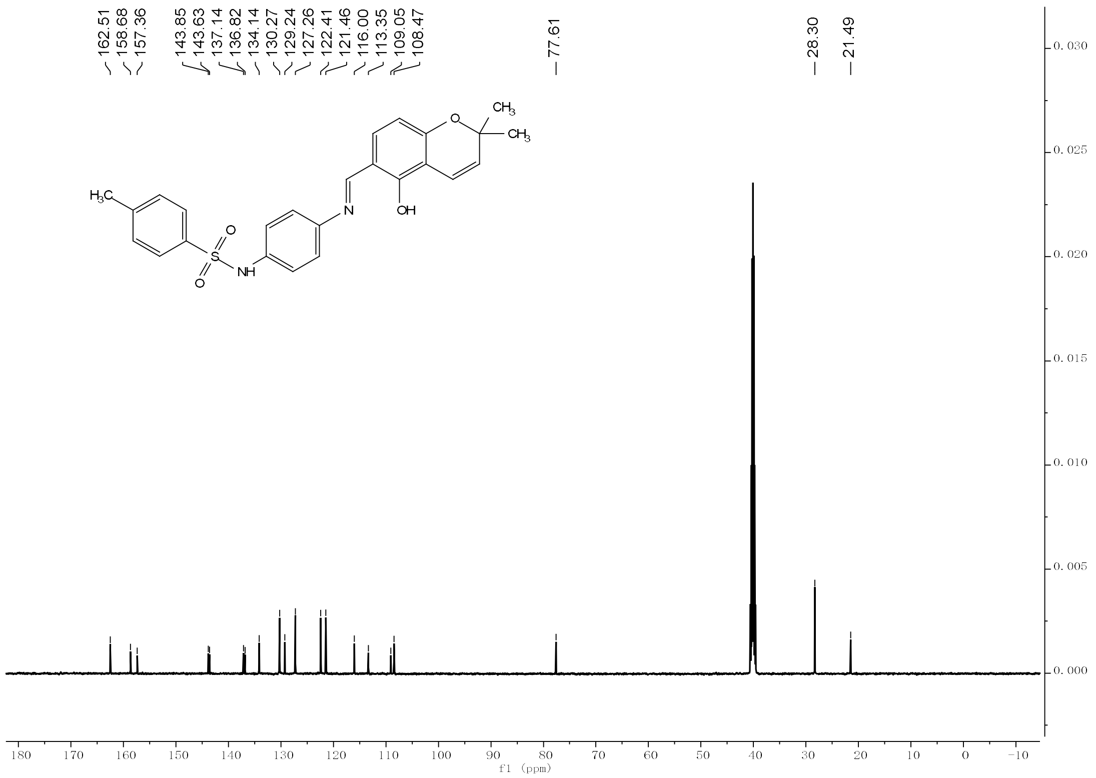

^{13}C NMR (126 MHz, DMSO-d_6) spectrum of compound **C50**.

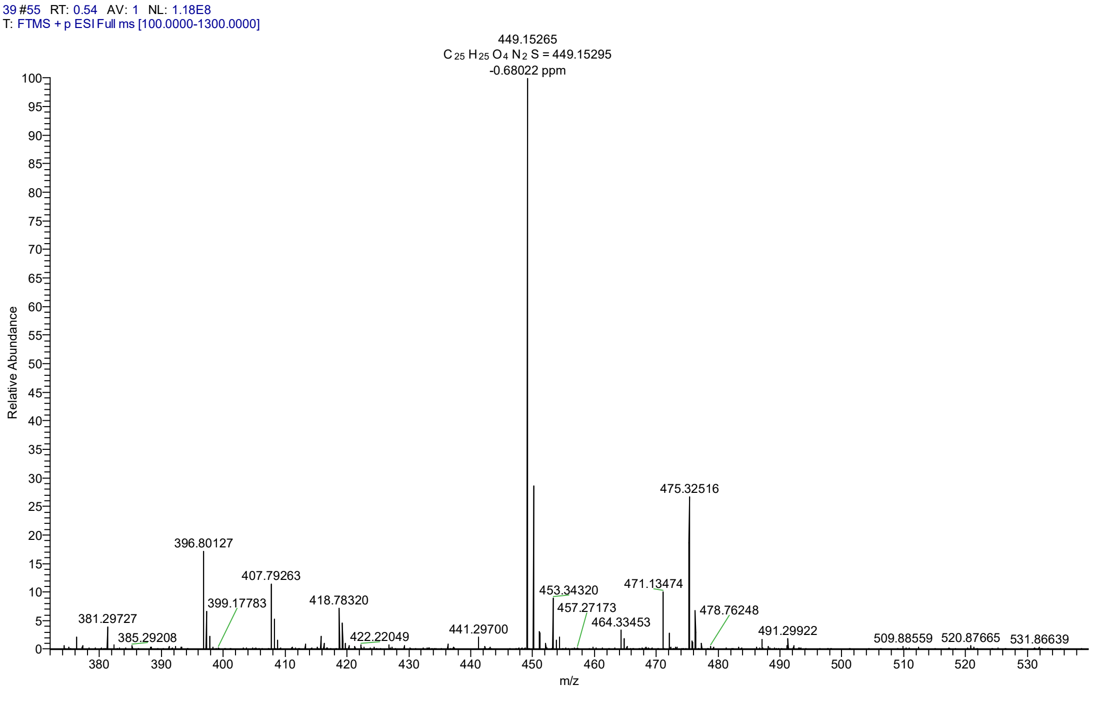

HRMS of compound **C50**.
